# Neighboring-Group Participation by a Less Electron-Donating, Participating C-2-Ester Ensures Higher 1,2-*trans* Stereoselectivity in Nucleophilic Substitution Reactions of Furanosyl Acetals

**DOI:** 10.1021/acs.joc.4c02612

**Published:** 2025-01-15

**Authors:** Yuge Chun, Wouter A. Remmerswaal, Jeroen D. C. Codée, K. A. Woerpel

**Affiliations:** a Department of Chemistry, New York University, 100 Washington Square East, New York, New York 10003, United States; b Leiden Institute of Chemistry, Leiden University, Einsteinweg 55, Leiden 2300 RA, The Netherlands

## Abstract

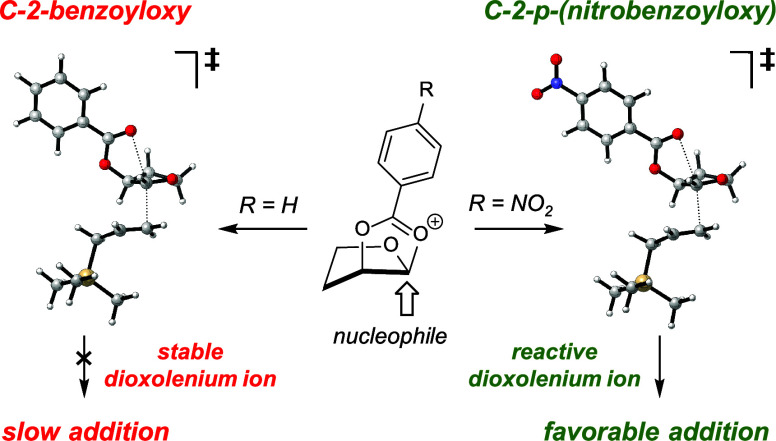

Nucleophilic substitution reactions of C-2-acyloxy furanosyl acetals can be highly diastereoselective. We here show that the presence of a less electron-donating *p*-nitrobenzoyloxy group at C-2 of a furanosyl acetal can be of use to control the 1,2-*trans* stereoselectivity of acetal substitution reactions with higher stereoselectivity than the analogue with the more electron-donating benzoyloxy group, just as what was observed in the pyranosyl system. Computational results support a reaction manifold involving both open oxocarbenium ions and *cis*-dioxolenium ions to provide the 1,2-*cis* and 1,2-*trans* products. Participation by the less electron-donating C-2-(*p*-nitrobenzoyloxy) group forms a less stabilized *cis*-dioxolenium ion that reacts with the incoming nucleophile more readily to provide 1,2-*trans* products. The relative stability of the furanosyl *cis*-dioxolenium ion versus the open oxocarbenium ion is much higher than the pyranosyl system as a result of the lower energy penalty for forming the *cis*-fused [5,5]-bicyclic dioxolenium ion.

## Introduction

**Figure 1:**
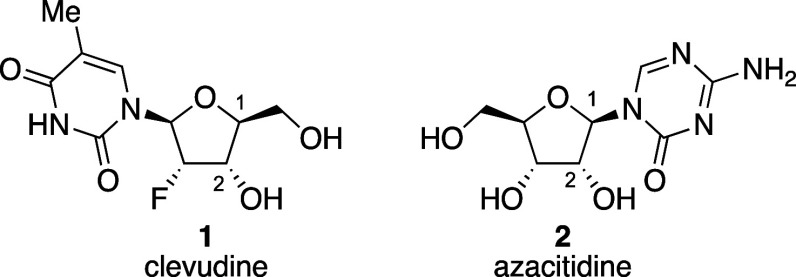
Bioactive compounds containing a furanosyl ring with the 1,2-*trans* configuration.

**Scheme 1:**
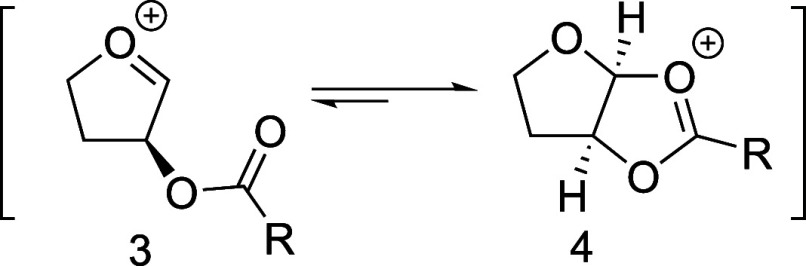
C-2-Acyloxy Groups Can Stabilize Oxocarbenium Ions **3** to Form *cis*-Dioxolenium Ions **4**

Tetrahydrofurans are important synthetic motifs in many structurally complex drugs and natural products (Figure 1).^1^ For example, clevudine **1** is a nucleoside antibiotic with potent inhibition to hepatitis B, and azacitidine **2** is an FDA-approved drug used for treatment of myelodysplastic syndromes. These drugs are representative examples of nucleoside antibiotics or anticancer drugs and consist of a ribose-derived scaffold with the anomeric functionalization in a 1,2-*trans* configuration. To construct such linkages with the correct stereochemical configuration, neighboring-group participation has been widely used.^2−4^ An acyloxy group at the C-2 position of a five-membered-ring acetal can stabilize an oxocarbenium ion (i.e., **3**) to form a *cis*-dioxolenium ion (i.e., **4**),^5,6^ and subsequent nucleophilic attack on this intermediate occurs from the sterically favored face of the bicyclic structure, resulting in selective formation of 1,2-*trans* products (Scheme 1).^7,8^

**Scheme 2:**
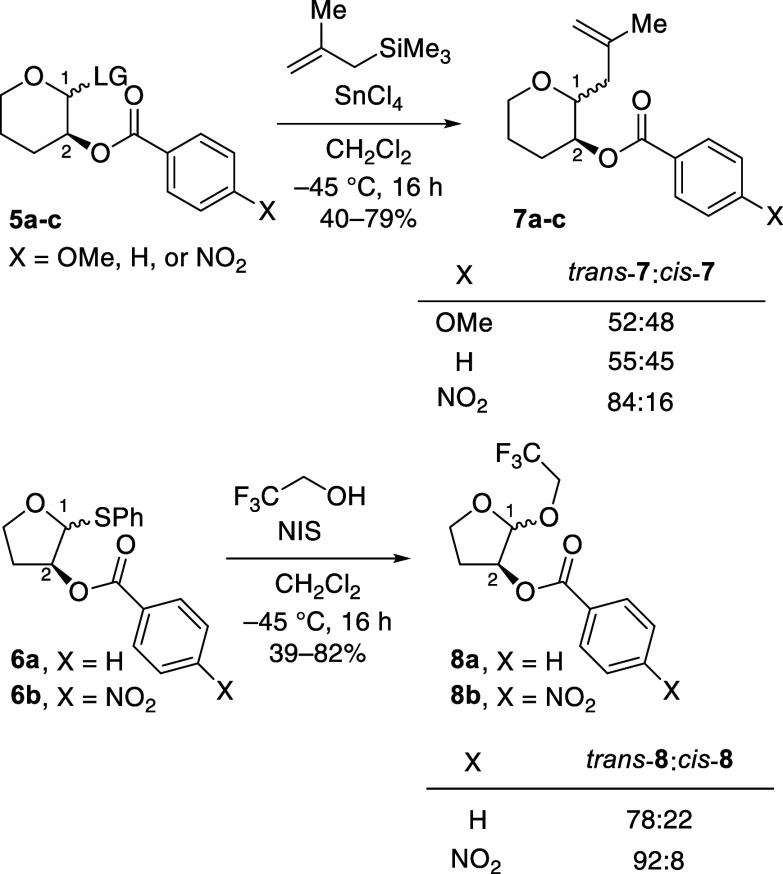
Higher 1,2-*trans* Selectivity with the Presence of a C-2-(*p*-Nitrobenzoyloxy) Group in Both the Pyran and Furan-Derived Systems

Our recent work has shown that C-2-acyloxy groups can engage in participation to promote the 1,2-*trans* selectivity of substitution reactions of pyran acetals, but the extent of stereochemical control is sensitive to the nature of the incoming nucleophile and the electron-donating ability of the neighboring group.^9^ The use of a less electron-donating C-2-(*p*-nitrobenzoyloxy) led to higher 1,2-*trans* selectivity than the use of the more electron-donating C-2-(*p*-methoxybenzoyloxy) or C-2-benzoyloxy groups (compounds **7a**-**c**, Scheme 2). Preliminary data suggest that this trend of higher 1,2-*trans* selectivity with decreasing electron-donating ability of acyloxy groups extends to furanosyl acetals (**8a** and **8b**, Scheme 2).

Considering that the findings of the six-membered-ring system can be extended to the furan system, we here follow up with this initial observation by investigating the role of neighboring-group participation to control the stereoselectivity of acetal substitution reactions of furanosyl acetals. In this study, we provide more evidence that the use of a *p*-nitrobenzoyloxy group at C-2 of tetrahydrofuran acetals is more effective in enabling diastereoselective substitution reactions. Substitution reactions of furanosyl acetals carrying a C-2-benzoyloxy group, which exhibited similar electron-donating and participating properties to the *p*-methoxybenozyloxy group,^9^ were performed as control experiments to determine the degree of stereoselectivity controlled by neighboring groups bearing different electron-donating properties. With all nucleophiles examined, higher 1,2-*trans* selectivity was observed in the substitution reactions of the C-2-(*p*-nitrobenzoyloxy) acetals than the corresponding C-2-benzoyloxy acetal analogues. The observed trends follow the stereochemical model used to analyze substitutions of six-membered-ring C-2-acyloxy acetals, where both oxocarbenium ions and *cis*-dioxolenium ions are involved as reactive intermediates in the stereochemistry-determining step.

## Results and Discussion

We started our investigation by exploring nucleophilic substitution reactions of furanosyl thioacetals bearing a single C-2-(*p*-nitrobenzoyloxy) or C-2-benzoyloxy group (i.e., **6a** and **6b**, respectively) with a range of oxygen nucleophiles (Table 1). In these reactions, consistent higher 1,2-*trans* selectivities with the less electron-donating *p*-nitrobenzoyloxy group at C-2 were observed (entries 1–6, Table 1). Reactions of the C-2-(*p*-nitrobenzoyloxy) thioacetal **6b** with 2,2,2-trifluoroethanol (**20a**), 2,2-difluoroethanol (**20b**), and 1,1,1,3,3,3-hexafluoroisopropanol (**20f**) proceeded with high 1,2-*trans* stereoselectivities (entries 1, 2, and 6, Table 1). Orthoester side-products (i.e., **14–19a,b**) were isolated in most cases, except for reactions using 1,1,1,3,3,3-hexafluoroisopropanol (**20f**). These bicyclic compounds are products formed by alcohol nucleophiles attacking the cationic carbon atom of the *cis*-fused dioxolenium ions (i.e., **4**, Scheme 1).^7,10^ Control experiments were performed to ensure that all of these observed diastereomeric ratios were the results of kinetically controlled reactions and that the orthoester side-products were not reactive intermediates that underwent rearrangement processes to provide 1,2-substituted products.^11^ The fact that reactions with isopropanol (**20e**) and 1,1,1,3,3,3-hexafluoroisopropanol (**20f**) proceeded with different selectivities (entries 5 and 6, Table 1), while the reactions with ethanol (**20d**) and isopropanol (**20e**) led to similar stereochemical outcomes (entries 4 and 5, Table 1) suggests that the steric hindrance presented by the incoming nucleophiles does not affect the stereochemical outcomes of the acetal substitutions. Considering that the reactivity and the size of oxygen nucleophiles **20a–f** are similar to secondary carbohydrate-derived glycosyl acceptors, the observed diastereomeric ratios reveal that the installation of a C-2-(*p*-nitrobenzoyloxy) group can be useful to promote 1,2-*trans* stereoselectivity in *O*-glycosylation reactions.^12,13^

**Table 1:**
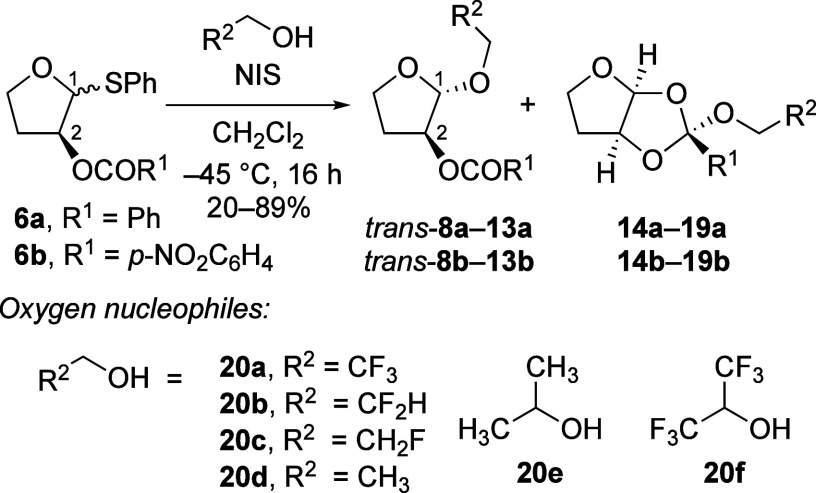
Nucleophilic Substitution Reactions of C-2-Acyloxy Thioacetals with Oxygen Nucleophiles^a^^,^^b^

Entry	Nucleophile	dr (*trans*:*cis*)^c^ (**8a**–**13a**, R^1^ = Ph)	dr (*trans*:*cis*)^c^ (**8b**–**13b**, R^1^ = *p*-NO_2_C_6_H_4_)
1	**20a**	78:22	92:8
2	**20b**	93:7	≥97:3
3	**20c**	65:35	75:25
4	**20d**	70:30	80:20
5	**20e**	76:24	80:20
6	**20f**	≥97:3	≥97:3

a Thioacetals **6a** and **6b** were used as a mixture of diastereomers. The diastereomer ratio of the starting material should not influence the stereochemical outcomes of acetal substitution reactions.

b Details of control experiments are provided in the Supporting Information.

c Diastereomeric ratios were determined by ^13^C{^1^H} NMR spectroscopic analysis of the unpurified reaction mixtures.^14^

As illustrated for reactions using 2,2,2-trifluoroethanol (**20a**) as the nucleophile, consistent 1,2-*trans* stereoselectivities were observed in the substitution reactions of C-2-(*p*-nitrobenzoyloxy) thioacetal **6b** (Table 2). The stereoselectivity does not significantly depend upon modifications of the reaction conditions such as the choice of activators, solvents, or temperature. Reactions with or without triflate counterions occurred with similar stereoselectivities, indicating that anomeric triflates are likely not involved as reactive intermediates in the stereochemistry-determining step (entries 1 and 2, Table 2). Orthoester side-product **15b** was not observed in the reaction promoted by Me_3_SiOTf, indicating that the orthoester compound likely underwent decomposition under the triflic acid-catalyzed conditions.^15^ The increased preference for the formation of the 1,2-*cis* product upon the use of *N*-bromosuccinimide (NBS) as the promotor for the activation of the thiophenol group hints at the possible involvement of an anomeric bromide under these conditions (entry 3, Table 2).^16^ Changes in the temperature or the concentration of the reactions did not alter the diastereomeric ratios (entries 4 and 5, Table 2). Reactions performed in nonpolar solvents such as CH_2_Cl_2_ and toluene favored the formation of 1,2-*trans* products, even at an elevated temperature (entries 5 and 6, Table 2). Upon changing the solvent to a more polar solvent (CH_3_CN), the preference for the formation of 1,2-*trans* products decreased (entry 6, Table 2). The moderate change in selectivity, however, does not support the intermediacy of nitrilium ions as the major product-forming intermediate, considering that the S_N_2-like attack on a nitrilium ions should lead to a reaction favoring the 1,2-*trans* products.^17−20^ Rather, the observed stereochemical outcomes suggest that the use of more polar solvents favors the formation of the oxocarbenium ions, in which the positive charge is less stabilized than in the dioxolenium ions, leading to the formation of more 1,2-*cis* products.

**Table 2:**
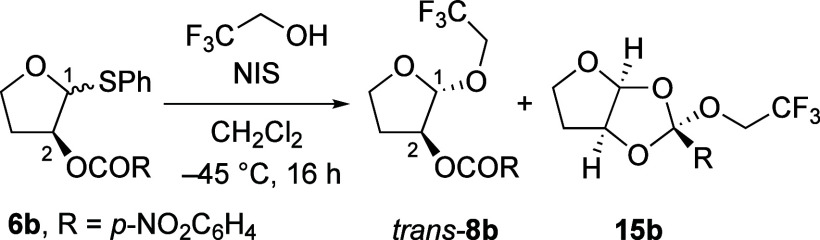
Influence of Reaction Condition on Selectivity of Substitution Reactions of Thioacetal **6b** with 2,2,2-Trifluoroethanol^a^

Entry	Solvent	Activator	Temperature (°C)	dr^b^ (**8b**, *trans*:*cis*)
1	CH_2_Cl_2_	NIS	–45	92:8
2	CH_2_Cl_2_	NIS/Me_3_SiOTf	–45	95:5
3	CH_2_Cl_2_	NBS	–45	65:35
4^c^	CH_2_Cl_2_	NIS	–45	≥97:3
5	CH_2_Cl_2_	NIS	–45 to 23	92:8
6	PhMe	NIS	–45 to 23	82:18
7	MeCN	NIS	–45 to 23	57:43

a Thioacetal **6b** was used as a mixture of diastereomers. The diastereomer ratio of the starting material should not influence the stereochemical outcomes of acetal substitution reactions.

b Diastereomeric ratios were determined by ^13^C{^1^H} NMR spectroscopic analysis of the unpurified reaction mixtures.^14^

c Reaction was conducted at 0.05 M in CH_2_Cl_2_.

To establish the correlation between the stereoselectivity and the electron-donating ability of the neighboring group, acetals **21a** and **21b**, bearing a single C-2-benzoyloxy group or C-2-(*p*-nitrobenzoyloxy) group, respectively, were treated with different carbon nucleophiles (Table 3).^21,22^ The set of carbon nucleophiles, including allylchlorodimethylsilane (**23**), allyltrimethylsilane (**24**), and allyltributylstannane (**25**), was selected because it consists of small nucleophiles that react irreversibly with carbocations to afford the same products, which simplifies the analysis of selectivity.^23,24^

**Table 3:**
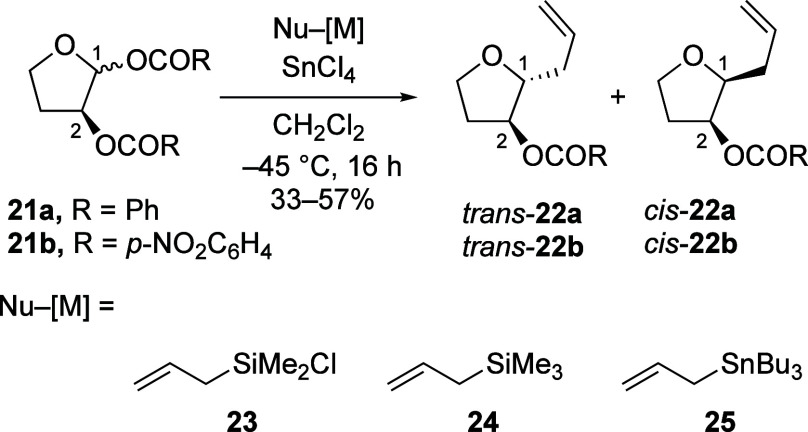
Nucleophilic Substitution Reactions of C-2-Acyloxy Acetals with Carbon Nucleophiles^a^

Entry	Nu–[M]	*N* Number^b^	dr (*trans*:*cis*)^c^ (**22a**, R = Ph)	dr (*trans*:*cis*)^c^ (**22b**, R = *p-*NO_2_C_6_H_4_)
1	**23**	–0.6	–d	61:39
2^e^	**23**	–0.6	70:30	75:25
3	**24**	1.7	55:45	70:30
4^f^	**25**	5.5	87:13	90:10

a Reaction conditions were adapted from the previously optimized reaction conditions.^9^ Acetals **21a** and **21b** were used as a mixture of diastereomers. The diastereomer ratio of the starting material should not influence the stereochemical outcomes of acetal substitution reactions.

b Higher *N* numbers correspond to higher reactivity of the nucleophiles.

c Diastereomeric ratios were determined by ^13^C{^1^H} NMR spectroscopic analysis of the unpurified reaction mixtures.^14^

d No formation of the expected product (**22a**) was observed. Instead, a 1,2-*trans* acetal product carrying an anomeric methoxy group was obtained as the sole product, which formed upon quenching the reaction with MeOH as −45 °C.^25^

e Reactions were warmed to 23 °C for efficient nucleophilic additions.

f BF_3_•OEt_2_ was used as the activator because SnCl_4_ was not able to activate acetals in the presence of allyltributylstannane. Reactions were warmed to 23 °C for efficient activation of the acetal leaving group.

Just as observed for the six-membered-ring system, substitution reactions of the furanosyl acetal bearing the less electron-donating C-2-(*p*-nitrobenzoyloxy) group occurred with higher 1,2-*trans* selectivities than those of the analogous C-2-benzoyloxy acetal, although the difference in selectivity was moderate.^9,26^ The substitution reaction of C-2-benzoyloxy acetal **21a** with allyltrimethylsilane (**24**) afforded acetal products **22a** as an equal mixture of diastereomers. The preference for the formation of the 1,2-*trans* product increased when the benzoyloxy group at C-2 was replaced by the less electron-donating *p*-nitrobenzoyloxy group, although the overall stereoselectivity was still moderate (entry 3, Table 3). When a stronger nucleophile, allyltributylstannane (**25**), was used, the reactions with both substrates achieved the expected high 1,2-*trans* selectivities (entry 4, Table 3).^11^ The trend of increasing 1,2-*trans* selectivity with increasing reactivity of the nucleophiles is consistent with the argument that strong nucleophiles can attack the less electrophilic *cis*-dioxolenium ions (i.e., **4**, Scheme 1) more readily to form the desired 1,2-*trans* products.^9^

The influence of the solvent on the stereochemical outcome was examined with acetal substitution reactions of C-2-(*p*-nitrobenzoyloxy) acetal **21b** (Table 4). In line with the results obtained with the *O*-nucleophiles (entries 5–7, Table 2), the preference for the formation of the 1,2-*trans* products decreased with increasing polarity of the solvents (Table 4).^27^ Nonpolar solvents such as toluene can be of use to control 1,2-*trans* selectivity, particularly when substitution reactions require high reaction temperatures (entry 1, Table 4).^28^ The fact that the allylations of acetal **21b** in the most polar solvent (acetonitrile) occurred with the lowest stereoselectivity again argues against the participating effect of the nitrile solvent, involving an α-nitrilium ion intermediate, which should favor the formation of 1,2-*trans* products (entry 3, Table 4).^17−20^

**Table 4:**
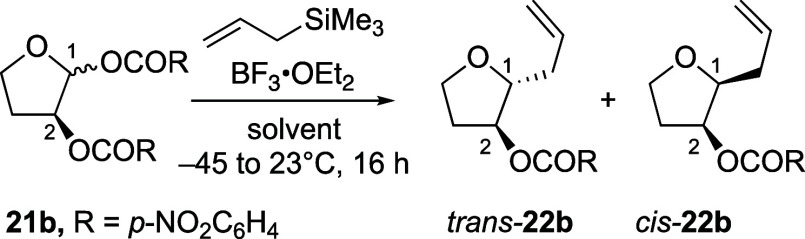
Influence of the Choice of the Solvent on the Stereoselectivity of Substitution Reactions of C-2-(*p-*Nitrobenzoyloxy) Acetal **21b** with Allyltrimethylsilane^a^

Entry	Solvent	Dielectric constant (ε)^b^	Lewis acid	dr (*trans*:*cis*)^c^ (**22b**)
1^d^	PhMe	2.38	BF_3_•OEt_2_	70:30
2^d^	CH_2_Cl_2_	8.93	BF_3_•OEt_2_	60:40
3^d^	MeCN	37.5	BF_3_•OEt_2_	58:42

a Acetal **21b** was used as a mixture of diastereomers. The diastereomer ratio of the starting material should not influence the stereochemical outcomes of acetal substitution reactions.

b Higher dielectric constant (ε) corresponds to higher solvent polarity.^27^

c Diastereomeric ratios were determined by ^13^C{^1^H} NMR spectroscopic analysis of the unpurified reaction mixtures.^14^

d Reactions were warmed to 23 °C for efficient activation of the leaving group by BF_3_•OEt_2_.

**Figure 2:**
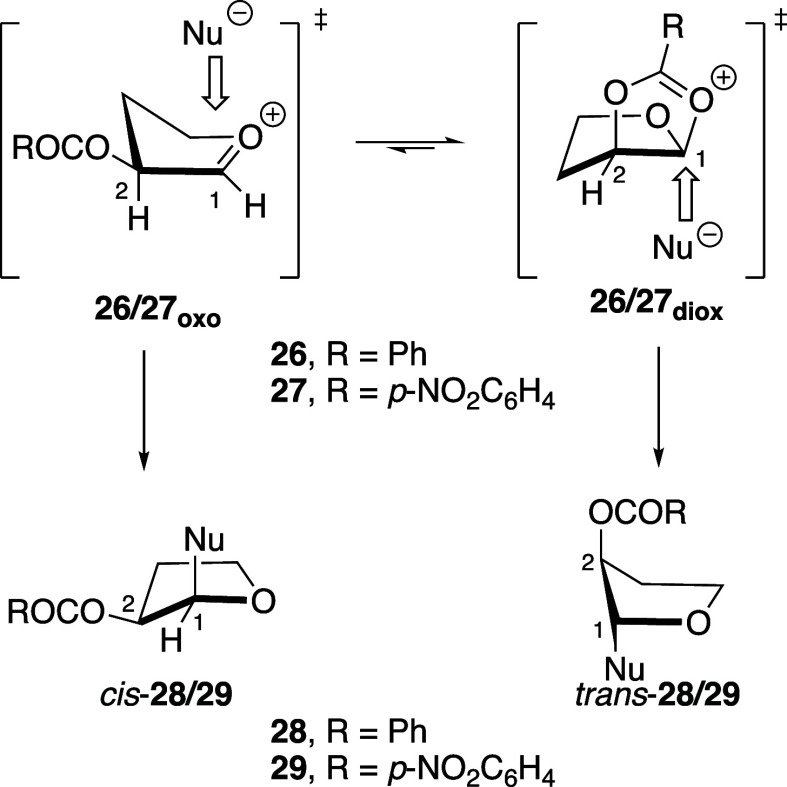
Nucleophilic additions to the oxocarbenium and dioxolenium forms of cations **26** and **27**.

Taken together, the experimental results above suggest that the presence of both oxocarbenium ions **26**_**oxo**_/**27**_**oxo**_ and *cis*-fused dioxolenium ions **26**_**diox**_/**27**_**diox**_ in the reaction mixtures should be considered to explain the formation of products *cis*-**28**/**29** and *trans*-**28**/**29** (Figure 2). Nucleophiles can react with the “open” oxocarbenium intermediates from the direction considered to be the inside face of the envelope conformers to minimize the development of eclipsing interactions in the products, allowing for the formation of 1,2-*cis* products.^23,29^ Experimental observation of orthoester side-products confirms the existence of *cis*-dioxolenium ions **26**_**diox**_ and **27**_**diox**_.^10^ The observed higher 1,2-*trans* selectivity in the presence of a C-2-(*p*-nitrobenzoyloxy) group is consistent with faster nucleophilic additions to the less stabilized *cis*-dioxolenium ion **27**_**diox**_ from the sterically more accessible face of the bicyclic intermediate.

To provide further support for the roles of oxocarbenium ions and *cis*-dioxolenium ions in controlling the selectivity of substitution reactions of furanosyl acetals, we investigated the shape and stability of the furanosyl cations and the subsequent addition reactions to these cations computationally. All computations were performed with ORCA5.03^30,31^ at the SMD(dichloromethane)-revDSD-PBEP86D4-def2TZVPP//PCM-(dichloromethane)-B3LYP-D3BJ-def2TZVPP^32−40^ level of theory, which previously provided accurate assessments of the energy of oxocarbenium ions.^9,41^

**Figure 3:**
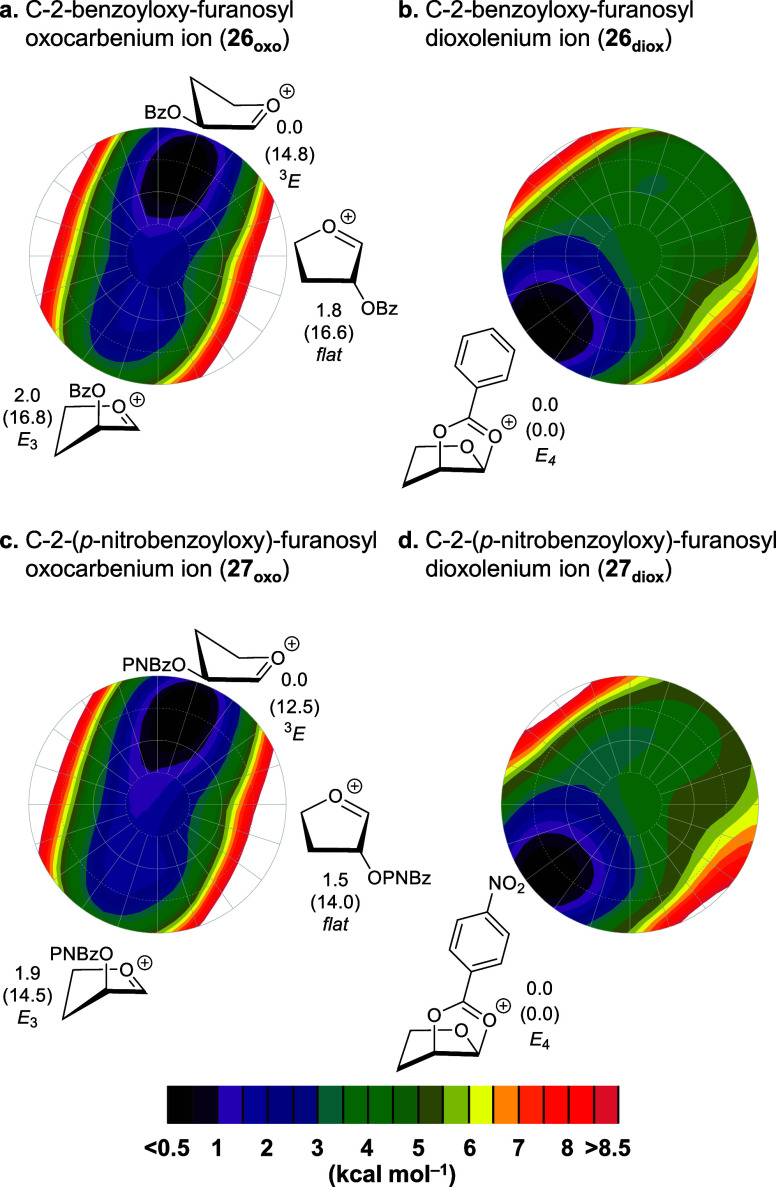
Conformational energy landscape (CEL) maps of C-2-benzoyloxy and C-2-(*p*-nitrobenzoyloxy) furanosyl oxocarbenium and dioxolenium ions in which the local minima identified are shown with their respective energy. Energies of all conformations in the CEL are computed at SMD(dichloromethane)-revDSD-PBEP86-D4-def2TZVPP//PCM(dichloromethane)-B3LYP-D3BJ-def2TZVPP and expressed as relative Gibbs free energy (*T* = 228.15 K) in kcal mol^–1^. Energies given are relative to the *cis*-dioxolenium ion. The CEL map of the C-2-methoxy furanosyl oxocarbenium ion is provided in the Supporting Information.

First, we investigated the overall shape of the cations by conformational energy landscape mapping (Figure 3). This methodology calculated the energies of the ions mapped as a function of their shape.^9,42−46^ The computed CEL maps indicate that furanosyl oxocarbenium ions **26**_**oxo**_ and **27**_**oxo**_, the lowest-energy conformations of the furanosyl oxocarbenium ions, favor a ^3^*E* conformation in which the C-2 neighboring group is positioned in a *pseudo*-equatorial orientation, allowing the *pseudo*-axial H-2 to provide stabilization of the cationic center through donation of electron density from the σ_C–H_ bond.^47^ Notably, the ring-flipped *E*_3_-counterpart, in which the C-2 group is positioned in a *pseudo*-axial fashion, is always higher in energy than the corresponding flat furanosyl oxocarbenium ion structures (located in the center of the CEL maps). In all cases, the *cis*-dioxolenium ions are significantly more stable than their oxocarbenium ion counterparts. As the electron-donating nature of the acyloxy groups decreases, the *cis*-dioxolenium ions become less stabilized. The relationship between the stability of *cis*-dioxolenium ions and the electron-donating ability of the acyloxy groups is in line with our previous investigations of pyranosyl cations,^9^ in which we established that the higher 1,2-*trans* stereoselectivity, observed in addition reactions of the pyranosyl electrophiles carrying the more electron-withdrawing C-2-esters, correlates with the less stabilized dioxolenium ions, which are more readily substituted by the incoming nucleophiles.

We note that the relative stability of the *cis*-dioxolenium ion versus the oxocarbenium ion is much higher in the furanosyl system (for the C-2-benzyloxy and C-2-(*p*-nitrobenzoyloxy), 14.8 and 12.5 kcal mol^–1^, respectively) than in the pyranosyl ions (for the C-2-benzyloxy and C-2-(*p*-nitrobenzoyloxy), 11.2 and 9.0 kcal mol^–1^, respectively).^9^ The difference in stability could be the result of lower energy penalty for forming the *cis*-fused [5,5]-bicyclic dioxolenium ion compared to the corresponding *cis*-fused [5,6]-bicyclic ion.^48^ In contrast to the pyranosyl cations, where the *cis*-dioxolenium ion is located in the same region of conformational space as the most stable oxocarbenium ion, and which thus requires only a minimal conformational change to position the C-2-substituent optimally for formation of the dioxolenium ion,^9^ the interconversion of the most favorable furanosyl oxocarbenium ion and the corresponding dioxolenium ion requires a more significant conformational change. The furanosyl oxocarbenium ion must first undergo a ring-flip to reorient the C-2 group into a *pseudo*-axial position before the formation of the dioxolenium ion can occur.

**Figure 4:**
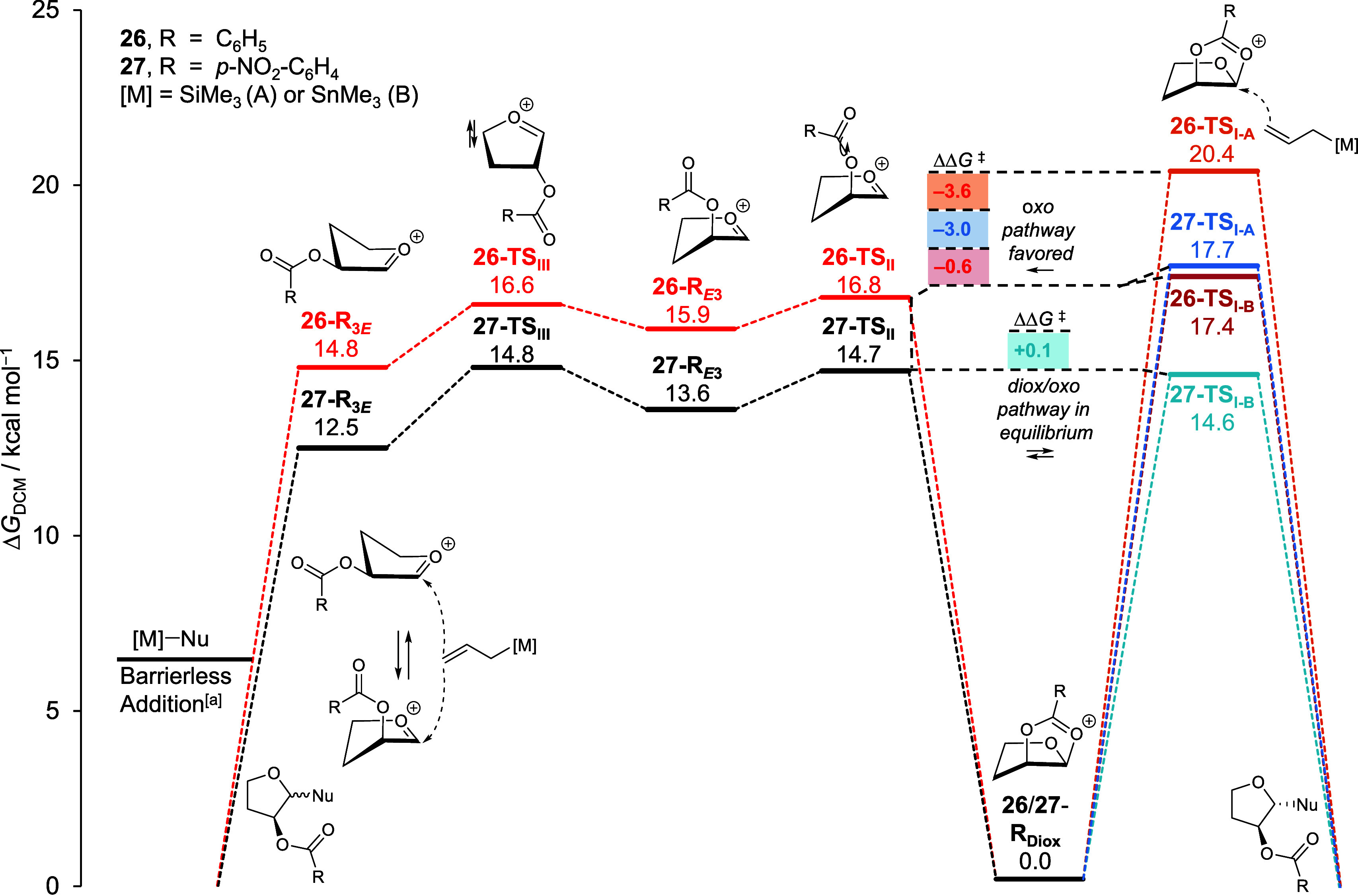
Reaction profiles of nucleophilic substitution reactions of C-2-benzoyloxy furan cation **26** and C-2-*p*-(nitrobenzoyloxy) furan cation **27** with *C*-nucleophiles **24** (**A**) and **25** (**B**). ^[b]^Computed at SMD(dichloromethane)-revDSD-PBEP86-D4-def2TZVPP//PCM(dichloromethane)-B3LYP-D3BJ-def2TZVPP and expressed as relative Gibbs free energy (*T* = 228.15 K) in kcal mol^–1^. ^[a]^Electronically barrierless, determined by constrained PES analysis. ^[b]^The arrow in transition states **26**/**27**-TS_II_ is an indication of bond rotation. The butyl groups in nucleophile **25** were replaced with the smaller methyl group, which are electronically similar, for computational feasibility.

We next investigated the influence of nucleophilicity and the electron-donating capacity of the C-2-acyloxy group on the stereochemical outcome by computing the reaction profiles for the addition of nucleophiles **24** and **25**(49) to furanosyl cations **26** and **27**. For these investigations, carbon nucleophiles were chosen because their reaction pathways have been extensively investigated using both computational and experimental techniques,^9,41,50,51^ generally showing good correlation.^9^ Analogous to our work on pyranosyl cations, the computed reaction profiles suggest two reaction pathways from the lowest energy dioxolenium ions: direct attack to the *cis*-dioxolenium ions (**TS**_**I**_) to afford the 1,2-*trans* products (right pathway in Figure 4) or ring-opening to form furanosyl oxocarbenium ions (via **TS**_**II**_), generating a mixture of possible conformers followed by nucleophilic additions to these ions (left pathway in Figure 4).

We then examined the nucleophilicity-stereoselectivity relationships (Figure 4). For the weakest nucleophile **24**, the energy barrier for the nucleophile to react with *cis*-dioxolenium ions (**26**/**27**-**TS**_**I-A**_, right pathway) is computed to be higher than the transition state leading to the formation of the “open” oxocarbenium ions (**26**/**27**-**TS**_**II**_, left pathway). This difference indicates that the reaction more readily proceeds through ring-opening of the *cis*-dioxolenium ion to provide the oxocarbenium ion, which is followed by a barrierless nucleophilic addition to this ion^9,52^ to form the products as a mixture of diastereomers. With increasing reactivity of the incoming nucleophile, the transition states **26**/**27**-**TS**_**I–B**_ become more favorable, enhancing the direct substitution pathway (right pathway), leading to the formation of more 1,2-*trans* product, in agreement with the experimental observations.

Finally, the calculations show that the difference in the energy barriers for the formation of the oxocarbenium ions (left pathway) and nucleophilic attack on the *cis*-dioxolenium ions (right pathway) for C-2-(*p*-nitrobenzoyloxy) ions **27** are modestly smaller than for the corresponding C-2-benzoyloxy ions **27**. For C-2-benzoyloxy ion **26**, direct addition to the dioxolenium ion (**26**-**TS**_**I–B**_) is less favorable than formation of the oxocarbenium ion (**26**-**TS**_**II**_). In contrast, for the C-2-(*p*-nitrobenzoyloxy) ions **27**, these two transition states are similar in energy (**27**-**TS**_**IB**_ and **27**-**TS**_**II**_). The computed reaction profiles thus indicate that more 1,2-*trans* products would be formed through the *cis* dioxolenium ions carrying a *p*-nitrobenzoyloxy group at C-2 because the right pathway is less disfavored. To summarize, the computational assessment agrees with the higher 1,2-*trans* selectivities observed in reactions with the C-2-(*p*-nitrobenzoyloxy) substrates.

## Conclusions

In conclusion, neighboring-group participation by C-2-acyloxy groups can guide the stereoselectivities of acetal substitutions in the furanosyl system. Nucleophilic substitution reactions of furanosyl acetals bearing acyloxy groups at C-2 can be highly 1,2-*trans* selective with strong carbon nucleophiles or oxygen nucleophiles. Experimental and computational investigations reveal that a less electron-donating *p*-nitrobenzoyloxy group at C-2 can lead to higher 1,2-*trans* selectivity by formation of a less stabilized, and therefore more reactive, *cis*-dioxolenium ion intermediate.

## Experimental Section

### General Experimental

^1^H and ^13^C NMR Spectra were acquired at room temperature using Bruker AVIII-400 (400 and 100 MHz, respectively) and AVIII-600 (600 MHz) spectrometers as indicated. The diastereomeric ratios of nucleophilic substitution reactions were obtained by ^13^C spectroscopic analysis of the representative peaks of products in the unpurified crude reaction mixture.^14^ All spectroscopic data were reported as follows: chemical shifts reported in ppm as referenced to solvent peaks (^1^H NMR: CDCl_3_ δ 7.26 ppm; ^13^C NMR: CDCl_3_ δ 77.16 ppm), multiplicity (s = singlet, br = broad, br s = broad singlet, d = doublet, ddt = doublet of doublet of triplet, t = triplet, dd = doublet of doublet, dt = doublet of triplet, m = multiplet), *J* coupling constants (Hz), and integration. High-resolution mass spectra (HRMS) were acquired using an Agilent 6224 Accurate-Mass time-of-flight spectrometer through ESI (electrospray ionization) mode. Infrared (IR) data were acquired using Nicolet 6700 FT-IR spectrometer via attenuated total reflectance (ATR). All reactions were performed under inert nitrogen atmosphere using glassware that has been flame-dried under reduced pressure. Solvents including dichloromethane, acetonitrile, toluene, and methanol were anhydrous and degassed through a solvent purification system prior to use in the reported reactions. Aqueous solutions were prepared using distilled water. Flash column chromatography was performed using the solvent system on silica gel (SiO_2_) 60 (230–400 mesh) under air flow. All reagents were commercially available unless otherwise notified.

### General Procedure for Nucleophilic Substitution Reactions of Furanosyl Thioacetals Using Oxygen Nucleophiles

To a solution of thioacetal (1.0 equiv) in solvent (0.1 M) at −45 °C was added nucleophile (6.0 equiv unless otherwise noted), followed by the addition of N-iodosuccinimide (2.0 equiv unless otherwise noted). The reaction mixture was then stirred at −45 °C for 16 h. A solution of Me_2_S:CH_2_Cl_2_:Et_3_N (1:1:1, 1 mL per mmol of thioacetal) was added at −45 °C and the reaction mixture was warmed to room temperature. The reaction mixture was then diluted with CH_2_Cl_2_ (1 × 20 mL per mmol of thioacetal) and washed with saturated aqueous Rochelle’s salt solution (1 × 20 mL per mmol of acetate). The aqueous layer was extracted with CH_2_Cl_2_ (2 × 20 mL per mmol of acetate). The combined organic layers were dried over Na_2_SO_4_, filtered, and concentrated in vacuo. The diastereomeric ratios were determined by ^13^C NMR spectroscopic analysis of the unpurified reaction mixture.^14^ The reaction mixture was purified by flash column chromatography to provide products. The relative stereochemical configurations of products were assigned by analysis of coupling constants. Details of stereochemical proofs were provided in section VII in the Supporting Information.

### General Procedure for Nucleophilic Substitution Reactions of Acetals with Carbon Nucleophiles

To a solution of acetate (1.0 equiv) in solvent (0.1 M) at −45 °C was added nucleophile (2.5 equiv unless otherwise noted), followed by the addition of Lewis acid (2.5 equiv unless otherwise noted). The reaction mixture was then stirred at −45 °C for 16 h. A solution of MeOH:CH_2_Cl_2_:Et_3_N (1:1:1, 1 mL per mmol of acetate) was added at −45 °C and the reaction mixture was warmed to room temperature. The reaction mixture was then diluted with CH_2_Cl_2_ (1 × 20 mL per mmol of acetate) and washed with saturated aqueous Rochelle’s salt solution (1 × 20 mL per mmol of acetate). The aqueous layer was extracted with CH_2_Cl_2_ (2 × 20 mL per mmol of acetate). The combined organic layers were dried over Na_2_SO_4_, filtered, and concentrated *in vacuo*. The diastereomeric ratios were determined by ^13^C{^1^H} NMR analysis of the unpurified reaction mixture.^14^ The reaction mixture was purified by flash column chromatography to provide products. The relative stereochemical configurations of products were assigned by analysis of coupling constants. Details of stereochemical proofs are provided in section VII in the Supporting Information.

#### (2R*,3S*)-2-(2,2-Difluoroethoxy)tetrahydrofuran-3-yl Benzoate (*trans*-**9a**), (2R*,3R*)-2-(2,2-Difluoroethoxy)tetrahydrofuran-3-yl Benzoate (*cis*-**9a**)

The general procedure for nucleophilic substitution reactions of thioacetals was followed using thioacetal **6a** (0.300 g, 1.00 mmol), 2,2-difluoroethanol (0.380 mL, 6.0 mmol), and *N*-iodosuccinimide (0.500 g, 2.00 mmol) in CH_2_Cl_2_ (10 mL). ^13^C{^1^H} NMR spectroscopic analysis of the unpurified reaction mixture revealed that acetal **9a** was formed as a mixture of diastereomers (*trans*-**9a**:*cis*-**9a** = 93:7).^14^ Purification by flash column chromatography (10:90 EtOAc:hexanes) afforded acetal *trans-***9a** as a yellow oil (0.117 g, 43%) and acetal *cis*-**9a** as a yellow oil as a mixture with side-product *trans*-**21a** (0.0400 g, 18%, *cis*-**9a**:*trans*-**9a** = 16:84). Note: Nucleophilic substitution reactions of thioacetal **6a** with 2,2-difluoroethanol (**20b**) were low-yielding because of the formation of orthoester side-product **15a**, which was observed in the reaction mixture but decomposed in the sequential purification step. Purification by column chromatography afforded acetal *cis*-**9a** as a mixture with an inseparable decomposition product that appeared after purification, which was identified to be dibenzoate *trans*-**21a** (*cis*-**9a**:*trans*-**21a** = 16:84). Major diastereomer *trans*-**9a**: ^1^H NMR (400 MHz, CDCl_3_) δ 8.05–8.00 (m, 2H), 7.62–7.55 (m, 1H), 7.49–7.41 (m, 2H), 5.92 (tt, *J* = 55.8, 4.4 Hz, 1H), 5.39 (dd, *J* = 5.9, 1.7 Hz, 1H), 5.21 (br s, 1H), 4.20 (q, *J* = 8.0 Hz, 1H), 4.09 (td, *J* = 8.9, 4.8 Hz, 1H), 3.91–3.80 (m, 1H), 3.79–3.70 (m, 1H), 2.55–2.43 (m, 1H), 2.15–2.05 (m, 1H); ^13^C{^1^H} NMR (100 MHz, CDCl_3_) δ 165.9 (C), 136.7 (C), 133.5 (CH), 129.8 (CH), 128.6 (CH), 114.3 (br t, ^1^*J*_C–F_ = 239.0 Hz, CH), 106.2 (CH), 78.1 (CH), 67.2 (CH_2_), 66.3 (br t, ^2^*J*_C–F_ = 28.6 Hz, CH_2_), 29.9 (CH_2_); IR (ATR) 2903, 1719, 1265, 1100, 1051, 711 cm^–1^; HRMS (TOF MS ES^+^) *m*/*z* calcd for C_13_H_18_F_2_NO_4_ (M + NH_4_)^+^ 290.1198, found 290.1178. Minor diastereomer *cis*-**9a**: ^1^H NMR (400 MHz, CDCl_3_, characteristic peaks) δ 5.84 (tt, J = 56.3, 4.4 Hz, 1H), 5.30 (d, *J* = 4.2 Hz, 1H), 5.17 (td, *J* = 8.6, 4.3 Hz, 1H), 4.16–4.08 (m, 1H), 3.97 (q, *J* = 8.0 Hz, 1H), 2.47–2.38 (m, 1H); ^13^C{^1^H} NMR (100 MHz, CDCl_3_, characteristic peaks) 166.4 (C), 136.7 (C), 134.1 (CH), 114.3 (br t, ^1^*J*_C–F_ = 242.2 Hz, CH), 105.9 (CH), 73.9 (CH), 67.2 (br t, ^2^*J*_C–F_ = 29.3 Hz, CH_2_), 64.8 (CH_2_), 27.5 (CH_2_); IR (ATR) 2980, 1719, 1261, 1061, 944, 708 cm^–1^; HRMS (TOF MS ES^+^) *m*/*z* calcd for C_13_H_14_F_2_NaO_4_ (M + Na)^+^ 295.0752, found 295.0764.

#### (2R*,3S*)-2-(2-Fluoroethoxy)tetrahydrofuran-3-yl Benzoate (*trans*-**10a**), (2R*,3R*)-2-(2-Fluoroethoxy)tetrahydrofuran-3-yl Benzoate (*cis*-**10a**), and (2R*,3aR*,6aS*)-2-Ethoxy-2-phenyltetrahydrofuro[2,3-*d*][1,3]dioxole (**16a**)

The general procedure for nucleophilic substitution reactions of thioacetals was followed using thioacetal **6a** (0.300 g, 1.00 mmol), 2-fluoroethanol (0.352 mL, 6.00 mmol), and *N*-iodosuccinimide (0.450 g, 2.00 mmol) in CH_2_Cl_2_ (10 mL). ^13^C{^1^H} NMR spectroscopic analysis of the unpurified reaction mixture revealed that acetal **10a** was formed as a mixture of diastereomers (*trans*-**10a**:*cis*-**10a** = 65:35).^14^ Purification by flash column chromatography (15:85 EtOAc:hexanes) afforded acetal *trans*-**10a** as a yellow oil (0.0400 g, 16%) and acetal *cis*-**10a** as a mixture with side-product orthoester **16a** as a yellow oil (0.0290 g, 12%, *cis*-**10a**:**16a** = 33:67). Note: Nucleophilic substitution reactions of thioacetal **6a** with 2-fluoroethanol (**20c**) were low-yielding because of the formation of orthoester side-product **16a**, which was observed in the reaction mixture but subjected to possible decomposition in the sequential purification step. Purification by column chromatography afforded acetal *cis*-**10a** as a mixture with orthoester **16a** (*cis*-**10a**:**16a** = 33:67) and an inseparable decomposition product that appeared after purification. Attempts to separate acetal *cis*-**10a** and orthoester **16a** from the unidentified decomposition impurities failed after multiple columns. Major diastereomer *trans*-**10a**: ^1^H NMR (400 MHz, CDCl_3_) δ 8.06–8.00 (m, 2H), 7.61–7.54 (m, 1H), 7.48–7.41 (m, 2H), 5.39 (dt, *J* = 5.9, 1.9 Hz, 1H), 5.21 (br s, 1H), 4.63 (t, *J* = 3.9 Hz, 1H), 4.52 (t, *J* = 3.9 Hz, 1H), 4.17 (dt, *J* = 15.9, 8.1 Hz, 1H), 4.09 (ddd, *J* = 16.0, 8.7, 5.0 Hz, 1H), 3.99–3.84 (m, 1H), 3.82–3.68 (m, 1H), 2.56–2.44 (m, 1H), 2.13–2.04 (m, 1H); ^13^C{^1^H} NMR (100 MHz, CDCl_3_) δ 166.0 (C), 133.4 (C), 129.92 (CH), 129.85 (CH), 128.6 (CH), 106.0 (CH), 82.9 (br d, ^1^*J*_C–F_ = 171.1 Hz, CH_2_), 78.3 (CH), 66.9 (CH_2_), 66.3 (br d, ^2^*J*_C–F_ = 20.0 Hz, CH2), 30.1 (CH_2_); IR (ATR) 2928, 1718, 1266, 1044, 1024, 710 cm^–1^; HRMS (TOF MS ES+) *m*/*z* calcd for C_13_H_19_FNO_4_ (M + NH_4_)^+^ 272.1393, found 272.1415. Minor diastereomer *cis*-**10a** and orthoester **16a** (*cis*-**10a**:**16a** = 33:67): ^1^H NMR (400 MHz, CDCl_3_, characteristic peaks) δ 6.09 (d, *J* = 4.2 Hz, 1H), 5.30 (d, *J* = 4.4 Hz, 0.5H), 5.17 (td, *J* = 8.5, 4.1 Hz, 0.5H), 5.03 (t, *J* = 4.5 Hz, 1H), 2.45–2.36 (m, 0.5H), 2.33–2.22 (m, 0.5H), 2.11 (dd, *J* = 14.5, 4.6 Hz, 1H), 1.94–1.83 (m, 1H); Peaks attributed to acetal *cis*-**10a**: ^13^C{^1^H} NMR (100 MHz, CDCl_3_, characteristic peaks) δ 166.5 (C), 100.4 (CH), 82.7 (br d, ^1^*J*_C–F_ = 169.5 Hz, CH_2_), 74.0 (CH), 64.5 (CH_2_), 63.4 (br d, ^2^*J*_C–F_ = 20.5 Hz, CH_2_), 26.7 (CH_2_); Peaks attributed to orthoester **16a**: ^13^C{^1^H} NMR (100 MHz, CDCl_3_, characteristic peaks) δ 144.3 (C), 106.0 (CH), 82.3 (br d, ^2^*J*_C–F_ = 170.2 Hz, CH2), 81.3 (CH), 67.0 (CH_2_), 62.8 (br d, ^2^*J*_C–F_ = 20.2 Hz, CH_2_), 33.1 (CH_2_); IR (ATR) 2956, 1717, 1263, 1103, 1024, 781 cm^–1^; HRMS (TOF MS ES^+^) *m*/*z* calcd for C_13_H_15_FNaO_4_ (M + Na)^+^ 277.0847, found 277.0849.

#### (2R*,3S*)-2-Ethoxytetrahydrofuran-3-yl Benzoate (*trans*-**11a**) and (2R*,3R*)-2-Ethoxytetrahydrofuran-3-yl Benzoate (*cis*-**11a**)

The general procedure for nucleophilic substitution reactions of thioacetals was followed using thioacetal **6a** (0.300 g, 1.00 mmol), ethanol (1.18 mL, 20.0 mmol), and *N*-iodosuccinimide (1.35 g, 6.00 mmol) in CH_2_Cl_2_ (10 mL). ^13^C{^1^H} NMR spectroscopic analysis of the unpurified reaction mixture revealed that acetal **11a** was formed as a mixture of diastereomers (*trans*-**11a**:*cis*-**11a** = 70:30).^14^ Purification by flash column chromatography (10:90 EtOAc:hexanes) afforded acetal *trans-***11a** as a yellow oil (0.0380 g, 16%) and acetal *cis*-**11a** as a yellow oil (0.0100 g, 4%) as a mixture of diastereomers (*trans*-**11a**:*cis*-**11a** = 37:63). Note: Nucleophilic substitution reactions of thioacetal **6a** with ethanol (**20d**) were low-yielding because of the formation of orthoester side-product **17a**, which was observed in the reaction mixture but decomposed in the sequential purification step. Purification by column chromatography afforded acetal *cis*-**11a** as a mixture with acetal *trans*-**11a** (*trans*-**11a**:*cis*-**11a** = 37:63) and an inseparable decomposition product that appeared after purification, which was identified to be dibenzoate *trans*-**21a**. Attempts to separate acetal *cis*-**11a** and acetal *trans*-**11a** from the decomposition product dibenzoate *trans*-**21a** failed after multiple columns. Major diastereomer *trans*-**11a**: ^1^H NMR (400 MHz, CDCl_3_) δ 8.07–7.99 (m, 2H), 7.59–7.50 (m, 1H), 7.47–7.38 (m, 2H), 5.33 (dd, *J* = 6.2, 1.5 Hz, 1H), 5.16 (br s, 1H), 4.20–4.03 (m, 2H), 3.81–3.71 (m, 1H), 3.57–3.48 (m, 1H), 2.53–2.42 (m, 1H), 2.10–2.01 (m, 1H), 1.22 (t, *J* = 7.3 Hz, 3H); ^13^C{^1^H} NMR (100 MHz, CDCl_3_) δ 166.0 (C), 133.3 (C), 130.0 (CH), 129.8 (CH), 128.5 (CH), 105.6 (CH), 78.4 (CH), 66.6 (CH_2_), 62.9 (CH_2_), 30.1 (CH_2_), 15.2 (CH_3_); IR (ATR) 2977, 1718, 1267, 1097, 1045, 709 cm^–1^; HRMS (TOF MS ES^+^) *m*/*z* calcd for C_13_H_16_NaO_4_ (M + Na)^+^ 259.0941, found 259.0949. Minor diastereomer *cis*-**11a**: ^1^H NMR (400 MHz, CDCl_3_, characteristic peaks) δ 5.25 (d, *J* = 4.6 Hz, 1H), 5.15 (td, *J* = 8.4, 4.4 Hz, 1H), 4.32–4.24 (m, 1H), 1.12 (t, *J* = 6.9 Hz, 3H); ^13^C{^1^H} NMR (100 MHz, CDCl_3_, characteristic peaks) δ 166.5 (C), 100.1 (CH), 74.1 (CH), 64.3 (CH_2_), 63.9 (CH_2_), 28.0 (CH_2_), 15.4 (CH_3_); IR (ATR) 2899, 1721, 1271, 1104, 1027, 712 cm^–1^; HRMS (TOF MS ES^+^) *m*/*z* calcd for C_13_H_18_NO_3_ (M + NH_4_ – H_2_O)^+^ 236.1281, found 236.1292.

#### (2R*,3S*)-2-Isopropoxytetrahydrofuran-3-yl Benzoate (*trans*-**12a**), (2R*,3R*)-2-Isopropoxytetrahydrofuran-3-yl Benzoate (*cis*-**12a**), and (2R*,3aR*,6aS*)-2-Isopropoxy-2-phenyltetrahydrofuro[2,3-*d*][1,3]dioxole (**18a**)

The general procedure for nucleophilic substitution reactions of thioacetals was followed using thioacetal **6a** (0.300 g, 1.00 mmol), isopropyl alcohol (0.763 mL, 10.0 mmol), and *N*-iodosuccinimide (0.500 g, 2.00 mmol) in CH_2_Cl_2_ (10 mL). ^13^C{^1^H} NMR spectroscopic analysis of the unpurified reaction mixture revealed that acetal **12a** was formed as a mixture of diastereomers (*trans*-**12a**:*cis*-**12a** = 76:24).^14^ Purification by flash column chromatography (10:90 EtOAc:hexanes) afforded acetal *trans-***12a** as a yellow oil (0.111 g, 44%), acetal *cis*-**12a** as a yellow oil (0.0141 g, 6%) as an inseparable mixture with side-product isopropyl benzenesulfinate (*cis*-**12a**:isopropyl benzenesulfinate = 67:33), and orthoester **18a** as a yellow oil (0.113 g, 45%). Major diastereomer *trans*-**12a**: ^1^H NMR (400 MHz, CDCl_3_) δ 8.06–8.00 (m, 2H), 7.58–7.51 (m, 1H), 7.47–7.41 (m, 2H), 5.29 (dd, *J* = 5.9, 1.6 Hz, 1H), 5.25 (br s, 1H), 4.17–4.03 (m, 2H), 3.98–3.89 (m, 1H), 2.53–2.42 (m, 1H), 2.09–2.00 (m, 1H), 1.20 (d, *J* = 6.1 Hz, 3H), 1.19 (d, *J* = 6.6 Hz, 3H); ^13^C{^1^H} NMR (100 MHz, CDCl_3_) δ 166.1 (C), 134.7 (C), 133.3 (CH), 129.8 (CH), 128.5 (CH), 104.1 (CH), 78.9 (CH), 69.2 (CH), 66.4 (CH_2_), 30.2 (CH_2_), 23.6 (CH_3_), 21.9 (CH_3_); IR (ATR) 2975, 1788, 1211, 1034, 703, 614 cm^–1^; HRMS (TOF MS ES^+^) *m*/*z* calcd for C_14_H_17_O_3_ (M + H – H_2_O)^+^ 233.1172, found 233.1171. Minor diastereomer *cis*-**12a**: ^1^H NMR (400 MHz, CDCl_3_) δ 8.10–8.02 (m, 2H), 7.77–7.67 (m, 1H), 7.49–7.40 (m, 2H), 5.35 (d, *J* = 4.4 Hz, 1H), 5.12 (td, *J* = 8.5, 4.2 Hz, 1H), 4.16–4.05 (m, 1H), 3.95–3.79 (m, 2H), 2.42–2.32 (m, 1H), 2.30–2.20 (m, 1H), 1.19 (d, *J* = 6.7 Hz, 3H), 1.03 (d, *J* = 6.0 Hz, 3H); ^13^C{^1^H} NMR (100 MHz, CDCl_3_) 166.5 (C), 136.7 (C), 133.2 (CH), 129.9 (CH), 128.5 (CH), 99.3 (CH), 74.1 (CH), 71.0 (CH), 64.0 (CH_2_), 27.9 (CH_2_), 23.6 (CH_3_), 22.2 (CH_3_); IR (ATR) 2974, 1720, 1271, 1117, 1029, 712 cm^–1^; HRMS (TOF MS ES^+^) *m*/*z* calcd for C_14_H_20_NO_3_ (M + NH_4_ – H_2_O)^+^ 250.1438, found 250.1437. Orthoester **18a**: ^1^H NMR (400 MHz, CDCl_3_) δ 7.67–7.63 (m, 2H), 7.38–7.34 (m, 3H), 6.61 (d, *J* = 4.0 Hz, 1H), 5.00 (t, *J* = 4.4 Hz, 1H), 3.88 (t, *J* = 7.8 Hz, 1H), 3.79–3.69 (m, 1H), 3.60 (ddd, *J* = 13.3, 8.6, 4.6 Hz, 1H), 2.08 (dd, *J* = 13.7, 4.9 Hz, 1H), 1.93–1.82 (m, 1H), 1.15 (d, *J* = 6.2 Hz, 3H), 1.17 (d, *J* = 6.2 Hz, 3H); ^13^C{^1^H} NMR (100 MHz, CDCl_3_) δ 138.1 (C), 129.2 (CH), 128.2 (CH), 126.3 (CH), 123.1 (C), 105.9 (CH), 81.0 (CH), 67.0 (CH_2_), 66.6 (CH), 33.3 (CH_2_), 23.7 (CH_3_), 23.6 (CH_3_).

#### (2R*,3S*)-2-((1,1,1,3,3,3-Hexafluoropropan-2-yl)oxy)tetrahydrofuran-3-yl Benzoate (*trans*-**13a**)

The general procedure for nucleophilic substitution reactions of thioacetals was followed using thioacetal **6a** (0.0600 g, 0.200 mmol), 1,1,3,3-hexafluoroisopropanol (0.126 mL, 1.20 mmol), and *N*-iodosuccinimide (0.0900 g, 0.400 mmol) in CH_2_Cl_2_ (2 mL). ^13^C{^1^H} NMR spectroscopic analysis of the unpurified reaction mixture revealed that acetal *trans-***13a** was formed as a single diastereomer.^14^ Purification by flash column chromatography (10:90 EtOAc:hexanes) afforded acetal *trans-***13a** as a yellow oil (0.0150 g, 21%). Note: Purification by column chromatography afforded acetal *trans*-**13a** as a mixture with inseparable unidentified decomposition impurities that appeared after purification. Attempts to separate acetal *trans*-**13a** from the decomposition impurities failed after multiple columns. Acetal *trans*-**13a**: ^1^H NMR (400 MHz, CDCl_3_) δ 8.07–7.99 (m, 2H), 7.62–7.56 (m, 1H), 7.48–7.42 (m, 2H), 5.51 (dd, *J* = 5.8, 1.1 Hz, 1H), 5.43 (br s, 1H), 4.62–4.54 (m, 1H), 4.27 (dt, *J* = 16.3, 8.0 Hz, 1H), 4.16 (td, *J* = 9.1, 4.3 Hz, 1H), 2.56–2.45 (m, 1H), 2.22–2.12 (m, 1H); ^13^C{^1^H} NMR (100 MHz, CDCl_3_, characteristic peaks) δ 165.6 (C), 136.6 (CH), 129.9 (CH), 128.6 (CH), 105.9 (CH), 77.7 (CH), 67.0 (br m, ^2^*J*_C–F_ = 28.6 Hz, CH), 68.5 (CH_2_), 29.3 (CH_2_); IR (ATR) 2963, 1724, 1191, 1102, 934, 710 cm^–1^; HRMS (TOF MS ES^+^) *m*/*z* calcd for C_14_H_12_F_6_NaO_4_ (M + Na)^+^ 381.0532, found 381.0497.

#### (2R*,3S*)-2-(2,2-Difluoroethoxy)tetrahydrofuran-3-yl 4-Nitrobenzoate (*trans*-**9b**) and (2R*,3aR*,6aS*)-2-(2,2-Difluoroethoxy)-2-(4-nitrophenyl)tetrahydrofuro[2,3-*d*][1,3]dioxole (**15b**)

The general procedure for nucleophilic substitution reactions of thioacetals was followed using thioacetal **6b** (0.0500 g, 0.149 mmol), 2,2-difluoroethanol (0.0551 mL, 0.870 mmol), and *N*-iodosuccinimide (0.0650 g, 0.290 mmol) in CH_2_Cl_2_ (1.5 mL). ^13^C{^1^H} NMR spectroscopic analysis of the unpurified reaction mixture revealed that acetal **9b** was formed as a single diastereomer.^14^ Purification by flash column chromatography (15:85 EtOAc:hexanes) afforded acetal *trans-***9b** as a yellow oil (0.0140 g, 30%) and orthoester **15b** as a yellow oil (0.0120 g, 26%). Acetal *trans-***9b**: ^1^H NMR (400 MHz, CDCl_3_) δ 8.33–8.28 (m, 2H), 8.22–8.17 (m, 2H), 5.92 (tt, *J* = 55.2, 4.3 Hz, 1H), 5.42 (dd, *J* = 6.5, 1.6 Hz, 1H), 5.23 (br s, 1H), 4.21 (q, *J* = 7.8 Hz, 1H), 4.10 (td, *J* = 8.6, 4.9 Hz, 1H), 3.94–3.66 (m, 2H), 2.60–2.47 (m, 1H), 2.17–2.06 (m, 1H); ^13^C{^1^H} NMR (100 MHz, CDCl_3_) δ 164.1 (C), 150.9 (C), 135.1 (C), 131.0 (CH), 123.8 (CH), 114.3 (br t, ^1^*J*_C–F_ = 240.7 Hz, CH), 105.9 (CH), 79.1 (CH), 67.1 (CH_2_), 66.3 (br t, ^2^*J*_C–F_ = 28.3 Hz, CH_2_), 29.8 (CH_2_); IR (ATR) 2996, 1726, 1525, 1269, 1054, 719 cm^–1^; HRMS (TOF MS ES^+^) *m*/*z* calcd for C_13_H_14_F_2_NO_6_ (M + H)^+^ 318.0784, found 318.0801. Orthoester **15b**: ^1^H NMR (400 MHz, CDCl_3_) δ 8.27–8.22 (m, 2H), 7.84–7.79 (m, 2H), 6.13 (d, *J* = 3.9 Hz, 1H), 3.87 (tt, *J* = 55.8, 4.1 Hz, 1H), 5.07 (t, *J* = 4.3 Hz, 1H), 3.93 (t, *J* = 8.6 Hz, 1H), 3.68–3.57 (m, 2H), 3.59–3.45 (m, 1H), 2.14–2.08 (m, 1H), 2.00–1.88 (m, 1H); ^13^C{^1^H} NMR (100 MHz, CDCl_3_) δ 148.8 (C), 143.1 (C), 127.4 (CH), 123.8 (CH), 121.6 (C), 114.0 (br t, ^1^*J*_C–F_ = 239.4 Hz, CH), 106.4 (CH), 82.1 (CH), 67.3 (CH_2_), 62.7 (br t, ^2^*J*_C–F_ = 29.9 Hz, CH_2_), 33.1 (CH_2_); IR (ATR) 2980, 1516, 1269, 1113, 1025, 709 cm^–1^; HRMS (TOF MS ES^+^) *m*/*z* calcd for C_13_H_11_F_2_NNaO_5_ (M + Na – H_2_O)^+^ 322.0497, found 322.0513.

#### (2R*,3S*)-2-(2-Fluoroethoxy)tetrahydrofuran-3-yl 4-Nitrobenzoate (*trans*-**10b**), (2R*,3R*)-2-(2-Fluoroethoxy)tetrahydrofuran-3-yl 4-Nitrobenzoate (*cis*-**10b**), and (2R*,3aR*,6aS*)-2-(2-Fluoroethoxy)-2-(4-nitrophenyl)tetrahydrofuro[2,3-*d*][1,3]dioxole (**16b**)

The general procedure for nucleophilic substitution reactions of thioacetals was followed using thioacetal **6b** (0.100 g, 0.290 mmol), 2-fluoroethanol (0.102 mL, 1.74 mmol), and *N*-iodosuccinimide (0.130 g, 0.290 mmol) in CH_2_Cl_2_ (3 mL). ^13^C{^1^H} NMR spectroscopic analysis of the unpurified reaction mixture revealed that acetal **10b** was formed as a mixture of diastereomers (*trans*-**10b**:*cis*-**10b** = 75:25).^14^ Purification by flash column chromatography (10:90 EtOAc:hexanes) afforded acetal *trans-***10b** as a yellow oil (0.0210 g, 25%), acetal *cis*-**10b** as a yellow oil (0.0090 g, 10%), and orthoester **16b** as a yellow oil (0.0190 g, 22%). Major diastereomer *trans-***10b**: ^1^H NMR (400 MHz, CDCl_3_) δ 8.30–8.26 (m, 2H), 8.21–8.17 (m, 2H), 5.41 (dt, *J* = 3.9, 1.7 Hz, 1H), 5.22 (br s, 1H), 4.63 (td, *J* = 3.6, 1.5 Hz, 1H), 4.51 (td, *J* = 3.8, 1.4 Hz, 1H), 4.21–4.14 (m, 1H), 4.12–4.05 (m, 1H), 3.98–3.84 (m, 2H), 2.59–2.48 (m, 1H), 2.13–2.02 (m, 1H); ^13^C{^1^H} NMR (100 MHz, CDCl_3_) δ 164.1 (C), 150.9 (C), 135.2 (C), 131.0 (CH), 123.7 (CH), 105.6 (CH), 82.9 (br d, ^1^*J*_C–F_ = 170.3 Hz, CH_2_), 79.3 (CH), 66.8 (CH_2_), 66.3 (br d, ^2^*J*_C–F_ = 19.8 Hz, CH_2_), 30.0 (CH_2_); IR (ATR) 2958, 1727, 1529, 1266, 1104, 733 cm^–1^; HRMS (TOF MS ES^+^) *m*/*z* calcd for C_13_H_14_FNNaO_6_ (M + Na)^+^ 322.0697, found 322.0687. Minor diastereomer *cis-***10b**: ^1^H NMR (400 MHz, CDCl_3_) δ 8.32–8.26 (m, 2H), 8.25–8.21 (m, 2H), 5.31 (d, *J* = 4.3 Hz, 1H), 5.21 (td, *J* = 8.4, 4.3 Hz, 1H), 4.56–4.48 (m, 1H), 4.44–4.39 (m, 1H), 4.17–4.08 (m, 1H), 4.04–3.93 (m, 1H), 3.91–3.85 (m, 1H), 3.79–3.65 (m, 1H), 2.49–2.39 (m, 1H), 2.35–2.23 (m, 1H); ^13^C{^1^H} NMR (100 MHz, CDCl_3_) δ 164.7 (C), 150.8 (C), 135.4 (C), 131.0 (CH), 123.7 (CH), 100.3 (CH), 82.8 (br d, ^1^*J*_C–F_ = 169.2 Hz, CH_2_), 74.8 (CH), 67.2 (br d, ^2^*J*_C–F_ = 20.3 Hz, CH_2_), 64.6 (CH_2_), 27.8 (CH_2_); IR (ATR) 2959, 1726, 1527, 1268, 1028, 733 cm^–1^; HRMS (TOF MS ES^+^) *m*/*z* calcd for C_13_H_14_FNNaO_6_ (M + Na)^+^ 323.073, found 323.0732. Orthoester **16b**: δ 8.26–8.21 (m, 2H), 7.86–7.80 (m, 2H), 6.12 (d, *J* = 4.0 Hz, 1H), 5.06 (t, *J* = 4.6 Hz, 1H), 4.60 (t, *J* = 4.4 Hz, 1H), 4.48 (t, *J* = 4.6 Hz, 1H), 3.93 (t, *J* = 8.3 Hz, 1H), 3.75–3.67 (m, 1H), 3.65–3.60 (m, 1H), 3.54 (ddd, *J* = 16.5, 8.8, 5.4 Hz, 1H), 2.11 (dd, *J* = 14.2, 5.0 Hz, 1H), 1.98–1.87 (m, 1H); ^13^C{^1^H} NMR (100 MHz, CDCl_3_) δ 148.6 (C), 143.7 (C), 127.4 (CH), 123.7 (CH), 121.5 (C), 106.3 (CH), 82.1 (br d, ^1^*J*_C–F_ = 168.9 Hz, CH_2_), 81.9 (CH), 67.2 (CH_2_), 62.5 (br d, ^2^*J*_C–F_ = 20.9 Hz, CH_2_), 33.2 (CH_2_); IR (ATR) 2960, 1525, 1268, 1048, 974, 733 cm^–1^; HRMS (TOF MS ES^+^) *m*/*z* calcd for C_13_H_14_FNNaO_6_ (M + Na)^+^ 322.0697, found 322.0709.

#### (2R*,3S*)-2-Ethoxytetrahydrofuran-3-yl 4-Nitrobenzoate (*trans*-**11b**), (2R*,3R*)-2-Ethoxytetrahydrofuran-3-yl 4-Nitrobenzoate (*cis*-**11b**), and (2R*,3aR*,6aS*)-2-Ethoxy-2-(4-nitrophenyl)tetrahydrofuro[2,3-*d*][1,3]dioxole (**17b**)

The general procedure for nucleophilic substitution reactions of thioacetals was followed using thioacetal **6b** (0.0550 g, 0.159 mmol), ethanol (0.100 mL, 1.59 mmol), and *N*-iodosuccinimide (0.215 g, 0.955 mmol) in CH_2_Cl_2_ (1.6 mL). ^13^C{^1^H} NMR spectroscopic analysis of the unpurified reaction mixture revealed that acetal **11b** was formed as a mixture of diastereomers (*trans*-**11b**:*cis*-**11b** = 80:20).^14^ Purification by flash column chromatography (10:90 EtOAc:hexanes) afforded acetal **11b** as a yellow oil (0.0210 g, 47%) as a mixture of diastereomers (*trans*-**11b**:*cis*-**11b** = 40:60) and orthoester **17b** as a yellow oil (0.0120 g, 27%). Major diastereomer *trans*-**11b** and minor diastereomer *cis*-**11b** (*trans*-**11b**:*cis*-**11b** = 40:60): ^1^H NMR (400 MHz, CDCl_3_) δ 8.33–8.26 (m, 3.6H), 8.26–8.17 (m, 3.6H), 5.36 (dd, *J* = 6.2, 1.5 Hz, 0.8H), 5.27 (d, *J* = 4.3 Hz, 1H), 5.19 (td, *J* = 8.1, 4.4 Hz, 1H), 5.17 (br s, 0.8H), 4.19–4.04 (m, 2.8H), 3.94 (dt, *J* = 10.4, 6.5 Hz, 1H), 3.81–3.70 (m, 1.8H), 3.59–3.43 (m, 1.8H), 2.57–2.50 (m, 0.8H), 2.45–2.35 (m, 1H), 2.32–2.20 (m, 1H), 2.16–2.01 (m, 0.8H), 1.22 (t, *J* = 7.2 Hz, 2.4H), 1.22 (t, *J* = 7.2 Hz, 3H); Peaks attributed to isomer *trans-***11b**: ^13^C{^1^H} NMR (100 MHz, CDCl_3_) δ 164.2 (C), 150.81 (C), 135.3 (C), 130.9 (CH), 123.72 (CH), 105.4 (CH), 79.5 (CH), 66.5 (CH_2_), 63.0 (CH_2_), 30.1 (CH_2_), 15.2 (CH_3_); Peaks attributed to isomer *cis-***11b**: ^13^C{^1^H} NMR (100 MHz, CDCl_3_) δ 164.6 (C), 150.80 (C), 135.5 (C), 131.0 (CH), 123.70 (CH), 100.0 (CH), 74.9 (CH), 64.3 (CH_2_), 63.9 (CH_2_), 28.1 (CH_2_), 15.3 (CH_3_); IR (ATR) 2977, 1725, 1526, 1269, 1100, 592 cm^–1^; HRMS (TOF MS ES^+^) *m*/*z* calcd for C_13_H_15_NNaO_6_ (M + Na)^+^ 304.0792, found 304.0779. Orthoester **17b**: ^1^H NMR (400 MHz, CDCl_3_) δ 8.25–8.19 (m, 2H), 7.85–7.79 (m, 2H), 6.11 (d, *J* = 3.7 Hz, 1H), 5.03 (t, *J* = 4.9 Hz, 1H), 3.91 (t, *J* = 8.5 Hz, 1H), 3.55–3.44 (m, 3H), 2.15–2.04 (m, 1H), 1.96–1.87 (m, 1H), 1.21 (t, *J* = 7.2 Hz, 3H); ^13^C{^1^H} NMR (100 MHz, CDCl_3_) δ 148.5 (C), 144.7 (C), 127.3 (CH), 123.6 (CH), 121.8 (C), 106.2 (CH), 81.9 (CH), 67.1 (CH_2_), 58.8 (CH_2_), 33.3 (CH_2_), 15.1 (CH_3_); IR (ATR) 2980, 2361, 1727, 1275, 1106, 976 cm^–1^; HRMS (TOF MS ES^+^) *m*/*z* calcd for C_13_H_15_NNaO_6_ (M + Na)^+^ 304.0792, found 304.0799.

#### (2R*,3S*)-2-Isopropoxytetrahydrofuran-3-yl 4-Nitrobenzoate (*trans*-**12b**), (2R*,3R*)-2-Isopropoxytetrahydrofuran-3-yl 4-Nitrobenzoate (*cis*-**12b**), and (2R*,3aR*,6aS*)-2-Isopropoxy-2-(4-nitrophenyl)tetrahydrofuro[2,3-*d*][1,3]dioxole (**18b**)

The general procedure for nucleophilic substitution reactions of thioacetals was followed using thioacetal **6b** (0.345 g, 1.00 mmol), isopropyl alcohol (0.763 mL, 6.00 mmol), and *N*-iodosuccinimide (0.450 g, 2.00 mmol) in CH_2_Cl_2_ (10 mL). ^13^C{^1^H} NMR spectroscopic analysis of the unpurified reaction mixture revealed that acetal **12b** was formed as a mixture of diastereomers (*trans*-**12b**:*cis*-**12b** = 80:20).^14^ Purification by flash column chromatography (10:90 EtOAc:hexanes) afforded acetal *trans-***12b** as a yellow oil (0.0950 g, 32%), acetal *cis*-**12b** as a yellow oil (0.0200 g, 8%), and orthoester **18b** as a yellow oil (0.144 g, 49%). Major diastereomer *trans-***12b**: ^1^H NMR (400 MHz, CDCl_3_) δ 8.29 (d, *J* = 8.8 Hz, 2H), 8.20 (d, *J* = 8.5 Hz, 2H), 5.32 (dd, *J* = 6.2, 1.2 Hz, 1H), 5.26 (br s, 1H), 4.18–4.04 (m, 2H), 3.99–3.88 (m, 1H), 2.59–2.44 (m, 1H), 2.12–2.60 (m, 1H), 1.20 (t, *J* = 6.4 Hz, 6H); ^13^C{^1^H} NMR (100 MHz, CDCl_3_) δ 164.2 (C), 150.8 (C), 135.4 (C), 131.0 (CH), 123.7 (CH), 103.7 (CH), 79.8 (CH), 69.2 (CH_2_), 66.2 (CH_2_), 30.1 (CH_2_), 23.5 (CH_3_), 21.8 (CH_3_); IR (ATR) 3285, 1724, 1528, 1269, 1034, 719 cm^–1^; HRMS (TOF MS ES^+^) *m*/*z* calcd for C_14_H_17_NNaO_6_ (M + Na)^+^ 318.0948, found 318.0947. Minor diastereomer *cis*-**12b**: ^1^H NMR (400 MHz, CDCl_3_) δ 8.33–8.27 (m, 2H), 8.25–8.19 (m, 2H), 5.36 (d, *J* = 4.5 Hz, 1H), 5.16 (ddd, *J* = 11.9, 7.5, 4.2 Hz, 1H), 4.15–4.07 (m, 1H), 3.95–3.89 (m, 1H), 3.87–3.81 (m, 1H), 2.44–2.33 (m, 1H), 2.32–2.20 (m, 1H), 1.18 (d, *J* = 6.3 Hz, 3H), 1.00 (d, *J* = 6.1 Hz, 3H); ^13^C{^1^H} NMR (100 MHz, CDCl_3_) δ 164.7 (C), 150.7 (C), 135.7 (C), 130.9 (CH), 123.7 (CH), 99.1 (CH), 74.9 (CH), 71.1 (CH_2_), 64.0 (CH_2_), 28.1 (CH_2_), 23.7 (CH_3_), 22.2 (CH_3_); IR (ATR) 2980, 1727, 1527, 1273, 1031, 720 cm^–1^; HRMS (TOF MS ES^+^) *m*/*z* calcd for C_14_H_15_KNO_5_ (M + K – H_2_O)^+^ 316.0582, found 316.0577. Orthoester **18b**: ^1^H NMR (400 MHz, CDCl_3_) δ 8.24–8.18 (m, 2H), 7.86–7.77 (m, 2H), 6.09 (d, *J* = 4.1 Hz, 1H), 5.02 (t, *J* = 4.5 Hz, 1H), 3.89 (t, *J* = 8.5 Hz, 1H), 3.84–3.76 (m, 1H), 3.50–3.40 (m, 1H), 2.07 (dd, *J* = 13.5, 4.4 Hz, 1H), 1.96–1.84 (m, 1H), 1.18 (d, *J* = 6.3 Hz, 3H), 1.14 (d, *J* = 6.3 Hz, 3H); ^13^C{^1^H} NMR (100 MHz, CDCl_3_) δ 148.5 (C), 144.7 (C), 127.3 (CH), 123.6 (CH), 121.8 (C), 106.2 (CH), 81.9 (CH), 67.2 (CH_2_), 58.8 (CH_2_), 33.3 (CH_2_), 15.1 (CH_3_); IR (ATR) 2977, 1523, 1271, 1119, 1050, 735 cm^–1^; HRMS (TOF MS ES^+^) *m*/*z* calcd for C_14_H_15_KNO_5_ (M + K – H_2_O)^+^ 316.0582, found 316.0577.

#### (2R*,3S*)-2-((1,1,1,3,3,3-Hexafluoropropan-2-yl)oxy)tetrahydrofuran-3-yl 4-Nitrobenzoate (*trans*-**13b**)

The general procedure for nucleophilic substitution reactions of thioacetals was followed using thioacetal **6b** (0.0700 g, 0.203 mmol), 1,1,3,3-hexafluoroisopopanol (0.128 mL, 1.22 mmol), and *N*-iodosuccinimide (0.0911 g, 0.405 mmol) in CH_2_Cl_2_ (2 mL). ^13^C{^1^H} NMR spectroscopic analysis of the unpurified reaction mixture revealed that acetal *trans*-**13b** was formed as a single diastereomer.^14^ Purification by flash column chromatography (5:95 EtOAc:hexanes) afforded acetal *trans-***13b** as a yellow oil (0.0183 g, 22%): ^1^H NMR (400 MHz, CDCl_3_) δ 8.34–8.27 (m, 2H), 8.23–8.18 (m, 2H), 5.54 (dd, *J* = 5.8, 1.2 Hz, 1H), 5.45 (br s, 1H), 4.61–4.53 (m, 1H), 4.28 (dt, *J* = 8.2, 8.0 Hz, 1H), 4.18 (td, *J* = 9.6, 4.2 Hz, 1H), 2.62–2.50 (m, 1H), 2.25–2.15 (m, 1H); ^13^C{^1^H} NMR (100 MHz, CDCl_3_, characteristic peaks) δ 163.7 (C), 150.9 (C), 134.6 (C), 130.9 (CH), 123.7 (CH), 105.5 (CH), 78.5 (CH), 69.8 (br q, ^2^*J*_C–F_ = 33.8 Hz, CH), 68.3 (CH_2_), 29.1 (CH_2_); IR (ATR) 2917, 1730, 1529, 1264, 1100, 717 cm^–1^; HRMS (TOF MS ES^+^) *m*/*z* calcd for C_14_H_13_F_6_N_2_O_5_ (M + NH_4_ – H_2_O)^+^ 403.0723, found 403.0733.

#### (2R*,3S*)-2-Allyltetrahydrofuran-3-yl Benzoate (*trans*-**22a**) and (2R*,3R*)-2-Allyltetrahydrofuran-3-yl Benzoate (*cis*-**22a**)

To a solution of dibenzoate **21a** (0.0310 g, 0.100 mmol) in CH_2_Cl_2_ (1 mL) at −45 °C was added allylchlorodimethylsilane (0.110 mL, 1.00 mmol) followed by the addition of BF_3_•OEt_2_ (0.0620 mL, 0.500 mmol). After 1 h, the reaction mixture was warmed to 25 °C. After 16 h, a solution of saturated aqueous NaHCO_3_ (5 mL) was added and the layers were separated. The aqueous layer was extracted with CH_2_Cl_2_ (2 × 10 mL). The combined organic layers were dried over Na_2_SO_4_, filtered, and concentrated *in vacuo*. ^13^C{^1^H} NMR spectroscopic analysis of the unpurified reaction mixture revealed that benzoate **5** was formed as a mixture of diastereomers (*trans-***22a**:*cis*-**22a** = 70:30).^14^ The spectral data obtained are consistent with those of products from nucleophilic substitution reactions of dibenzoate **21a** and allyltrimethylsilane in the presence of SnCl_4_.

#### (2R*,3S*)-2-Allyltetrahydrofuran-3-yl Benzoate (*trans*-**22a**) and (2R*,3R*)-2-Allyltetrahydrofuran-3-yl Benzoate (*cis*-**22a**)

The general procedure for nucleophilic substitution reactions of acetates was followed using dibenzoate **21a** (0.100 g, 0.320 mmol), allyltrimethylsilane (0.254 mL, 1.60 mmol), and SnCl_4_ (0.800 mL, 0.80 mmol, 1.0 M in CH_2_Cl_2_) in CH_2_Cl_2_ (2.4 mL). ^13^C{^1^H} NMR spectroscopic analysis of the unpurified reaction mixture revealed that benzoate **22a** was formed as a mixture of diastereomers (*trans*-**22a**:*cis*-**22a** = 55:45).^14^ Purification by flash column chromatography (20:80 EtOAc:hexanes) afforded benzoate *trans-***22a** as a yellow oil (0.0311 g, 42%) and *cis*-**22a** as a yellow oil (0.0231 g, 31%). Note: Purification by column chromatography afforded acetal *cis*-**22a** as a mixture with an inseparable decomposition product that appeared after purification, which was identified to be dibenzoate *trans*-**21a** (*cis*-**22a**:*trans*-**21a** = 67:33). Major diastereomer *trans-***22a**: ^1^H NMR (400 MHz, CDCl_3_) δ 8.05–8.01 (m, 2H), 7.60–7.54 (m, 1H), 7.48–7.41 (m, 2H), 5.92–5.81 (m, 1H), 5.23 (dt, *J* = 6.5, 2.2 Hz, 1H), 5.17 (dq, *J* = 17.1, 1.6 Hz, 1H), 5.14–5.09 (m, 1H), 4.12–4.04 (m, 2H), 4.00–3.92 (m, 1H), 2.50–2.35 (m, 2H), 2.33–2.23 (m, 1H), 2.13–2.05 (m, 1H); ^13^C{^1^H} NMR (100 MHz, CDCl_3_) δ 166.3 (C), 134.0 (CH), 133.3 (CH), 130.2 (C), 129.8 (CH), 128.5 (CH), 117.8 (CH_2_), 83.5 (CH), 78.5 (CH), 67.2 (CH_2_), 38.1 (CH_2_), 32.7 (CH_2_);IR (ATR) 2981, 1716, 1269, 1069, 915, 710 cm^–1^; HRMS (TOF MS ES^+^) *m*/*z* calcd for C_14_H_16_NaO_3_ (M + Na)^+^ 255.0992, found 255.0997. Minor diastereomer *cis-***22a**: ^1^H NMR (400 MHz, CDCl_3_, characteristic peaks) δ 8.07–8.03 (m, 2H), 7.60–7.55 (m, 1H), 7.48–7.44 (m, 2H), 5.91–5.78 (m, 1H), 5.55 (ddd, *J* = 7.8, 3.9, 1.8 Hz, 1H), 5.14–5.02 (m, 2H), 4.12 (q, *J* = 7.8 Hz, 1H), 3.93 (ddd, *J* = 9.4, 6.7, 3.8 Hz, 1H), 3.87 (td, *J* = 8.7, 5.3 Hz, 1H), 2.53–2.37 (m, 3H), 2.17–2.09 (m, 1H); ^13^C{^1^H} NMR (100 MHz, CDCl_3_, characteristic peaks) δ 166.1 (C), 134.5 (CH), 133.3 (CH), 129.81 (C), 129.78 (CH), 128.6 (CH), 117.4 (CH_2_), 81.3 (CH), 75.1 (CH), 66.3 (CH_2_), 34.0 (CH_2_), 33.8 (CH_2_); IR (ATR) 2920, 1728, 1238, 1056, 801, 695 cm^–1^; HRMS (TOF MS ES^+^) *m*/*z* calcd for C_14_H_14_NaO_2_ (M + Na – H_2_O)^+^ 237.0886, found 237.0891.

#### (2R*,3S*)-2-Allyltetrahydrofuran-3-yl Benzoate (*trans*-**22a**) and (2R*,3R*)-2-Allyltetrahydrofuran-3-yl Benzoate (*cis*-**22a**)

To a solution of dibenzoate **21a** (0.150 g, 0.480 mmol) in CH_2_Cl_2_ (5 mL) at −45 °C was added allyltributylstannane (0.595 mL, 1.92 mmol) followed by the addition of BF_3_•OEt_2_ (0.118 mL, 0.961 mmol). After 1 h, the reaction mixture was warmed to 25 °C. After 16 h, a solution of saturated aqueous NaHCO_3_ (5 mL) was added and the layers were separated. The aqueous layer was extracted with CH_2_Cl_2_ (2 × 10 mL). The combined organic layers were dried over Na_2_SO_4_, filtered, and concentrated *in vacuo*. ^13^C{^1^H} NMR spectroscopic analysis of the unpurified reaction mixture revealed that benzoate **22a** was formed as a mixture of diastereomers (*trans*-**22a**:*cis*-**22a** = 87:13).^14^ Purification by flash column chromatography (20:80 EtOAc:hexanes) afforded benzoate **22a** as a colorless oil (0.0630 g, 57%) as a mixture of diastereomers (*trans*-**22a**:*cis*-**22a** = 87:13). The spectral data obtained are consistent with those of products from nucleophilic substitution reactions of dibenzoate **21a** and allyltrimethylsilane in the presence of SnCl_4_.

#### (2R*,3S*)-2-Allyltetrahydrofuran-3-yl Benzoate (*trans*-**22a**) and (2R*,3R*)-2-Allyltetrahydrofuran-3-yl Benzoate (*cis*-**22a**) Formed Using Me_3_SiOTf as the Lewis Acid

The general procedure for nucleophilic substitution reactions of acetates was followed using dibenzoate **21a** (0.100 g, 0.320 mmol), allyltributylstannane (0.397 mL, 1.28 mmol), and Me_3_SiOTf (0.397 mL, 1.28 mmol) in CH_2_Cl_2_ (3 mL). ^13^C{^1^H} NMR spectroscopic analysis of the unpurified reaction mixture revealed that benzoate *trans*-**22a** was formed as a single diastereomer (*trans*-**22a**:*cis*-**22a** ≥ 97:3).^14^ The spectral data obtained are consistent with those of products from nucleophilic substitution reactions of dibenzoate **21a** and allyltrimethylsilane in the presence of SnCl_4_.

#### (2R*,3S*)-2-Allyltetrahydrofuran-3-yl 4-Nitrobenzoate (*trans*-**22b**) and (2R*,3R*)-2-Allyltetrahydrofuran-3-yl 4-Nitrobenzoate (*cis*-**22b**)

The general procedure for nucleophilic substitution reactions of acetates was followed using *p*-nitrobenzoate **21b** (0.0300 g, 0.0746 mmol), allylchlorodimethylsilane (0.0413 mL, 0.372 mmol), and SnCl_4_ (0.186 mL, 0.19 mmol, 1.0 M in CH_2_Cl_2_) in CH_2_Cl_2_ (0.5 mL). ^13^C{^1^H} NMR spectroscopic analysis of the unpurified reaction mixture revealed that *p*-nitrobenzoate **22b** was formed as a mixture of diastereomers (*trans-***22b**:*cis-***22b** = 61:39).^14^ The spectral data obtained are consistent with those of products from nucleophilic substitution reactions of *p*-nitrobenzoate **21b** and allyltrimethylsilane in the presence of SnCl_4_.

#### (2R*,3S*)-2-Allyltetrahydrofuran-3-yl 4-Nitrobenzoate (*trans*-**22b**) and (2R*,3R*)-2-Allyltetrahydrofuran-3-yl 4-Nitrobenzoate (*cis*-**22b**) Formed Using BF_3_•OEt_2_ as the Lewis Acid

To a solution of *p*-nitrobenzoate **21b** (0.0300 g, 0.0961 mmol) in CH_2_Cl_2_ (0.96 mL) at −45 °C was added allylchlorodimethylsilane (0.0540 mL, 0.481 mmol) followed by the addition of BF_3_•OEt_2_ (0.0593 mL, 0.481 mmol). After 1 h, the reaction mixture was warmed to 25 °C. After 16 h, a solution of saturated aqueous NaHCO_3_ (5 mL) was added and the layers were separated. The aqueous layer was extracted with CH_2_Cl_2_ (2 × 10 mL). The combined organic layers were dried over Na_2_SO_4_, filtered, and concentrated *in vacuo*. ^13^C{^1^H} NMR spectroscopic analysis of the unpurified reaction mixture revealed that *p*-nitrobenzoate **22b** was formed as a mixture of diastereomers (*trans-***22b**:*cis*-**22b** = 75:25).^14^ The spectral data obtained are consistent with those of products from nucleophilic substitution reactions of *p*-nitrobenzoate **21b** and allyltrimethylsilane in the presence of SnCl_4_.

#### (2R*,3S*)-2-Allyltetrahydrofuran-3-yl 4-Nitrobenzoate (*trans*-**22b**) and (2R*,3R*)-2-Allyltetrahydrofuran-3-yl 4-Nitrobenzoate (*cis*-**22b**)

The general procedure for nucleophilic substitution reactions of acetates was followed using *p*-nitrobenzoate **21b** (0.0800 g, 0.199 mmol), allyltrimethylsilane (0.158 mL, 0.995 mmol), and SnCl_4_ (0.500 mL, 0.50 mmol, 1.0 M in CH_2_Cl_2_) in CH_2_Cl_2_ (1.5 mL). ^13^C{^1^H} NMR spectroscopic analysis of the unpurified reaction mixture revealed that *p*-nitrobenzoate **22b** was formed as a mixture of diastereomers (*trans-***22b**:*cis-***22b** = 72:28).^14^ Purification by flash column chromatography (20:80 EtOAc:hexanes) afforded *p*-nitrobenzoate *trans-***22b** as a yellow oil (0.0273 g, 49%) and *cis-***22b** as a yellow oil (0.0100 g, 18%). Major diastereomer *trans-***22b**: ^1^H NMR (400 MHz, CDCl_3_) δ 8.32–8.27 (m, 2H), 8.22–8.18 (m, 2H), 5.92–5.80 (m, 1H), 5.27 (dt, *J* = 6.5, 2.2 Hz, 1H), 5.18 (dq, *J* = 17.2, 1.7 Hz, 1H), 5.15–5.11 (m, 1H), 4.13–4.06 (m, 2H), 3.97 (dt, *J* = 9.9, 6.1 Hz, 1H), 2.47–2.38 (m, 2H), 2.28–2.26 (m, 1H), 2.15–2.08 (m, 1H); ^13^C{^1^H} NMR (100 MHz, CDCl_3_) δ 164.4 (C), 150.8 (C), 135.5 (C), 133.7 (CH), 130.9 (CH), 123.7 (CH), 118.1 (CH_2_), 83.4 (CH), 79.6 (CH), 67.1 (CH_2_), 37.9 (CH_2_), 32.6 (CH_2_); IR (ATR) 2868, 1722, 1527, 1270, 1101, 748 cm^–1^; HRMS (TOF MS ES^+^) *m*/*z* calcd for C_14_H_17_N_2_O_4_ (M + NH_4_ – H_2_O)^+^ 277.1183, found 277.1192. Minor diastereomer *cis-***22b**: ^1^H NMR (400 MHz, CDCl_3_) δ 8.33–8.28 (m, 2H), 8.25–8.21 (m, 2H), 5.90–5.77 (m, 1H), 5.58 (ddd, *J* = 7.7, 3.8, 1.7 Hz, 1H), 5.13–5.03 (m, 2H), 4.13 (q, *J* = 7.9 Hz, 1H), 3.93 (ddd, *J* = 7.7, 6.2, 3.7 Hz, 1H), 3.90–3.84 (m, 1H), 2.56–2.39 (m, 3H), 2.20–2.10 (m, 1H); ^13^C{^1^H} NMR (100 MHz, CDCl_3_) δ 164.2 (C), 150.8 (C), 135.5 (C), 134.2 (CH), 130.9 (CH), 123.8 (CH), 117.6 (CH_2_), 81.2 (CH), 76.3 (CH), 66.3 (CH_2_), 33.9 (CH_2_), 33.7 (CH_2_); IR (ATR) 2927, 1723, 1527, 1271, 1102, 718 cm^–1^; HRMS (TOF MS ES^+^) *m*/*z* calcd for C_14_H_16_NO_5_ (M + H)^+^ 278.1023, found 278.1030.

#### (2R*,3S*)-2-Allyltetrahydrofuran-3-yl 4-Nitrobenzoate (*trans*-**22b**) and (2R*,3R*)-2-Allyltetrahydrofuran-3-yl 4-Nitrobenzoate (*cis*-**22b**) Formed Using BF_3_•OEt_2_ as the Lewis Acid

To a solution of *p*-nitrobenzoate **21b** (0.200 g, 0.497 mmol) in CH_2_Cl_2_ (5 mL) at −45 °C was added allyltrimethylsilane (0.316 mL, 1.99 mmol) followed by the addition of BF_3_•OEt_2_ (0.123 mL, 0.994 mmol). After 1 h, the reaction mixture was warmed to 25 °C. After 16 h, a solution of saturated aqueous NaHCO_3_ (5 mL) was added and the layers were separated. The aqueous layer was extracted with CH_2_Cl_2_ (2 × 10 mL). The combined organic layers were dried over Na_2_SO_4_, filtered, and concentrated *in vacuo*. ^13^C{^1^H} NMR spectroscopic analysis of the unpurified reaction mixture revealed that *p*-nitrobenzoate **22b** was formed as a mixture of diastereomers (*trans*-**22b**:*cis*-**22b** = 60:40).^14^ The spectral data obtained are consistent with those of products from nucleophilic substitution reactions of *p*-nitrobenzoate **21b** and allyltrimethylsilane in the presence of SnCl_4_.

#### (2R*,3S*)-2-Allyltetrahydrofuran-3-yl 4-Nitrobenzoate (*trans*-**22b**) Formed Using Me_3_SiOTf as the Lewis Acid

The general procedure for nucleophilic substitution reactions of acetates was followed using *p*-nitrobenzoate **21b** (0.0900 g, 0.224 mmol), allyltributylstannane (0.278 mL, 0.896 mmol), and Me_3_SiOTf (0.120 mL, 0.663 mmol) in CH_2_Cl_2_ (2 mL). ^13^C{^1^H} NMR spectroscopic analysis of the unpurified reaction mixture revealed that *p*-nitrobenzoate *trans*-**22b** was formed as a single diastereomer.^14^ The spectral data obtained are consistent with those of products from nucleophilic substitution reactions of *p*-nitrobenzoate **21b** and allyltrimethylsilane in the presence of SnCl_4_.

#### (2R*,3S*)-2-Allyltetrahydrofuran-3-yl 4-Nitrobenzoate (*trans*-**22b**) and (2R*,3R*)-2-Allyltetrahydrofuran-3-yl 4-Nitrobenzoate (*cis*-**22b**) Formed Using BF_3_•OEt_2_ as the Lewis Acid

To a solution of *p*-nitrobenzoate **21b** (0.200 g, 0.497 mmol) in CH_2_Cl_2_ (5 mL) at −45 °C was added allyltributylstannane (0.616 mL, 1.99 mmol) followed by the addition of BF_3_•OEt_2_ (0.123 mL, 0.994 mmol). After 1 h, the reaction mixture was warmed to 25 °C. After 16 h, a solution of saturated aqueous NaHCO_3_ (5 mL) was added and the layers were separated. The aqueous layer was extracted with CH_2_Cl_2_ (2 × 10 mL). The combined organic layers were dried over Na_2_SO_4_, filtered, and concentrated *in vacuo*. ^13^C{^1^H} NMR spectroscopic analysis of the unpurified reaction mixture revealed that *p*-nitrobenzoate **22b** was formed as a mixture of diastereomers (*trans*-**22b**:*cis*-**22b** = 90:10).^14^ The spectral data obtained are consistent with those of products from nucleophilic substitution reactions of *p*-nitrobenzoate **21b** and allyltrimethylsilane in the presence of SnCl_4_.

## Data Availability

The data underlying this study are available in the published article and its Supporting Information.

## Supporting Information Available

Procedures for substrate synthesis, detailed proofs of stereochemistry of critical compounds, control experiments, and computational methods (PDF)
