# Supplementary material for: Neighboring-Group Participation by a Less Electron-Donating, Participating C-2-Ester Ensures Higher 1,2-trans Stereoselectivity in Nucleophilic Substitution Reactions of Furanosyl Acetals
Source: J Org Chem. 2025 Jan 15;90(4):1585–96. doi: 10.1021/acs.joc.4c02612 (PMC11791888; doi:10.1021/acs.joc.4c02612)
Supplement: Supplementary file 1 — jo4c02612_si_001.pdf [file jo4c02612_si_001.pdf]

Supporting Information

Neighboring-group Participation by Less Electron-donating, Participating C-2-Ester Ensures Higher 1,2-*Trans* Stereoselectivity in Nucleophilic Substitution Reactions of Furanosyl Acetals

Yuge Chun,[a] Wouter A. Remmerswaal,[b] Jeroen D. C. Codée,*[b] and K. A. Woerpel*[a]

[a] Y. Chun, Prof. K. A. Woerpel
Department of Chemistry, New York University
100 Washington Square East, New York, NY 10003 (USA)
E-mail: kwoerpel@nyu.edu

[b] W. A. Remmerswaal, Prof. J. D. C. Codée
Leiden Institute of Chemistry, Leiden University
Einsteinweg 55, 2300 RA Leiden (The Netherlands)
E-mail: jcodee@chem.leidenuniv.nl

Table of Contents

I. General Experimental S3

II. Substrate Synthesis S3

III. Nucleophilic Substitution Reactions of Furanosyl Thiocetals with *O*-nucleophiles S6

IV. Nucleophilic Substitution Reactions of Furanosyl Acetals with *C*-nucleophiles S17

V. Parameter Screen for Substitution Reactions with 2,2,2-Trifluoroethanol S24

VI. Parameter Screen for Substitution Reactions with Allyltrimethylsilane S27

VII. Stereochemical Proofs S29

VIII. Epimerization Nucleophilic Substitution Studies S39

IX. Computational methods S44

X. References S67

XI. Selected ^{1}H and $^{13}C\{^{1}H\}$ NMR Spectra S67

I. General Experimental

^1H and $^{13}C\{^1H\}$ NMR Spectra were acquired at room temperature using Bruker AVIII-400 (400 MHz and 100 MHz, respectively) and AVIII-600 (600 MHz) spectrometers as indicated. The diastereomeric ratios of nucleophilic substitution reactions were obtained by $^{13}C\{^1H\}$ spectroscopic analysis of the representative peaks of products in the unpurified crude reaction mixture.[1] All spectroscopic data were reported as follows: chemical shifts reported in ppm as referenced to solvent peaks (^1H NMR: $CDCl_3$ δ 7.26 ppm; $^{13}C\{^1H\}$ NMR: $CDCl_3$ δ 77.16 ppm), multiplicity (s = singlet, br = broad, br s = broad singlet, d = doublet, ddt = doublet of doublet of triplet, t = triplet, dd = doublet of doublet, dt = doublet of triplet, m = multiplet), *J* coupling constants (Hz), and integration. High-resolution mass spectra (Hrms) were acquired using an Agilent 6224 Accurate-Mass time-of-flight spectrometer through ESI (electrospray ionization) mode. Infrared (IR) data were acquired using Nicolet 6700 FT-IR spectrometer via attenuated total reflectance (ATR). All reactions were performed under inert nitrogen atmosphere using glassware that has been flame-dried under reduced pressure. Solvents including dichloromethane, acetonitrile, toluene, and methanol were anhydrous and degassed through a solvent purification system prior to use in the reported reactions. Aqueous solutions were prepared using distilled water. Flash column chromatography was performed using the solvent system on silica gel (SiO_2) 60 (230–400 mesh) under air flow. All reagents were commercially available unless otherwise notified.

II. Substrate Synthesis

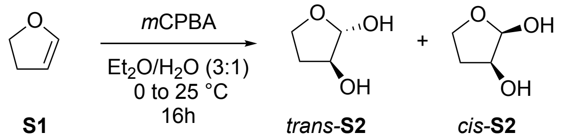

(2*R,3*S**)-Tetrahydrofuran-2,3-diol (*trans*-S2) and (2*R**,3*R**)-tetrahydrofuran-2,3-diol (*cis*-S2).** To a solution of *m*CPBA (75% in H_2O, 7.98 g, 34.2 mmol) in Et_2O/H_2O (3:1, 100 mL) at 0 °C was added 2,3-dihydrofuran (**S1**, 2.16 mL, 28.5 mmol) dropwise. The reaction mixture was stirred at 0 °C for an additional 30 min and then warmed to 25 °C. After 16 h, the ethereal solution was extracted with H_2O (2 × 100 mL). The combined aqueous layer was washed with Et_2O (2 × 100 mL), and the combined organic layers were

concentrated *in vacuo* to provide diol **S2** (1.64 g, 53%) as a colorless oil. The oil was used without further purification. The spectral data obtained are consistent with those reported in the literature.

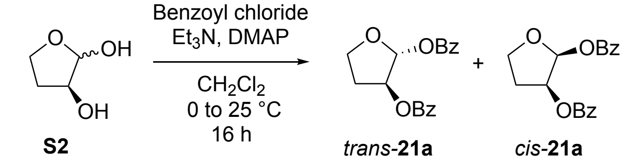

(2*R,3*S**)-Tetrahydrofuran-2,3-diyl dibenzoate (*trans*-21a) and (2*R**,3*R**)-tetrahydrofuran-2,3-diyl dibenzoate (*cis*-21a).** To a solution of diol **S2** (0.633 g, 6.08 mmol) in CH_2Cl_2 (6 mL) at 0 °C was added Et_3N (3.39 mL, 24.3 mmol) and *N,N*-dimethylaminopyridine (0.149 g, 1.22 mmol). After 30 min, benzoyl chloride (2.85 mL, 24.3 mmol) was added dropwise. The reaction mixture was stirred at 0 °C for an additional 30 min and then it was warmed to 25 °C. After 16 h, the reaction mixture was diluted with CH_2Cl_2 (20 mL) and a solution of saturated aqueous NH_4Cl (30 mL) was added. The aqueous layer was extracted with CH_2Cl_2 (2 × 30 mL). The combined organic layers were dried over Na_2SO_4, filtered, and concentrated *in vacuo*. Purification by flash chromatography (15:85 EtOAc:hexanes) afforded dibenzoate **21a** as a yellow solid (0.744 g, 39%) as a mixture of diastereomers (*trans*-**21a**:*cis*-**21a** = 80:20). The spectral data obtained are consistent with those reported in the literature.[2]

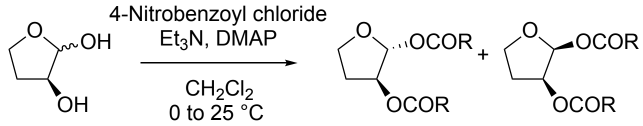

(2*R,3*S**)-Tetrahydrofuran-2,3-diyl bis(4-nitrobenzoate) (*trans*-21b) and (2*R**,3*R**)-tetrahydrofuran-2,3-diyl bis(4-nitrobenzoate) (*cis*-21b).** To a solution of diol **S2** (0.633 g, 6.08 mmol) in CH_2Cl_2 (8 mL) at 0 °C was added Et_3N (3.40 mL, 24.3 mmol) and *N,N*-dimethylaminopyridine (0.149 g, 1.22 mmol). After 30 min, 4-nitrobenzoyl chloride (4.51 g, 24.3 mmol) was added. The reaction mixture was stirred at 0 °C for an additional 30 min and then it was warmed to 25 °C. After 16 h, the reaction mixture was diluted with CH_2Cl_2 (20 mL) and a solution of saturated aqueous NH_4Cl (30 mL) was added. The aqueous layer was extracted with CH_2Cl_2 (2 × 30 mL). The combined organic layers were dried over Na_2SO_4, filtered, and concentrated *in vacuo*. Purification by flash chromatography (30:70 EtOAc:hexanes) afforded *p*-nitrobenzoate **21b** as a white solid (1.79 g, 74%) as a mixture of diastereomers (*trans*-**21b**:*cis*-**21b** = 40:60):

^{1}H NMR (400 MHz, $CDCl_3$) δ 8.36–8.21 (m, 0.7H), 8.37–8.30 (m, 4H), 8.30–8.22 (m, 4.2H), 8.21–8.20 (m, 0.7H), 8.17–8.10 (m, 4H), 6.74 (d, J = 4.2 Hz, 1H), 6.62 (br s, 0.7H), 5.68 (dd, J = 5.8, 1.5 Hz, 0.7H), 5.58 (td, J = 8.6, 4.4 Hz, 1H), 4.44–4.30 (m, 2.4H), 4.20 (dt, J = 8.7, 7.9 Hz, 1H), 2.71–2.60 (m, 1.7H), 2.46–2.29 (m, 1.7H);

Peaks attributed to the minor diastereomer *trans*-**21b**: $^{13}C\{^1H\}$ NMR (100 MHz, $CDCl_3$) δ 163.96 (C), 163.4 (C), 151.05 (C), 150.97 (C, overlapping major and minor diastereomer), 135.0 (C), 134.7 (C), 131.9 (CH), 131.12 (CH), 123.9 (CH), 123.82 (CH), 101.1 (CH), 78.6 (CH), 68.9 (CH_2), 29.9 (CH_2);

Peaks attributed to the major diastereomer *cis*-**21b**: $^{13}C\{^1H\}$ NMR (100 MHz, $CDCl_3$) δ 163.98 (C), 163.6 (C), 151.00 (C), 150.97 (C, overlapping major and minor diastereomer), 135.1 (C), 134.6 (C), 131.1 (CH), 130.9 (CH), 123.8 (CH), 123.85 (CH), 95.9 (CH), 74.1 (CH), 67.0 (CH_2), 27.8 (CH_2);

IR (ATR) 2908, 1725, 1524, 1257, 1099, 715 cm^{-1};

HRMS (TOF MS ES$^+$) m / z calcd for $C_{18}H_{14}N_2NaO_9$ $(M + Na)^+$ 425.0592, found 425.0599;

Melting point: 142–147 °C.

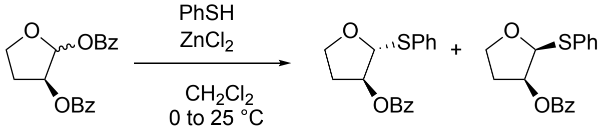

(2*R,3*S**)-2-(Phenylthio)tetrahydrofuran-3-yl benzoate (*trans*-6a) and (2*R**,3*R**)-2-(phenylthio)tetrahydrofuran-3-yl benzoate (*cis*-6a).** To a solution of dibenzoate **21a** (0.340 g, 1.09 mmol) in CH_2Cl_2 (11 mL) at –78 °C was added thiophenol (0.134 mL, 1.31 mmol) dropwise. The reaction mixture was stirred at –78 °C for 20 min and $ZnCl_2$ (0.418 g, 1.31 mmol) was added. The reaction mixture was stirred at –78 °C for an additional 30 min and then it was warmed to 25 °C. After 16 h, a solution of saturated aqueous $NaHCO_3$ (15 mL) was added. The aqueous layer was extracted with CH_2Cl_2 (2 × 20 mL). The combined organic layers were dried over Na_2SO_4, filtered, and concentrated *in vacuo*. Purification by flash chromatography (5:95 EtOAc:hexanes) afforded thioacetal **6a** as a colorless oil (0.120 g, 37%) as a mixture of diastereomers (*trans*-**6a**:*cis*-**6a** = 83:17). The spectral data obtained are consistent with those reported in the literature.[2]

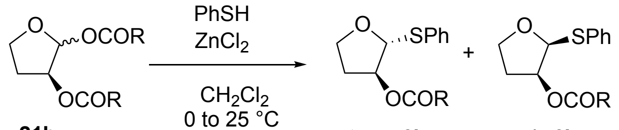

21b
R = 4-$NO_2C_6H_4$

trans-**6b** *cis*-**6b**
R = *p*-$NO_2C_6H_4$

(2*R,3*S**)-2-(Phenylthio)tetrahydrofuran-3-yl 4-nitrobenzoate (*trans*-6b) and (2*R**,3*R**)-2-(phenylthio)tetrahydrofuran-3-yl 4-nitrobenzoate (*cis*-6b).** To a solution of *p*-nitrobenzoate **21b** (0.467 g, 1.16 mmol) in CH_2Cl_2 (12 mL) at –78 °C was added thiophenol (0.143 mL, 1.39 mmol) dropwise. The reaction mixture was stirred at –78 °C for 20 min and $ZnCl_2$ (0.444 g, 1.39 mmol) was added. The reaction mixture was stirred at –78 °C for an additional 30 min and then it was warmed to 25 °C. After 16 h, a solution of saturated aqueous $NaHCO_3$ (15 mL) was added. The aqueous layer was extracted with CH_2Cl_2 (2 × 20 mL). The combined organic layers were dried over Na_2SO_4, filtered, and concentrated *in vacuo*. Purification by flash chromatography (10:90 EtOAc:hexanes) afforded thioacetal **6b** as a yellow oil (0.176 g, 44%) as a mixture of diastereomers (*trans*-**6b**:*cis*-**6b** = 84:16). The spectral data obtained are consistent with those reported in the literature.[2]

III. Nucleophilic Substitution Reactions of Furanosyl Thiocetals with *O*-nucleophiles

General procedure for nucleophilic substitution reactions of furanosyl thioacetals using oxygen nucleophiles. To a solution of thioacetal (1.0 equiv) in solvent (0.1 M) at –45 °C was added nucleophile (6.0 equiv unless otherwise noted), followed by the addition of *N*-iodosuccinimide (2.0 equiv unless otherwise noted). The reaction mixture was then stirred at –45 °C for 16 h. A solution of Me_2S:CH_2Cl_2:Et_3N (1:1:1, 1 mL per mmol of thioacetal) was added at –45 °C and the reaction mixture was warmed to room temperature. The reaction mixture was then diluted with CH_2Cl_2 (1 × 20 mL per mmol of thioacetal) and washed with saturated aqueous Rochelle's salt solution (1 × 20 mL per mmol of acetate). The aqueous layer was extracted with CH_2Cl_2 (2 × 20 mL per mmol of acetate). The combined organic layers were dried over Na_2SO_4, filtered, and concentrated *in vacuo*. The diastereomeric ratios were determined by $^{13}C\{^1H\}$ NMR analysis of the unpurified reaction mixture.[1] The reaction mixture was purified by flash column chromatography to provide products. The relative stereochemical configurations of products were assigned by analysis of coupling constants. Details of stereochemical proofs were provided in section VII.

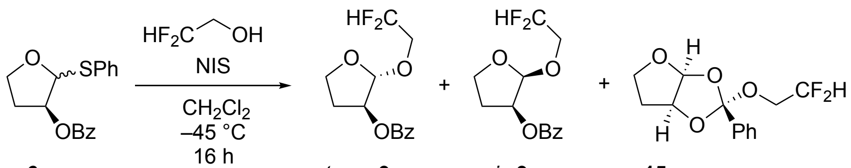

(2*R,3*S**)-2-(2,2-Difluoroethoxy)tetrahydrofuran-3-yl benzoate (*trans*-9a), (2*R**,3*R**)-2-(2,2-difluoroethoxy)tetrahydrofuran-3-yl benzoate (*cis*-9a).** The general procedure for nucleophilic substitution reactions of thioacetals was followed using thioacetal **6a** (0.300 g, 1.00 mmol), 2,2-difluoroethanol (0.380 mL, 6.0 mmol), and *N*-iodosuccinimide (0.500 g, 2.00 mmol) in CH_2Cl_2 (10 mL). $^{13}C\{^1H\}$ NMR spectroscopic analysis of the unpurified reaction mixture revealed that acetal **9a** was formed as a mixture of diastereomers (*trans*-**9a**:*cis*-**9a** = 93:7).[1] Purification by flash column chromatography (10:90 EtOAc:hexanes) afforded acetal *trans*-**9a** as a yellow oil (0.117 g, 43%) and acetal *cis*-**9a** as a yellow oil as a mixture with side product *trans*-**21a** (0.0400 g, 18%, *cis*-**9a**:*trans*-**9a**= 16:84).

Note: Nucleophilic substitution reactions of thioacetal **6a** with 2,2-difluoroethanol were low-yielding because of the formation of orthoester side-product **15a**, which was observed in the reaction mixture but decomposed in the sequential purification step. Purification by column chromatography afforded acetal *cis*-**9a** as a mixture with an inseparable decomposition product that appeared after purification, which was identified to be dibenzoate *trans*-**21a** (*cis*-**9a**:*trans*-**21a** = 16:84).

Major diastereomer *trans*-**9a**: ^1H NMR (400 MHz, $CDCl_3$) δ 8.05–8.00 (m, 2H), 7.62–7.55 (m, 1H), 7.49–7.41 (m, 2H), 5.92 (tt, J = 55.8, 4.4 Hz, 1H), 5.39 (dd, J = 5.9, 1.7 Hz, 1H), 5.21 (br s, 1H), 4.20 (q, J = 8.0 Hz, 1H), 4.09 (td, J = 8.9, 4.8 Hz, 1H), 3.91–3.80 (m, 1H), 3.79–3.70 (m, 1H), 2.55–2.43 (m, 1H), 2.15–2.05 (m, 1H);

$^{13}C\{^1H\}$ NMR (100 MHz, $CDCl_3$) δ 165.9 (C), 136.7 (C), 133.5 (CH), 129.8 (CH), 128.6 (CH), 114.3 (br t, $^1J_{C–F}$ = 239.0 Hz, CH), 106.2 (CH), 78.1 (CH), 67.2 (CH_2), 66.3 (br t, $^2J_{C–F}$ = 28.6 Hz, CH_2), 29.9 (CH_2);

IR (ATR) 2903, 1719, 1265, 1100, 1051, 711 cm^{-1};

HRMS (TOF MS ES^+) m / z calcd for $C_{13}H_{14}F_2NaO_4$ (M + NH_4)$^+$ 290.1198, found 290.1178.

Minor diastereomer *cis*-**9a**: ^{1}H NMR (400 MHz, $CDCl_3$, characteristic peaks) δ 5.84 (tt, J = 56.3, 4.4 Hz, 1H), 5.30 (d, J = 4.2 Hz, 1H), 5.17 (td, J = 8.6, 4.3 Hz, 1H), 4.16–4.08 (m, 1H), 3.97 (q, J = 8.0 Hz, 1H), 2.47–2.38 (m, 1H);

^{13}C{^{1}H} NMR (100 MHz, $CDCl_3$, characteristic peaks) 166.4 (C), 136.7 (C), 134.1 (CH), 114.3 (br t, $^1J_{C–F}$ = 242.2 Hz, CH), 105.9 (CH), 73.9 (CH), 67.2 (br t, $^2J_{C–F}$ = 29.3 Hz, CH_2), 64.8 (CH_2), 27.5 (CH_2);

IR (ATR) 2980, 1719, 1261, 1061, 944, 708 cm^{-1};

HRMS (TOF MS ES$^+$) *m* / *z* calcd for $C_{13}H_{14}F_2NaO_4$ (M + Na)$^+$ 295.0752, found 295.0764.

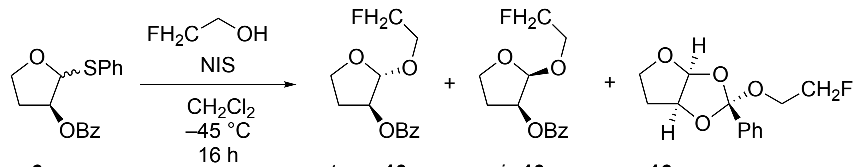

6a *trans*-**10a** *cis*-**10a** **16a**

(2*R,3*S**)-2-(2-Fluoroethoxy)tetrahydrofuran-3-yl benzoate (*trans*-10a), (2*R**,3*R**)-2-(2-fluoroethoxy)tetrahydrofuran-3-yl benzoate (*cis*-10a) and (2*R**,3a*R**,6a*S**)-2-ethoxy-2-phenyltetrahydrofuro[2,3-*d*][1,3]dioxole (16a).** The general procedure for nucleophilic substitution reactions of thioacetals was followed using thioacetal **6a** (0.300 g, 1.00 mmol), 2-fluoroethanol (0.352 mL, 6.00 mmol), and *N*-iodosuccinimide (0.450 g, 2.00 mmol) in CH_2Cl_2 (10 mL). ^{13}C{^{1}H} NMR spectroscopic analysis of the unpurified reaction mixture revealed that acetal **10a** was formed as a mixture of diastereomers (*trans*-**10a**:*cis*-**10a** = 65:35).[1] Purification by flash column chromatography (15:85 EtOAc:hexanes) afforded acetal *trans*-**10a** as a yellow oil (0.0400 g, 16%) and acetal *cis*-**10a** as a mixture with side-product orthoester **16a** as a yellow oil (0.0290 g, 12%, *cis*-**10a**:**16a** = 33:67).

Major diastereomer *trans*-**10a**: ^{1}H NMR (400 MHz, $CDCl_3$) δ 8.06–8.00 (m, 2H), 7.61–7.54 (m, 1H), 7.48–7.41 (m, 2H), 5.39 (dt, J = 5.9, 1.9 Hz, 1H), 5.21 (br s, 1H), 4.63 (t, J = 3.9 Hz, 1H), 4.52 (t, J = 3.9 Hz, 1H), 4.17 (dt, J = 15.9, 8.1 Hz, 1H), 4.09 (ddd, J = 16.0, 8.7, 5.0 Hz, 1H), 3.99–3.84 (m, 1H), 3.82–3.68 (m, 1H), 2.56–2.44 (m, 1H), 2.13–2.04 (m, 1H);

^{13}C{^{1}H} NMR (100 MHz, $CDCl_3$) δ 166.0 (C), 133.4 (C), 129.92 (CH), 129.85 (CH), 128.6 (CH), 106.0 (CH), 82.9 (br d, $^1J_{C–F}$ = 171.1 Hz, CH_2), 78.3 (CH), 66.9 (CH_2), 66.3 (br d, $^2J_{C–F}$ = 20.0 Hz, CH_2), 30.1 (CH_2);

IR (ATR) 2928, 1718, 1266, 1044, 1024, 710 cm^{-1};

HRMS (TOF MS ES$^+$) *m* / *z* calcd for $C_{13}H_{19}FNO_4$ (M + NH_4)$^+$ 272.1393, found 272.1415.

Minor diastereomer *cis*-**10a** and orthoester **16a** (*cis*-**10a**:**16a** = 33:67): ^{1}H NMR (400 MHz, $CDCl_3$, characteristic peaks) δ 6.09 (d, J = 4.2 Hz, 1H), 5.30 (d, J = 4.4 Hz, 0.5H), 5.17 (td, J = 8.5, 4.1 Hz, 0.5H), 5.03 (t, J = 4.5 Hz, 1H), 2.45–2.36 (m, 0.5H), 2.33–2.22 (m, 0.5H), 2.11 (dd, J = 14.5, 4.6 Hz, 1H), 1.94–1.83 (m, 1H);

Peaks attributed to acetal *cis*-**10a**: ^{13}C{^{1}H} NMR (100 MHz, $CDCl_3$, characteristic peaks) δ 166.5 (C), 100.4 (CH), 82.7 (br d, $^{1}J_{C-F}$ = 169.5 Hz, CH_2), 74.0 (CH), 64.5 (CH_2), 63.4 (br d, $^{2}J_{C-F}$ = 20.5 Hz, CH_2), 26.7 (CH_2);

Peaks attributed to orthoester **16a**: ^{13}C{^{1}H} NMR (100 MHz, $CDCl_3$, characteristic peaks) δ 144.3 (C), 106.0 (CH), 82.3 (br d, $^{2}J_{C-F}$ = 170.2 Hz, CH_2), 81.3 (CH), 67.0 (CH_2), 62.8 (br d, $^{2}J_{C-F}$ = 20.2 Hz, CH_2), 33.1 (CH_2);

IR (ATR) 2956, 1717, 1263, 1103, 1024, 781 cm^{-1};

HRMS (TOF MS ES^{+}) m / z calcd for $C_{13}H_{15}FNaO_4$ (M + Na)$^{+}$ 277.0847, found 277.0849.

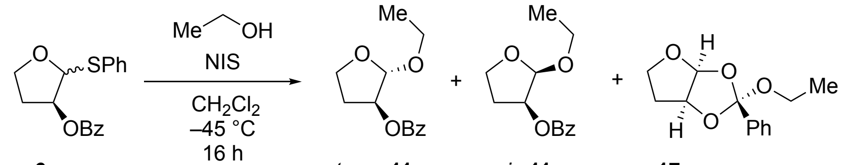

6a trans-11a cis-11a 17a

(2*R,3*S**)-2-Ethoxytetrahydrofuran-3-yl benzoate (*trans*-11a) and (2*R**,3*R**)-2-ethoxytetrahydrofuran-3-yl benzoate (*cis*-11a).** The general procedure for nucleophilic substitution reactions of thioacetals was followed using thioacetal **6a** (0.300 g, 1.00 mmol), ethanol (1.18 mL, 20.0 mmol), and *N*-iodosuccinimide (1.35 g, 6.00 mmol) in CH_2Cl_2 (10 mL). ^{13}C{^{1}H} NMR spectroscopic analysis of the unpurified reaction mixture revealed that acetal **11a** was formed as a mixture of diastereomers (*trans*-**11a**:*cis*-**11a** = 70:30).[1] Purification by flash column chromatography (10:90 EtOAc:hexanes) afforded acetal *trans*-**11a** as a yellow oil (0.0380 g, 16%) and acetal *cis*-**11a** as a yellow oil (0.0100 g, 4%) as a mixture of diastereomers (*trans*-**11a**:*cis*-**11a** = 37:63).

Note: Nucleophilic substitution reactions of thioacetal **6a** with ethanol were low-yielding because of the formation of orthoester side-product **17a**, which was observed in the reaction mixture but decomposed in the sequential purification step. Purification by column chromatography afforded acetal *cis*-**11a** as a mixture with acetal *trans*-**11a** (*trans*-**11a**:*cis*-**11a** = 37:63) and an inseparable decomposition product that appeared after purification, which was identified to be dibenzoate *trans*-**21a**.

Major diastereomer *trans*-**11a**: ^{1}H NMR (400 MHz, $CDCl_3$) δ 8.07–7.99 (m, 2H), 7.59–7.50 (m, 1H), 7.47–7.38 (m, 2H), 5.33 (dd, J = 6.2, 1.5 Hz, 1H), 5.16 (br s, 1H), 4.20–4.03 (m, 2H), 3.81–3.71 (m, 1H), 3.57–3.48 (m, 1H), 2.53–2.42 (m, 1H), 2.10–2.01 (m, 1H), 1.22 (t, J = 7.3 Hz, 3H);

$^{13}C\{^1H\}$ NMR (100 MHz, $CDCl_3$) δ 166.0 (C), 133.3 (C), 130.0 (CH), 129.8 (CH), 128.5 (CH), 105.6 (CH), 78.4 (CH), 66.6 (CH_2), 62.9 (CH_2), 30.1 (CH_2), 15.2 (CH_3);

IR (ATR) 2977, 1718, 1267, 1097, 1045, 709 cm^{-1};

HRMS (TOF MS ES^+) m / z calcd for $C_{13}H_{16}NaO_4$ $(M + Na)^+$ 259.0941, found 259.0949.

Minor diastereomer *cis*-**11a**: ^{1}H NMR (400 MHz, $CDCl_3$, characteristic peaks) δ 5.25 (d, J = 4.6 Hz, 1H), 5.15 (td, J = 8.4, 4.4 Hz, 1H), 4.32–4.24 (m, 1H), 1.12 (t, J = 6.9 Hz, 3H);

$^{13}C\{^1H\}$ NMR (100 MHz, $CDCl_3$, characteristic peaks) δ 166.5 (C), 100.1 (CH), 74.1 (CH), 64.3 (CH_2), 63.9 (CH_2), 28.0 (CH_2), 15.4 (CH_3);

IR (ATR) 2899, 1721, 1271, 1104, 1027, 712 cm^{-1};

HRMS (TOF MS ES^+) m / z calcd for $C_{13}H_{18}NO_3$ $(M + NH_4 - H_2O)^+$ 236.1281, found 236.1292.

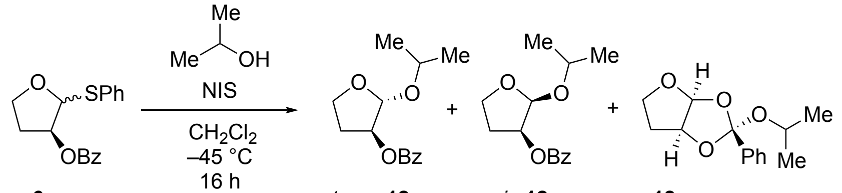

(2*R,3*S**)-2-Isopropoxytetrahydrofuran-3-yl benzoate (*trans*-12a), (2*R**,3*R**)-2-isopropoxytetrahydrofuran-3-yl benzoate (*cis*-12a) and (2*R**,3a*R**,6a*S**)-2-isopropoxy-2-phenyltetrahydrofuro[2,3-*d*][1,3]dioxole (18a).** The general procedure for nucleophilic substitution reactions of thioacetals was followed using thioacetal **6a** (0.300 g, 1.00 mmol), isopropyl alcohol (0.763 mL, 10.0 mmol), and *N*-iodosuccinimide (0.500 g, 2.00 mmol) in CH_2Cl_2 (10 mL). $^{13}C\{^1H\}$ NMR spectroscopic analysis of the unpurified reaction mixture revealed that acetal **12a** was formed as a mixture of diastereomers (*trans*-**12a**:*cis*-**12a** = 76:24).[1] Purification by flash column chromatography (10:90 EtOAc:hexanes) afforded acetal *trans*-**12a** as a yellow oil (0.111 g, 44%), acetal *cis*-**12a** as a yellow oil (0.0141 g, 6%) as an inseparable mixture with side-product isopropyl benzenesulfinate (*cis*-**12a**:isopropyl benzenesulfinate = 67:33), and orthoester **18a** as a yellow oil (0.113 g, 45%).

Major diastereomer *trans*-**12a**: ^{1}H NMR (400 MHz, $CDCl_3$) δ 8.06–8.00 (m, 2H), 7.58–7.51 (m, 1H), 7.47–7.41 (m, 2H), 5.29 (dd, J = 5.9, 1.6 Hz, 1H), 5.25 (br s, 1H), 4.17–4.03 (m, 2H), 3.98–3.89 (m, 1H), 2.53–2.42 (m, 1H), 2.09–2.00 (m, 1H), 1.20 (d, J = 6.1 Hz, 3H), 1.19 (d, J = 6.6 Hz, 3H);

$^{13}C\{^1H\}$ NMR (100 MHz, $CDCl_3$) δ 166.1 (C), 134.7 (C), 133.3 (CH), 129.8 (CH), 128.5 (CH), 104.1 (CH), 78.9 (CH), 69.2 (CH), 66.4 (CH_2), 30.2 (CH_2), 23.6 (CH_3), 21.9 (CH_3);

IR (ATR) 2975, 1788, 1211, 1034, 703, 614 cm^{-1};

HRMS (TOF MS ES$^+$) m / z calcd for $C_{14}H_{17}O_3$ $(M + H – H_2O)^+$ 233.1172, found 233.1171.

Minor diastereomer *cis*-**12a**: ^{1}H NMR (400 MHz, $CDCl_3$) δ 8.10–8.02 (m, 2H), 7.77–7.67 (m, 1H), 7.49–7.40 (m, 2H), 5.35 (d, J = 4.4 Hz, 1H), 5.12 (td, J = 8.5, 4.2 Hz, 1H), 4.16–4.05 (m, 1H), 3.95–3.79 (m, 2H), 2.42–2.32 (m, 1H), 2.30–2.20 (m, 1H), 1.19 (d, J = 6.7 Hz, 3H), 1.03 (d, J = 6.0 Hz, 3H);

$^{13}C\{^1H\}$ NMR (100 MHz, $CDCl_3$) 166.5 (C), 136.7 (C), 133.2 (CH), 129.9 (CH), 128.5 (CH), 99.3 (CH), 74.1 (CH), 71.0 (CH), 64.0 (CH_2), 27.9 (CH_2), 23.6 (CH_3), 22.2 (CH_3);

IR (ATR) 2974, 1720, 1271, 1117, 1029, 712 cm^{-1};

HRMS (TOF MS ES$^+$) m / z calcd for $C_{14}H_{20}NO_3$ $(M + NH_4 – H_2O)^+$ 250.1438, found 250.1437.

Orthoester **18a**: ^{1}H NMR (400 MHz, $CDCl_3$) δ 7.67–7.63 (m, 2H), 7.38–7.34 (m, 3H), 6.61 (d, J = 4.0 Hz, 1H), 5.00 (t, J = 4.4 Hz, 1H), 3.88 (t, J = 7.8 Hz, 1H), 3.79–3.69 (m, 1H), 3.60 (ddd, J = 13.3, 8.6, 4.6 Hz, 1H), 2.08 (dd, J = 13.7, 4.9 Hz, 1H), 1.93–1.82 (m, 1H), 1.15 (d, J = 6.2 Hz, 3H), 1.17 (d, J = 6.2 Hz, 3H);

$^{13}C\{^1H\}$ NMR (100 MHz, $CDCl_3$) δ 138.1 (C), 129.2 (CH), 128.2 (CH), 126.3 (CH), 123.1 (C), 105.9 (CH), 81.0 (CH), 67.0 (CH_2), 66.6 (CH), 33.3 (CH_2), 23.7 (CH_3), 23.6 (CH_3).

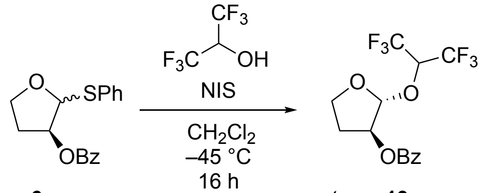

6a *trans*-**13a**

(2*R,3*S**)-2-((1,1,1,3,3,3-Hexafluoropropan-2-yl)oxy)tetrahydrofuran-3-yl benzoate (*trans*-13a).** The general procedure for nucleophilic substitution reactions of thioacetals was followed using thioacetal **6a** (0.0600 g, 0.200 mmol), 1,1,3,3-hexafluoroisopropanol (0.126 mL, 1.20 mmol), and *N*-iodosuccinimide (0.0900 g, 0.400 mmol) in CH_2Cl_2 (2 mL). $^{13}C\{^1H\}$ NMR spectroscopic analysis of the unpurified reaction mixture revealed that acetal *trans*-**13a** was formed as a single diastereomer.[1] Purification by flash

column chromatography (10:90 EtOAc:hexanes) afforded acetal *trans*-**13a** as a yellow oil (0.0150 g, 21%).

Note: Purification by column chromatography afforded acetal *trans*-**13a** as a mixture with inseparable unidentified decomposition impurities that appeared after purification. Attempts to separate acetal *trans*-**13a** from the decomposition impurities failed after multiple columns.

Acetal *trans*-**13a**: ^{1}H NMR (400 MHz, $CDCl_3$) δ 8.07–7.99 (m, 2H), 7.62–7.56 (m, 1H), 7.48–7.42 (m, 2H), 5.51 (dd, J = 5.8, 1.1 Hz, 1H), 5.43 (br s, 1H), 4.62–4.54 (m, 1H), 4.27 (dt, J = 16.3, 8.0 Hz, 1H), 4.16 (td, J = 9.1, 4.3 Hz, 1H), 2.56–2.45 (m, 1H), 2.22–2.12 (m, 1H);

^{13}C{^{1}H} NMR (100 MHz, $CDCl_3$, characteristic peaks) δ 165.6 (C), 136.6 (CH), 129.9 (CH), 128.6 (CH), 105.9 (CH), 77.7 (CH), 67.0 (br m, $^2J_{C\text{–}F}$ = 28.6 Hz, CH), 68.5 (CH_2), 29.3 (CH_2);

IR (ATR) 2963, 1724, 1191, 1102, 934, 710 cm^{-1};

HRMS (TOF MS ES$^+$) m / z calcd for $C_{14}H_{12}F_6NaO_4$ (M + Na)$^+$ 381.0532, found 381.0497.

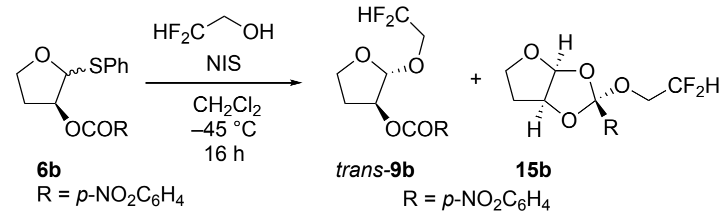

(2*R,3*S**)-2-(2,2-difluoroethoxy)tetrahydrofuran-3-yl 4-nitrobenzoate (*trans*-9b) and (2*R**,3a*R**,6a*S**)-2-(2,2-difluoroethoxy)-2-(4-nitrophenyl)tetrahydrofuro[2,3-*d*][1,3]dioxole (15b).** The general procedure for nucleophilic substitution reactions of thioacetals was followed using thioacetal **6b** (0.0500 g, 0.149 mmol), 2,2-difluoroethanol (0.0551 mL, 0.870 mmol), and *N*-iodosuccinimide (0.0650 g, 0.290 mmol) in CH_2Cl_2 (1.5 mL). ^{13}C{^{1}H} NMR spectroscopic analysis of the unpurified reaction mixture revealed that acetal **9b** was formed as a single diastereomer.[1] Purification by flash column chromatography (15:85 EtOAc:hexanes) afforded acetal *trans*-**9b** as a yellow oil (0.0140 g, 30%) and orthoester **15b** as a yellow oil (0.0120 g, 26%).

Major diastereomer *trans*-**9b**: ^{1}H NMR (400 MHz, $CDCl_3$) δ 8.33–8.28 (m, 2H), 8.22–8.17 (m, 2H), 5.92 (tt, J = 55.2, 4.3 Hz, 1H), 5.42 (dd, J = 6.5, 1.6 Hz, 1H), 5.23 (br s, 1H), 4.21 (q, J = 7.8 Hz, 1H), 4.10 (td, J = 8.6, 4.9 Hz, 1H), 3.94–3.66 (m, 2H), 2.60–2.47 (m, 1H), 2.17–2.06 (m, 1H);

$^{13}C\{^1H\}$ NMR (100 MHz, $CDCl_3$) δ 164.1 (C), 150.9 (C), 135.1 (C), 131.0 (CH), 123.8 (CH), 114.3 (br t, $^1J_{C–F}$ = 240.7 Hz, CH), 105.9 (CH), 79.1 (CH), 67.1 (CH_2), 66.3 (br t, $^2J_{C–F}$ = 28.3 Hz, CH_2), 29.8 (CH_2);

IR (ATR) 2996, 1726, 1525, 1269, 1054, 719 cm^{-1};

HRMS (TOF MS ES$^+$) *m* / *z* calcd for $C_{13}H_{17}F_2N_2O_6$ (M + H)$^+$ 318.0784, found 318.0801.

Orthoester **15b**: ^1H NMR (400 MHz, $CDCl_3$) δ 8.27–8.22 (m, 2H), 7.84–7.79 (m, 2H), 6.13 (d, *J* = 3.9 Hz, 1H), 3.87 (tt, *J* = 55.8, 4.1 Hz, 1H), 5.07 (t, *J* = 4.3 Hz, 1H), 3.93 (t, *J* = 8.6 Hz, 1H), 3.68–3.57 (m, 2H), 3.59–3.45 (m, 1H), 2.14–2.08 (m, 1H), 2.00–1.88 (m, 1H);

$^{13}C\{^1H\}$ NMR (100 MHz, $CDCl_3$) δ 148.8 (C), 143.1 (C), 127.4 (CH), 123.8 (CH), 121.6 (C), 114.0 (br t, $^1J_{C–F}$ = 239.4 Hz, CH), 106.4 (CH), 82.1 (CH), 67.3 (CH_2), 62.7 (br t, $^2J_{C–F}$ = 29.9 Hz, CH_2), 33.1 (CH_2);

IR (ATR) 2980, 1516, 1269, 1113, 1025, 709 cm^{-1};

HRMS (TOF MS ES$^+$) *m* / *z* calcd for $C_{13}H_{14}F_2NO_6$ (M + Na – H_2O)$^+$ 322.0497, found 322.0513.

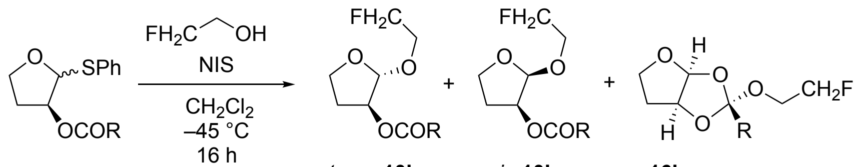

6b R = *p*-$NO_2C_6H_4$ *trans*-**10b** *cis*-**10b** R = *p*-$NO_2C_6H_4$ **16b**

(2*R,3*S**)-2-(2-Fluoroethoxy)tetrahydrofuran-3-yl 4-nitrobenzoate (*trans*-10b), (2*R**,3*R**)-2-(2-fluoroethoxy)tetrahydrofuran-3-yl 4-nitrobenzoate (*cis*-10b), and (2*R**,3a*R**,6a*S**)-2-(2-fluoroethoxy)-2-(4-nitrophenyl)tetrahydrofuro[2,3-*d*][1,3]dioxole (16b).** The general procedure for nucleophilic substitution reactions of thioacetals was followed using thioacetal **6b** (0.100 g, 0.290 mmol), 2-fluoroethanol (0.102 mL, 1.74 mmol), and *N*-iodosuccinimide (0.130 g, 0.290 mmol) in CH_2Cl_2 (3 mL). $^{13}C\{^1H\}$ NMR spectroscopic analysis of the unpurified reaction mixture revealed that acetal **10b** was formed as a mixture of diastereomers (*trans*-**10b**:*cis*-**10b** = 75:25).[1] Purification by flash column chromatography (10:90 EtOAc:hexanes) afforded acetal *trans*-**10b** as a yellow oil (0.0210 g, 25%), acetal *cis*-**10b** as a yellow oil (0.0090 g, 10%), and orthoester **16b** as a yellow oil (0.0190 g, 22%).

Major diastereomer *trans*-**10b**: ^1H NMR (400 MHz, $CDCl_3$) δ 8.30–8.26 (m, 2H), 8.21–8.17 (m, 2H), 5.41 (dt, *J* = 3.9, 1.7 Hz, 1H), 5.22 (br s, 1H), 4.63 (td, *J* = 3.6, 1.5 Hz, 1H), 4.51 (td, *J* = 3.8, 1.4 Hz, 1H), 4.21–4.14 (m, 1H), 4.12–4.05 (m, 1H), 3.98–3.84 (m, 2H), 2.59–2.48 (m, 1H), 2.13–2.02 (m, 1H);

$^{13}C\{^1H\}$ NMR (100 MHz, $CDCl_3$) δ 164.1 (C), 150.9 (C), 135.2 (C), 131.0 (CH), 123.7 (CH), 105.6 (CH), 82.9 (br d, $^1J_{C–F}$ = 170.3 Hz, CH_2), 79.3 (CH), 66.8 (CH_2), 66.3 (br d, $^2J_{C–F}$ = 19.8 Hz, CH_2), 30.0 (CH_2);

IR (ATR) 2958, 1727, 1529, 1266, 1104, 733 cm^{-1};

HRMS (TOF MS ES$^+$) *m* / *z* calcd for $C_{13}H_{14}FNNaO_6$ (M + Na)$^+$ 322.0697, found 322.0687.

Minor diastereomer *cis*-**10b**: ^1H NMR (400 MHz, $CDCl_3$) δ 8.32–8.26 (m, 2H), 8.25–8.21 (m, 2H), 5.31 (d, *J* = 4.3 Hz, 1H), 5.21 (td, *J* = 8.4, 4.3 Hz, 1H), 4.56–4.48 (m, 1H), 4.44–4.39 (m, 1H), 4.17–4.08 (m, 1H), 4.04–3.93 (m, 1H), 3.91–3.85 (m, 1H), 3.79–3.65 (m, 1H), 2.49–2.39 (m, 1H), 2.35–2.23 (m, 1H);

$^{13}C\{^1H\}$ NMR (100 MHz, $CDCl_3$) δ 164.7 (C), 150.8 (C), 135.4 (C), 131.0 (CH), 123.7 (CH), 100.3 (CH), 82.8 (br d, $^1J_{C–F}$ = 169.2 Hz, CH_2), 74.8 (CH), 67.2 (br d, $^2J_{C–F}$ = 20.3 Hz, CH_2), 64.6 (CH_2), 27.8 (CH_2);

IR (ATR) 2959, 1726, 1527, 1268, 1028, 733 cm^{-1};

HRMS (TOF MS ES$^+$) *m* / *z* calcd for $C_{13}H_{14}FNNaO_6$ (M + Na)$^+$ 323.073, found 323.0732.

Orthoester **16b**: δ 8.26–8.21 (m, 2H), 7.86–7.80 (m, 2H), 6.12 (d, *J* = 4.0 Hz, 1H), 5.06 (t, *J* = 4.6 Hz, 1H), 4.60 (t, *J* = 4.4 Hz, 1H), 4.48 (t, *J* = 4.6 Hz, 1H), 3.93 (t, *J* = 8.3 Hz, 1H), 3.75–3.67 (m, 1H), 3.65–3.60 (m, 1H), 3.54 (ddd, *J* = 16.5, 8.8, 5.4 Hz, 1H), 2.11 (dd, *J* = 14.2, 5.0 Hz, 1H), 1.98–1.87 (m, 1H);

$^{13}C\{^1H\}$ NMR (100 MHz, $CDCl_3$) δ 148.6 (C), 143.7 (C), 127.4 (CH), 123.7 (CH), 121.5 (C), 106.3 (CH), 82.1 (br d, $^1J_{C–F}$ = 168.9 Hz, CH_2), 81.9 (CH), 67.2 (CH_2), 62.5 (br d, $^2J_{C–F}$ = 20.9 Hz, CH_2), 33.2 (CH_2);

IR (ATR) 2960, 1525, 1268, 1048, 974, 733 cm^{-1};

HRMS (TOF MS ES$^+$) *m* / *z* calcd for $C_{13}H_{14}FNNaO_6$ (M + Na)$^+$ 322.0697, found 322.0709.

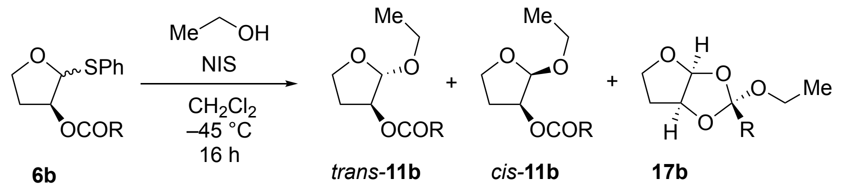

6b R = *p*-$NO_2C_6H_4$ *trans*-**11b** *cis*-**11b** R = *p*-$NO_2C_6H_4$ **17b**

(2*R,3*S**)-2-Ethoxytetrahydrofuran-3-yl 4-nitrobenzoate (*trans*-11b), (2*R**,3*R**)-2-ethoxytetrahydrofuran-3-yl 4-nitrobenzoate (*cis*-11b) and (2*R**,3a*R**,6a*S**)-2-ethoxy-2-(4-nitrophenyl)tetrahydrofuro[2,3-*d*][1,3]dioxole (17b).** The general procedure for nucleophilic substitution reactions of thioacetals was followed using thioacetal **6b** (0.0550 g, 0.159 mmol), ethanol (0.100 mL, 1.59 mmol), and *N*-iodosuccinimide (0.215 g, 0.955 mmol) in CH_2Cl_2 (1.6 mL). $^{13}C\{^1H\}$ NMR spectroscopic analysis of the unpurified reaction mixture revealed that acetal **11b** was formed as a mixture of

diastereomers (*trans*-**11b**:*cis*-**11b**= 80:20).[1] Purification by flash column chromatography (10:90 EtOAc:hexanes) afforded acetal **11b** as a yellow oil (0.0210 g, 47%) as a mixture of diastereomers (*trans*-**11b**:*cis*-**11b** = 40:60) and orthoester **17b** as a yellow oil (0.0120 g, 27%).

Major diastereomer *trans*-**11b** and minor diastereomer *cis*-**11b** (*trans*-**16**:*cis*-**16** = 40:60): ^{1}H NMR (400 MHz, $CDCl_3$) δ 8.33–8.26 (m, 3.6H), 8.26–8.17 (m, 3.6H), 5.36 (dd, J = 6.2, 1.5 Hz, 0.8H), 5.27 (d, J = 4.3 Hz, 1H), 5.19 (td, J = 8.1, 4.4 Hz, 1H), 5.17 (br s, 0.8H), 4.19–4.04 (m, 2.8H), 3.94 (dt, J = 10.4, 6.5 Hz, 1H), 3.81–3.70 (m, 1.8H), 3.59–3.43 (m, 1.8H), 2.57–2.50 (m, 0.8H), 2.45–2.35 (m, 1H), 2.32–2.20 (m, 1H), 2.16–2.01 (m, 0.8H), 1.22 (t, J = 7.2 Hz, 2.4H), 1.22 (t, J = 7.2 Hz, 3H);

Peaks attributed to isomer *trans*-**11b**: ^{13}C{^{1}H} NMR (100 MHz, $CDCl_3$) δ 164.2 (C), 150.81 (C), 135.3 (C), 130.9 (CH), 123.72 (CH), 105.4 (CH), 79.5 (CH), 66.5 (CH_2), 63.0 (CH_2), 30.1 (CH_2), 15.2 (CH_3);

Peaks attributed to isomer *cis*-**11b**: ^{13}C{^{1}H} NMR (100 MHz, $CDCl_3$) δ 164.6 (C), 150.80 (C), 135.5 (C), 131.0 (CH), 123.70 (CH), 100.0 (CH), 74.9 (CH), 64.3 (CH_2), 63.9 (CH_2), 28.1 (CH_2), 15.3 (CH_3);

IR (ATR) 2977, 1725, 1526, 1269, 1100, 592 cm^{-1};

HRMS (TOF MS ES$^+$) *m* / *z* calcd for $C_{13}H_{15}NNaO_6$ (M + Na)$^+$ 304.0792, found 304.0779.

Orthoester **17b**: ^{1}H NMR (400 MHz, $CDCl_3$) δ 8.25–8.19 (m, 2H), 7.85–7.79 (m, 2H), 6.11 (d, J = 3.7 Hz, 1H), 5.03 (t, J = 4.9 Hz, 1H), 3.91 (t, J = 8.5 Hz, 1H), 3.55–3.44 (m, 3H), 2.15–2.04 (m, 1H), 1.96–1.87 (m, 1H), 1.21 (t, J = 7.2 Hz, 3H);

^{13}C{^{1}H} NMR (100 MHz, $CDCl_3$) δ 148.5 (C), 144.7 (C), 127.3 (CH), 123.6 (CH), 121.8 (C), 106.2 (CH), 81.9 (CH), 67.1 (CH_2), 58.8 (CH_2), 33.3 (CH_2), 15.1 (CH_3);

IR (ATR) 2980, 2361, 1727, 1275, 1106, 976 cm^{-1};

HRMS (TOF MS ES$^+$) *m* / *z* calcd for $C_{13}H_{15}NNaO_6$ (M + Na)$^+$ 304.0792, found 304.0799.

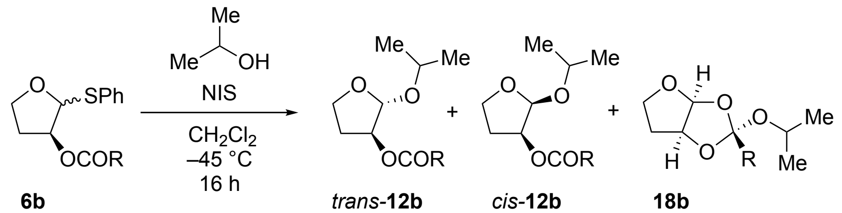

6b R = *p*-$NO_2C_6H_4$ *trans*-**12b** *cis*-**12b** **18b** R = *p*-$NO_2C_6H_4$

(2*R,3*S**)-2-Isopropoxytetrahydrofuran-3-yl 4-nitrobenzoate (*trans*-12b), (2*R**,3*R**)-2-isopropoxytetrahydrofuran-3-yl 4-nitrobenzoate (*cis*-12b) and (2*R**,3a*R**,6a*S**)-2-isopropoxy-2-(4-nitrophenyl)tetrahydrofuro[2,3-*d*][1,3]dioxole (18b).** The general procedure for nucleophilic substitution

reactions of thioacetals was followed using thioacetal **6b** (0.345 g, 1.00 mmol), isopropyl alcohol (0.763 mL, 6.00 mmol), and *N*-iodosuccinimide (0.450 g, 2.00 mmol) in CH_2Cl_2 (10 mL). $^{13}C\{^1H\}$ NMR spectroscopic analysis of the unpurified reaction mixture revealed that acetal **12b** was formed as a mixture of diastereomers (*trans*-**12b**:*cis*-**12b** = 80:20).[1] Purification by flash column chromatography (10:90 EtOAc:hexanes) afforded acetal *trans*-**12b** as a yellow oil (0.0950 g, 32%), acetal *cis*-**12b** as a yellow oil (0.0200 g, 8%), and orthoester **18b** as a yellow oil (0.144 g, 49%).

Major diastereomer *trans*-**12b**: ^1H NMR (400 MHz, $CDCl_3$) δ 8.29 (d, J = 8.8 Hz, 2H), 8.20 (d, J = 8.5 Hz, 2H), 5.32 (dd, J = 6.2, 1.2 Hz, 1H), 5.26 (br s, 1H), 4.18–4.04 (m, 2H), 3.99–3.88 (m, 1H), 2.59–2.44 (m, 1H), 2.12–2.60 (m, 1H), 1.20 (t, J = 6.4 Hz, 6H);

$^{13}C\{^1H\}$ NMR (100 MHz, $CDCl_3$) δ 164.2 (C), 150.8 (C), 135.4 (C), 131.0 (CH), 123.7 (CH), 103.7 (CH), 79.8 (CH), 69.2 (CH_2), 66.2 (CH_2), 30.1 (CH_2), 23.5 (CH_3), 21.8 (CH_3);

IR (ATR) 3285, 1724, 1528, 1269, 1034, 719 cm^{-1};

HRMS (TOF MS ES^+) *m* / *z* calcd for $C_{14}H_{17}NNaO_6$ $(M + Na)^+$ 318.0948, found 318.0947.

Minor diastereomer *cis*-**12b**: ^1H NMR (400 MHz, $CDCl_3$) δ 8.33–8.27 (m, 2H), 8.25–8.19 (m, 2H), 5.36 (d, J = 4.5 Hz, 1H), 5.16 (ddd, J = 11.9, 7.5, 4.2 Hz, 1H), 4.15–4.07 (m, 1H), 3.95–3.89 (m, 1H), 3.87–3.81 (m, 1H), 2.44–2.33 (m, 1H), 2.32–2.20 (m, 1H), 1.18 (d, J = 6.3 Hz, 3H), 1.00 (d, J = 6.1 Hz, 3H);

$^{13}C\{^1H\}$ NMR (100 MHz, $CDCl_3$) δ 164.7 (C), 150.7 (C), 135.7 (C), 130.9 (CH), 123.7 (CH), 99.1 (CH), 74.9 (CH), 71.1 (CH_2), 64.0 (CH_2), 28.1 (CH_2), 23.7 (CH_3), 22.2 (CH_3);

IR (ATR) 2980, 1727, 1527, 1273, 1031, 720 cm^{-1};

HRMS (TOF MS ES^+) *m* / *z* calcd for $C_{14}H_{17}FNO_6$ $(M + K - H_2O)^+$ 316.0582, found 316.0577.

Orthoester **18b**: ^1H NMR (400 MHz, $CDCl_3$) δ 8.24–8.18 (m, 2H), 7.86–7.77 (m, 2H), 6.09 (d, J = 4.1 Hz, 1H), 5.02 (t, J = 4.5 Hz, 1H), 3.89 (t, J = 8.5 Hz, 1H), 3.84–3.76 (m, 1H), 3.50–3.40 (m, 1H), 2.07 (dd, J = 13.5, 4.4 Hz, 1H), 1.96–1.84 (m, 1H), 1.18 (d, J = 6.3 Hz, 3H), 1.14 (d, J = 6.3 Hz, 3H);

$^{13}C\{^1H\}$ NMR (100 MHz, $CDCl_3$) δ 148.5 (C), 144.7 (C), 127.3 (CH), 123.6 (CH), 121.8 (C), 106.2 (CH), 81.9 (CH), 67.2 (CH_2), 58.8 (CH_2), 33.3 (CH_2), 15.1 (CH_3);

IR (ATR) 2977, 1523, 1271, 1119, 1050, 735 cm^{-1};

HRMS (TOF MS ES^+) *m* / *z* calcd for $C_{14}H_{15}KNO_5$ $(M + K - H_2O)^+$ 316.0582, found 316.0577.

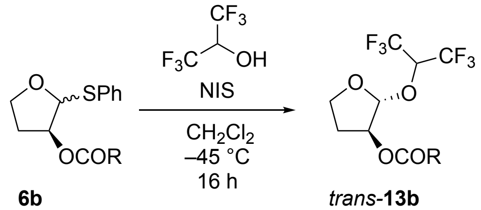

6b
R = *p*-$NO_2C_6H_4$

trans-**13b**
R = *p*-$NO_2C_6H_4$

(2*R**,3*S****)-2-((1,1,1,3,3,3-Hexafluoropropan-2-yl)oxy)tetrahydrofuran-3-yl 4-nitrobenzoate (*trans*-13b).** The general procedure for nucleophilic substitution reactions of thioacetals was followed using thioacetal **6b** (0.0700 g, 0.203 mmol), 1,1,3,3-hexafluoroisopopanol (0.128 mL, 1.22 mmol), and *N*-iodosuccinimide (0.0911 g, 0.405 mmol) in CH_2Cl_2 (2 mL). $^{13}C\{^1H\}$ NMR spectroscopic analysis of the unpurified reaction mixture revealed that acetal *trans*-**13b** was formed as a single diastereomer.[1] Purification by flash column chromatography (5:95 EtOAc:hexanes) afforded acetal *trans*-**13b** as a yellow oil (0.0183 g, 22%):

^1H NMR (400 MHz, $CDCl_3$) δ 8.34–8.27 (m, 2H), 8.23–8.18 (m, 2H), 5.54 (dd, J = 5.8, 1.2 Hz, 1H), 5.45 (br s, 1H), 4.61–4.53 (m, 1H), 4.28 (dt, J = 8.2, 8.0 Hz, 1H), 4.18 (td, J = 9.6, 4.2 Hz, 1H), 2.62–2.50 (m, 1H), 2.25–2.15 (m, 1H);

$^{13}C\{^1H\}$ NMR (100 MHz, $CDCl_3$, characteristic peaks) δ 163.7 (C), 150.9 (C), 134.6 (C), 130.9 (CH), 123.7 (CH), 105.5 (CH), 78.5 (CH), 69.8 (br q, $^2J_{C–F}$ = 33.8 Hz, CH), 68.3 (CH_2), 29.1 (CH_2);

IR (ATR) 2917, 1730, 1529, 1264, 1100, 717 cm^{-1};

HRMS (TOF MS ES^+) m / z calcd for $C_{14}H_{13}F_6N_2O_5$ (M + NH_4 – H_2O)$^+$ 403.0723, found 403.0733.

IV. Nucleophilic Substitution Reactions of Furanosyl Acetals with *C*-nucleophiles

General procedure for nucleophilic substitution reactions of acetals with carbon nucleophiles. To a solution of acetate (1.0 equiv) in solvent (0.1 M) at –45 °C was added nucleophile (2.5 equiv unless otherwise noted), followed by the addition of Lewis acid (2.5 equiv unless otherwise noted). The reaction mixture was then stirred at –45 °C for 16 h. A solution of MeOH:CH_2Cl_2:Et_3N (1:1:1, 1 mL per mmol of acetate) was added at –45 °C and the reaction mixture was warmed to room temperature. The reaction mixture was then diluted with CH_2Cl_2 (1 × 20 mL per mmol of acetate) and washed with saturated aqueous Rochelle's salt solution (1 × 20 mL per mmol of acetate). The aqueous layer was extracted with CH_2Cl_2 (2 × 20 mL per mmol of acetate). The combined organic layers were dried over Na_2SO_4, filtered,

and concentrated *in vacuo*. The diastereomeric ratios were determined by $^{13}C\{^1H\}$ NMR analysis of the unpurified reaction mixture.[1] The reaction mixture was purified by flash column chromatography to provide products. The relative stereochemical configurations of products were assigned by analysis of coupling constants. Details of stereochemical proofs are provided in section VII.

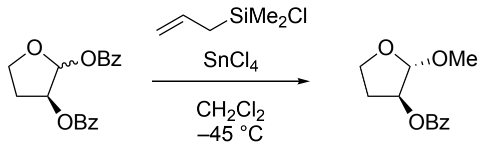

(2*R,3*S**)-2-Methoxytetrahydrofuran-3-yl benzoate (*trans*-S3).** The general procedure for nucleophilic substitution reactions of acetates was followed using dibenzoate **21a** (0.0300 g, 0.0961 mmol), allylchlorodimethylsilane (0.0534 mL, 0.481 mmol), and $SnCl_4$ (0.240 mL, 0.24 mmol, 1.0 M in CH_2Cl_2) in CH_2Cl_2 (0.72 mL). $^{13}C\{^1H\}$ NMR spectroscopic analysis of the unpurified reaction mixture revealed that benzoate *trans*-**S3** was formed as a single diastereomer.[1] Purification by flash column chromatography (20:80 EtOAc:hexanes) afforded benzoate *trans*-**S3** as a yellow oil (0.0150 g, 70%):

^1H NMR (400 MHz, $CDCl_3$) δ 8.07–8.00 (m, 2H), 7.63–7.54 (m, 1H), 7.49–7.41 (m, 2H), 5.33 (dd, J = 6.5, 1.2 Hz, 1H), 5.08 (br s, 1H), 4.17 (dt, J = 8.1, 7.7 Hz, 1H), 4.07 (ddd, J = 13.8, 8.6, 5.0 Hz, 1H), 3.40 (s, 3H), 2.52–2.40 (m, 1H), 2.13–2.01 (m, 1H);

$^{13}C\{^1H\}$ NMR (100 MHz, $CDCl_3$) δ 166.0 (C), 133.4 (CH), 130.0 (C), 129.8 (CH), 128.5 (CH), 106.9 (CH), 78.3 (CH), 66.7 (CH_2), 54.7 (CH_3), 30.1 (CH_2).

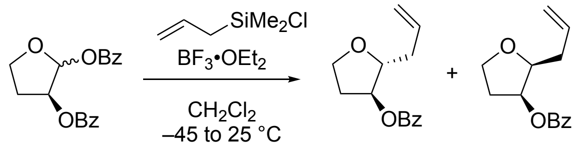

(2*R,3*S**)-2-Allyltetrahydrofuran-3-yl benzoate (*trans*-22a) and (2*R**,3*R**)-2-allyltetrahydrofuran-3-yl benzoate (*cis*-22a).** To a solution of dibenzoate **21a** (0.0310 g, 0.100 mmol) in CH_2Cl_2 (1 mL) at –45 °C was added allylchlorodimethylsilane (0.110 mL, 1.00 mmol) followed by the addition of BF_3•OEt_2 (0.0620 mL, 0.500 mmol). After 1 h, the reaction mixture was warmed to 25 °C. After 16 h, a solution of saturated aqueous $NaHCO_3$ (5 mL) was added and the layers were separated. The aqueous layer was extracted with CH_2Cl_2 (2 × 10 mL). The combined organic layers were dried over Na_2SO_4, filtered, and concentrated *in vacuo*. $^{13}C\{^1H\}$ NMR spectroscopic analysis of the unpurified reaction mixture revealed that benzoate **5**

was formed as a mixture of diastereomers (*trans*-**22a**:*cis*-**22a** = 70:30).[1] The spectral data obtained are consistent with those of products from nucleophilic substitution reactions of dibenzoate **21a** and allyltrimethylsilane in the presence of $SnCl_4$.

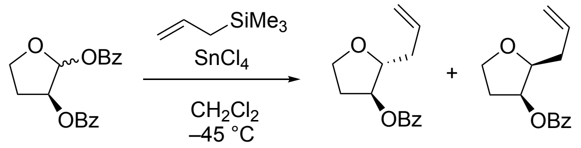

21a *trans*-**22a** *cis*-**22a**

(2*R**,3*S****)-2-Allyltetrahydrofuran-3-yl benzoate (*trans*-22a) and (2*R****,3*R****)-2-allyltetrahydrofuran-3-yl benzoate (*cis*-22a).** The general procedure for nucleophilic substitution reactions of acetates was followed using dibenzoate **21a** (0.100 g, 0.320 mmol), allyltrimethylsilane (0.254 mL, 1.60 mmol), and $SnCl_4$ (0.800 mL, 0.80 mmol, 1.0 M in CH_2Cl_2) in CH_2Cl_2 (2.4 mL). $^{13}C\{^1H\}$ NMR spectroscopic analysis of the unpurified reaction mixture revealed that benzoate **22a** was formed as a mixture of diastereomers (*trans*-**22a**:*cis*-**22a** = 55:45).[1] Purification by flash column chromatography (20:80 EtOAc:hexanes) afforded benzoate *trans*-**22a** as a yellow oil (0.0311 g, 42%) and *cis*-**22a** as a yellow oil (0.0231 g, 31%).

Note: Purification by column chromatography afforded acetal *cis*-**22a** as a mixture with an inseparable decomposition product that appeared after purification, which was identified to be dibenzoate *trans*-**21a** (*cis*-**22a**:*trans*-**21a** = 67:33).

Major diastereomer *trans*-**22a**: ^1H NMR (400 MHz, $CDCl_3$) δ 8.05–8.01 (m, 2H), 7.60–7.54 (m, 1H), 7.48–7.41 (m, 2H), 5.92–5.81 (m, 1H), 5.23 (dt, J = 6.5, 2.2 Hz, 1H), 5.17 (dq, J = 17.1, 1.6 Hz, 1H), 5.14–5.09 (m, 1H), 4.12–4.04 (m, 2H), 4.00–3.92 (m, 1H), 2.50–2.35 (m, 2H), 2.33–2.23 (m, 1H), 2.13–2.05 (m, 1H); $^{13}C\{^1H\}$ NMR (100 MHz, $CDCl_3$) δ 166.3 (C), 134.0 (CH), 133.3 (CH), 130.2 (C), 129.8 (CH), 128.5 (CH), 117.8 (CH_2), 83.5 (CH), 78.5 (CH), 67.2 (CH_2), 38.1 (CH_2), 32.7 (CH_2);
IR (ATR) 2981, 1716, 1269, 1069, 915, 710 cm^{-1};
HRMS (TOF MS ES^+) m / z calcd for $C_{14}H_{16}NaO_3$ $(M + Na)^+$ 255.0992, found 255.0997.
Minor diastereomer *cis*-**22a**: ^1H NMR (400 MHz, $CDCl_3$, characteristic peaks) δ 8.07–8.03 (m, 2H), 7.60–7.55 (m, 1H), 7.48–7.44 (m, 2H), 5.91–5.78 (m, 1H), 5.55 (ddd, J = 7.8, 3.9, 1.8 Hz, 1H), 5.14–5.02 (m,

2H), 4.12 (q, J = 7.8 Hz, 1H), 3.93 (ddd, J = 9.4, 6.7, 3.8 Hz, 1H), 3.87 (td, J = 8.7, 5.3 Hz, 1H), 2.53–2.37 (m, 3H), 2.17–2.09 (m, 1H);

$^{13}C\{^1H\}$ NMR (100 MHz, $CDCl_3$, characteristic peaks) δ 166.1 (C), 134.5 (CH), 133.3 (CH), 129.81 (C), 129.78 (CH), 128.6 (CH), 117.4 (CH_2), 81.3 (CH), 75.1 (CH), 66.3 (CH_2), 34.0 (CH_2), 33.8 (CH_2);

IR (ATR) 2920, 1728, 1238, 1056, 801, 695 cm^{-1};

HRMS (TOF MS ES^+) m / z calcd for $C_{14}H_{14}NaO_2$ (M + Na – H_2O)$^+$ 237.0886, found 237.0891.

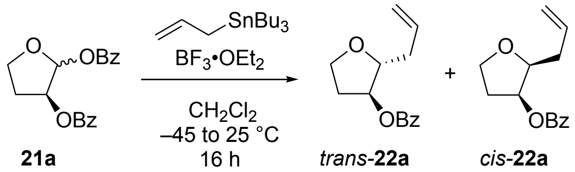

(2*R,3*S**)-2-Allyltetrahydrofuran-3-yl benzoate (*trans*-22a) and (2*R**,3*R**)-2-allyltetrahydrofuran-3-yl benzoate (*cis*-22a).** To a solution of dibenzoate **21a** (0.150 g, 0.480 mmol) in CH_2Cl_2 (5 mL) at –45 °C was added allyltributylstannane (0.595 mL, 1.92 mmol) followed by the addition of BF_3•OEt_2 (0.118 mL, 0.961 mmol). After 1 h, the reaction mixture was warmed to 25 °C. After 16 h, a solution of saturated aqueous $NaHCO_3$ (5 mL) was added and the layers were separated. The aqueous layer was extracted with CH_2Cl_2 (2 × 10 mL). The combined organic layers were dried over Na_2SO_4, filtered, and concentrated *in vacuo*. $^{13}C\{^1H\}$ NMR spectroscopic analysis of the unpurified reaction mixture revealed that benzoate **22a** was formed as a mixture of diastereomers (*trans*-**22a**:*cis*-**22a** = 87:13).[1] Purification by flash column chromatography (20:80 EtOAc:hexanes) afforded benzoate **22a** as a colorless oil (0.0630 g, 57%) as a mixture of diastereomers (*trans*-**22a**:*cis*-**22a** = 87:13). The spectral data obtained are consistent with those of products from nucleophilic substitution reactions of dibenzoate **21a** and allyltrimethylsilane in the presence of $SnCl_4$.

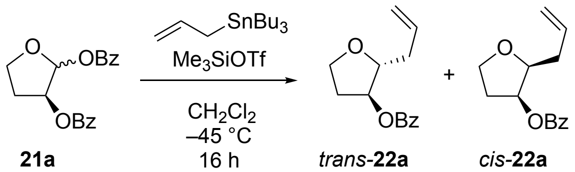

(2*R,3*S**)-2-Allyltetrahydrofuran-3-yl benzoate (*trans*-22a) and (2*R**,3*R**)-2-allyltetrahydrofuran-3-yl benzoate (*cis*-22a) formed using Me_3SiOTf as the Lewis acid.** The general procedure for nucleophilic substitution reactions of acetates was followed using dibenzoate **21a** (0.100 g, 0.320 mmol), allyltributylstannane (0.397 mL, 1.28 mmol), and Me_3SiOTf (0.397 mL, 1.28 mmol) in CH_2Cl_2 (3 mL). $^{13}C\{^1H\}$

NMR spectroscopic analysis of the unpurified reaction mixture revealed that benzoate *trans*-**22a** was formed as a single diastereomer (*trans*-**22a**:*cis*-**22a** ≥ 97:3).[1] The spectral data obtained are consistent with those of products from nucleophilic substitution reactions of dibenzoate **21a** and allyltrimethylsilane in the presence of $SnCl_4$.

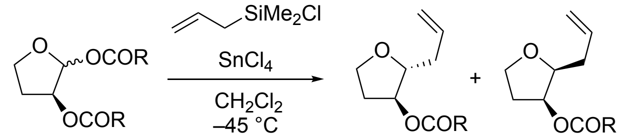

21b R = *p*-$NO_2C_6H_4$ *trans*-**22b** *cis*-**22b** R = *p*-$NO_2C_6H_4$

(2*R,3*S**)-2-Allyltetrahydrofuran-3-yl 4-nitrobenzoate (*trans*-22b) and (2*R**,3*R**)-2-allyltetrahydrofuran-3-yl 4-nitrobenzoate (*cis*-22).** The general procedure for nucleophilic substitution reactions of acetates was followed using *p*-nitrobenzoate **21b** (0.0300 g, 0.0746 mmol), allylchlorodimethylsilane (0.0413 mL, 0.372 mmol), and $SnCl_4$ (0.186 mL, 0.19 mmol, 1.0 M in CH_2Cl_2) in CH_2Cl_2 (0.5 mL). $^{13}C\{^1H\}$ NMR spectroscopic analysis of the unpurified reaction mixture revealed that *p*-nitrobenzoate **22b** was formed as a mixture of diastereomers (*trans*-**22b**:*cis*-**22b** = 61:39).[1] The spectral data obtained are consistent with those of products from nucleophilic substitution reactions of *p*-nitrobenzoate **22b** and allyltrimethylsilane in the presence of $SnCl_4$.

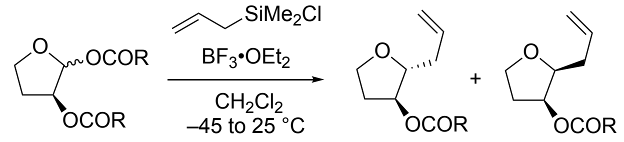

21b R = *p*-$NO_2C_6H_4$ *trans*-**22b** *cis*-**22b** R = *p*-$NO_2C_6H_4$

(2*R,3*S**)-2-Allyltetrahydrofuran-3-yl 4-nitrobenzoate (*trans*-22b) and (2*R**,3*R**)-2-allyltetrahydrofuran-3-yl 4-nitrobenzoate (*cis*-22b) formed using BF_3•OEt_2 as the Lewis acid.** To a solution of *p*-nitrobenzoate **21b** (0.0300 g, 0.0961 mmol) in CH_2Cl_2 (0.96 mL) at –45 °C was added allylchlorodimethylsilane (0.0540 mL, 0.481 mmol) followed by the addition of BF_3•OEt_2 (0.0593 mL, 0.481 mmol). After 1 h, the reaction mixture was warmed to 25 °C. After 16 h, a solution of saturated aqueous $NaHCO_3$ (5 mL) was added and the layers were separated. The aqueous layer was extracted with CH_2Cl_2 (2 × 10 mL). The combined organic layers were dried over Na_2SO_4, filtered, and concentrated *in vacuo*. $^{13}C\{^1H\}$ NMR spectroscopic analysis of the unpurified reaction mixture revealed that *p*-nitrobenzoate **22b** was formed as a mixture of diastereomers (*trans*-**22b**:*cis*-**22b** = 75:25).[1] The spectral data obtained are

consistent with those of products from nucleophilic substitution reactions of *p*-nitrobenzoate **21b** and allyltrimethylsilane in the presence of $SnCl_4$.

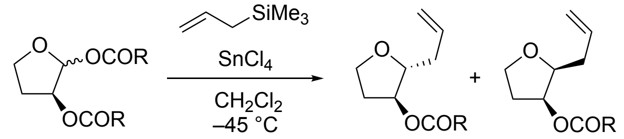

21b
R = *p*-$NO_2C_6H_4$

trans-**22b** *cis*-**22b**
R = *p*-$NO_2C_6H_4$

(2*R,3*S**)-2-Allyltetrahydrofuran-3-yl 4-nitrobenzoate (*trans*-22b) and (2*R**,3*R**)-2-allyltetrahydrofuran-3-yl 4-nitrobenzoate (*cis*-22b).** The general procedure for nucleophilic substitution reactions of acetates was followed using *p*-nitrobenzoate **21b** (0.0800 g, 0.199 mmol), allyltrimethylsilane (0.158 mL, 0.995 mmol), and $SnCl_4$ (0.500 mL, 0.50 mmol, 1.0 M in CH_2Cl_2) in CH_2Cl_2 (1.5 mL). $^{13}C\{^1H\}$ NMR spectroscopic analysis of the unpurified reaction mixture revealed that *p*-nitrobenzoate **22b** was formed as a mixture of diastereomers (*trans*-**22b**:*cis*-**22b** = 72:28).[1] Purification by flash column chromatography (20:80 EtOAc:hexanes) afforded *p*-nitrobenzoate *trans*-**22b** as a yellow oil (0.0273 g, 49%) and *cis*-**22b** as a yellow oil (0.0100 g, 18%).

Major diastereomer *trans*-**22b**: ^1H NMR (400 MHz, $CDCl_3$) δ 8.32–8.27 (m, 2H), 8.22–8.18 (m, 2H), 5.92–5.80 (m, 1H), 5.27 (dt, J = 6.5, 2.2 Hz, 1H), 5.18 (dq, J = 17.2, 1.7 Hz, 1H), 5.15–5.11 (m, 1H), 4.13–4.06 (m, 2H), 3.97 (dt, J = 9.9, 6.1 Hz, 1H), 2.47–2.38 (m, 2H), 2.28–2.26 (m, 1H), 2.15–2.08 (m, 1H);

$^{13}C\{^1H\}$ NMR (100 MHz, $CDCl_3$) δ 164.4 (C), 150.8 (C), 135.5 (C), 133.7 (CH), 130.9 (CH), 123.7 (CH), 118.1 (CH_2), 83.4 (CH), 79.6 (CH), 67.1 (CH_2), 37.9 (CH_2), 32.6 (CH_2);

IR (ATR) 2868, 1722, 1527, 1270, 1101, 748 cm^{-1};

HRMS (TOF MS ES^+) m / z calcd for $C_{14}H_{17}N_2O_4$ $(M + NH_4 – H_2O)^+$ 277.1183, found 277.1192.

Minor diastereomer *cis*-**22b**: ^1H NMR (400 MHz, $CDCl_3$) δ 8.33–8.28 (m, 2H), 8.25–8.21 (m, 2H), 5.90–5.77 (m, 1H), 5.58 (ddd, J = 7.7, 3.8, 1.7 Hz, 1H), 5.13–5.03 (m, 2H), 4.13 (q, J = 7.9 Hz, 1H), 3.93 (ddd, J = 7.7, 6.2, 3.7 Hz, 1H), 3.90–3.84 (m, 1H), 2.56–2.39 (m, 3H), 2.20–2.10 (m, 1H);

$^{13}C\{^1H\}$ NMR (100 MHz, $CDCl_3$) δ 164.2 (C), 150.8 (C), 135.5 (C), 134.2 (CH), 130.9 (CH), 123.8 (CH), 117.6 (CH_2), 81.2 (CH), 76.3 (CH), 66.3 (CH_2), 33.9 (CH_2), 33.7 (CH_2);

IR (ATR) 2927, 1723, 1527, 1271, 1102, 718 cm^{-1};

HRMS (TOF MS ES^+) m / z calcd for $C_{14}H_{16}NO_5$ $(M + H)^+$ 278.1023, found 278.1030.

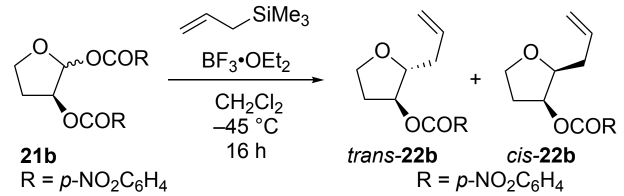

(2*R,3*S**)-2-Allyltetrahydrofuran-3-yl 4-nitrobenzoate (*trans*-22b) and (2*R**,3*R**)-2-allyltetrahydrofuran-3-yl 4-nitrobenzoate (*cis*-22b) formed using BF_3•OEt_2 as the Lewis acid.** To a solution of *p*-nitrobenzoate **21b** (0.200 g, 0.497 mmol) in CH_2Cl_2 (5 mL) at –45 °C was added allyltrimethylsilane (0.316 mL, 1.99 mmol) followed by the addition of BF_3•OEt_2 (0.123 mL, 0.994 mmol). After 1 h, the reaction mixture was warmed to 25 °C. After 16 h, a solution of saturated aqueous $NaHCO_3$ (5 mL) was added and the layers were separated. The aqueous layer was extracted with CH_2Cl_2 (2 × 10 mL). The combined organic layers were dried over Na_2SO_4, filtered, and concentrated *in vacuo*. $^{13}C\{^1H\}$ NMR spectroscopic analysis of the unpurified reaction mixture revealed that *p*-nitrobenzoate **21b** was formed as a mixture of diastereomers (*trans*-**22b**:*cis*-**22b** = 60:40).[1] The spectral data obtained are consistent with those of products from nucleophilic substitution reactions of *p*-nitrobenzoate **21b** and allyltrimethylsilane in the presence of $SnCl_4$.

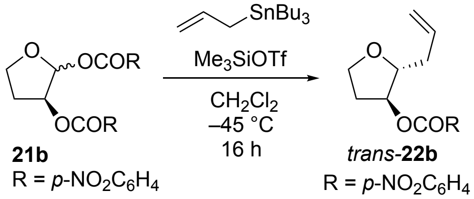

(2*R,3*S**)-2-Allyltetrahydrofuran-3-yl 4-nitrobenzoate (*trans*-22b) formed using Me_3SiOTf as the Lewis acid.** The general procedure for nucleophilic substitution reactions of acetates was followed using 4-nitrobenzoate **21b** (0.0900 g, 0.224 mmol), allyltributylstannane (0.278 mL, 0.896 mmol), and Me_3SiOTf (0.120 mL, 0.663 mmol) in CH_2Cl_2 (2 mL). $^{13}C\{^1H\}$ NMR spectroscopic analysis of the unpurified reaction mixture revealed that *p*-nitrobenzoate *trans*-**22b** was formed as a single diastereomer.[1] The spectral data obtained are consistent with those of products from nucleophilic substitution reactions of *p*-nitrobenzoate **21b** and allyltrimethylsilane in the presence of $SnCl_4$.

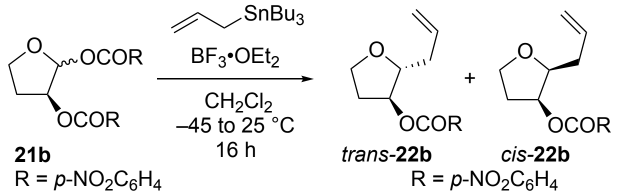

(2*R,3*S**)-2-Allyltetrahydrofuran-3-yl 4-nitrobenzoate (*trans*-22b) and (2*R**,3*R**)-2-allyltetrahydrofuran-3-yl 4-nitrobenzoate (*cis*-22b) formed using BF_3•OEt_2 as the Lewis acid.** To a solution of *p*-nitrobenzoate **21b** (0.200 g, 0.497 mmol) in CH_2Cl_2 (5 mL) at –45 °C was added allyltributylstannane (0.616 mL, 1.99 mmol) followed by the addition of BF_3•OEt_2 (0.123 mL, 0.994 mmol). After 1 h, the reaction mixture was warmed to 25 °C. After 16 h, a solution of saturated aqueous $NaHCO_3$ (5 mL) was added and the layers were separated. The aqueous layer was extracted with CH_2Cl_2 (2 × 10 mL). The combined organic layers were dried over Na_2SO_4, filtered, and concentrated *in vacuo*. $^{13}C\{^1H\}$ NMR spectroscopic analysis of the unpurified reaction mixture revealed that *p*-nitrobenzoate **22b** was formed as a mixture of diastereomers (*trans*-**22b**:*cis*-**22b** = 90:10).[1] The spectral data obtained are consistent with those of products from nucleophilic substitution reactions of *p*-nitrobenzoate **21b** and allyltrimethylsilane in the presence of $SnCl_4$.

V. Parameter Screen for Substitution Reactions with 2,2,2-Trifluoroethanol

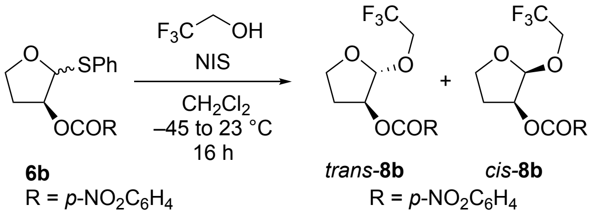

(2*R,3*S**)-2-(2,2,2-Trifluoroethoxy)tetrahydrofuran-3-yl 4-nitrobenzoate (*trans*-8b) and (2*R**,3*R**)-2-(2,2,2-trifluoroethoxy)tetrahydrofuran-3-yl 4-nitrobenzoate (*cis*-8b).** To a solution of thioacetal **6b** (0.0350 g, 0.100 mmol) in CH_2Cl_2 (1 mL) at –45 °C was added 2,2,2-trifluoroethanol (0.0437 mL, 0.600 mmol) followed by the addition of *N*-iodosuccinimide (0.0450 g, 0.200 mmol). After 1 h, the reaction mixture was warmed to 23 °C. After 16 h, a solution of saturated aqueous $Na_2S_2O_3$ (5 mL) was added and the layers were separated. The aqueous layer was extracted with CH_2Cl_2 (2 × 10 mL). The combined organic layers were dried over Na_2SO_4, filtered, and concentrated *in vacuo*. $^{13}C\{^1H\}$ NMR spectroscopic analysis of the unpurified reaction mixture revealed acetal **8b** was formed as a mixture of diastereomers (*trans*-**8b**:*cis*-

8b = 92:8) with no formation of side product orthoester **15b**.[1] The spectral data obtained are consistent with those of products from nucleophilic substitution reactions of thioacetal **6b** and 2,2,2-trifluoroethanol in the presence of *N*-iodosuccinimide at –45 °C.

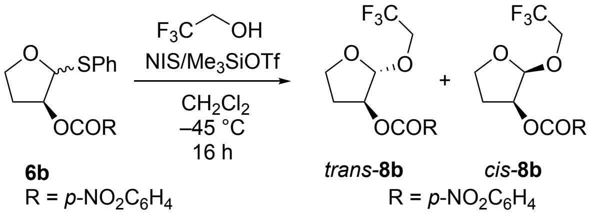

(2*R,3*S**)-2-(2,2,2-Trifluoroethoxy)tetrahydrofuran-3-yl 4-nitrobenzoate (*trans*-8b) and (2*R**,3*R**)-2-(2,2,2-trifluoroethoxy)tetrahydrofuran-3-yl 4-nitrobenzoate (*cis*-8b).** The general procedure for nucleophilic substitution reactions of thioacetals was followed using thioacetal **6b** (0.0350 g, 0.100 mmol), 2,2,2-trifluoroethanol (0.0437 mL, 0.600 mmol), *N*-iodosuccinimide (0.0450 g, 0.200 mmol) and Me_3SiOTf (0.0180 mL, 0.100 mmol) in CH_2Cl_2 (1 mL). $^{13}C\{^1H\}$ NMR spectroscopic analysis of the unpurified reaction mixture revealed that acetal **8b** was formed as a mixture of diastereomers (*trans*-**8b**:*cis*-**8b** = 95:5) with no formation of the corresponding orthoester side-product **15b**.[1] The spectral data obtained are consistent with those of products from nucleophilic substitution reactions of thioacetal **6b** and 2,2,2-trifluoroethanol in the presence of *N*-iodosuccinimide.

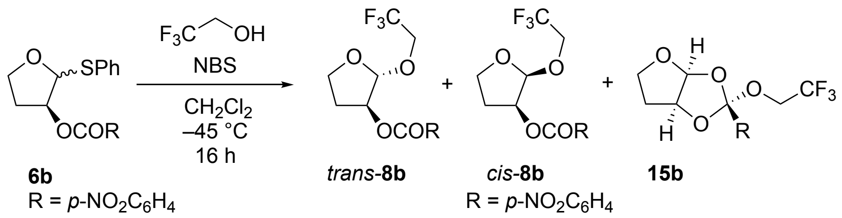

(2*R,3*S**)-2-(2,2,2-Trifluoroethoxy)tetrahydrofuran-3-yl 4-nitrobenzoate (*trans*-8b), (2*R**,3*R**)-2-(2,2,2-trifluoroethoxy)tetrahydrofuran-3-yl 4-nitrobenzoate (*cis*-8b) and (2*R**,3a*R**,6a*S**)-2-(4-nitrophenyl)-2-(2,2,2-trifluoroethoxy)hexahydrofuro[2,3-*b*]furan (15b).** The general procedure for nucleophilic substitution reactions of thioacetals was followed using thioacetal **6b** (0.0350 g, 0.100 mmol), 2,2,2-trifluoroethanol (0.0437 mL, 0.600 mmol), and *N*-bromosuccinimide (0.0356 g, 0.200 mmol) in CH_2Cl_2 (1 mL). $^{13}C\{^1H\}$ NMR spectroscopic analysis of the unpurified reaction mixture revealed that acetal **8b** was formed as a mixture of diastereomers (*trans*-**8b**:*cis*-**8b** = 65:35) with the formation of side-product orthoester **15b** (*trans*-**8b**:*cis*-**8b**: **15b** = 58:31:11).[1] The spectral data obtained are consistent with those of products

from nucleophilic substitution reactions of thioacetal **6b** and 2,2,2-trifluoroethanol in the presence of *N*-iodosuccinimide.

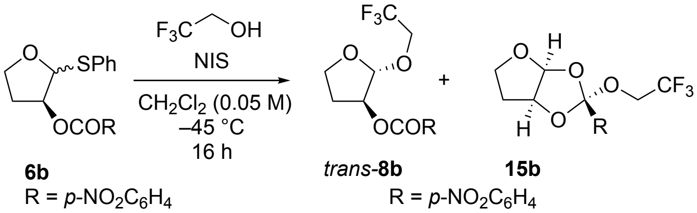

(2*R,3*S**)-2-(2,2,2-Trifluoroethoxy)tetrahydrofuran-3-yl 4-nitrobenzoate (*trans*-8b) and (2*R**,3a*R**,6a*S**)-2-(4-nitrophenyl)-2-(2,2,2-trifluoroethoxy)hexahydrofuro[2,3-*b*]furan (15b).** The general procedure for nucleophilic substitution reactions of thioacetals was followed using thioacetal **6b** (0.0350 g, 0.100 mmol), 2,2,2-trifluoroethanol (0.0437 mL, 0.600 mmol), and *N*-iodosuccinimide (0.0356 g, 0.200 mmol) in CH_2Cl_2 (2 mL). $^{13}C\{^1H\}$ NMR spectroscopic analysis of the unpurified reaction mixture revealed that acetal *trans*-**8b** was formed as a single diastereomer with the formation of the corresponding side-product orthoester **15b** (*trans*-**8b**:**15b** = 12:88).[1] The spectral data obtained are consistent with those of products from nucleophilic substitution reactions of thioacetal **6b** and 2,2,2-trifluoroethanol in the presence of *N*-iodosuccinimide at 0.1 M concentration in CH_2Cl_2.

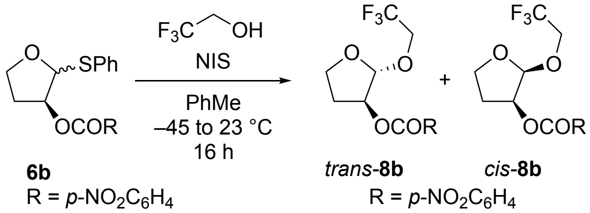

(2*R,3*S**)-2-(2,2,2-Trifluoroethoxy)tetrahydrofuran-3-yl 4-nitrobenzoate (*trans*-8b) and (2*R**,3*R**)-2-(2,2,2-trifluoroethoxy)tetrahydrofuran-3-yl 4-nitrobenzoate (*cis*-8b).** To a solution of thioacetal **6b** (0.0350 g, 0.100 mmol) in PhMe (1 mL) at –45 °C was added 2,2,2-trifluoroethanol (0.0437 mL, 0.600 mmol) followed by the addition of *N*-iodosuccinimide (0.0450 g, 0.200 mmol). After 1 h, the reaction mixture was warmed to 23 °C. After 16 h, a solution of saturated aqueous $Na_2S_2O_3$ (5 mL) was added and the layers were separated. The aqueous layer was extracted with CH_2Cl_2 (2 × 10 mL). The combined organic layers were dried over Na_2SO_4, filtered, and concentrated *in vacuo*. $^{13}C\{^1H\}$ NMR spectroscopic analysis of the unpurified reaction mixture revealed that acetal **8b** was formed as a mixture of diastereomers (*trans*-**8b**:*cis*-**8b** = 82:18) with no formation of the corresponding side-product orthoester **15b**.[1]The spectral data

obtained are consistent with those of products from nucleophilic substitution reactions of thioacetal **6b** and 2,2,2-trifluoroethanol in the presence of *N*-iodosuccinimide in CH_2Cl_2.

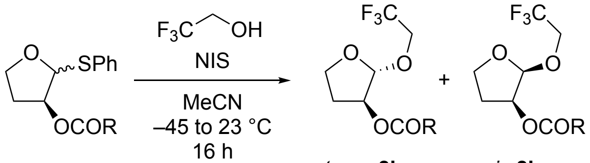

6b
R = *p*-$NO_2C_6H_4$

trans-**8b** *cis*-**8b**
R = *p*-$NO_2C_6H_4$

(2*R,3*S**)-2-(2,2,2-Trifluoroethoxy)tetrahydrofuran-3-yl 4-nitrobenzoate (*trans*-8b) and (2*R**,3*R**)-2-(2,2,2-trifluoroethoxy)tetrahydrofuran-3-yl 4-nitrobenzoate (*cis*-8b).** To a solution of thioacetal **6b** (0.0350 g, 0.100 mmol) in MeCN (1 mL) at –45 °C was added 2,2,2-trifluoroethanol (0.0437 mL, 0.600 mmol) followed by the addition of *N*-iodosuccinimide (0.0450 g, 0.200 mmol). After 1 h, the reaction mixture was warmed to 23 °C. After 16 h, a solution of saturated aqueous $Na_2S_2O_3$ (5 mL) was added and the layers were separated. The aqueous layer was extracted with CH_2Cl_2 (2 × 10 mL). The combined organic layers were dried over Na_2SO_4, filtered, and concentrated *in vacuo*. $^{13}C\{^1H\}$ NMR spectroscopic analysis of the unpurified reaction mixture revealed that acetal **8b** was formed as a mixture of diastereomers (*trans*-**8b**:*cis*-**8b** = 57:43) with no formation of the corresponding side-product orthoester **15b**.[1] The spectral data obtained are consistent with those of products from nucleophilic substitution reactions of thioacetal **8b** and 2,2,2-trifluoroethanol in the presence of *N*-iodosuccinimide in CH_2Cl_2.

VI. Parameter Screen for Substitution Reactions with Allyltrimethylsilane

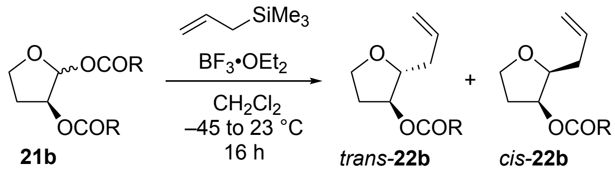

21b
R = *p*-$NO_2C_6H_4$

trans-**22b** *cis*-**22b**
R = *p*-$NO_2C_6H_4$

(2*R,3*S**)-2-Allyltetrahydrofuran-3-yl 4-nitrobenzoate (*trans*-22b) and (2*R**,3*R**)-2-allyltetrahydrofuran-3-yl 4-nitrobenzoate (*cis*-22b).** To a solution of *p*-nitrobenzoate **21b** (0.200 g, 0.497 mmol) in CH_2Cl_2 (5 mL) at –45 °C was added allyltrimethylsilane (0.316 mL, 1.99 mmol) followed by the addition of BF_3•OEt_2 (0.123 mL, 0.994 mmol). After 1 h, the reaction mixture was warmed to 23 °C. After 16 h, a solution of saturated aqueous $NaHCO_3$ (5 mL) was added and the layers were separated. The

aqueous layer was extracted with CH_2Cl_2 (2 × 10 mL). The combined organic layers were dried over Na_2SO_4, filtered, and concentrated *in vacuo*. $^{13}C\{^1H\}$ NMR spectroscopic analysis of the unpurified reaction mixture revealed that *p*-nitrobenzoate **22b** was formed as a mixture of diastereomers (*trans*-**22b**:*cis*-**22b** = 60:40).[1] The spectral data obtained are consistent with those of products from nucleophilic substitution reactions of *p*-nitrobenzoate **21b** and allyltrimethylsilane in the presence of $SnCl_4$.

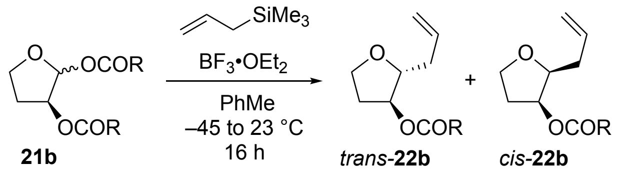

21b
R = *p*-$NO_2C_6H_4$

trans-**22b** *cis*-**22b**
R = *p*-$NO_2C_6H_4$

(2*R,3*S**)-2-Allyltetrahydrofuran-3-yl 4-nitrobenzoate (*trans*-22b) and (2*R**,3*R**)-2-allyltetrahydrofuran-3-yl 4-nitrobenzoate (*cis*-22b).** To a solution of 4-nitrobenzoate **21b** (0.0300 g, 0.0746 mmol) in PhMe (0.75 mL) at –45 °C was added allyltrimethylsilane (0.0591 mL, 0.372 mmol) followed by the addition of BF_3•OEt_2 (0.0230 mL, 0.186 mmol). After 1 h, the reaction mixture was warmed to 23 °C. After 16 h, a solution of saturated aqueous $NaHCO_3$ (5 mL) was added and the layers were separated. The aqueous layer was extracted with CH_2Cl_2 (2 × 10 mL). The combined organic layers were dried over Na_2SO_4, filtered, and concentrated *in vacuo*. $^{13}C\{^1H\}$ NMR spectroscopic analysis of the unpurified reaction mixture revealed that *p*-nitrobenzoate **22b** was formed as a mixture of diastereomers (*trans*-**22b**:*cis*-**22b** = 70:30).[1] The spectral data obtained are consistent with those of products from nucleophilic substitution reactions of *p*-nitrobenzoate **21b** and allyltrimethylsilane in the presence of $SnCl_4$.

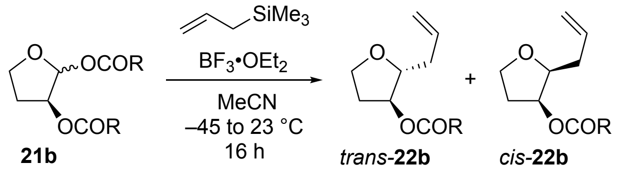

21b
R = *p*-$NO_2C_6H_4$

trans-**22b** *cis*-**22b**
R = *p*-$NO_2C_6H_4$

(2*R,3*S**)-2-Allyltetrahydrofuran-3-yl 4-nitrobenzoate (*trans*-22b) and (2*R**,3*R**)-2-allyltetrahydrofuran-3-yl 4-nitrobenzoate (*cis*-22b).** To a solution of *p*-nitrobenzoate **21b** (0.0300 g, 0.0746 mmol) in MeCN (0.75 mL) at –45 °C was added allyltrimethylsilane (0.0591 mL, 0.372 mmol) followed by the addition of BF_3•OEt_2 (0.0230 mL, 0.186 mmol). After 1 h, the reaction mixture was warmed to 23 °C. After 16 h, a solution of saturated aqueous $NaHCO_3$ (5 mL) was added and the layers were separated. The aqueous layer was extracted with CH_2Cl_2 (2 × 10 mL). The combined organic layers were

dried over Na_2SO_4, filtered, and concentrated *in vacuo*. $^{13}C\{^1H\}$ NMR spectroscopic analysis of the unpurified reaction mixture revealed that *p*-nitrobenzoate **22b** was formed as a mixture of diastereomers (*trans*-**22b**:*cis*-**22b** = 58:42).[1] The spectral data obtained are consistent with those of products from nucleophilic substitution reactions of *p*-nitrobenzoate **21b** and allyltrimethylsilane in the presence of $SnCl_4$.

VII. Stereochemical Proofs

- **Proofs of the Stereochemical Configurations of Acetals 9a–13a and 9b–13b**

Extensive studies have shown that *J*-coupling constants could provide evidence for the spatial orientation between two adjacent protons in cyclic systems such as five- and six-membered rings.[3] The relative stereochemical configurations between the C-1-allyl group and the acyl groups at C-2 in acetals **9a**–**13a** and **9b**–**13b** were assigned by analysis of ^1H NMR and *J*-coupling constants of relevant protons.

In acetals acetals **9a**–**13a** and **9b**–**13b**, the alkoxy substituent at C-1 prefers to be at an axial orientation due to the stabilizing orbital interactions. Distinctive splitting patterns of protons at C-1 and C-2 were observed for equatorial and axial conformations, with $J_{ax–ax}$ constants (9–12 Hz) being much larger than $J_{ax–eq}$ (2–6 Hz) and $J_{eq–eq}$ (2–9 Hz).

Note: In this section, only details of relevant signals were shown to provide evidence for conformational analysis of the stereochemistry between the substituents at C-1 and C-2. Full characterization of compounds is shown in sections VII and VIII.

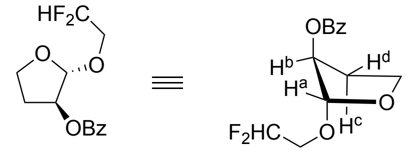

trans-**9a**

Acetal *trans*-**9a** (major diastereomer, 1,2-*trans*):

^1H NMR (400 MHz, $CDCl_3$) δ 5.39 (H^b dd, J = 5.9 ($eq^b–ax^c$), 1.7 ($eq^b–eq^d$) Hz, 1H), 5.21 (H^a br s, ($eq^b–eq^a$), 1H).

Note: The small coupling constant observed from H^a to H^b (br s) suggests that H^a adopts an equatorial orientation, in agreement with the preference for the axial orientation of C-1-alkoxy groups concluded from the anomeric effect. The equatorial orientation of H^b can be concluded from the small coupling constant between H^b and H^d (1.7 Hz) and the moderate coupling constant observed from H^b to H^c (5.9 Hz). In

conclusion, the *J*-coupling constants of H[a] and H[b] reveal that the relative stereochemical configuration of acetal *trans*-**9a** is 1,2-*trans.*

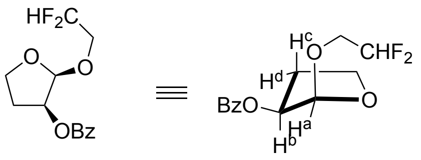

cis-**9a**

Acetal *cis*-**9a** (minor diastereomer, 1,2-*cis*):

^{1}H NMR (400 MHz, CDCl$_3$) δ 5.30 (H^a d, J = 4.2 (eq^a–ax^b) Hz, 1H), 5.17 (H^b td, J = 8.6 (ax^b–eq^d), 4.3 (ax^b–eq^a/eq^d) Hz, 1H).

Note: The larger coupling constant observed from H[a] to H[b] (4.1 Hz) compared to the *J*-coupling constant of H[a] to H[b] (br s) of the 1,2-*trans* diastereomer *trans*-**9a** suggests that H[a] adopts an equatorial orientation, with the C-1-alkoxy group in an axial orientation. The axial orientation of H[b] can be concluded from the large coupling constants observed from H[b] to H[c] (8.6 Hz). In conclusion, the *J*-coupling constants of H[a] and H[b] reveal that the relative stereochemical configuration of acetal *cis*-**9a** is 1,2-*cis.*

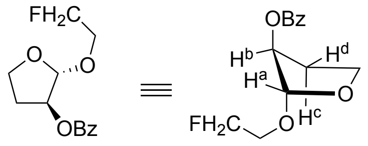

trans-**10a**

Acetal *trans*-**10a** (major diastereomer, 1,2-*trans*):

^{1}H NMR (400 MHz, CDCl$_3$) δ 5.39 (H^b dt, J = 5.9 (eq^b–ax^c), 1.9 (eq^b–eq^d) Hz, 1H), 5.21 (H^a br s, (eq^b–eq^a), 1H).

Note: The small coupling constant observed from H[a] to H[b] (br s) suggests that H[a] adopts an equatorial orientation, in agreement with the preference for the axial orientation of C-1-alkoxy groups concluded from the anomeric effect. The equatorial orientation of H[b] can be concluded from the small coupling constant between H[b] and H[d] (1.9 Hz) and the moderate coupling constant observed from H[b] to H[c] (5.9 Hz). In conclusion, the *J*-coupling constants of H[a] and H[b] reveal that the relative stereochemical configuration of acetal *trans*-**10a** is 1,2-*trans.*

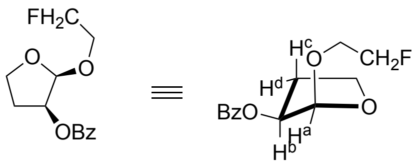

cis-**10a**

Acetal *cis*-**10a** (minor diastereomer, 1,2-*cis*):

^{1}H NMR (400 MHz, $CDCl_3$) δ 5.30 (H^a d, J = 4.4 (eq^a–ax^b) Hz, 1H), 5.17 (H^b td, J = 8.5 (ax^b–ax^c), 4.1 (ax^b–eq^a/eq^d) Hz, 1H).

Note: The larger coupling constant observed from H^a to H^b (4.1 Hz) compared to the *J*-coupling constant of H^a to H^b (br s) of the 1,2-*trans* diastereomer *trans*-**10a** suggests that H^a adopts an equatorial orientation, with the C-1-alkoxy group in an axial orientation. The axial orientation of H^b can be concluded from the large coupling constants observed from H^b to H^c (8.5 Hz). In conclusion, the *J*-coupling constants of H^a and H^b reveal that the relative stereochemical configuration of acetal *cis*-**10a** is 1,2-*cis.*

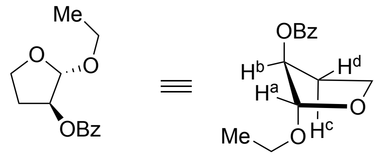

trans-**11a**

Acetal *trans*-**11a** (major diastereomer, 1,2-*trans*):

^{1}H NMR (400 MHz, $CDCl_3$) δ 5.33 (H^b dd, J = 6.2 (eq^b–ax^c), 1.5 (eq^b–eq^d) Hz, 1H), 5.16 (H^a br s, (eq^b–eq^a), 1H).

Note: The small coupling constant observed from H^a to H^b (br s) suggests that H^a adopts an equatorial orientation, in agreement with the preference for the axial orientation of C-1-alkoxy groups concluded from the anomeric effect. The equatorial orientation of H^b can be concluded from the small coupling constant between H^b and H^d (1.5 Hz) and the moderate coupling constant observed from H^b to H^c (6.2 Hz). In conclusion, the *J*-coupling constants of H^a and H^b reveal that the relative stereochemical configuration of acetal *trans*-**11a** is 1,2-*trans.*

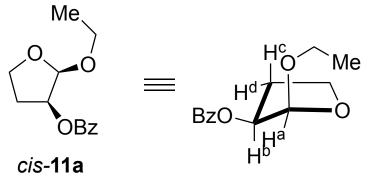

cis-**11a**

Acetal *cis*-**11a** (minor diastereomer, 1,2-*cis*):

^{1}H NMR (400 MHz, CDCl$_3$) δ 5.25 (H^a d, J = 4.6 (eq^a–ax^b) Hz, 1H), 5.15 (H^b td, J = 8.6 (ax^b–aq^c), 4.4 (ax^b–eq^a/eq^d) Hz, 1H).

Note: The larger coupling constant observed from H^a to H^b (4.6 Hz) compared to the J-coupling constant of H^a to H^b (br s) of the 1,2-*trans* diastereomer *trans*-**11a** suggests that H^a adopts an equatorial orientation, with the C-1-alkoxy group in an axial orientation. The axial orientation of H^b can be concluded from the large coupling constants observed from H^b to H^c (8.6 Hz). In conclusion, the J-coupling constants of H^a and H^b reveal that the relative stereochemical configuration of acetal *cis*-**11a** is 1,2-*cis.*

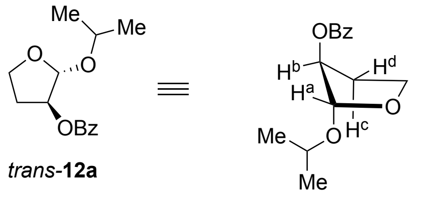

trans-**12a**

Acetal *trans*-**12a** (major diastereomer, 1,2-*trans*):

^{1}H NMR (400 MHz, CDCl$_3$) δ 5.29 (H^b dd, J = 5.9 (eq^b–ax^c), 1.6 (eq^b–eq^d) Hz, 1H), 5.25 (H^a br s, (eq^b–eq^a), 1H).

Note: The small coupling constant observed from H^a to H^b (br s) suggests that H^a adopts an equatorial orientation, in agreement with the preference for the axial orientation of C-1-alkoxy groups concluded from the anomeric effect. The equatorial orientation of H^b can be concluded from the small coupling constant between H^b and H^d (1.6 Hz) and the moderate coupling constant observed from H^b to H^c (5.9 Hz). In conclusion, the J-coupling constants of H^a and H^b reveal that the relative stereochemical configuration of acetal *trans*-**12a** is 1,2-*trans.*

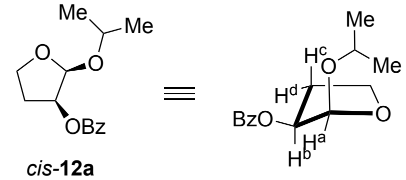

cis-**12a**

Acetal *cis*-**12a** (minor diastereomer, 1,2-*cis*):

^{1}H NMR (400 MHz, CDCl$_3$) δ 5.35 (H^a d, J = 4.4 (eq^a–ax^b) Hz, 1H), 5.12 (H^b td, J = 8.5 (ax^b–ax^c), 4.2 (ax^b–eq^a/eq^d) Hz, 1H).

Note: The larger coupling constant observed from H^a to H^b (4.4 Hz) compared to the J-coupling constant of H^a to H^b (br s) of the 1,2-*trans* diastereomer *trans*-**12a** suggests that H^a adopts an equatorial orientation,

with the C-1-alkoxy group in an axial orientation. The axial orientation of H^b can be concluded from the large coupling constants observed from H^b to H^c (8.5 Hz). In conclusion, the *J*-coupling constants of H^a and H^b reveal that the relative stereochemical configuration of acetal *cis*-**12a** is 1,2-*cis.*

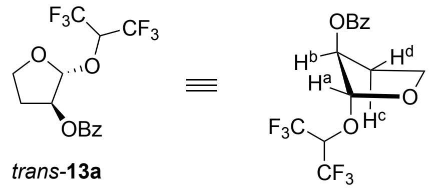

trans-**13a**

Acetal *trans*-**13a** (single diastereomer, 1,2-*trans*):

^{1}H NMR (400 MHz, $CDCl_3$) δ 5.51 (H^b dd, *J* = 5.8 (eq^b–ax^c), 1.1 (eq^b–eq^d) Hz, 1H), 5.43 (H^a br s, (eq^b–eq^a), 1H).

Note: The small coupling constant observed from H^a to H^b (br s) suggests that H^a adopts an equatorial orientation, in agreement with the preference for the axial orientation of C-1-alkoxy groups concluded from the anomeric effect. The equatorial orientation of H^b can be concluded from the small coupling constant between H^b and H^d (1.1 Hz) and the moderate coupling constant observed from H^b to H^c (5.8 Hz). In conclusion, the *J*-coupling constants of H^a and H^b reveal that the relative stereochemical configuration of acetal *trans*-**13a** is 1,2-*trans.*

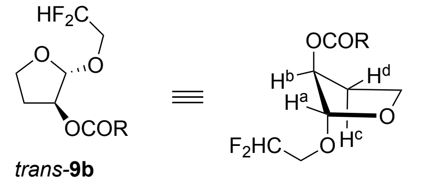

trans-**9b**
R = *p*-$NO_2C_6H_4$

Acetal *trans*-**9b** (single diastereomer, 1,2-*trans*):

^{1}H NMR (400 MHz, $CDCl_3$) δ 5.42 (H^b dd, *J* = 6.5 (eq^b–ax^c), 1.6 (eq^b–eq^d) Hz, 1H), 5.22 (H^a br s, (eq^b–eq^a), 1H).

Note: The small coupling constant observed from H^a to H^b (br s) suggests that H^a adopts an equatorial orientation, in agreement with the preference for the axial orientation of C-1-alkoxy groups concluded from the anomeric effect. The equatorial orientation of H^b can be concluded from the small coupling constant between H^b and H^d (1.6 Hz) and the moderate coupling constant observed from H^b to H^c (6.5 Hz). In conclusion, the *J*-coupling constants of H^a and H^b reveal that the relative stereochemical configuration of acetal *trans*-**9b** is 1,2-*trans.*

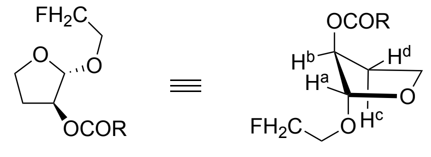

trans-**10b**
R = *p*-$NO_2C_6H_4$

Acetal *trans*-**10b** (major diastereomer, 1,2-*trans*):

^{1}H NMR (400 MHz, $CDCl_3$) δ 5.41 (H^b dt, *J* = 3.9 (eq^b–ax^c), 1.7 (eq^b–eq^d) Hz, 1H), 5.22 (H^a br s, (eq^b–eq^a), 1H).

Note: The small coupling constant observed from H^a to H^b (br s) suggests that H^a adopts an equatorial orientation, in agreement with the preference for the axial orientation of C-1-alkoxy groups concluded from the anomeric effect. The equatorial orientation of H^b can be concluded from the small coupling constant between H^b and H^d (1.7 Hz) and the moderate coupling constant observed from H^b to H^c (3.9 Hz). In conclusion, the *J*-coupling constants of H^a and H^b reveal that the relative stereochemical configuration of acetal *trans*-**10b** is 1,2-*trans.*

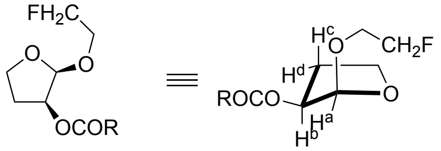

cis-**10b**
R = *p*-$NO_2C_6H_4$

Acetal *cis*-**10b** (minor diastereomer, 1,2-*cis*):

^{1}H NMR (400 MHz, $CDCl_3$) δ 5.31 (H^a d, *J* = 4.3 (eq^a–ax^b) Hz, 1H), 5.21 (H^b td, *J* = 8.4 (ax^b–ax^c), 4.3 (ax^b–eq^a/eq^d) Hz, 1H).

Note: The larger coupling constant observed from H^a to H^b (4.3 Hz) compared to the *J*-coupling constant of H^a to H^b (br s) of the 1,2-*trans* diastereomer *trans*-**10b** suggests that H^a adopts an equatorial orientation, with the C-1-alkoxy group in an axial orientation. The axial orientation of H^b can be concluded from the large coupling constants observed between H^b and H^c (8.4 Hz). In conclusion, the *J*-coupling constants of H^a and H^b reveal that the relative stereochemical configuration of acetal *cis*-**10b** is 1,2-*cis.*

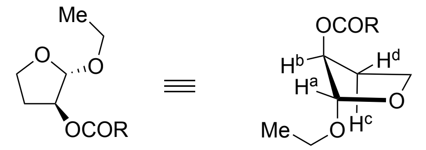

trans-**11b**
R = *p*-$NO_2C_6H_4$

Acetal *trans*-**11b** (major diastereomer, 1,2-*trans*):

^{1}H NMR (400 MHz, $CDCl_3$) δ 5.36 (H^b dd, J = 6.2 (eq^b–ax^c), 1.5 (eq^b–eq^d) Hz, 1H), 5.17 (H^a br s, (eq^b–eq^a), 1H).

Note: The small coupling constant observed from H^a to H^b (br s) suggests that H^a adopts an equatorial orientation, in agreement with the preference for the axial orientation of C-1-alkoxy groups concluded from the anomeric effect. The equatorial orientation of H^b can be concluded from the small coupling constant between H^b and H^d (1.5 Hz) and the moderate coupling constant observed from H^b to H^c (6.2 Hz). In conclusion, the *J*-coupling constants of H^a and H^b reveal that the relative stereochemical configuration of acetal *trans*-**11b** is 1,2-*trans.*

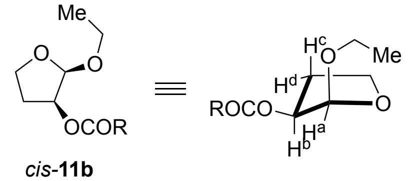

cis-**11b**
R = *p*-$NO_2C_6H_4$

Acetal *cis*-**11b** (minor diastereomer, 1,2-*cis*):

^{1}H NMR (400 MHz, $CDCl_3$) δ 5.27 (H^a d, J = 4.3 (eq^a–ax^b) Hz, 1H), 5.19 (H^b td, J = 8.1 (ax^b–ax^c), 4.4 (ax^b–eq^a/eq^d) Hz, 1H).

Note: The larger coupling constant observed from H^a to H^b (4.3 Hz) compared to the *J*-coupling constant of H^a to H^b (br s) of the 1,2-*trans* diastereomer *trans*-**11b** suggests that H^a adopts an equatorial orientation, with the C-1-alkoxy group in an axial orientation. The axial orientation of H^b can be concluded from the large coupling constants observed from H^b to H^c (8.1 Hz). In conclusion, the *J*-coupling constants of H^a and H^b reveal that the relative stereochemical configuration of acetal *cis*-**11b** is 1,2-*cis.*

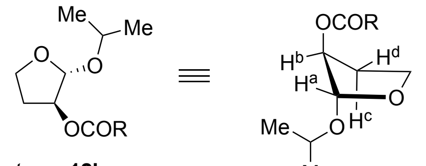

trans-**12b**
R = *p*-$NO_2C_6H_4$

Acetal *trans*-**12b** (major diastereomer, 1,2-*trans*):

^{1}H NMR (400 MHz, $CDCl_3$) δ 5.32 (H^b dd, J = 6.2 (eq^b–ax^c), 1.2 (eq^b–eq^d) Hz, 1H), 5.26 (H^a br s, (eq^b–eq^a), 1H).

Note: The small coupling constant observed from H^a to H^b (br s) suggests that H^a adopts an equatorial orientation, in agreement with the preference for the axial orientation of C-1-alkoxy groups concluded from the anomeric effect. The equatorial orientation of H^b can be concluded from the small coupling constant between H^b and H^d (1.2 Hz) and the moderate coupling constant observed from H^b to H^c (6.2 Hz). In conclusion, the *J*-coupling constants of H^a and H^b reveal that the relative stereochemical configuration of acetal *trans*-**12b** is 1,2-*trans.*

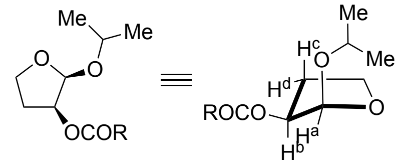

cis-**12b**
R = *p*-$NO_2C_6H_4$

Acetal *cis*-**12b** (minor diastereomer, 1,2-*cis*):

^{1}H NMR (400 MHz, $CDCl_3$) δ 5.36 (H^a d, J = 4.5 (eq^a–ax^b) Hz, 1H), 5.16 (H^b ddd, J = 11.9 (ax^b–ax^c), 7.5 (ax^b–eq^d), 4.2 (ax^b–eq^a) Hz, 1H).

Note: The larger coupling constant observed from H^a to H^b (4.5 Hz) compared to the *J*-coupling constant of H^a to H^b (br s) of the 1,2-*trans* diastereomer *trans*-**12b** suggests that H^a adopts an equatorial orientation, with the C-1-alkoxy group in an axial orientation. The axial orientation of H^b can be concluded from the large coupling constants observed from H^b to H^c (11.9 Hz). In conclusion, the *J*-coupling constants of H^a and H^b reveal that the relative stereochemical configuration of acetal *cis*-**12b** is 1,2-*cis.*

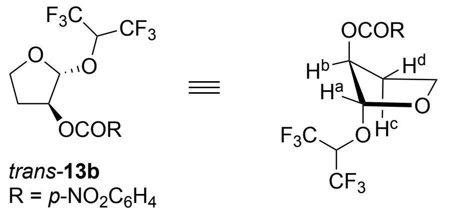

Acetal *trans*-**13b** (single diastereomer, 1,2-*trans*):

^{1}H NMR (400 MHz, $CDCl_3$) δ 5.54 (H^b dd, J = 5.8 (eq^b–ax^c), 1.2 (eq^b–eq^d) Hz, 1H), 5.45 (H^a br s, (eq^b–eq^a), 1H).

Note: The small coupling constant observed from H^a to H^b (br s) suggests that H^a adopts an equatorial orientation, in agreement with the preference for the axial orientation of C-1-alkoxy groups concluded from the anomeric effect. The equatorial orientation of H^b can be concluded from the small coupling constant between H^b and H^d (1.2 Hz) and the moderate coupling constant observed from H^b to H^c (5.8 Hz). In conclusion, the *J*-coupling constants of H^a and H^b reveal that the relative stereochemical configuration of acetal *trans*-**13b** is 1,2-*trans.*

- **Proofs of the Stereochemical Configurations of Acetals 22a and 22b**

The relative stereochemical configurations between the C-1-allyl group and the acyloxy group at C-2 of acetals **22a** and **22b** were confirmed by chemical correlation reactions to the previously reported C-1-allyl/C-2-benzyloxy acetal **S5** (Figure S1).[4]

Figure S1. Chemical correlation reactions for the stereochemical proofs of acetals **22a** and **22b**.

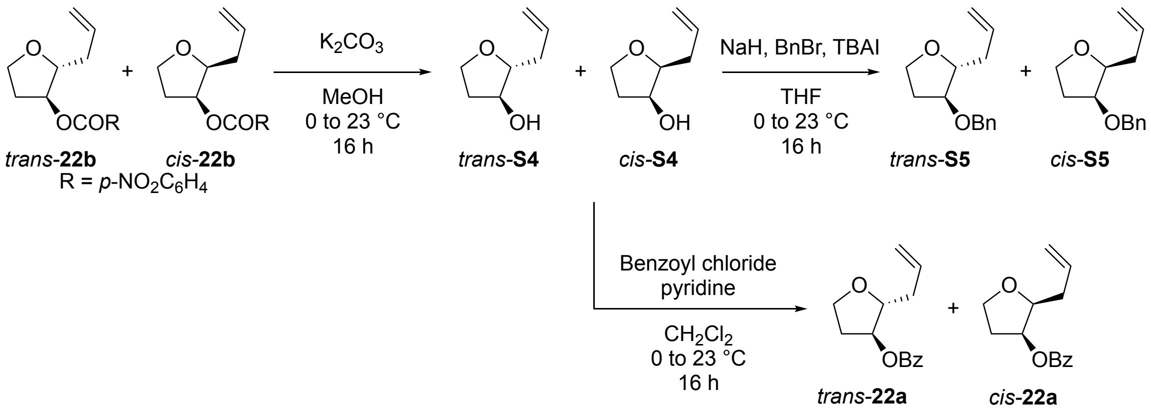

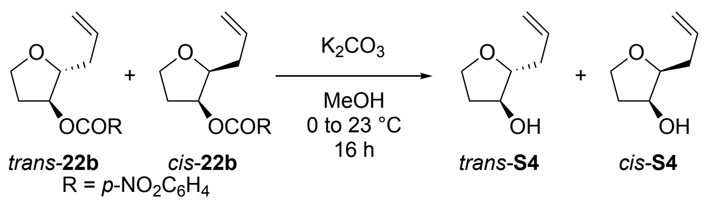

(2*R,3*S**)-2-Allyltetrahydrofuran-3-ol (*trans*-S4) and (2*R**,3*R**)-2-allyltetrahydrofuran-3-ol (*cis*-S4).** To a solution of *p*-nitrobenzoate **22b** as a mixture of diastereomers (0.0230 g, 0.0829 mmol, *trans*-**22b**:*cis*-**22b** = 72:28) in MeOH (0.8 mL) was added K_2CO_3 (0.0120 mg, 0.0829 mmol) at 0 °C. After 1 h, the reaction mixture was warmed to 23 °C. After 16 h, the reaction mixture was diluted with EtOAc (5 mL) and a solution of saturated aqueous NH_4Cl (5 mL) was added. The layers were separated, and the aqueous layer was extracted with EtOAc (2 × 5 mL). The combined organic layers were dried over Na_2SO_4, filtered, and concentrated *in vacuo*. $^{13}C\{^1H\}$ NMR spectroscopic analysis of the unpurified reaction mixture revealed that alcohol **S4** was formed as a mixture of diastereomers (*trans*-**S4**:*cis*-**S4** = 73:27).[1] Purification by flash column chromatography (35:65 EtOAc:hexanes) afforded alcohol **S4** as a colorless oil (0.0100 g, 94%) as a mixture of diastereomers (*trans*-**S4**:*cis*-**S4** = 71:29):

^1H NMR (400 MHz, $CDCl_3$) δ 5.95–5.78 (m, 1.4H), 5.22–5.06 (m, 2.8H), 4.30–4.24 (m, 0.4H), 4.11 (dt, J = 6.4, 3.2 Hz, 1H), 4.09–4.02 (m, 0.4H), 3.99–3.87 (m, 2H), 3.81–3.74 (m, 1.4H), 3.67 (td, J = 7.2, 3.2 Hz, 0.4H), 2.52–2.39 (m, 0.8H), 2.35–2.25 (m, 3.4H), 2.00–1.83 (m, 2.4H);

Peaks attributed to the major diastereomer *trans*-**22b**: $^{13}C\{^1H\}$ NMR (100 MHz, $CDCl_3$) δ 134.4 (CH), 117.6 (CH_2), 85.5 (CH), 75.7 (CH), 66.6 (CH_2), 38.1 (CH_2), 35.0 (CH_2);

Peaks attributed to the minor diastereomer *cis*-**22b**: $^{13}C\{^1H\}$ NMR (100 MHz, $CDCl_3$) δ 134.9 (CH), 117.2 (CH_2), 82.3 (CH), 72.5 (CH), 65.9 (CH_2), 35.6 (CH_2), 33.6 (CH_2);

HRMS (TOF MS ES^+) m / z calcd for $C_7H_{13}O_2$ $(M + H)^+$ 129.0910, found 129.0913.

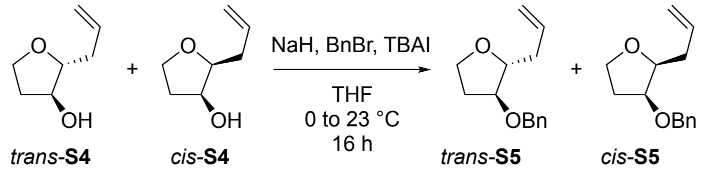

(2*R,3*S**)-2-Allyl-3-(benzyloxy)tetrahydrofuran (*trans*-S5) and (2*R**,3*R**)-2-allyl-3-(benzyloxy)tetrahydrofuran (*cis*-S5).** To a solution of NaH (0.0041 g, 60% in mineral oil, 0.103 mmol) in THF (0.5 mL) was added alcohol **S4** as a mixture of diastereomers (0.0120 mg, 0.0936 mmol, *trans*-**S4**:*cis*-

S4 = 72:28) in THF (0.5 mL) at 0 °C. After 1 h, BnBr (0.0180 mL, 0.0983 mmol) was added dropwise followed by the addition of *n*-Bu_4NI (0.0035 g, 10 mol%). After 1 h, the reaction mixture was warmed to 23 °C. After 16 h, the reaction mixture was diluted with Et_2O (5 mL) and H_2O (5 mL) was added. The layers were separated, and the aqueous layer was extracted with Et_2O (2 × 5 mL). The combined organic layers were dried over Na_2SO_4, filtered, and concentrated *in vacuo*. $^{13}C\{^1H\}$ NMR spectroscopic analysis of the unpurified reaction mixture revealed that acetal **S5** was obtained as a mixture of diastereomers (*trans*-**S5**:*cis*-**S5** = 73:27).[1] Purification by flash column chromatography (10:90 EtOAc:hexanes) afforded acetal **S5** as a yellow oil (0.0160 g, 79%) as a mixture of diastereomers (*trans*-**S5**:*cis*-**S5** = 73:27). The spectral data obtained are consistent with those reported.[4]

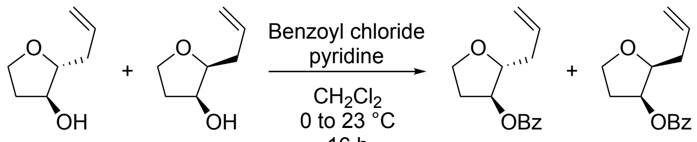

trans-**S4** *cis*-**S4** *trans*-**22a** *cis*-**22a**

(2*R,3*S**)-2-Allyltetrahydrofuran-3-yl benzoate (*trans*-22a) and (2*R**,3*R**)-2-allyltetrahydrofuran-3-yl benzoate (*cis*-22a).** To a solution of alcohol **S4** (0.0050 g, 0.0390 mmol) as a mixture of diastereomers (*trans*-**S4**:*cis*-**S4** = 72:28) in CH_2Cl_2 (0.5 mL) at 0 °C was added pyridine (0.1 mL, 24.3 mmol). After 30 min, benzoyl chloride (0.0009 mL, 0.078 mmol) was added dropwise. The reaction mixture was stirred at 0 °C for an additional 30 min and then it was warmed to 23 °C. After 16 h, the reaction mixture was diluted with CH_2Cl_2 (4 mL) and a solution of saturated aqueous NH_4Cl (4 mL) was added. The aqueous layer was extracted with CH_2Cl_2 (2 × 5 mL). The combined organic layers were dried over Na_2SO_4, filtered, and concentrated *in vacuo*. Purification by flash chromatography (10:90 EtOAc:hexanes) afforded benzoate **22a** as a yellow liquid (0.0083 g, 92%) as a mixture of diastereomers (*trans*-**22a**:*cis*-**22a** = 76:24). The spectral data obtained are consistent with those of products from nucleophilic substitution reactions of benzoate **2.90** and allyltrimethylsilane.

VIII. Epimerization Nucleophilic Substitution Studies

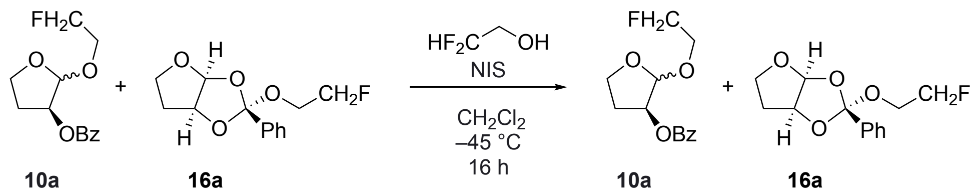

10a 16a 10a 16a

The epimerization nucleophilic substitution reaction of acetal **10a** and orthoester **16a** was performed following the general procedure for nucleophilic substitution reactions of thioacetals using acetal **10a** as a mixture of diastereomers (*trans*-**10a**:*cis*-**10a** = 77:23) and as a mixture with orthoester **16a** (0.0188 g, 0.0740 mmol, *trans*-**10a**:*cis*-**10a**:**16a** = 48:36:16), 2,2-difluoroethanol (0.0281 mL, 0.444 mmol), and *N*-iodosuccinimide (0.0333 g, 0.148 mmol) in CH_2Cl_2 (0.74 mL). $^{13}C\{^1H\}$ NMR spectroscopic analysis of the unpurified reaction mixture revealed that the diastereomeric ratio of acetal **10a** was unchanged (*trans*-**10a**:*cis*-**10a** = 77:23) and the product ratio between acetal **10a** and orthoester **16a** was also unchanged (*trans*-**10a**:*cis*-**10a**:**16a** = 48:36:16).[1]

Note: The observation of the consistent diastereomeric ratio of acetal **10a** and no incorporation of the other nucleophile suggested that nucleophilic substitution reactions of thioacetal **6a** with 2-fluoroethanol in the presence of *N*-iodosuccinimide at –45 °C were under kinetic control. The consistent product ratio between acetal **10a** and orthoester **16a** indicated that no decomposition of orthoester **16a** occurred with *N*-iodosuccinimide at –45 °C.

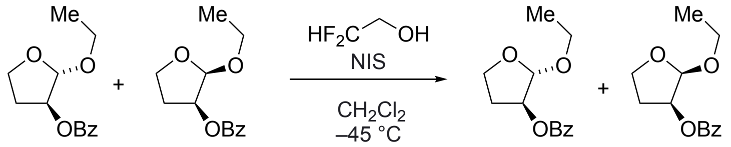

trans-**11a** *cis*-**11a** *trans*-**11a** *cis*-**11a**

The epimerization nucleophilic substitution reaction of acetal **11a** was performed following the general procedure for nucleophilic substitution reactions of thioacetals using acetal **11a** as a mixture of diastereomers (0.0224 g, 0.0944 mmol, *trans*-**11a**:*cis*-**11a** = 95:5), 2,2-difluoroethanol (0.0359 mL, 0.567 mmol), and *N*-iodosuccinimide (0.0420 g, 0.189 mmol) in CH_2Cl_2 (1 mL). $^{13}C\{^1H\}$ NMR spectroscopic analysis of the unpurified reaction mixture revealed that the diastereomeric ratio of acetal **11a** was unchanged (*trans*-**11a**:*cis*-**11a** = 90:10).[1]

Note: The observation of the consistent diastereomeric ratio of acetal **11a** and no incorporation of the other nucleophile suggested that nucleophilic substitution reactions of thioacetal **6a** with ethanol in the presence of *N*-iodosuccinimide at –45 °C were under kinetic control.

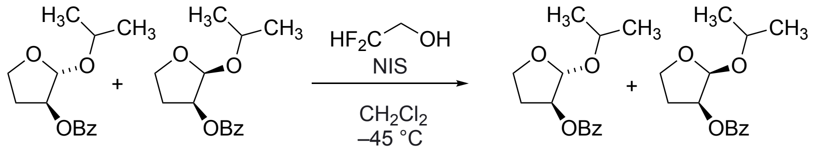

trans-**12a** *cis*-**12a** *trans*-**12a** *cis*-**12a**

The epimerization nucleophilic substitution reaction of acetal **12a** was performed following the general procedure for nucleophilic substitution reactions of thioacetals using acetal **12a** as a mixture of diastereomers (0.0250 g, 0.100 mmol, *trans*-**12a**:*cis*-**12a** = 50:50), 2,2-difluoroethanol (0.0380 mL, 0.600 mmol), and *N*-iodosuccinimide (0.0492 g, 0.600 mmol) in CH_2Cl_2 (1 mL). $^{13}C\{^1H\}$ NMR spectroscopic analysis of the unpurified reaction mixture revealed that the diastereomeric ratio of acetal **12a** was unchanged (*trans*-**12a**:*cis*-**12a** = 49:51).[1]

Note: The observation of the consistent diastereomeric ratio of acetal **12a** and no incorporation of the other nucleophile suggested that nucleophilic substitution reactions of thioacetal **6a** with isopropanol in the presence of *N*-iodosuccinimide at –45 °C were under kinetic control.

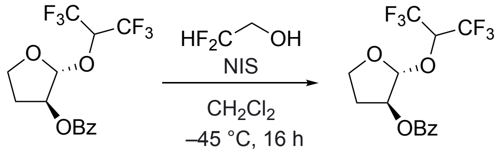

trans-**13a** *trans*-**13a**

The epimerization nucleophilic substitution reaction of acetal *trans*-**13a** was performed following the general procedure for nucleophilic substitution reactions of thioacetals using acetal *trans*-**13a** as a single diastereomer (0.0150 g, 0.0419 mmol), 2,2-difluoroethanol (0.0159 mL, 0.251 mmol), and *N*-iodosuccinimide (0.0188 g, 0.0837 mmol) in CH_2Cl_2 (0.4 mL). $^{13}C\{^1H\}$ NMR spectroscopic analysis of the unpurified reaction mixture revealed that the diastereomeric ratio of acetal *trans*-**13a** was unchanged.[1]

Note: The observation of the consistent diastereomeric ratio of acetal *trans*-**13a** and no incorporation of the other nucleophile suggested that nucleophilic substitution reactions of thioacetal **6a** with 1,1,3,3-hexafluoroisopropanol in the presence of *N*-iodosuccinimide at –45 °C were under kinetic control.

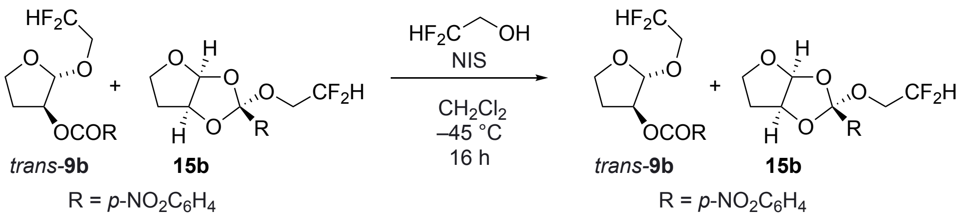

The epimerization nucleophilic substitution reaction of acetal *trans*-**9b** and orthoester **15b** was performed following the general procedure for nucleophilic substitution reactions of thioacetals using acetal *trans*-**9b** as a mixture with orthoester **15b** (0.0200 g, 0.0631 mmol, *trans*-**9b**:**15b** = 48:52), 2-fluoroethanol (0.0222 mL, 0.379 mmol), and *N*-iodosuccinimide (0.0284 g, 0.126 mmol) in CH_2Cl_2 (0.63 mL). $^{13}C\{^1H\}$ NMR spectroscopic analysis of the unpurified reaction mixture revealed that the product ratio between acetal *trans*-**9b** and orthoester **15b** was unchanged (*trans*-**9b**:**15b** = 49:51).[1]

Note: The observation of the consistent product ratio between acetal *trans*-**9b** and orthoester **15b** and no incorporation of the other nucleophile suggested that nucleophilic substitution reactions of thioacetal **6b** with 2-fluoroethanol in the presence of *N*-iodosuccinimide at –45 °C were under kinetic control and no decomposition of orthoester **15b** occurred with *N*-iodosuccinimide at –45 °C.

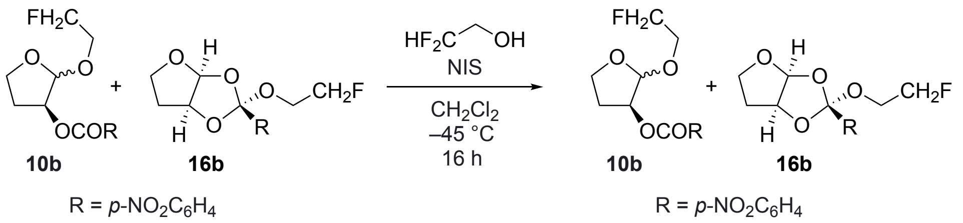

The epimerization nucleophilic substitution reaction of acetal **10b** and orthoester **16b** was performed following the general procedure for nucleophilic substitution reactions of thioacetals using acetal **10b** as a mixture of diastereomers (*trans*-**10b**:*cis*-**10b** = 92:8) and as a mixture with orthoester **16b** (0.0360 g, 0.121 mmol, *trans*-**10b**:*cis*-**10b**:**16b** = 56:7:37), 2,2-difluoroethanol (0.0500 mL, 0.726 mmol), and *N*-iodosuccinimide (0.0544 g, 0.241 mmol) in CH_2Cl_2 (1.2 mL). $^{13}C\{^1H\}$ NMR spectroscopic analysis of the unpurified reaction mixture revealed that the diastereomeric ratio of acetal **10b** unchanged (*trans*-**10b**:*cis*-**10b** = 92:8) and the product ratio between acetal **10b** and orthoester **16b** was also unchanged (*trans*-**10b**:*cis*-**10b**: **16b** = 58:6:36).[1]

Note: The observation of the consistent diastereomeric ratio of acetal **10b** and no incorporation of the other nucleophile suggested that nucleophilic substitution reactions of thioacetal **6b** with 2-fluoroethanol in the presence of *N*-iodosuccinimide at –45 °C were under kinetic control. The consistent product ratio between acetal **10b** and orthoester **16b** indicated that no decomposition of orthoester **16b** occurred with *N*-iodosuccinimide at –45 °C.

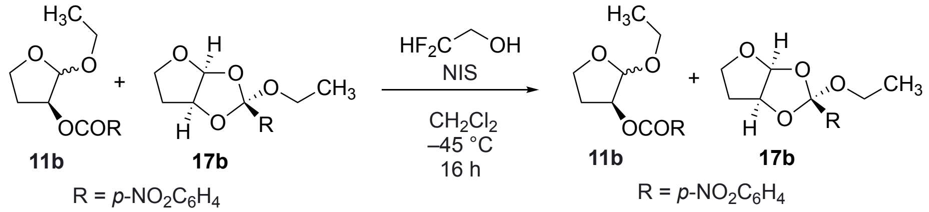

The epimerization nucleophilic substitution reaction of acetal **11b** and orthoester **17b** was performed following the general procedure for nucleophilic substitution reactions of thioacetals using acetal **11b** as a mixture of diastereomers (*trans*-**11b**:*cis*-**11b** = 48:52) and as a mixture with orthoester **17b** (0.0140 g, 0.0500 mmol, *trans*-**11b**:*cis*-**11b**:**17b** = 23:28:49), 2,2-difluoroethanol (0.0500 mL, 0.726 mmol), and *N*-iodosuccinimide (0.0544 g, 0.241 mmol) in CH_2Cl_2 (0.5 mL). $^{13}C\{^1H\}$ NMR spectroscopic analysis of the unpurified reaction mixture revealed that the diastereomeric ratio of acetal **11b** was unchanged (*trans*-**11b**:*cis*-**11b** = 50:50) and the product ratio between acetal **11b** and orthoester **17b** was also unchanged (*trans*-**11b**:*cis*-**11b**:**17b** = 23:28:49).[1]

Note: The observation of the consistent diastereomeric ratio of acetal **11b** and no incorporation of the other nucleophile suggested that nucleophilic substitution reactions of thioacetal **6b** with ethanol in the presence of *N*-iodosuccinimide at –45 °C were under kinetic control. The consistent product ratio between acetal **11b** and orthoester **17b** indicated that no decomposition of orthoester **17b** occurred with *N*-iodosuccinimide at –45 °C.

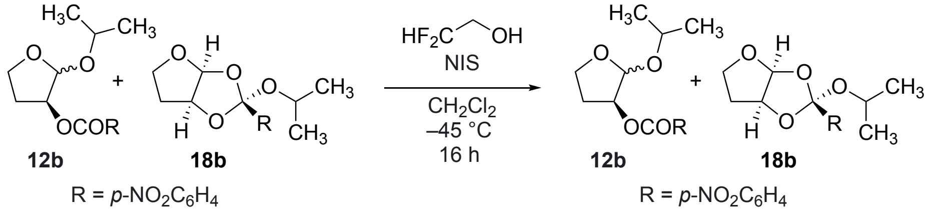

The epimerization nucleophilic substitution reaction of acetal **12b** and orthoester **18b** was performed following the general procedure for nucleophilic substitution reactions of thioacetals using acetal **12b** as a mixture of diastereomers (*trans*-**12b**:*cis*-**12b** = 95:5) and as a mixture with orthoester **18b** (0.0300 g, 0.102 mmol, *trans*-**12b**:*cis*-**12b**:**18b** = 46:2:52), 2,2-difluoroethanol (0.0386 mL, 0.610 mmol), and *N*-iodosuccinimide (0.0460 g, 0.204 mmol) in CH_2Cl_2 (1 mL). $^{13}C\{^1H\}$ NMR spectroscopic analysis of the unpurified reaction mixture revealed that the diastereomeric ratio of acetal **12b** was unchanged (*trans*-**12b**:*cis*-**12b** = 96:4) and the product ratio between acetal **12b** and orthoester **18b** was also unchanged (*trans*-**12b**:*cis*-**12b**:**18b** = 46:2:52).[1]

Note: The observation of the consistent diastereomeric ratio of acetal **12b** and no incorporation of the other nucleophile suggested that nucleophilic substitution reactions of thioacetal **6b** with ethanol in the presence of *N*-iodosuccinimide at –45 °C were under kinetic control. The consistent product ratio between acetal **12b** and orthoester **18b** indicated that no decomposition orthoester **18b** occurred with *N*-iodosuccinimide at –45 °C.

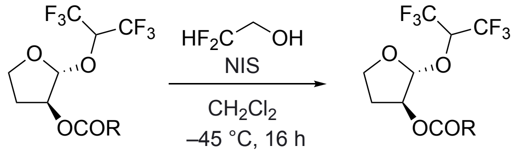

trans-**13b** R = *p*-$NO_2C_6H_4$ → *trans*-**13b** R = *p*-$NO_2C_6H_4$

The epimerization nucleophilic substitution reaction of acetal *trans*-**13b** was performed following the general procedure for nucleophilic substitution reactions of thioacetals using acetal *trans*-**13b** as a single diastereomer (0.0150 g, 0.0372 mmol), 2,2-difluoroethanol (0.0141 mL, 0.223 mmol), and *N*-iodosuccinimide (0.0167 g, 0.0744 mmol) in CH_2Cl_2 (0.4 mL). $^{13}C\{^1H\}$ NMR spectroscopic analysis of the unpurified reaction mixture revealed that the diastereomeric ratio of acetal *trans*-**13b** was unchanged.[1]

Note: The observation of the consistent diastereomeric ratio of acetal *trans*-**13b** and no incorporation of the other nucleophile suggested that nucleophilic substitution reactions of thioacetal **6b** with 1,1,3,3-hexafluoroisopropanol in the presence of *N*-iodosuccinimide at –45 °C were under kinetic control.

IX. Computational methods

a. General computational method

All computations were performed with ORCA5.03[5] at SMD(dichloromethane)-revDSD-PBEP86-D4-def2TZVPP//PCM(dichloromethane)-B3LYP-D3BJ-def2TZVPP[6] level of theory. Geometries were optimized without symmetry constraints. All calculated stationary points have been verified by performing a vibrational analysis, to be energy minima (no imaginary frequencies) or transition states (only one imaginary frequency). The character of the normal mode associated with the imaginary frequency of the transition state has been analyzed with an intrinsic reaction coordinate (IRC) calculation to ensure that it is associated with the reaction of interest. When no clear transition state was found, constrained potential energy surface (PES) analysis was employed to verify the absence of a clear transition state.

b. Generation of the computational energy landscapes

The workflow for the generation of computational energy landscapes (CEL) of pyranosyl[7] cations with ORCA5.03[5] was done similar to previous work.[2] Initial geometries were constructed by a highly constrained relaxed potential energy surface scan using the semi-empirical AM1 functional and the VerySlowConv keyword. All bond distances of directly connected atoms (i.e., C1-H1 O5-C1, C2-O2, etc.) were constrained, as well as the H2-C2-O2-$O_{acyloxy}$ dihedral angle. The subsequent output geometries were then used as starting geometries for the CEL computations with constrains only on the C1-C2-C3-C4 and the C5-O5-C1-C2 dihedral angles. The C1-C2-C3-C4, C3-C4-C5-O5 and the C5-O5-C1-C2 dihedral angles were scanned from -60 to 60 degrees with 15-degree steps, generating 729 unique structures. The subsequent output geometries were then used as starting geometries for the CEL computations with constrains only on the C1-C2-C3-C4, C3-C4-C5-O5 and the C5-O5-C1-C2 dihedral angles. The CEL computations were then performed by optimization of the generated geometries, with constrains on the mentioned dihedral angles, with DFT using the keywords TightSCF, DEFGRID2 (optimization step), DEFGRID3 (single point step) and SlowConv at SMD(dichloromethane)-revDSD-PBEP86-D4-def2TZVPP//PCM(dichloromethane)-B3LYP-D3BJ-def2TZVPP. The CEL maps were visualized using Origin 9.0.0[8] identical to Hansen *et al.*[7]

c. Computational tables and figures

Supplementary Table S1. Cartesian coordinates (in Å), energies (*E*, *H* and *G*, in Hartree), and number of imaginary vibrational frequencies (N_{imag}) of reaction profile geometries. All stationary points and transition states, computed at SMD(dichloromethane)-revDSD-PBEP86-D4-def2TZVPP//PCM(dichloromethane)-B3LYP-D3BJ-def2TZVPP.

Allyltrimethylsilane

E = -525.9194012

H = -525.7314076

G = -525.7644756

N_{imag} = 0

C	2.992237	-0.300160	0.123760
H	3.814618	0.050987	0.733596
H	2.994991	-1.348837	-0.152553
C	2.017649	0.518551	-0.268439
H	2.061936	1.559317	0.041424
C	0.816675	0.129372	-1.062236
H	0.611887	0.872087	-1.839652
H	0.972349	-0.833225	-1.557309
Si	-0.752775	-0.019957	0.000456
C	-1.044533	1.588680	0.925350
H	-1.149486	2.426366	0.232262
H	-0.216328	1.812058	1.600743
H	-1.957467	1.529814	1.522127
C	-2.205853	-0.377489	-1.135288
H	-2.350600	0.433543	-1.852324
H	-3.129191	-0.488998	-0.562517
H	-2.045940	-1.300140	-1.697449
C	-0.526582	-1.427459	1.221454
H	0.331780	-1.245429	1.870536
H	-0.361107	-2.373487	0.701352

H	-1.410786	-1.539208	1.852781

Allyltrimethylstannane

E = -450.5820421

H = -450.3965232

G = -450.4334908

N_{imag} = 0

C	3.174384	-0.131563	0.072087
H	3.940325	0.331418	0.680300
H	3.228491	-1.206935	-0.057089
C	2.202856	0.590178	-0.490598
H	2.197668	1.663731	-0.318103
C	1.074876	0.066185	-1.294892
H	0.830333	0.720274	-2.132115
H	1.264696	-0.939844	-1.669635
Sn	-0.730635	-0.056945	-0.049313
C	-0.928196	1.795466	1.051410
H	-1.021547	2.630476	0.358105
H	-0.049134	1.946945	1.676138
H	-1.813931	1.756695	1.684307
C	-2.471100	-0.384705	-1.295604
H	-2.578026	0.437041	-2.002404
H	-3.362681	-0.438251	-0.671950
H	-2.362351	-1.318606	-1.845342
C	-0.442201	-1.708934	1.315857
H	0.499402	-1.579394	1.847860
H	-0.414030	-2.645730	0.761045
H	-1.257757	-1.743790	2.037080

26–R_{3E}

E = -650.34733149

H = -650.13857426

G = -650.17360035

N_{imag} = 0

O	-0.121315	-0.758504	1.430841
C	0.553598	-1.228327	0.494935
C	0.597075	-0.375343	-0.715476
C	-0.583626	0.582025	-0.486130
C	-0.733765	0.576913	1.026351
H	1.056549	-2.180123	0.643072
H	1.556094	0.164455	-0.682280
H	-0.376069	1.576870	-0.867572
H	-1.471212	0.179280	-0.970914
H	-0.128446	1.312137	1.548331
H	-1.744502	0.546417	1.413947
O	0.531674	-1.177144	-1.871789
C	1.202157	-0.692164	-2.967390
H	2.274827	-0.268318	-5.344574
O	1.787729	0.361485	-2.928027
C	1.108405	-1.595983	-4.127467
C	0.419657	-2.811631	-4.068523
C	0.374642	-3.632056	-5.186424
C	1.011733	-3.245579	-6.362326
C	1.695941	-2.033952	-6.423975
C	1.745489	-1.209612	-5.310787
H	-0.073762	-3.111664	-3.156649
H	-0.156214	-4.573030	-5.141128

H	0.975375	-3.888919	-7.231316
H	2.189848	-1.735166	-7.338283

26−R$_{E3}$

E = -650.34518044

H = -650.1361774100001

G = -650.1717998500001

N_{imag} = 0

O	0.659925	-0.748693	1.070250
C	1.326828	-0.315927	0.113366
C	0.509558	0.336284	-0.964716
C	-0.842243	0.521471	-0.276330
C	-0.817096	-0.450775	0.891325
H	2.397069	-0.500594	0.108231
H	0.967725	1.249210	-1.336375
H	-0.925619	1.545851	0.078906
H	-1.658140	0.313376	-0.963148
H	-1.136076	-0.053309	1.847693
H	-1.254434	-1.426890	0.703918
O	0.404388	-0.641531	-2.008360
C	1.443932	-0.689830	-2.903674
H	3.137620	-1.300236	-4.835457
O	2.448577	-0.043663	-2.732589
C	1.169927	-1.597347	-4.032382
C	-0.062394	-2.239304	-4.189324
C	-0.268904	-3.075454	-5.277083
C	0.748509	-3.277293	-6.205574
C	1.977191	-2.640152	-6.050060

C	2.189436	-1.801004	-4.967669
H	-0.851565	-2.081909	-3.469856
H	-1.222564	-3.569750	-5.401372
H	0.583776	-3.931374	-7.051417
H	2.766453	-2.799092	-6.771853

26−R$_{diox}$

E = -650.37479902

H = -650.16407511

G = -650.19721295

N_{imag} = 0

O	0.128395	-0.507575	1.461232
C	1.155851	-0.655705	0.595238
C	0.806765	0.067986	-0.718634
C	-0.548705	0.692901	-0.437718
C	-1.077153	-0.146610	0.721391
H	2.113400	-0.465238	1.063909
H	1.570542	0.736838	-1.096552
H	-0.394617	1.724625	-0.125273
H	-1.190256	0.683098	-1.315314
H	-1.717418	0.395128	1.408861
H	-1.577591	-1.056813	0.389051
O	0.691008	-1.025230	-1.696515
C	0.950702	-2.158544	-1.127566
H	0.374243	-2.449945	-3.724261
O	1.253551	-2.100913	0.120653
C	0.903208	-3.393226	-1.856004
C	1.180735	-4.597863	-1.191208

C	1.138410	-5.785239	-1.898322
C	0.822324	-5.775323	-3.256037
C	0.546660	-4.578876	-3.916547
C	0.584867	-3.383322	-3.223288
H	1.424176	-4.591456	-0.138904
H	1.350645	-6.718889	-1.397290
H	0.791039	-6.707309	-3.804151
H	0.303982	-4.583486	-4.969609

26–TS$_{IA}$

E = -1176.27761347

H = -1175.87827287

G = -1175.92922951

N_{imag} = 1

O	-1.148951	0.391092	0.645015
C	-0.031710	-0.105946	1.071484
C	1.056487	-0.060512	0.015262
C	0.298814	0.440017	-1.227454
C	-0.940833	1.098129	-0.635575
H	-0.101310	-0.839958	1.855894
H	1.836979	0.636339	0.297980
H	0.897675	1.142011	-1.799868
H	0.024435	-0.397817	-1.862542
H	-0.801922	2.149968	-0.393182
H	-1.852913	0.958807	-1.204559
O	1.761100	-1.292875	-0.146486
C	1.028202	-2.423535	-0.291070
H	3.748774	-2.591283	-0.566197

O	-0.180200	-2.417327	-0.193985
H	1.467994	0.109062	3.813749
H	2.094522	3.753724	4.294957
C	1.641688	0.844157	3.033383
H	3.119183	1.583580	1.685491
H	3.586971	0.112863	2.609794
Si	3.957533	2.153723	3.921036
C	5.713451	2.271104	3.286762
C	3.018934	1.045362	2.629823
H	5.741897	2.719034	2.291856
H	6.177629	1.284779	3.232364
H	6.313941	2.892076	3.955021
C	3.881769	1.316824	5.592023
H	4.457646	1.886497	6.324455
H	4.298828	0.308973	5.545665
H	2.854569	1.245320	5.954064
C	0.555320	1.486312	2.520949
H	0.684045	2.288829	1.804121
H	-0.401923	1.412612	3.016240
C	3.128319	3.829219	3.953898
H	3.128508	4.286455	2.962580
H	3.660299	4.497995	4.633834
C	1.856672	-3.615948	-0.558774
C	1.209740	-4.846986	-0.700447
C	1.947829	-5.992511	-0.955295
C	3.333647	-5.915688	-1.069672
C	3.981345	-4.691540	-0.928829
C	3.247902	-3.541394	-0.673988
H	0.134095	-4.893301	-0.609555

H	1.445804	-6.944020	-1.064748
H	3.908569	-6.810279	-1.268738
H	5.057491	-4.633950	-1.018276

26–TS$_{IB}$

E = -1100.94447120

H = -1100.54803848

G = -1100.60300638

N$_{imag}$ = 1

O	-1.118125	0.346611	0.608997
C	-0.048940	-0.214884	1.019480
C	1.091784	-0.117435	0.039960
C	0.394936	0.423767	-1.220572
C	-0.875270	1.060164	-0.673783
H	-0.108100	-0.834919	1.896567
H	1.844828	0.572366	0.409465
H	1.017700	1.145278	-1.740296
H	0.158216	-0.395887	-1.894528
H	-0.765095	2.109178	-0.409862
H	-1.766747	0.906169	-1.270375
O	1.808909	-1.337490	-0.142650
C	1.061437	-2.460318	-0.247730
H	3.740114	-2.651205	-0.755226
O	-0.140246	-2.429804	-0.067761
C	1.683306	0.915197	3.104705
H	2.058609	4.152360	4.389529
H	1.540597	0.172534	3.884995
H	3.134709	1.670761	1.719043

H	3.634767	0.193908	2.620118
Sn	4.113551	2.385087	4.142710
C	6.147271	2.516808	3.434009
C	3.048422	1.110907	2.649967
H	6.159785	2.967600	2.443214
H	6.581931	1.519915	3.385457
H	6.730115	3.131821	4.118097
C	3.960205	1.399731	6.055047
H	4.512016	1.967723	6.802555
H	4.377268	0.396408	5.985863
H	2.914406	1.337598	6.351376
C	0.590734	1.574263	2.658523
H	0.675251	2.354903	1.911008
H	-0.370164	1.447508	3.136143
C	3.106872	4.291760	4.130808
H	3.179214	4.740216	3.141371
H	3.572309	4.952734	4.860390
C	1.847920	-3.661539	-0.575062
C	1.180464	-4.887904	-0.650266
C	1.885700	-6.041043	-0.957172
C	3.257301	-5.976014	-1.190343
C	3.924480	-4.756264	-1.116795
C	3.224881	-3.598157	-0.810462
H	0.116342	-4.925161	-0.466867
H	1.369397	-6.989363	-1.014691
H	3.806340	-6.876906	-1.429813
H	4.989295	-4.708682	-1.298834

26–TS$_{II}$

E = -650.34511040

H = -650.13676093

G = -650.17037198

N_{imag} = 1

O	0.878732	-0.729325	0.970868
C	1.428263	-0.137680	0.022228
C	0.454109	0.413289	-0.990200
C	-0.840732	0.425353	-0.181903
C	-0.632937	-0.667031	0.851974
H	2.510907	-0.094539	0.002564
H	0.772903	1.375976	-1.383318
H	-0.957032	1.392905	0.302294
H	-1.700443	0.235237	-0.818284
H	-0.976686	-0.448385	1.855900
H	-0.919435	-1.668019	0.542914
O	0.311341	-0.542122	-2.054598
C	1.419369	-0.795637	-2.807461
H	3.189839	-1.638308	-4.580807
O	2.506853	-0.377402	-2.480323
C	1.124931	-1.614749	-3.995988
C	-0.173098	-2.034723	-4.303702
C	-0.397054	-2.803359	-5.436497
C	0.666524	-3.153676	-6.263620
C	1.959850	-2.735581	-5.959300
C	2.191375	-1.968476	-4.828529
H	-0.997586	-1.761583	-3.662782
H	-1.400226	-3.128582	-5.675313
H	0.487713	-3.752994	-7.146347
H	2.784399	-3.008524	-6.603318

26−TS$_{III}$

E = -650.34454817

H = -650.13624091

G = -650.17075279

N$_{imag}$ = 1

O	0.602635	-0.697430	1.204486
C	1.257316	-0.483500	0.170305
C	0.498775	0.183098	-0.933117
C	-0.883705	0.426521	-0.310520
C	-0.817888	-0.191024	1.083314
H	2.288248	-0.825233	0.135275
H	1.016032	1.090776	-1.245936
H	-1.086795	1.491327	-0.253913
H	-1.648846	-0.050091	-0.917169
H	-0.923435	0.507932	1.905806
H	-1.425095	-1.075664	1.242724
O	0.414436	-0.741781	-2.017190
C	1.445370	-0.725220	-2.922637
H	3.123483	-1.200574	-4.906841
O	2.430789	-0.052468	-2.742681
C	1.187025	-1.606059	-4.076529
C	-0.020345	-2.294246	-4.230980
C	-0.213660	-3.104415	-5.340502
C	0.792060	-3.234003	-6.294241
C	1.995654	-2.550035	-6.141868
C	2.194698	-1.737063	-5.037046
H	-0.800482	-2.193513	-3.491818

H	-1.148012	-3.635024	-5.461895
H	0.637745	-3.868080	-7.157095
H	2.775879	-2.652165	-6.883481

27−R$_{3E}$

E = -854.62397480

H = -854.41127322

G = -854.45016348

N_{imag} = 0

O	-0.109159	-0.734932	1.441544
C	0.548814	-1.228311	0.506614
C	0.607284	-0.381995	-0.709226
C	-0.552846	0.599913	-0.483537
C	-0.694969	0.610632	1.029733
H	1.029874	-2.190807	0.658919
H	1.578001	0.135832	-0.679987
H	-0.328594	1.587338	-0.874758
H	-1.450568	0.209676	-0.959870
H	-0.071038	1.336517	1.542743
H	-1.704092	0.604464	1.422659
O	0.521113	-1.186811	-1.865186
C	1.190723	-0.721703	-2.961293
H	2.255142	-0.335643	-5.351586
O	1.805624	0.312887	-2.944081
C	1.060702	-1.630458	-4.124767
C	0.336963	-2.823124	-4.054518
C	0.251457	-3.645848	-5.165954
C	0.895837	-3.256476	-6.331225

C	1.619608	-2.075942	-6.425922
C	1.698691	-1.260021	-5.310704
H	-0.156774	-3.109814	-3.139310
H	-0.301842	-4.570731	-5.132881
N	0.806877	-4.130509	-7.516278
H	2.106129	-1.807629	-7.350050
O	0.148969	-5.155706	-7.422609
O	1.395745	-3.783164	-8.528603

27−R$_{E3}$

E = -854.62167259

H = -854.40872498

G = -854.4483845

N_{imag} = 0

O	0.677156	-0.750605	1.068280
C	1.335677	-0.317412	0.106851
C	0.507196	0.324240	-0.970456
C	-0.846179	0.491301	-0.281026
C	-0.804696	-0.473093	0.892716
H	2.408190	-0.490030	0.098460
H	0.953249	1.243795	-1.340946
H	-0.944292	1.516456	0.068073
H	-1.660127	0.267478	-0.965266
H	-1.125091	-0.073288	1.847629
H	-1.229356	-1.456375	0.713715
O	0.413327	-0.650501	-2.020877
C	1.434041	-0.669743	-2.927964
H	3.118501	-1.268478	-4.876405

O	2.428314	-0.004965	-2.788163
C	1.160411	-1.590512	-4.057046
C	-0.065187	-2.245476	-4.197067
C	-0.277677	-3.089784	-5.274766
C	0.749404	-3.267921	-6.190123
C	1.976150	-2.629872	-6.071505
C	2.176609	-1.782635	-4.995754
H	-0.850941	-2.095774	-3.473311
H	-1.217184	-3.603002	-5.403977
N	0.529980	-4.172883	-7.333859
H	2.749177	-2.794462	-6.804948
O	-0.577229	-4.672689	-7.463396
O	1.468243	-4.376132	-8.089002

27–R$_{diox}$

E = -854.64702163

H = -854.43258761

G = -854.47002323

N$_{imag}$ = 0

O	0.148485	-0.485623	1.462081
C	1.162047	-0.641352	0.590870
C	0.801795	0.065236	-0.729462
C	-0.562335	0.673492	-0.453328
C	-1.070083	-0.142320	0.732367
H	2.128860	-0.461408	1.044188
H	1.556727	0.738134	-1.117187
H	-0.423733	1.715993	-0.171068
H	-1.209913	0.629336	-1.325269

H	-1.699190	0.413777	1.418361
H	-1.572226	-1.061642	0.430014
O	0.698682	-1.039787	-1.702652
C	0.952958	-2.162252	-1.121246
H	0.399908	-2.471321	-3.728048
O	1.249282	-2.106415	0.121387
C	0.905127	-3.410143	-1.850565
C	1.169849	-4.607247	-1.174494
C	1.133637	-5.802170	-1.869422
C	0.832950	-5.769465	-3.223506
C	0.564771	-4.595531	-3.912990
C	0.601788	-3.400903	-3.217658
H	1.401212	-4.600013	-0.120001
H	1.333981	-6.740210	-1.377259
N	0.797598	-7.049101	-3.968838
H	0.335348	-4.620829	-4.966152
O	1.071954	-8.067937	-3.357291
O	0.493040	-7.008256	-5.148976

27–TS$_{IA}$

E = -1380.55439051

H = -1380.15118830

G = -1380.20621783

N_{imag} = 1

O	-1.142973	0.357436	0.636615
C	-0.032559	-0.141206	1.061194
C	1.066269	-0.077435	0.020004
C	0.321257	0.434018	-1.225236

C	-0.934385	1.070734	-0.643306
H	-0.095063	-0.856469	1.863500
H	1.843738	0.614183	0.322960
H	0.920929	1.150904	-1.777726
H	0.065602	-0.396293	-1.878013
H	-0.815775	2.123044	-0.393851
H	-1.839398	0.916963	-1.219571
O	1.773517	-1.310234	-0.150988
C	1.043387	-2.434768	-0.301175
H	3.769478	-2.600149	-0.561182
O	-0.162882	-2.442846	-0.207088
H	1.472885	0.116759	3.825825
H	2.090409	3.762670	4.316343
C	1.636405	0.855613	3.046848
H	3.101699	1.612288	1.693203
H	3.583365	0.138246	2.600436
Si	3.957387	2.171294	3.924338
C	5.709991	2.301564	3.282075
C	3.013480	1.069390	2.636464
H	5.730901	2.755432	2.289602
H	6.179109	1.318038	3.219106
H	6.311325	2.921640	3.950374
C	3.896852	1.326902	5.592708
H	4.473599	1.896693	6.324343
H	4.319512	0.321678	5.539678
H	2.872269	1.247654	5.960583
C	0.546492	1.494488	2.546521
H	0.662482	2.293875	1.823959
H	-0.413790	1.399457	3.032259

C	3.123342	3.844478	3.974033
H	3.119956	4.310511	2.986782
H	3.654260	4.509209	4.658730
C	1.878822	-3.629869	-0.575642
C	1.230024	-4.856564	-0.730696
C	1.962362	-6.002341	-0.988784
C	3.342643	-5.895747	-1.087739
C	4.011151	-4.689309	-0.938480
C	3.269037	-3.548362	-0.679954
H	0.154617	-4.905861	-0.648009
H	1.479556	-6.958687	-1.111164
N	4.129190	-7.112081	-1.361244
H	5.085333	-4.648461	-1.022744
O	3.527624	-8.170239	-1.467442
O	5.341001	-6.998522	-1.466523

27−TS$_{IB}$

E = -1305.22134364

H = -1304.82116823

G = -1304.88023842

N_{imag} = 1

O	-1.101475	0.318011	0.524009
C	-0.048249	-0.247380	0.950294
C	1.120254	-0.130073	0.009477
C	0.457285	0.407168	-1.270027
C	-0.839112	1.023752	-0.763779
H	-0.107702	-0.818412	1.860989
H	1.852533	0.563479	0.413986

H	1.086260	1.139147	-1.766852
H	0.254041	-0.413125	-1.954492
H	-0.758310	2.075793	-0.502746
H	-1.712152	0.844722	-1.380103
O	1.852371	-1.345685	-0.161513
C	1.114456	-2.468586	-0.244583
H	3.781831	-2.643336	-0.822750
O	-0.084059	-2.453492	-0.052136
C	1.620177	0.992673	3.106261
H	3.578822	4.939682	4.981628
H	1.448481	0.259961	3.890273
H	3.112741	1.715055	1.745479
H	3.560127	0.223795	2.642161
Sn	4.083129	2.385233	4.189520
C	6.125271	2.520964	3.503676
C	3.003975	1.159860	2.677248
H	6.153125	3.007489	2.530170
H	6.550860	1.522173	3.422410
H	6.708499	3.104081	4.214898
C	3.920543	1.360657	6.081494
H	4.468211	1.912449	6.844081
H	4.337707	0.358694	5.994216
H	2.873369	1.291973	6.371854
C	0.554605	1.671738	2.638113
H	0.665415	2.434029	1.874887
H	-0.424916	1.550175	3.078472
C	3.104653	4.307116	4.232703
H	2.054035	4.176193	4.486440
H	3.184210	4.784219	3.257223

C	1.907610	-3.674040	-0.569961
C	1.248638	-4.905137	-0.600434
C	1.950355	-6.059724	-0.901343
C	3.308075	-5.956413	-1.170155
C	3.984764	-4.745425	-1.148020
C	3.274934	-3.595376	-0.844283
H	0.191190	-4.952026	-0.387278
H	1.460780	-7.020061	-0.928002
N	4.062594	-7.182994	-1.490909
H	5.040704	-4.708146	-1.362916
O	3.456755	-8.243756	-1.489358
O	5.253265	-7.072563	-1.740590

27−TS$_{II}$

E = -854.62141846

H = -854.40911335

G = -854.44658741

N_{imag} = 1

O	0.925944	-0.756531	0.960340
C	1.476503	-0.164724	0.012935
C	0.500090	0.401209	-0.991592
C	-0.789049	0.419188	-0.175374
C	-0.585937	-0.685034	0.846913
H	2.559028	-0.120723	-0.004813
H	0.826977	1.363652	-1.378989
H	-0.891070	1.383420	0.318644
H	-1.655176	0.245256	-0.807670
H	-0.924888	-0.473678	1.853991

H	-0.879891	-1.681100	0.528999
O	0.333738	-0.536399	-2.072169
C	1.436159	-0.847507	-2.796583
H	3.192317	-1.782521	-4.543999
O	2.541712	-0.497106	-2.458827
C	1.119700	-1.656243	-3.997012
C	-0.192014	-2.009634	-4.321527
C	-0.442754	-2.768919	-5.452585
C	0.630187	-3.159072	-6.240611
C	1.941707	-2.818982	-5.939924
C	2.182996	-2.061882	-4.806671
H	-1.013260	-1.695297	-3.696710
H	-1.447456	-3.053574	-5.721140
N	0.366085	-3.969003	-7.444931
H	2.748673	-3.141703	-6.578124
O	-0.791763	-4.275768	-7.684409
O	1.319109	-4.289685	-8.138293

27–TS$_{III}$

E = -854.62111833

H = -854.40959870

G = -854.44641203

$\boldsymbol{N_{imag}}$ = 1

O	0.611313	-0.683708	1.205427
C	1.266495	-0.470980	0.172112
C	0.502085	0.179056	-0.938606
C	-0.884447	0.410580	-0.321480
C	-0.816944	-0.200708	1.075024

H	2.302586	-0.798009	0.143514
H	1.009226	1.090272	-1.257572
H	-1.097549	1.473702	-0.269942
H	-1.643401	-0.075329	-0.928603
H	-0.938220	0.499420	1.894311
H	-1.410235	-1.094834	1.234212
O	0.429456	-0.753164	-2.020262
C	1.443304	-0.720697	-2.933996
H	3.098416	-1.186693	-4.947020
O	2.421825	-0.034696	-2.786283
C	1.180363	-1.618198	-4.084784
C	-0.019085	-2.322353	-4.211461
C	-0.225147	-3.144128	-5.307279
C	0.779648	-3.246679	-6.258256
C	1.979790	-2.557217	-6.154857
C	2.176024	-1.737209	-5.056982
H	-0.789485	-2.229087	-3.462334
H	-1.144258	-3.695457	-5.425101
N	0.562598	-4.124062	-7.423880
H	2.736924	-2.663228	-6.915239
O	-0.497191	-4.727772	-7.494874
O	1.453574	-4.201182	-8.255919

Supplementary Figure S2. Conformational energy landscape (CEL) maps of the C-2-methoxy furanosyl oxocarbenium ion **S6$_{oxo}$** in which the local minima identified are shown with their respective energy. Energies of all conformations in the CEL are computed at SMD(dichloromethane)-revDSD-PBEP86-D4-def2TZVPP//PCM(dichloromethane)-B3LYP-D3BJ-def2TZVPP and expressed as relative Gibbs free energy (T = 228.15 K) in kcal mol^{-1}.

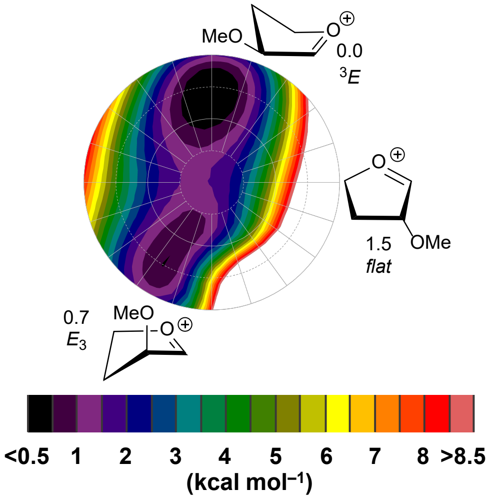

X. References

[1] D. A. Otte, D. E. Borchmann, C. Lin, M. Weck, K. A. Woerpel, *Org. Lett.* **2014**, *16*, 1566–1569.
[2] Y. Chun, W. A. Remmerswaal, J. D. C. Codée, K. A. Woerpel, *Chem. - Eur. J.* **2023**, *29*, e202301894.
[3] a) C. A. G. Haasnoot, *J. Am. Chem. Soc.* **1993**, *115*, 1460–1468; b) N. D. Bartolo, K. M. Demkiw, E. M. Valentin, C. T. Hu, A. A. Arabi, K. A. Woerpel, *J. Org. Chem.* **2021**, *86*, 7203–7217; c) H. J. Schneider, V. Hoppen, *J. Org. Chem.* **1978**, *43*, 3866–3873; d) C. H. Bushweller, J. W. O'Neil, *J. Org. Chem.* **1970**, *35*, 276–278.
[4] C. H. Larsen, B. H. Ridgway, J. T. Shaw, D. M. Smith, K. A. Woerpel, *J. Am. Chem. Soc.* **2005**, *127*, 10879–10884.
[5] a) F. Neese, *WIREs Comput. Mol. Sci.* **2022**, e1606; b) F. Neese, *WIREs Comput. Mol. Sci.* **2011**, *2*, 73–78.
[6] a) G. Santra, M. Cho, J. M. L. Martin, *J. Phys. Chem.* **2021**, *125*, 4614–4627; b) G. Santra, N. Sylvetsky, J. M. Martin, *J. Phys. Chem.* **2019**, *123*, 5129–5143; c) E. Caldeweyher, C. Bannwarth, S. Grimme, *J. Chem. Phys.* **2017**, *147*, 034112; d) A. D. Becke, *J. Chem. Phys.* **1993**, 5648–5652; e) S. H. Vosko, L. Wilk, M. Nusair, *Can. J. Phys.* **1980**, *58*, 1200–1211; f) S. Grimme, S. Ehrlich, L. Goerigk, *J. Comput. Chem.* **2011**, *32*, 1456–1465; g) J. Grimme, S. Antony, H. Ehrlich, J. Krieg, *J. Chem. Phys.* **2010**, *143*, 154104; h) F. Weigend, *Phys. Chem. Chem. Phys.* **2006**, *8*, 1057–1065; i) R. Weigend, P. Ahlrichs, *Phys. Chem. Chem. Phys.* **2005**, *7*, 3297–3305.
[7] T. Hansen, L. Lebedel, W. A. Remmerswaal, S. van der Vorm, D. P. A. Wander, M. Somers, H. S. Overkleeft, D. V. Filippov, J. Désiré, A. Mingot, Y. Bleriot, G. A. van der Marel, S. Thibaudeau, J. D. C. Codée, *ACS Cent. Sci.* **2019**, *5*, 781–788.
[8] OriginPro, 9.0.0., OriginLab Corporation, Northampton, MA, USA.

XI. Selected ^{1}H and ^{13}C{^{1}H} NMR Spectra

The $^{13}C\{^1H\}$ NMR Spectrum of Compounds *trans*-**21a** and *cis*-**21a**

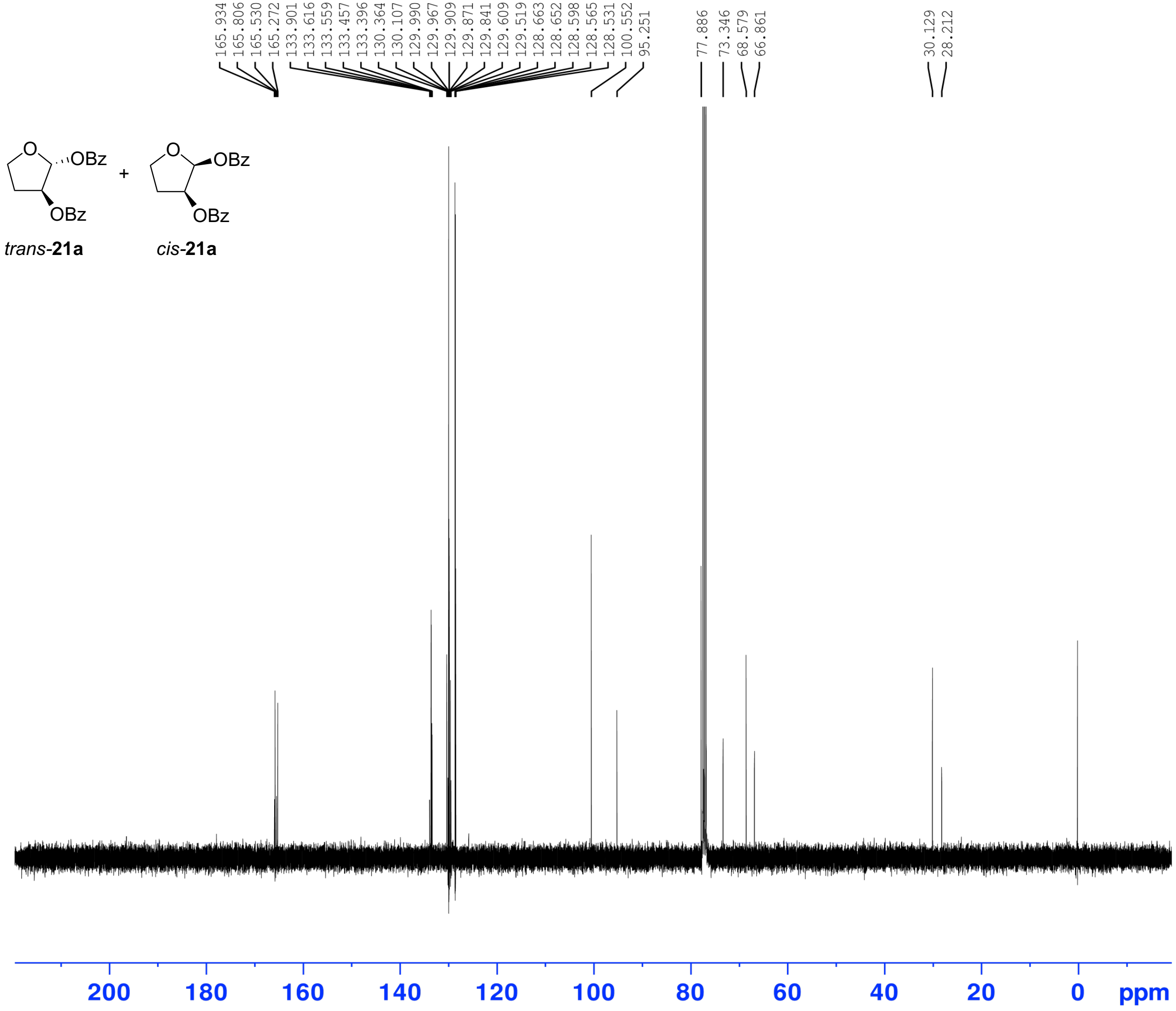

trans-**21a** *cis*-**21a**

```
Current Data Parameters
NAME       YC-3-124-col3_2
EXPNO                    2
PROCNO                   1

F2 - Acquisition Parameters
Date_             20230216
Time                  7.26 h
INSTRUM              spect
PROBHD       Z150354_0001 (
PULPROG            zgpg30
TD                   65356
SOLVENT              CDCl3
NS                     128
DS                       4
SWH              24038.461 Hz
FIDRES            0.735616 Hz
AQ               1.3594048 sec
RG                   51.78
DW                  20.800 usec
DE                   25.00 usec
TE                   298.0 K
D1              2.00000000 sec
D11             0.03000000 sec
TD0                      1
SFO1           100.6655806 MHz
NUC1                   13C
P1                   10.00 usec
PLW1           17.99699974 W
SFO2           400.3016012 MHz
NUC2                    1H
CPDPRG[2           waltz16
PCPD2                80.00 usec
PLW2            4.64209986 W
PLW12           0.10445000 W
PLW13           0.05245300 W

F2 - Processing parameters
SI                  131072
SF             100.6554999 MHz
WDW                     EM
SSB                      0
LB                       0 Hz
GB                       0
PC                    1.40
```

The ^{1}H NMR Spectrum of Compounds *trans*-**21a** and *cis*-**21a**

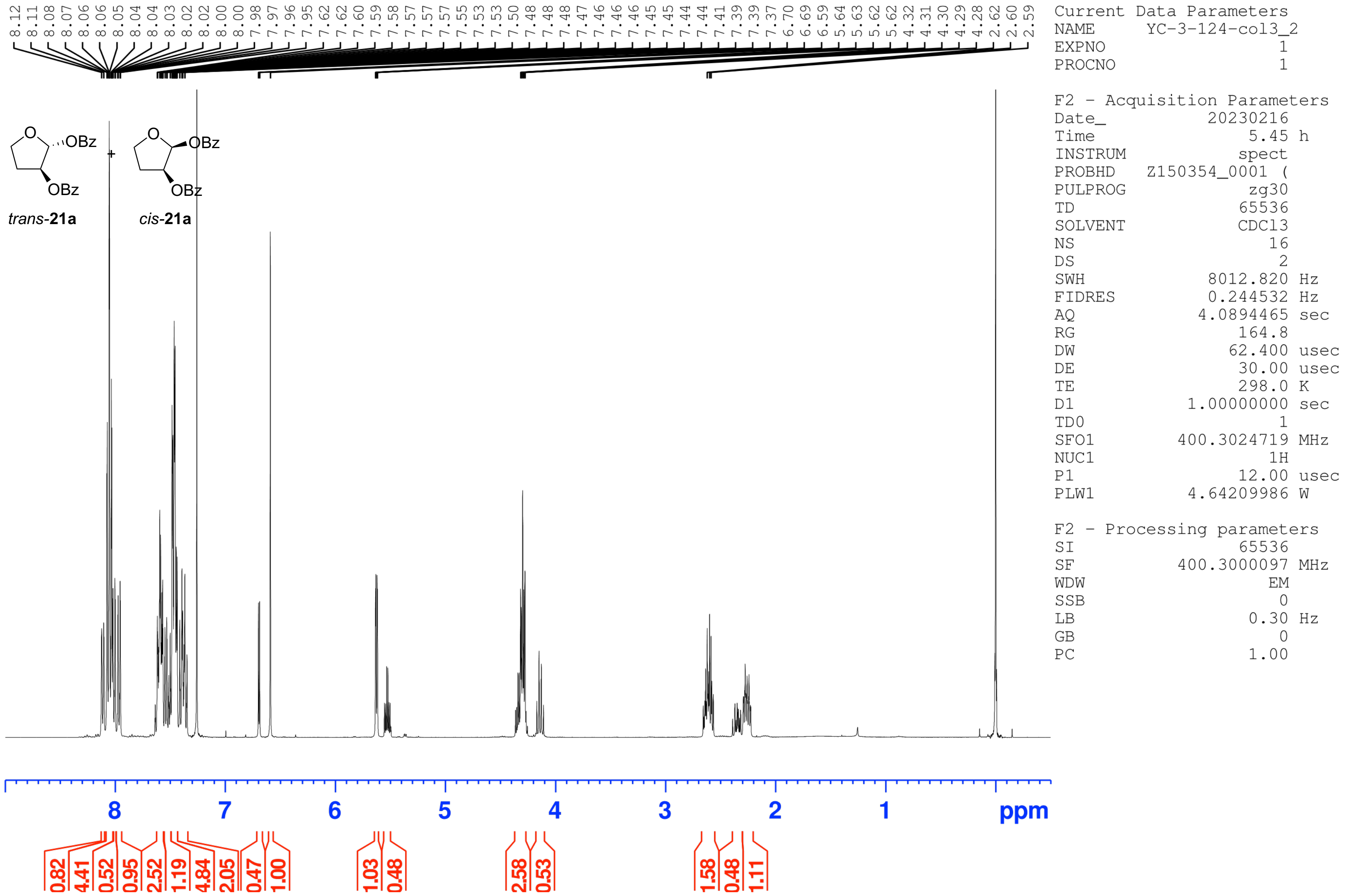

The ^{1}H NMR Spectrum of Compounds *trans*-**21b** and *cis*-**21b**

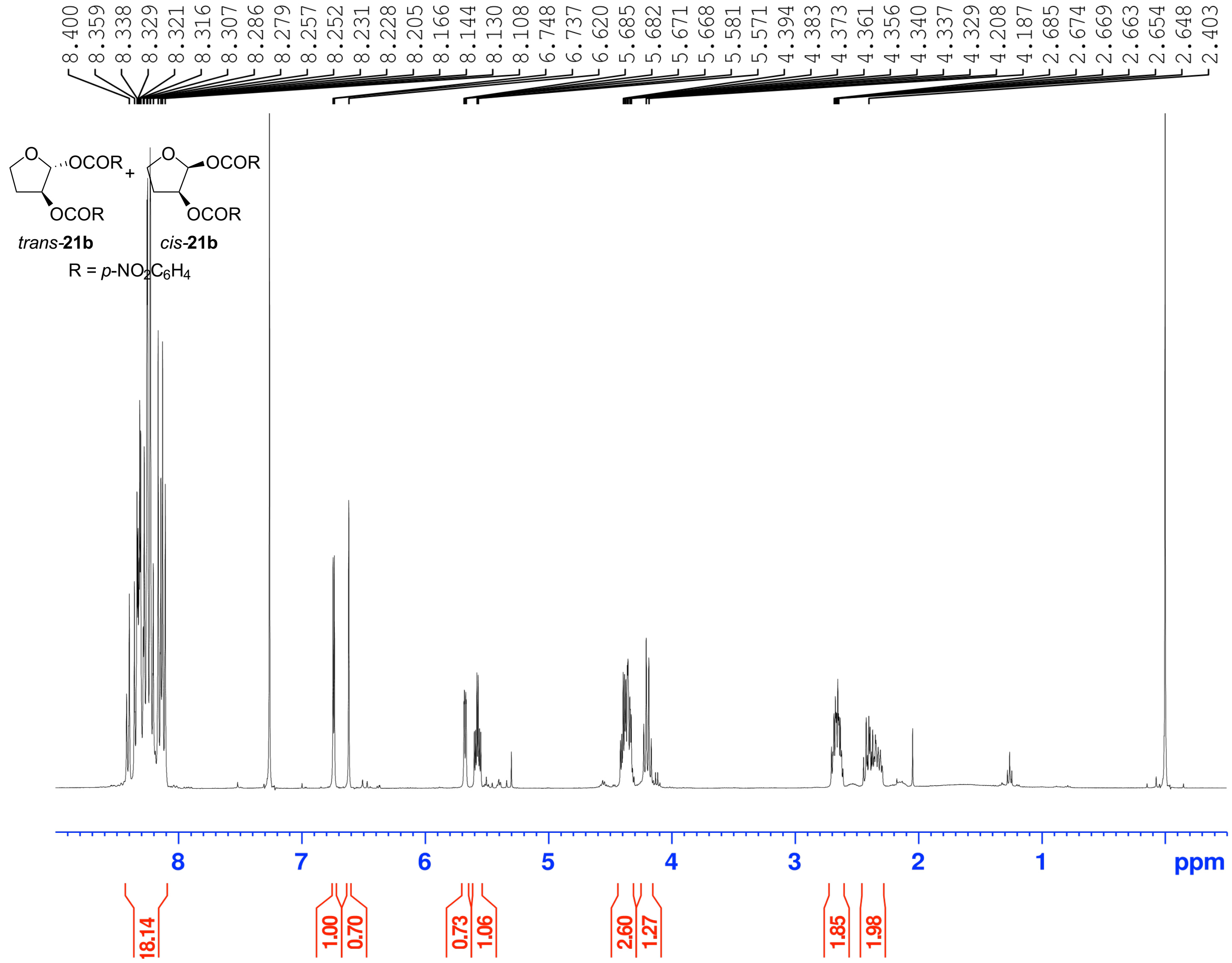

Current Data Parameters
NAME YC-3-125-COLUMN
EXPNO 1
PROCNO 1

F2 - Acquisition Parameters
Date_ 20230208
Time 0.19 h
INSTRUM spect
PROBHD Z150354_0001 (
PULPROG zg30
TD 65536
SOLVENT CDCl3
NS 16
DS 2
SWH 8012.820 Hz
FIDRES 0.244532 Hz
AQ 4.0894465 sec
RG 207.85
DW 62.400 usec
DE 30.00 usec
TE 298.0 K
D1 1.00000000 sec
TD0 1
SFO1 400.3024719 MHz
NUC1 1H
P1 12.00 usec
PLW1 4.64209986 W

F2 - Processing parameters
SI 65536
SF 400.3000079 MHz
WDW EM
SSB 0
LB 0.30 Hz
GB 0
PC 1.00

The $^{13}C\{^{1}H\}$ NMR Spectrum of Compounds *trans*-**21b** and *cis*-**21b**

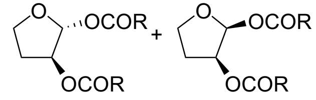

trans-**21b** *cis*-**21b**

R = *p*-$NO_2C_6H_4$

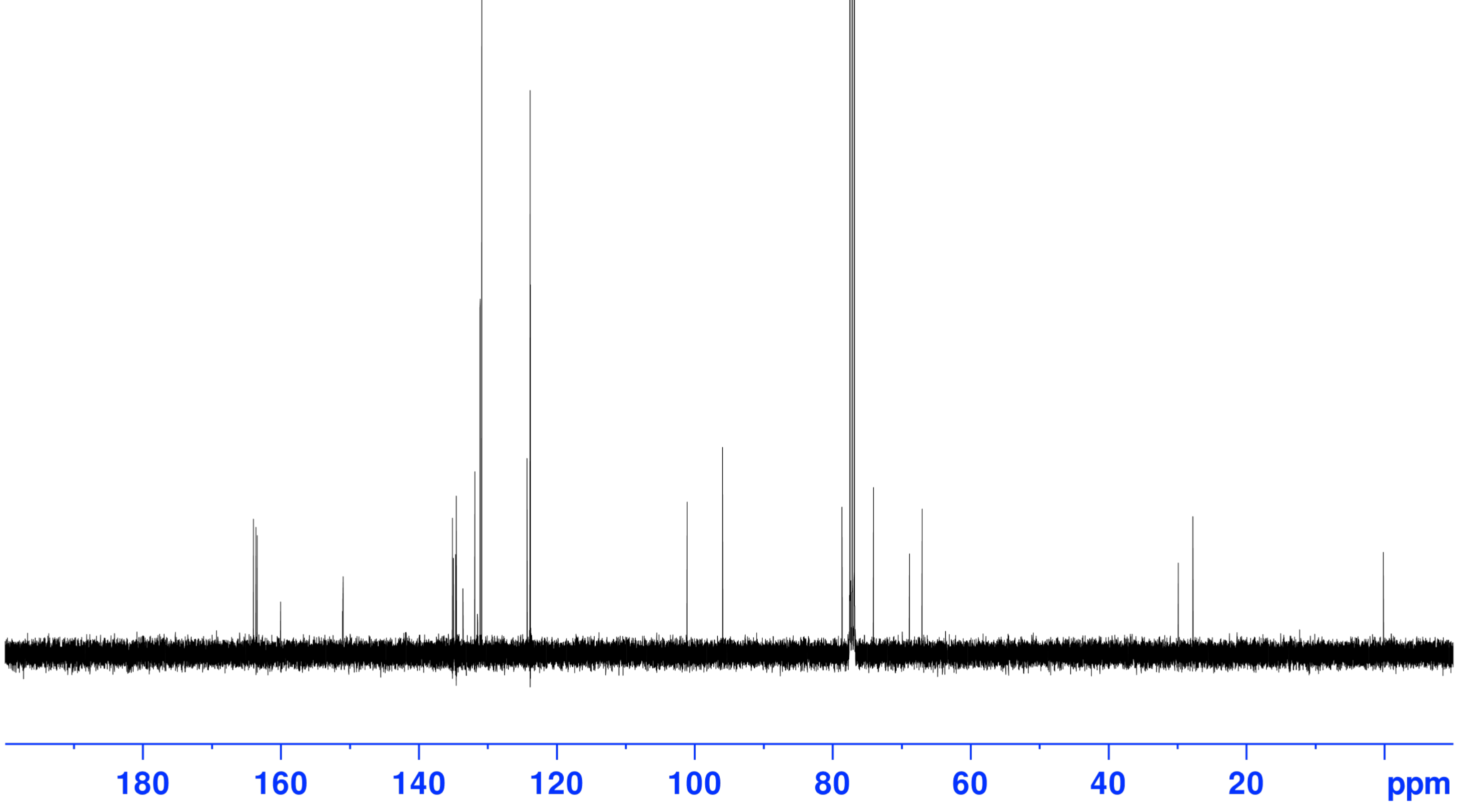

Current Data Parameters
NAME YC-3-125-COLUMN
EXPNO 2
PROCNO 1

F2 - Acquisition Parameters
Date_ 20230208
Time 1.40 h
INSTRUM spect
PROBHD Z150354_0001 (
PULPROG zgpg30
TD 65356
SOLVENT CDCl3
NS 128
DS 4
SWH 24038.461 Hz
FIDRES 0.735616 Hz
AQ 1.3594048 sec
RG 25.77
DW 20.800 usec
DE 25.00 usec
TE 298.0 K
D1 2.00000000 sec
D11 0.03000000 sec
TD0 1
SFO1 100.6655806 MHz
NUC1 13C
P1 10.00 usec
PLW1 17.99699974 W
SFO2 400.3016012 MHz
NUC2 1H
CPDPRG[2 waltz16
PCPD2 80.00 usec
PLW2 4.64209986 W
PLW12 0.10445000 W
PLW13 0.05245300 W

F2 - Processing parameters
SI 131072
SF 100.6555017 MHz
WDW EM
SSB 0
LB 0 Hz
GB 0
PC 1.40

The $^{13}C\{^1H\}$ NMR Spectrum of Compound *trans*-**9a**

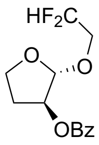

trans-**9a**

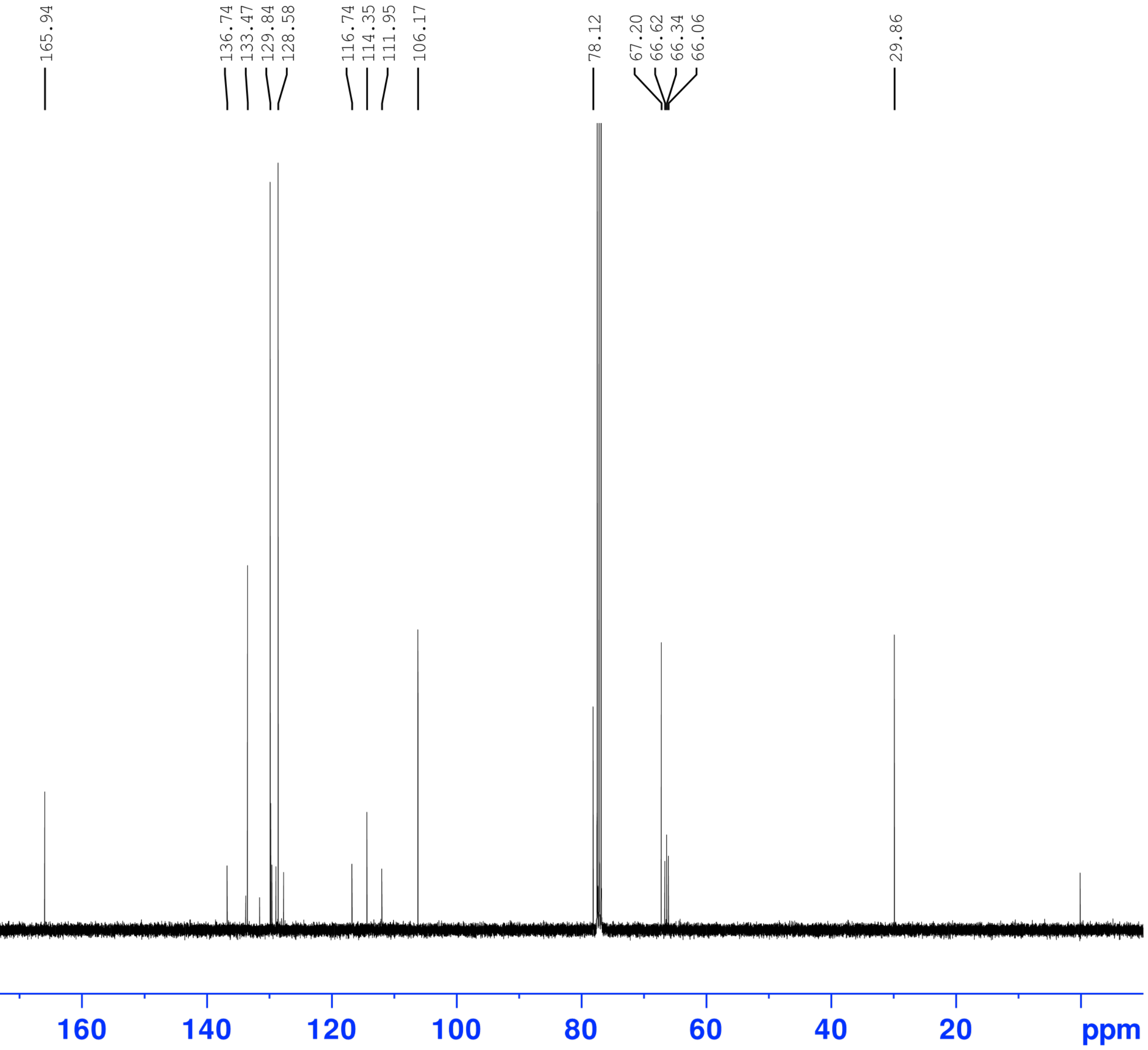

Current Data Parameters
NAME YC-4-173-Fr16-35
EXPNO 2
PROCNO 1

F2 - Acquisition Parameters
Date_ 20240509
Time 0.33 h
INSTRUM spect
PROBHD Z150354_0001 (
PULPROG zgpg30
TD 65356
SOLVENT CDCl3
NS 128
DS 4
SWH 24038.461 Hz
FIDRES 0.735616 Hz
AQ 1.3594048 sec
RG 36.68
DW 20.800 usec
DE 25.00 usec
TE 298.0 K
D1 2.00000000 sec
D11 0.03000000 sec
TD0 1
SFO1 100.6655806 MHz
NUC1 13C
P1 10.00 usec
PLW1 18.70700073 W
SFO2 400.3016012 MHz
NUC2 1H
CPDPRG[2 waltz16
PCPD2 80.00 usec
PLW2 4.64209986 W
PLW12 0.10445000 W
PLW13 0.05245300 W

F2 - Processing parameters
SI 131072
SF 100.6555024 MHz
WDW EM
SSB 0
LB 0 Hz
GB 0
PC 1.40

The $^{13}C\{^{1}H\}$ NMR Spectrum of Compound *cis*-**9a**

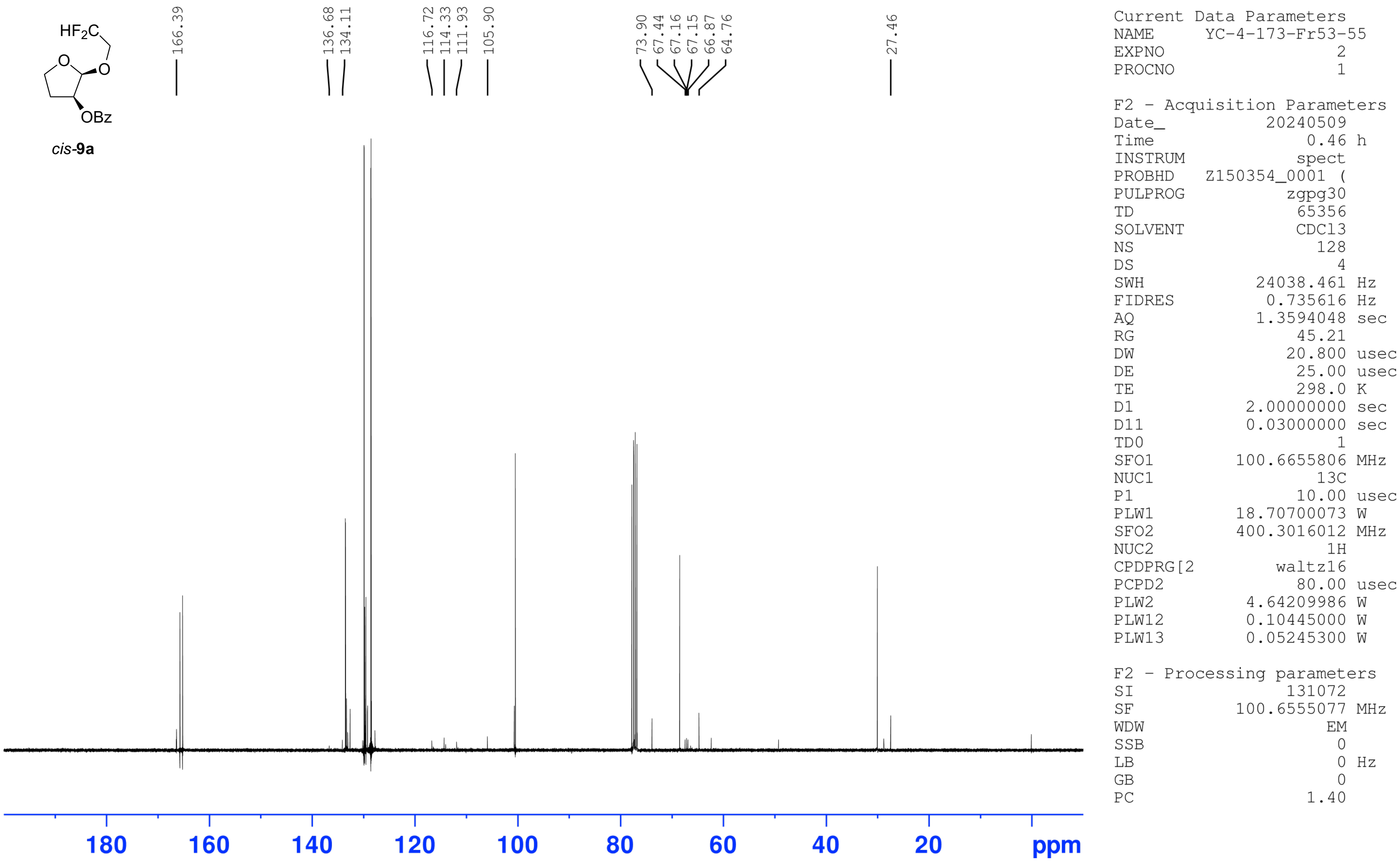

The ^{1}H NMR Spectrum of Compound *trans*-**9a**

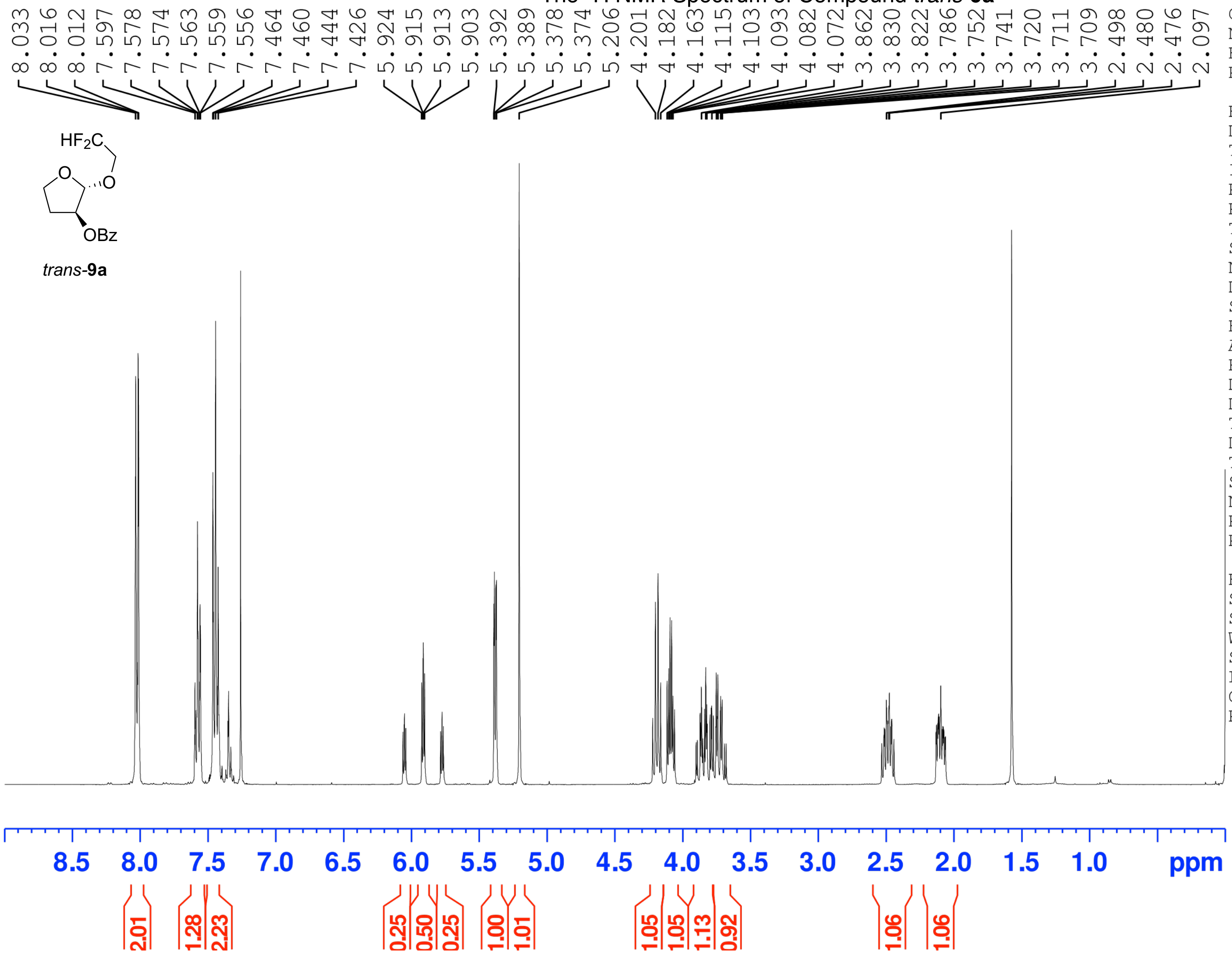

Current Data Parameters
NAME YC-4-173-Fr16-35_2D
EXPNO 2
PROCNO 1

F2 - Acquisition Parameters
Date_ 20240509
Time 2.26 h
INSTRUM spect
PROBHD Z150354_0001 (
PULPROG zg30
TD 65536
SOLVENT CDCl3
NS 16
DS 2
SWH 8012.820 Hz
FIDRES 0.244532 Hz
AQ 4.0894465 sec
RG 117.47
DW 62.400 usec
DE 30.00 usec
TE 298.0 K
D1 1.00000000 sec
TD0 1
SFO1 400.3024719 MHz
NUC1 1H
P1 12.00 usec
PLW1 4.64209986 W

F2 - Processing parameters
SI 65536
SF 400.3000094 MHz
WDW EM
SSB 0
LB 0.30 Hz
GB 0
PC 1.00

The ¹H NMR Spectrum of Compounds *trans*-**21a** and *cis*-**21a**

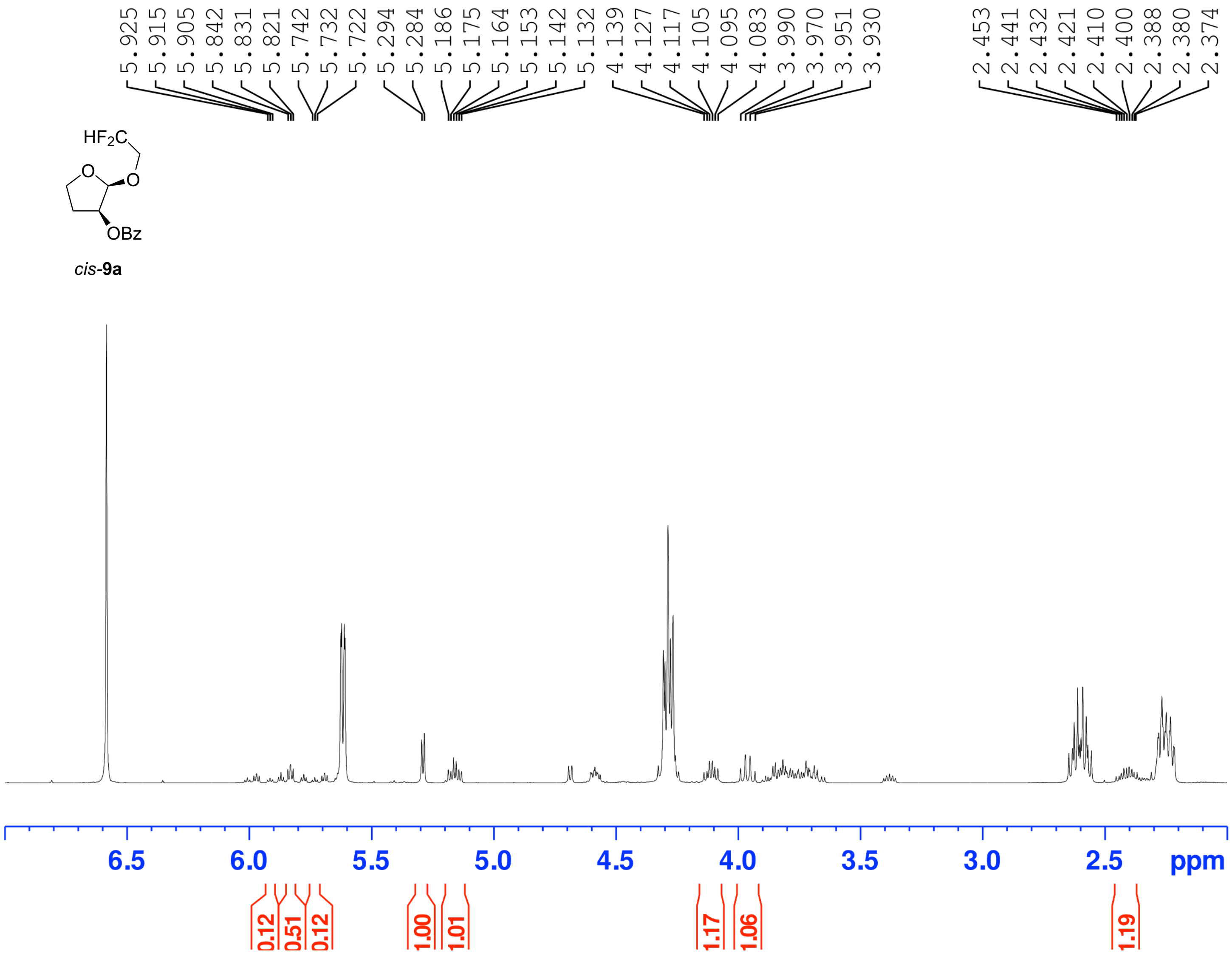

cis-**9a**

Current Data Parameters
NAME YC-4-173-Fr53-55
EXPNO 1
PROCNO 1

F2 - Acquisition Parameters
Date_ 20240509
Time 0.22 h
INSTRUM spect
PROBHD Z150354_0001 (
PULPROG zg30
TD 65536
SOLVENT CDCl3
NS 16
DS 2
SWH 8012.820 Hz
FIDRES 0.244532 Hz
AQ 4.0894465 sec
RG 58.09
DW 62.400 usec
DE 30.00 usec
TE 298.0 K
D1 1.00000000 sec
TD0 1
SFO1 400.3024719 MHz
NUC1 1H
P1 12.00 usec
PLW1 4.64209986 W

F2 - Processing parameters
SI 65536
SF 400.3000129 MHz
WDW EM
SSB 0
LB 0.30 Hz
GB 0
PC 1.00

The $^{13}C\{^{1}H\}$ NMR Spectrum of Compound *trans*-**10a**

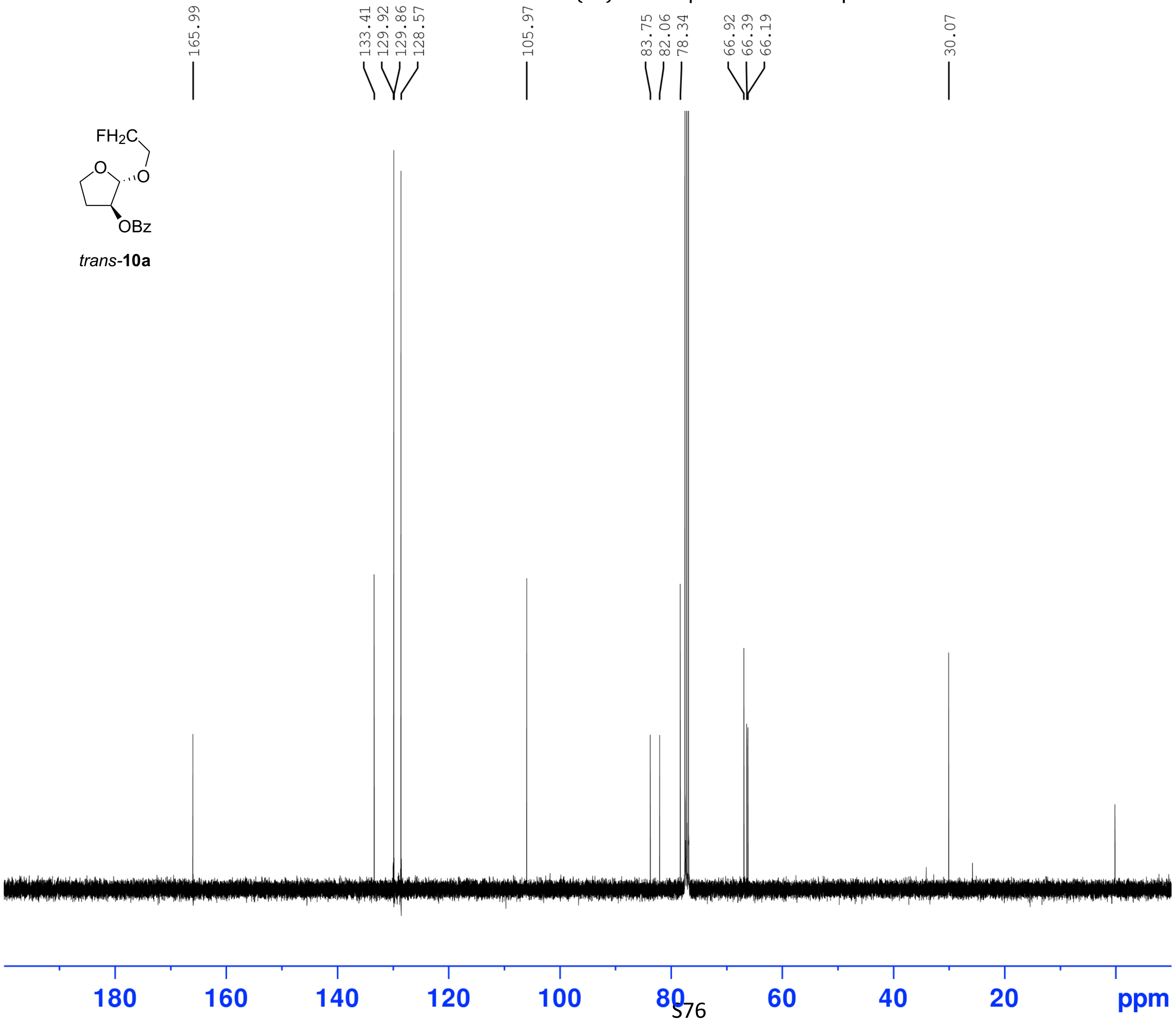

Current Data Parameters
NAME YC-4-138-Fr61.62
EXPNO 2
PROCNO 1

F2 - Acquisition Parameters
Date_ 20240321
Time 3.27 h
INSTRUM spect
PROBHD Z150354_0001 (
PULPROG zgpg30
TD 65356
SOLVENT CDCl3
NS 128
DS 4
SWH 24038.461 Hz
FIDRES 0.735616 Hz
AQ 1.3594048 sec
RG 31.4
DW 20.800 usec
DE 25.00 usec
TE 298.0 K
D1 2.00000000 sec
D11 0.03000000 sec
TD0 1
SFO1 100.6655806 MHz
NUC1 13C
P1 10.00 usec
PLW1 18.70700073 W
SFO2 400.3016012 MHz
NUC2 1H
CPDPRG[2 waltz16
PCPD2 80.00 usec
PLW2 4.64209986 W
PLW12 0.10445000 W
PLW13 0.05245300 W

F2 - Processing parameters
SI 131072
SF 100.6554999 MHz
WDW EM
SSB 0
LB 0 Hz
GB 0
PC 1.40

The ^{1}H NMR Spectrum of Compound *trans*-**10a**

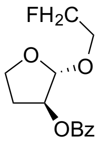

trans-**10a**

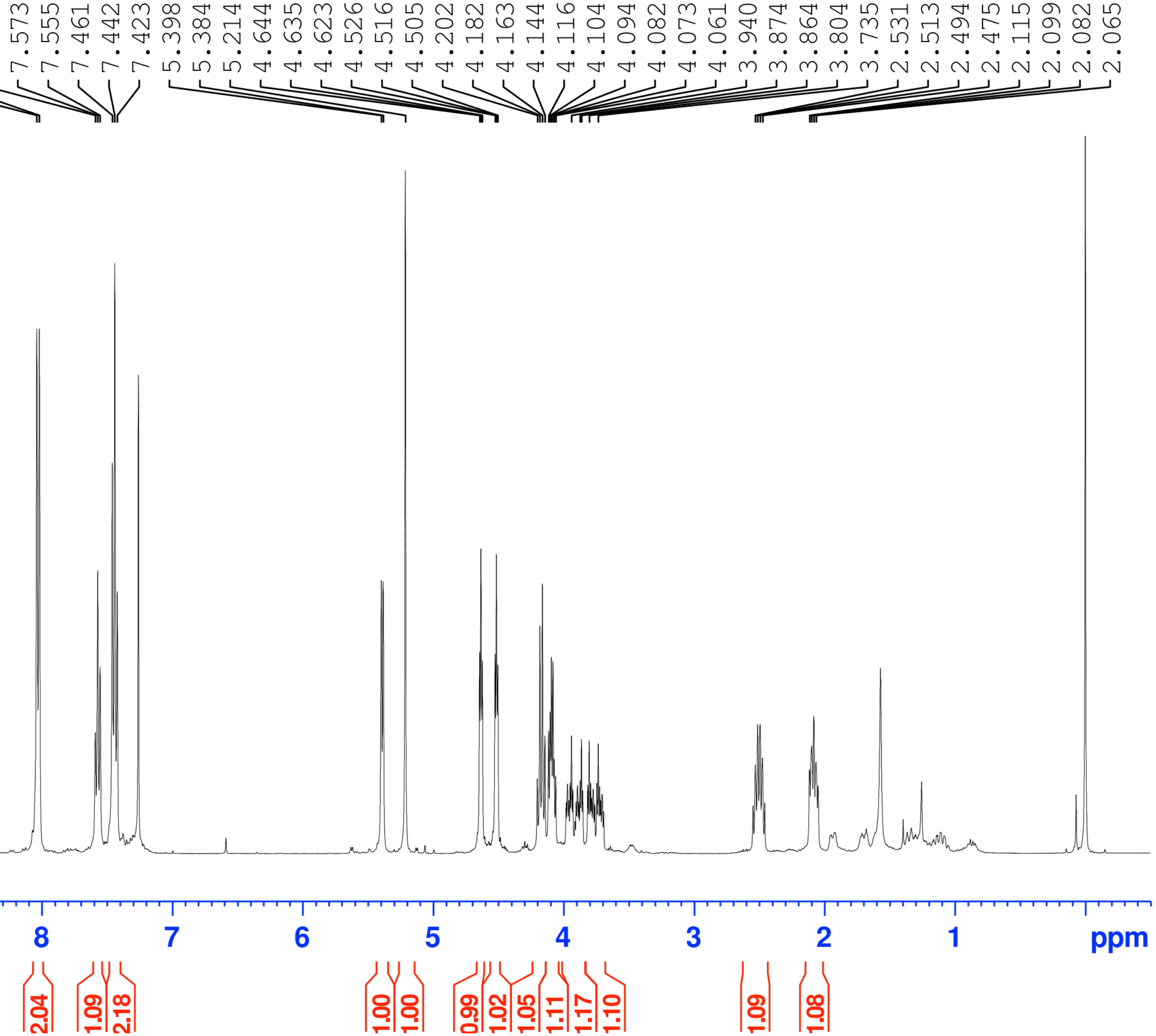

Current Data Parameters
NAME YC-4-138-Fr61.62
EXPNO 1
PROCNO 1

F2 - Acquisition Parameters
Date_ 20240321
Time 0.32 h
INSTRUM spect
PROBHD Z150354_0001 (
PULPROG zg30
TD 65536
SOLVENT CDCl3
NS 16
DS 2
SWH 8012.820 Hz
FIDRES 0.244532 Hz
AQ 4.0894465 sec
RG 125.92
DW 62.400 usec
DE 30.00 usec
TE 298.0 K
D1 1.00000000 sec
TD0 1
SFO1 400.3024719 MHz
NUC1 1H
P1 12.00 usec
PLW1 4.64209986 W

F2 - Processing parameters
SI 65536
SF 400.3000089 MHz
WDW EM
SSB 0
LB 0.30 Hz
GB 0
PC 1.00

The $^{13}C\{^1H\}$ NMR Spectrum of Compound *trans*-**9b**

trans-**9b**

R = *p*-$NO_2C_6H_4$

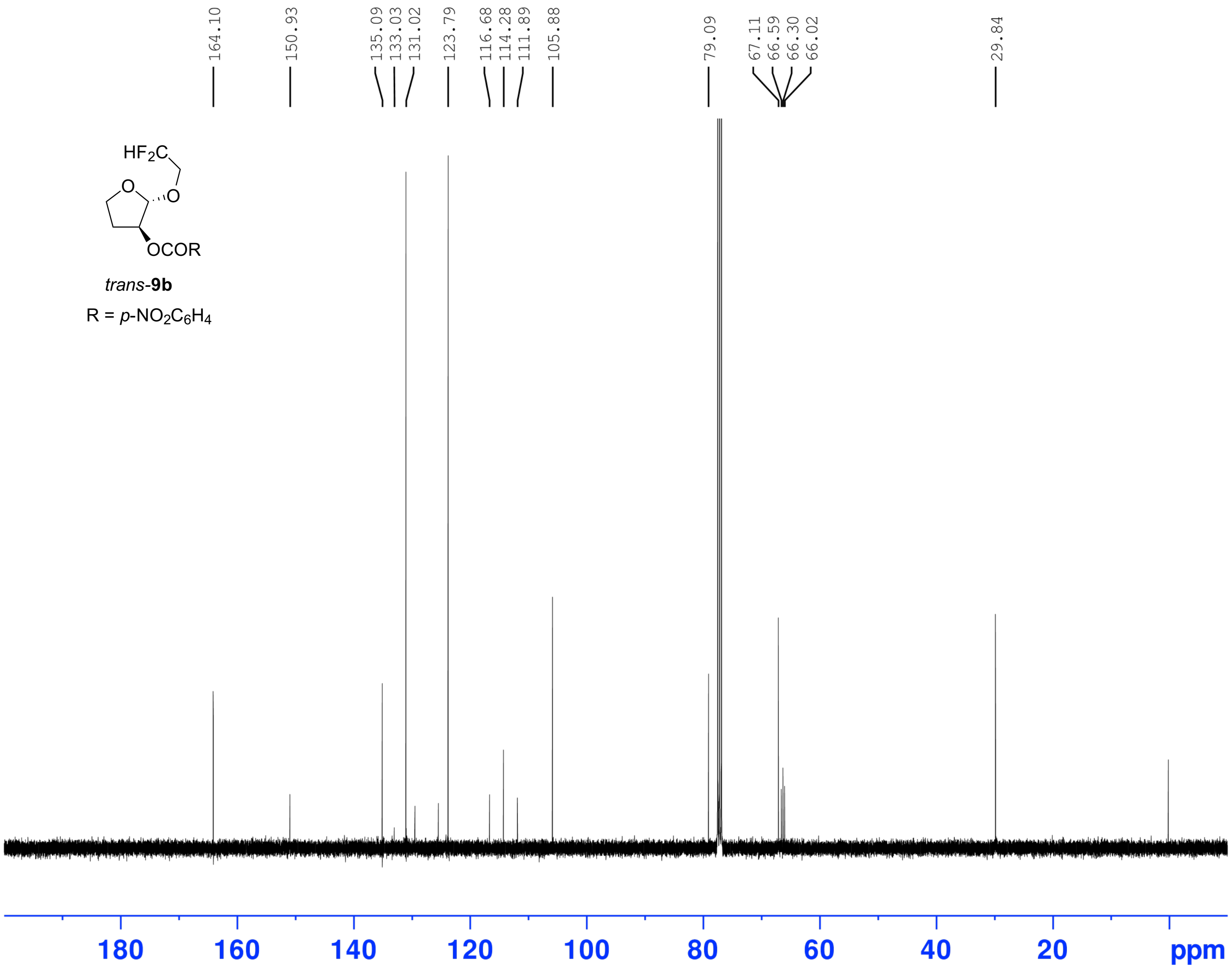

Current Data Parameters

NAME	YC-4-136-Fr31-34
EXPNO	3
PROCNO	1

F2 - Acquisition Parameters

Date_	20240305
Time	6.37 h
INSTRUM	spect
PROBHD	Z150354_0001 (
PULPROG	zgpg30
TD	65356
SOLVENT	CDCl3
NS	128
DS	4
SWH	24038.461 Hz
FIDRES	0.735616 Hz
AQ	1.3594048 sec
RG	29.21
DW	20.800 usec
DE	25.00 usec
TE	298.0 K
D1	2.00000000 sec
D11	0.03000000 sec
TD0	1
SFO1	100.6655806 MHz
NUC1	13C
P1	10.00 usec
PLW1	18.70700073 W
SFO2	400.3016012 MHz
NUC2	1H
CPDPRG[2	waltz16
PCPD2	80.00 usec
PLW2	4.64209986 W
PLW12	0.10445000 W
PLW13	0.05245300 W

F2 - Processing parameters

SI	131072
SF	100.6554989 MHz
WDW	EM
SSB	0
LB	0 Hz
GB	0
PC	1.40

The $^{13}C\{^1H\}$ NMR Spectrum of Compounds *cis*-**10a** and **16a**

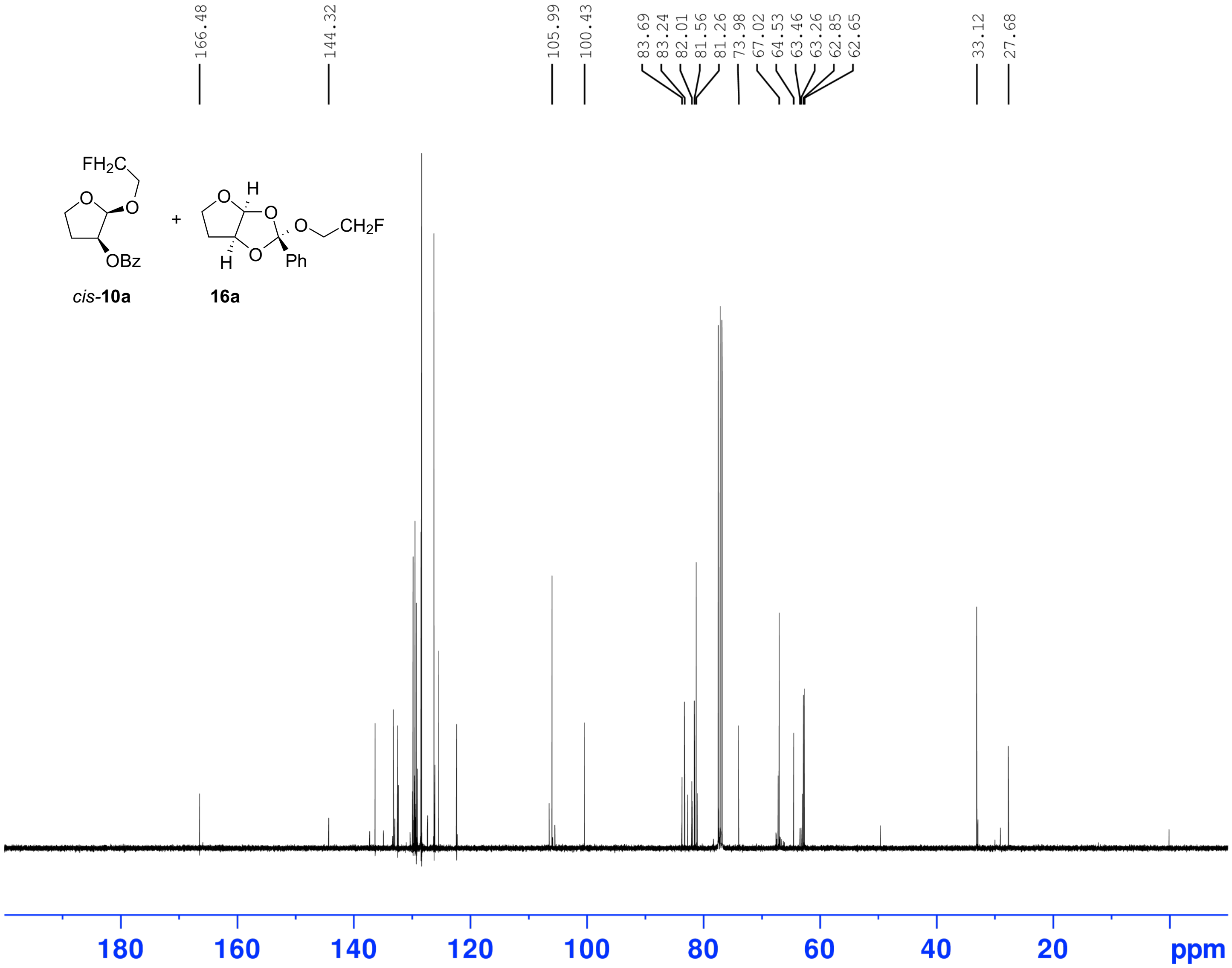

Current Data Parameters
NAME YC-4-180-Fr43-63
EXPNO 2
PROCNO 1

F2 - Acquisition Parameters
Date_ 20240511
Time 1.30 h
INSTRUM spect
PROBHD Z150354_0001 (
PULPROG zgpg30
TD 65356
SOLVENT CDCl3
NS 128
DS 4
SWH 24038.461 Hz
FIDRES 0.735616 Hz
AQ 1.3594048 sec
RG 58.09
DW 20.800 usec
DE 25.00 usec
TE 298.0 K
D1 2.00000000 sec
D11 0.03000000 sec
TD0 1
SFO1 100.6655806 MHz
NUC1 13C
P1 10.00 usec
PLW1 18.70700073 W
SFO2 400.3016012 MHz
NUC2 1H
CPDPRG[2 waltz16
PCPD2 80.00 usec
PLW2 4.64209986 W
PLW12 0.10445000 W
PLW13 0.05245300 W

F2 - Processing parameters
SI 131072
SF 100.6555065 MHz
WDW EM
SSB 0
LB 0 Hz
GB 0
PC 1.40

The ^{1}H NMR Spectrum of Compounds *cis*-**10a** and **16a**

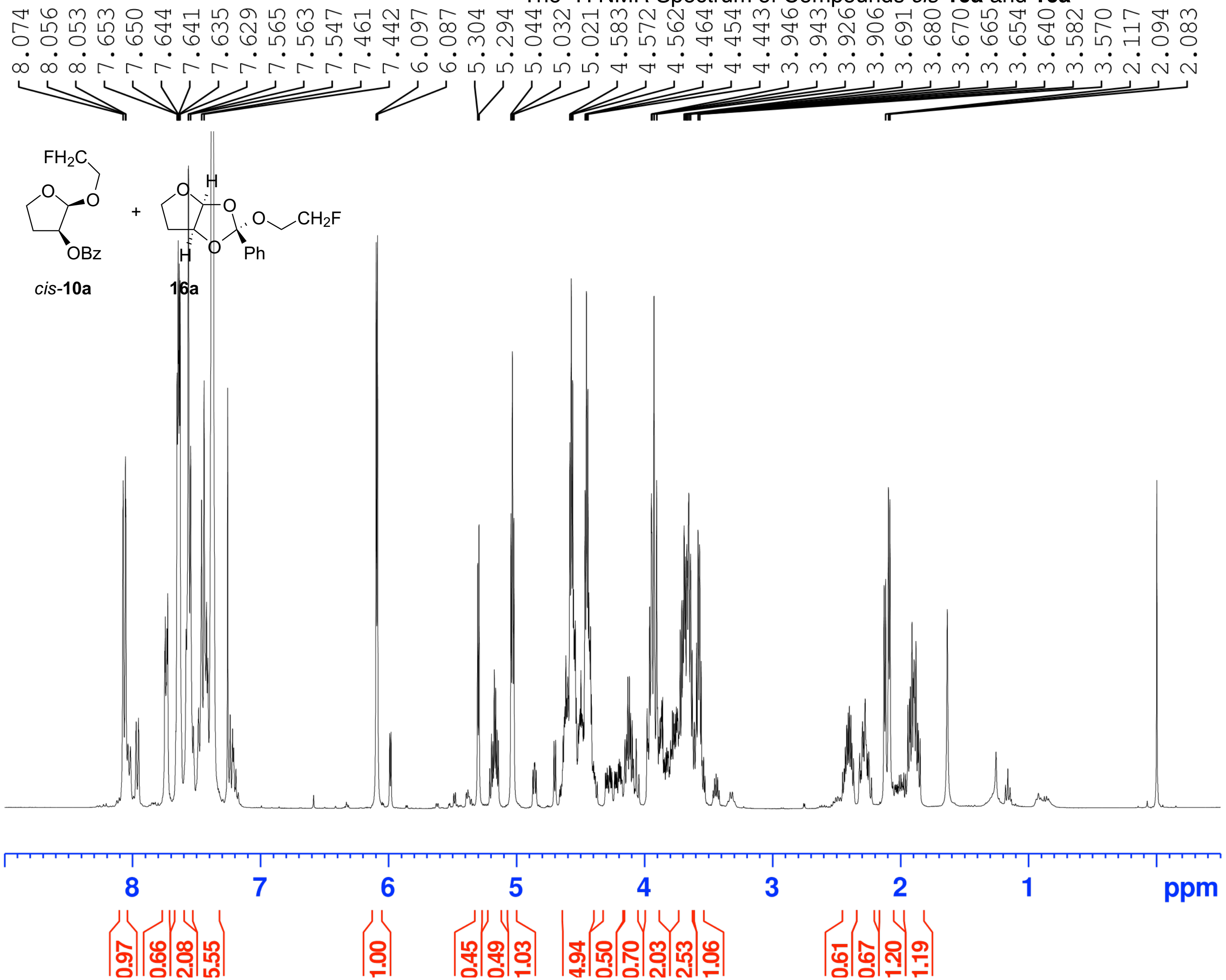


```
Current Data Parameters
NAME          YC-4-180-Fr43-63
EXPNO                        1
PROCNO                       1

F2 - Acquisition Parameters
Date_                 20240511
Time                    0.53 h
INSTRUM                  spect
PROBHD       Z150354_0001 (
PULPROG                   zg30
TD                       65536
SOLVENT                  CDCl3
NS                          16
DS                           2
SWH                   8012.820 Hz
FIDRES                0.244532 Hz
AQ                   4.0894465 sec
RG                       58.09
DW                      62.400 usec
DE                       30.00 usec
TE                       298.0 K
D1                  1.00000000 sec
TD0                          1
SFO1               400.3024719 MHz
NUC1                        1H
P1                       12.00 usec
PLW1                4.64209986 W

F2 - Processing parameters
SI                       65536
SF                 400.3000102 MHz
WDW                         EM
SSB                          0
LB                        0.30 Hz
GB                           0
PC                        1.00
```

The $^{13}C\{^{1}H\}$ NMR Spectrum of Compound *trans*-**11a**

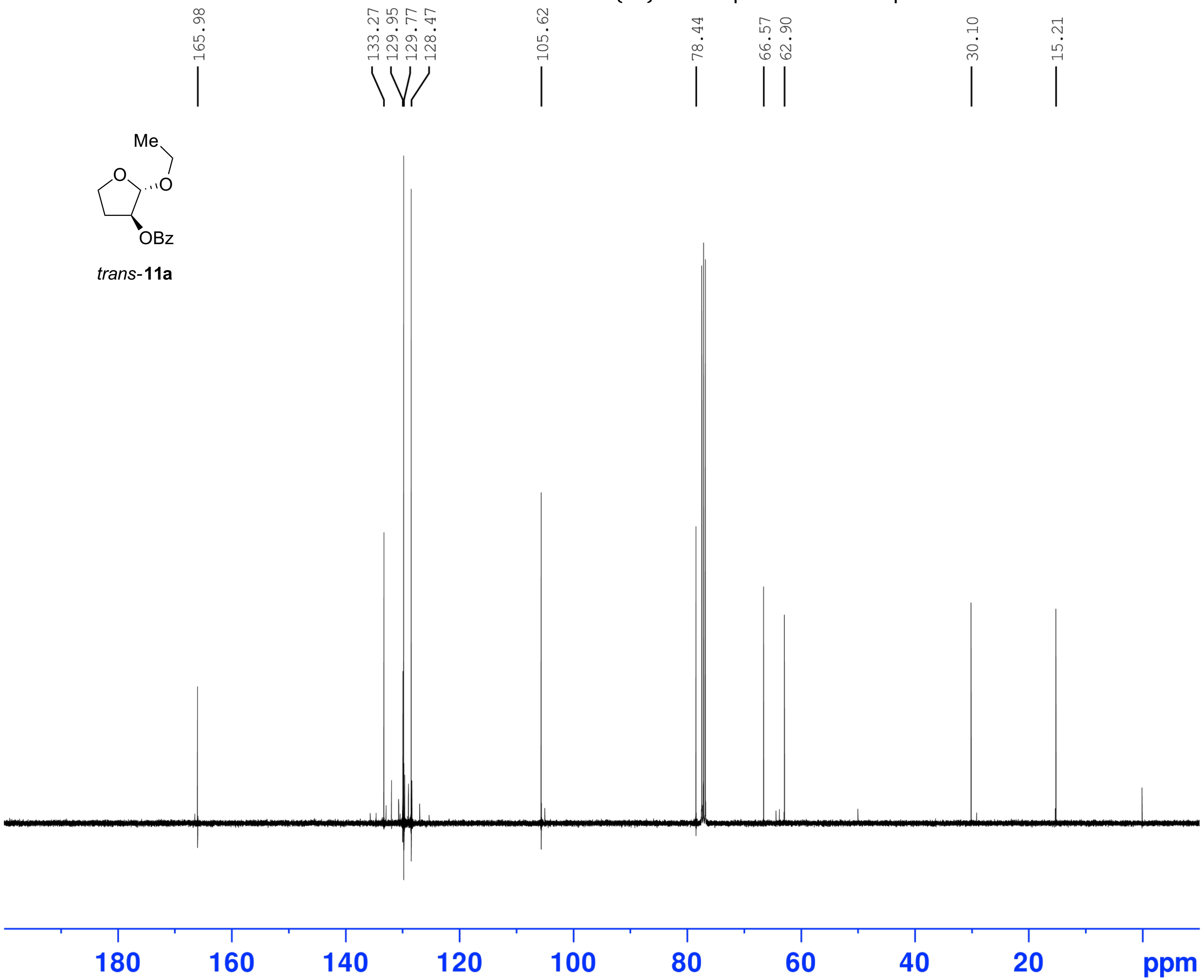


```
Current Data Parameters
NAME          YC-4-175-col2
EXPNO                     2
PROCNO                    1

F2 - Acquisition Parameters
Date_              20240504
Time                   6.19 h
INSTRUM               spect
PROBHD     Z150354_0001 (
PULPROG             zgpg30
TD                    65356
SOLVENT               CDCl3
NS                      128
DS                        4
SWH               24038.461 Hz
FIDRES             0.735616 Hz
AQ                1.3594048 sec
RG                    45.21
DW                   20.800 usec
DE                    25.00 usec
TE                    298.0 K
D1               2.00000000 sec
D11              0.03000000 sec
TD0                       1
SFO1            100.6655806 MHz
NUC1                    13C
P1                    10.00 usec
PLW1            18.70700073 W
SFO2            400.3016012 MHz
NUC2                     1H
CPDPRG[2            waltz16
PCPD2                 80.00 usec
PLW2             4.64209986 W
PLW12            0.10445000 W
PLW13            0.05245300 W

F2 - Processing parameters
SI                   131072
SF              100.6555076 MHz
WDW                      EM
SSB                       0
LB                        0 Hz
GB                        0
PC                     1.40
```

The ¹H NMR Spectrum of Compound *trans*-**11a**

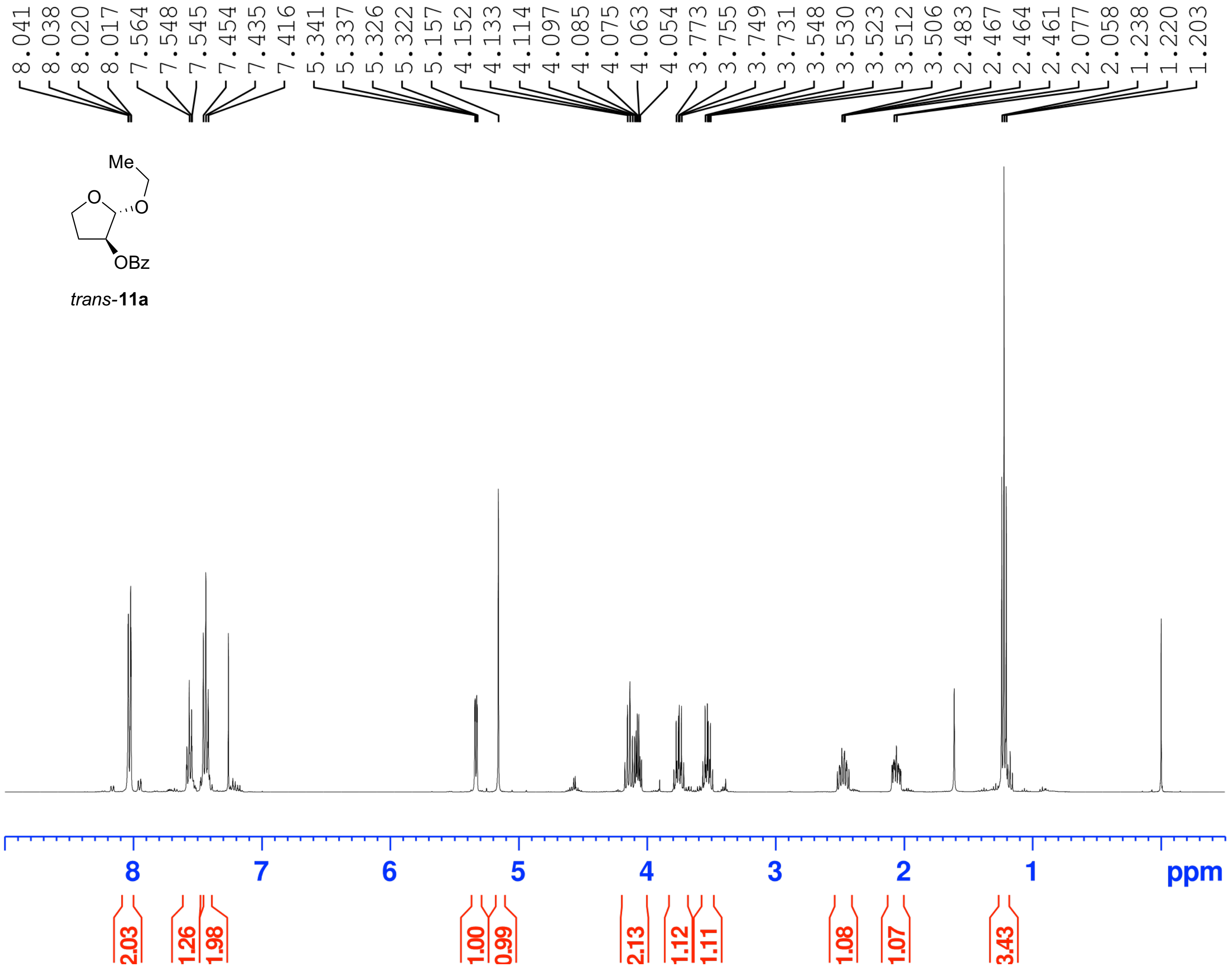

trans-**11a**

Current Data Parameters
NAME YC-4-175-col2
EXPNO 1
PROCNO 1

F2 - Acquisition Parameters
Date_ 20240504
Time 5.28 h
INSTRUM spect
PROBHD Z150354_0001 (
PULPROG zg30
TD 65536
SOLVENT CDCl3
NS 16
DS 2
SWH 8012.820 Hz
FIDRES 0.244532 Hz
AQ 4.0894465 sec
RG 81.45
DW 62.400 usec
DE 30.00 usec
TE 298.0 K
D1 1.00000000 sec
TD0 1
SFO1 400.3024719 MHz
NUC1 1H
P1 12.00 usec
PLW1 4.64209986 W

F2 - Processing parameters
SI 65536
SF 400.3000098 MHz
WDW EM
SSB 0
LB 0.30 Hz
GB 0
PC 1.00

The ¹H NMR Spectrum of Compound *cis*-**11a**

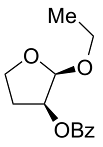

cis-**11a**

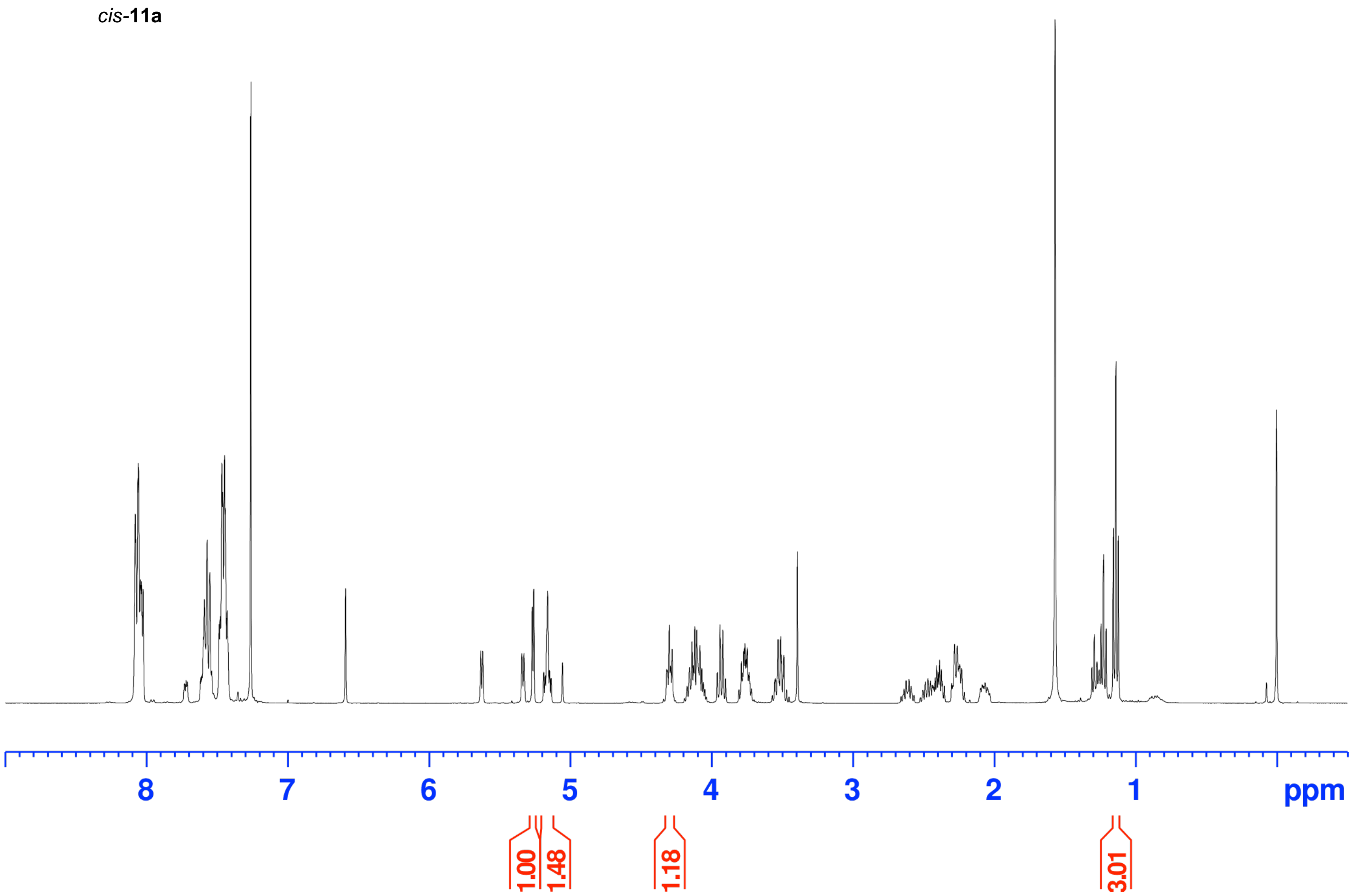

Current Data Parameters
NAME YC-4-176-col1-Fr62-66_2D
EXPNO 1
PROCNO 1

F2 - Acquisition Parameters
Date_ 20240511
Time 1.53 h
INSTRUM spect
PROBHD Z150354_0001 (
PULPROG zg30
TD 65536
SOLVENT CDCl3
NS 16
DS 2
SWH 8012.820 Hz
FIDRES 0.244532 Hz
AQ 4.0894465 sec
RG 164.8
DW 62.400 usec
DE 30.00 usec
TE 298.0 K
D1 1.00000000 sec
TD0 1
SFO1 400.3024719 MHz
NUC1 1H
P1 12.00 usec
PLW1 4.64209986 W

F2 - Processing parameters
SI 65536
SF 400.3000084 MHz
WDW EM
SSB 0
LB 0.30 Hz
GB 0
PC 1.00

The $^{13}C\{^{1}H\}$ NMR Spectrum of Compound *cis*-**11a**

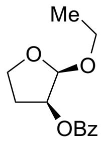

cis-**11a**

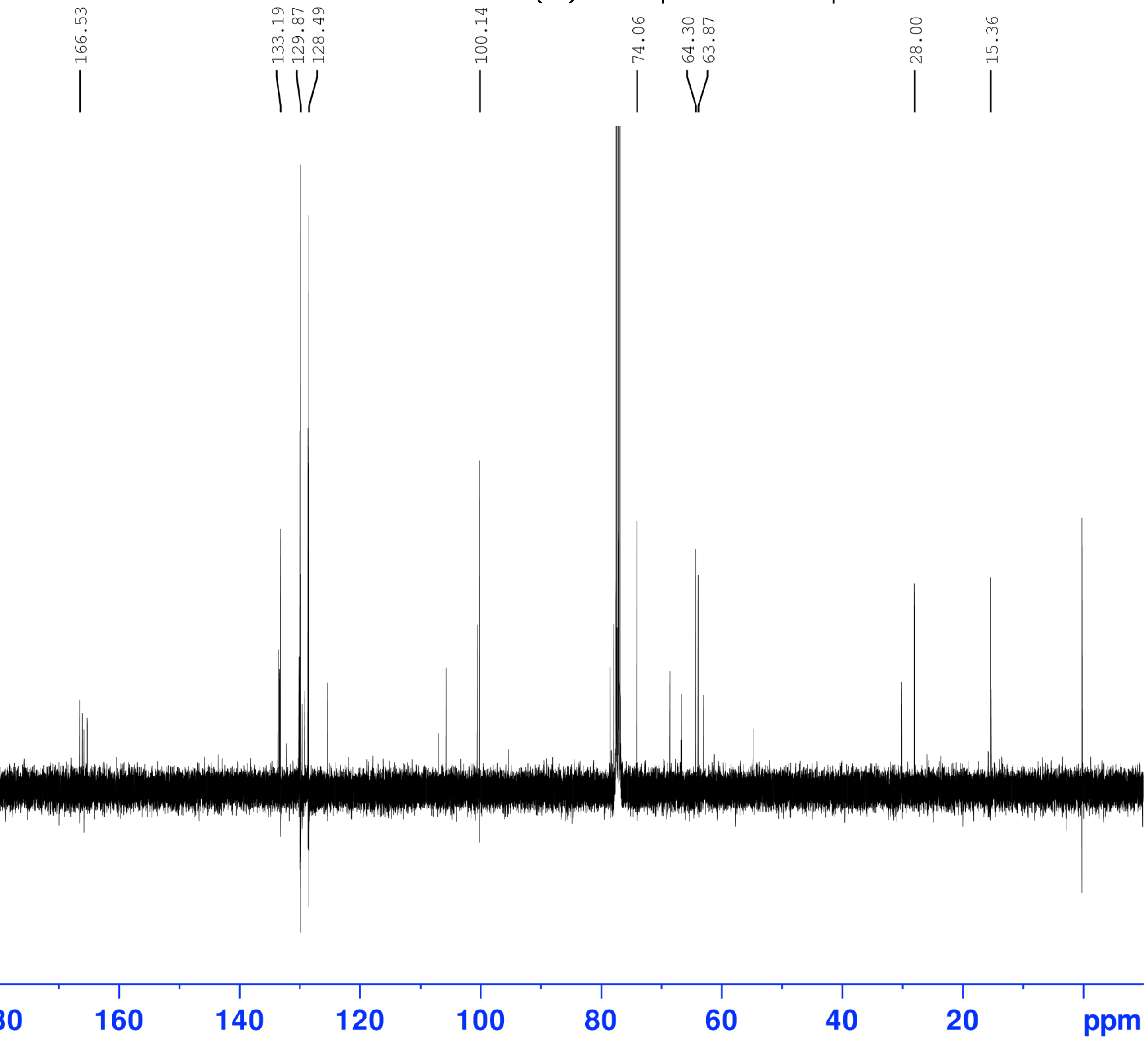


```
Current Data Parameters
NAME     YC-4-176-col1-Fr62-66
EXPNO                   2
PROCNO                  1

F2 - Acquisition Parameters
Date_            20240508
Time                 6.13 h
INSTRUM             spect
PROBHD    Z150354_0001 (
PULPROG           zgpg30
TD                 65356
SOLVENT            CDCl3
NS                   128
DS                     4
SWH           24038.461 Hz
FIDRES         0.735616 Hz
AQ            1.3594048 sec
RG                 58.09
DW                20.800 usec
DE                 25.00 usec
TE                 298.0 K
D1            2.00000000 sec
D11           0.03000000 sec
TD0                    1
SFO1         100.6655806 MHz
NUC1                 13C
P1                 10.00 usec
PLW1         18.70700073 W
SFO2         400.3016012 MHz
NUC2                  1H
CPDPRG[2         waltz16
PCPD2              80.00 usec
PLW2          4.64209986 W
PLW12         0.10445000 W
PLW13         0.05245300 W

F2 - Processing parameters
SI                131072
SF           100.6555016 MHz
WDW                   EM
SSB                    0
LB                     0 Hz
GB                     0
PC                  1.40
```

The $^{13}C\{^{1}H\}$ NMR Spectrum of Compound *trans*-**12a**

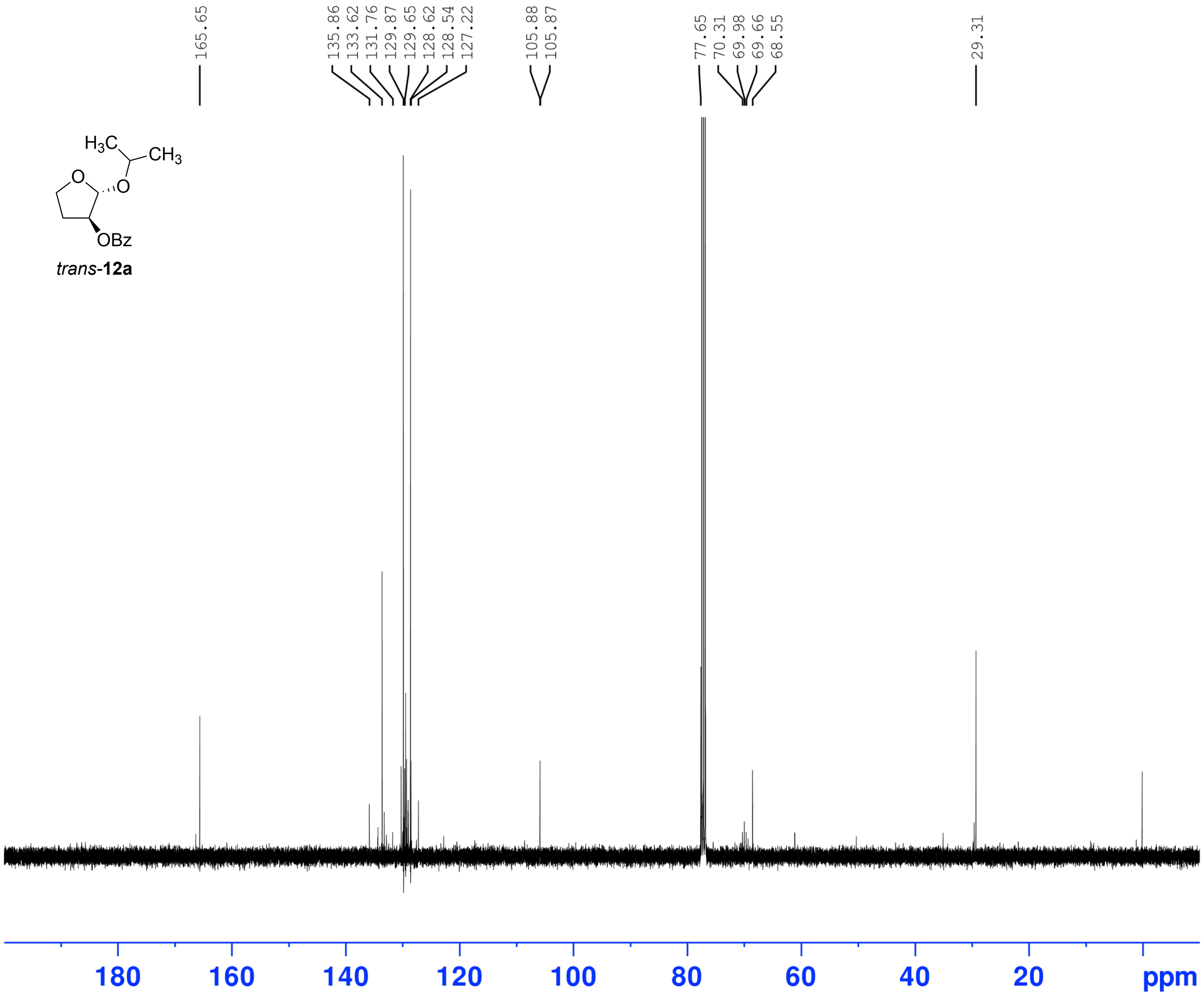

Current Data Parameters
NAME YC-4-149-col2
EXPNO 2
PROCNO 1

F2 - Acquisition Parameters
Date_ 20240330
Time 0.54 h
INSTRUM spect
PROBHD Z150354_0001 (
PULPROG zgpg30
TD 65356
SOLVENT CDCl3
NS 128
DS 4
SWH 24038.461 Hz
FIDRES 0.735616 Hz
AQ 1.3594048 sec
RG 36.68
DW 20.800 usec
DE 25.00 usec
TE 298.0 K
D1 2.00000000 sec
D11 0.03000000 sec
TD0 1
SFO1 100.6655806 MHz
NUC1 13C
P1 10.00 usec
PLW1 18.70700073 W
SFO2 400.3016012 MHz
NUC2 1H
CPDPRG[2 waltz16
PCPD2 80.00 usec
PLW2 4.64209986 W
PLW12 0.10445000 W
PLW13 0.05245300 W

F2 - Processing parameters
SI 131072
SF 100.6555028 MHz
WDW EM
SSB 0
LB 0 Hz
GB 0
PC 1.40

The ^{1}H NMR Spectrum of Compounds *trans*-**12a**

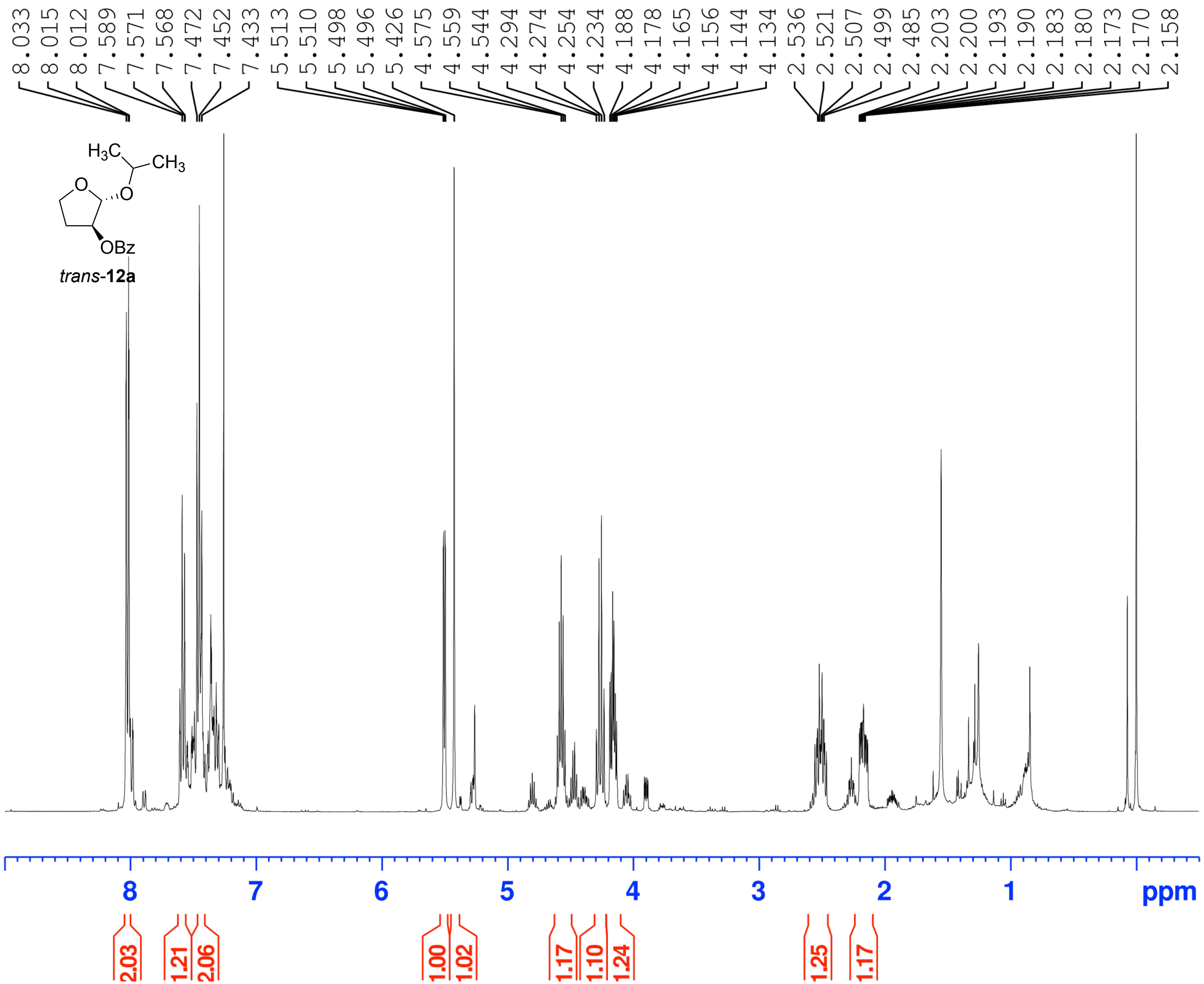

Current Data Parameters
NAME YC-4-149-col2
EXPNO 1
PROCNO 1

F2 - Acquisition Parameters
Date_ 20240330
Time 0.37 h
INSTRUM spect
PROBHD Z150354_0001 (
PULPROG zg30
TD 65536
SOLVENT CDCl3
NS 16
DS 2
SWH 8012.820 Hz
FIDRES 0.244532 Hz
AQ 4.0894465 sec
RG 125.92
DW 62.400 usec
DE 30.00 usec
TE 298.0 K
D1 1.00000000 sec
TD0 1
SFO1 400.3024719 MHz
NUC1 1H
P1 12.00 usec
PLW1 4.64209986 W

F2 - Processing parameters
SI 65536
SF 400.3000098 MHz
WDW EM
SSB 0
LB 0.30 Hz
GB 0
PC 1.00

The ¹H NMR Spectrum of Compounds *cis*-**12a**

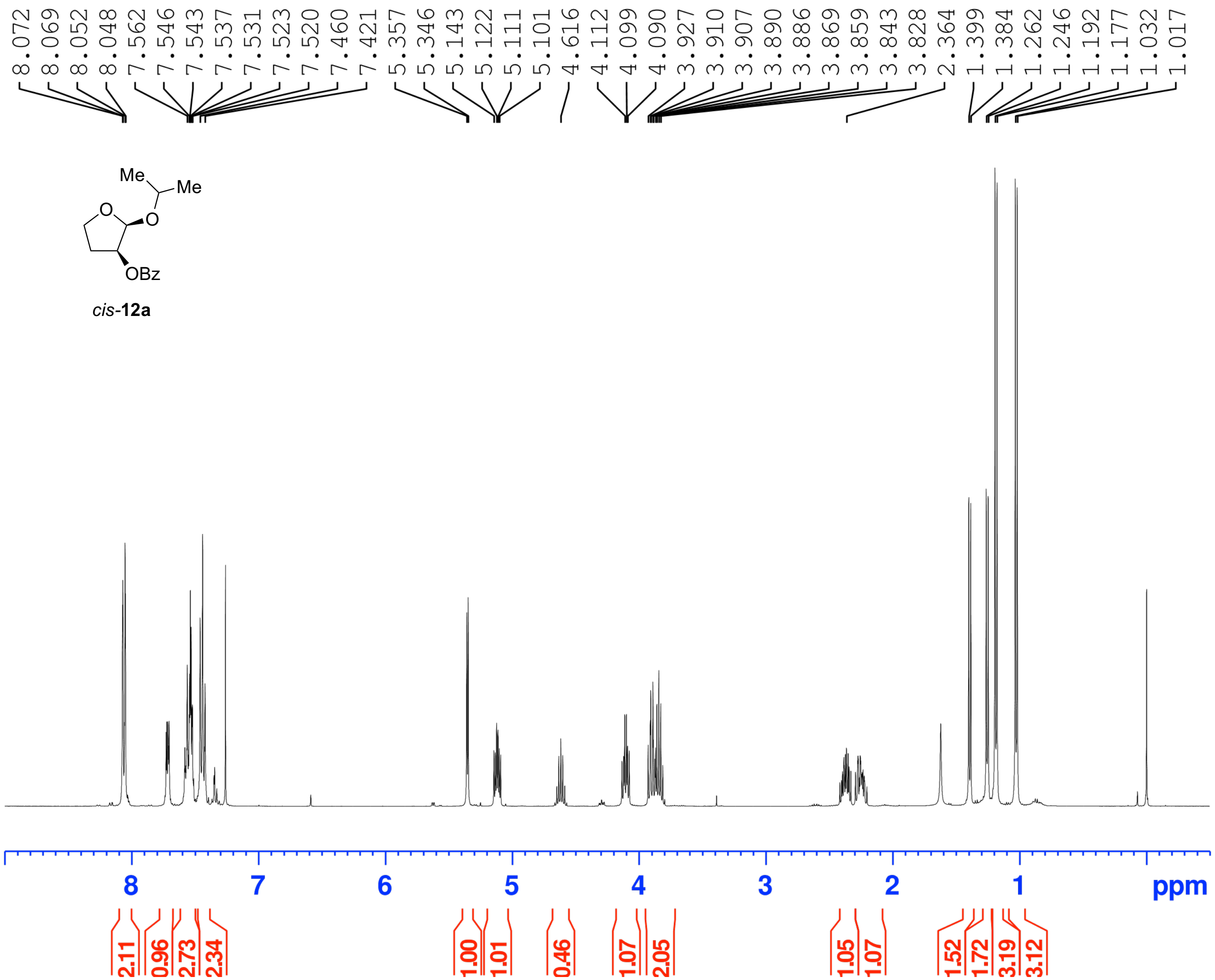

Current Data Parameters
NAME YC-4-175-col3-Fr55-65-2D
EXPNO 1
PROCNO 1

F2 - Acquisition Parameters
Date_ 20240509
Time 1.09 h
INSTRUM spect
PROBHD Z150354_0001 (
PULPROG zg30
TD 65536
SOLVENT CDCl3
NS 16
DS 2
SWH 8012.820 Hz
FIDRES 0.244532 Hz
AQ 4.0894465 sec
RG 81.45
DW 62.400 usec
DE 30.00 usec
TE 298.0 K
D1 1.00000000 sec
TD0 1
SFO1 400.3024719 MHz
NUC1 1H
P1 12.00 usec
PLW1 4.64209986 W

F2 - Processing parameters
SI 65536
SF 400.3000094 MHz
WDW EM
SSB 0
LB 0.30 Hz
GB 0
PC 1.00

The ¹H NMR Spectrum of Compounds *trans*-**13a**

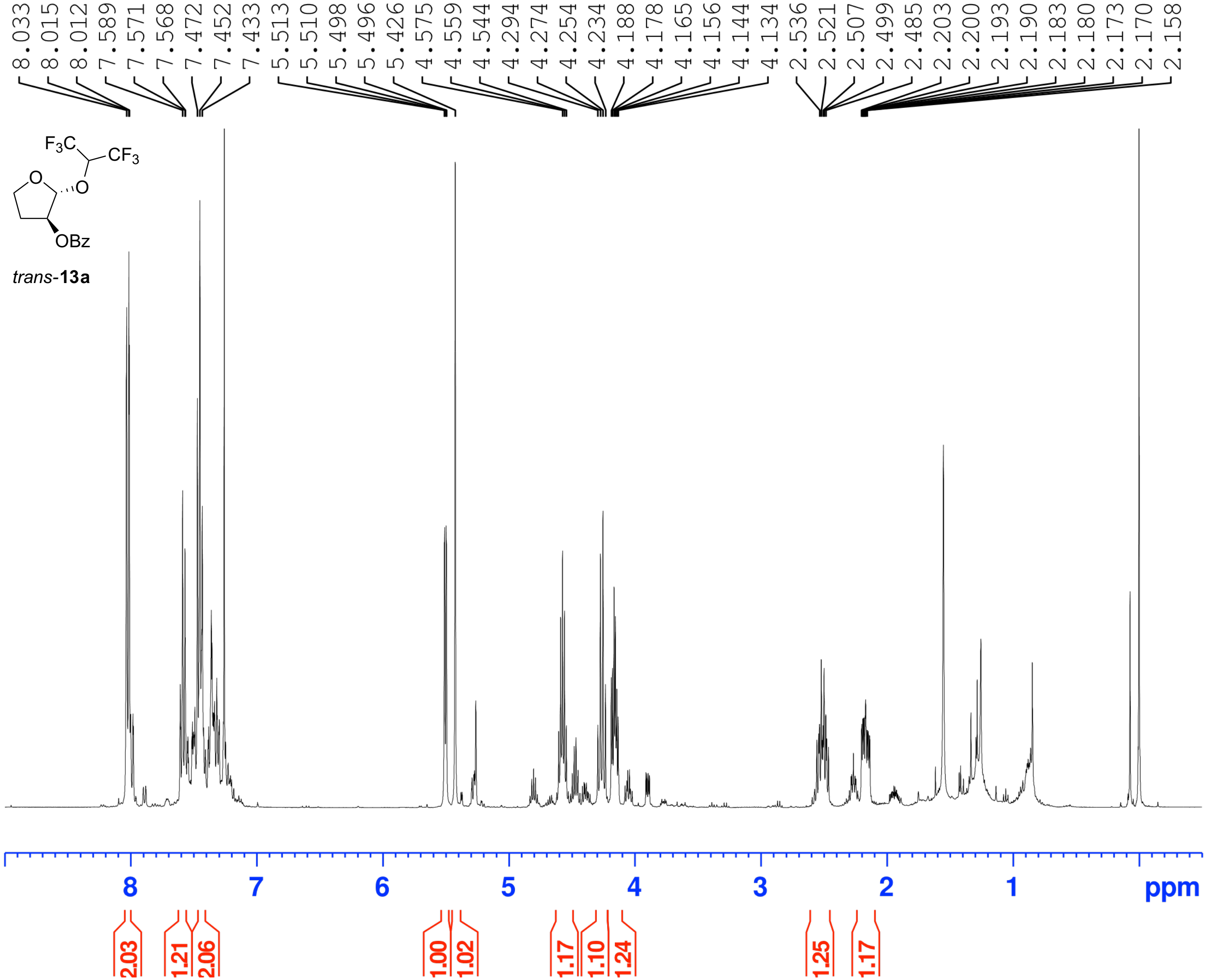

Current Data Parameters
NAME YC-4-149-col2
EXPNO 1
PROCNO 1

F2 - Acquisition Parameters
Date_ 20240330
Time 0.37 h
INSTRUM spect
PROBHD Z150354_0001 (
PULPROG zg30
TD 65536
SOLVENT CDCl3
NS 16
DS 2
SWH 8012.820 Hz
FIDRES 0.244532 Hz
AQ 4.0894465 sec
RG 125.92
DW 62.400 usec
DE 30.00 usec
TE 298.0 K
D1 1.00000000 sec
TD0 1
SFO1 400.3024719 MHz
NUC1 1H
P1 12.00 usec
PLW1 4.64209986 W

F2 - Processing parameters
SI 65536
SF 400.3000098 MHz
WDW EM
SSB 0
LB 0.30 Hz
GB 0
PC 1.00

The $^{13}C\{^{1}H\}$ NMR Spectrum of Compound *trans*-**13a**

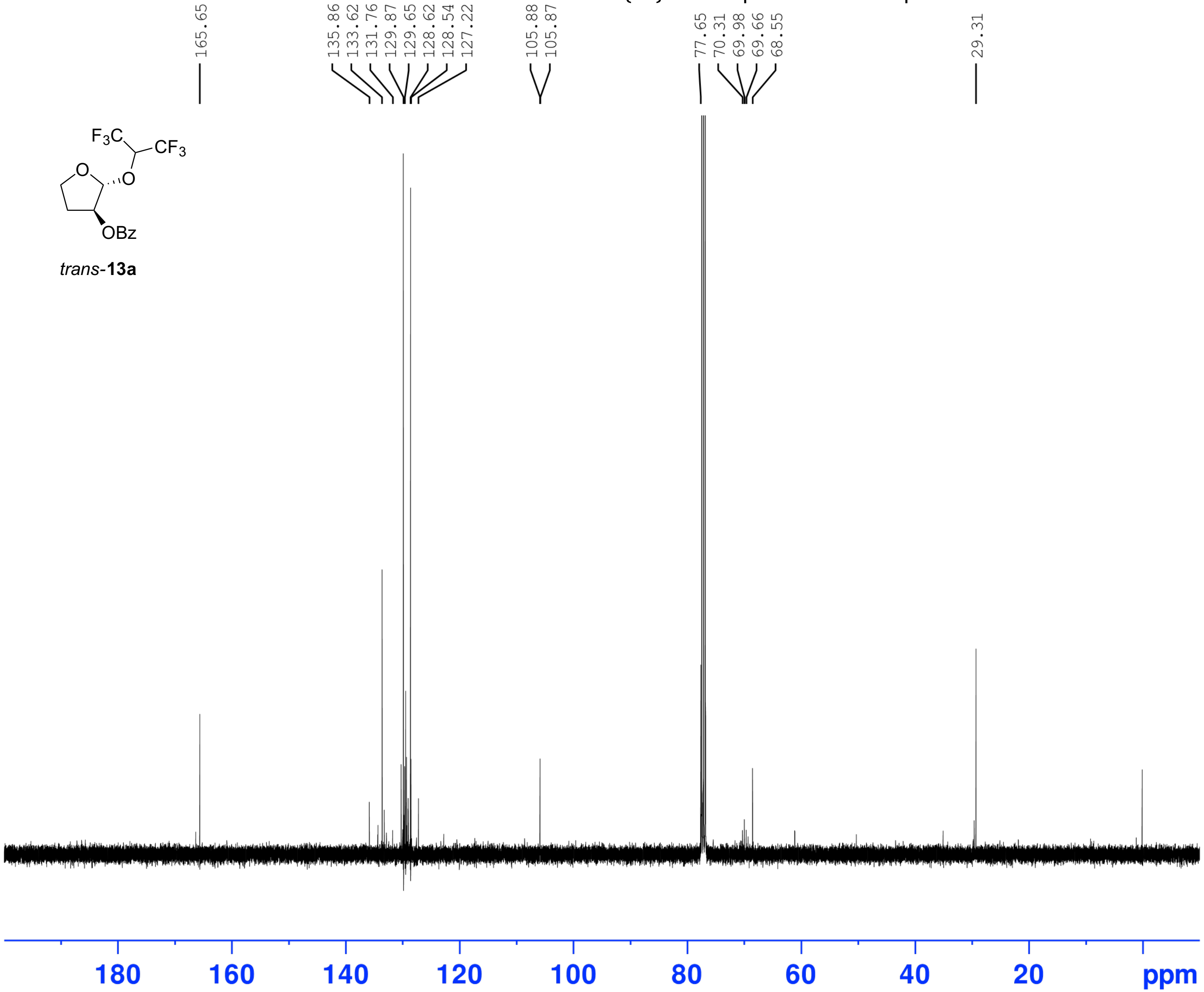

Current Data Parameters
NAME YC-4-149-col2
EXPNO 2
PROCNO 1

F2 - Acquisition Parameters
Date_ 20240330
Time 0.54 h
INSTRUM spect
PROBHD Z150354_0001 (
PULPROG zgpg30
TD 65356
SOLVENT CDCl3
NS 128
DS 4
SWH 24038.461 Hz
FIDRES 0.735616 Hz
AQ 1.3594048 sec
RG 36.68
DW 20.800 usec
DE 25.00 usec
TE 298.0 K
D1 2.00000000 sec
D11 0.03000000 sec
TD0 1
SFO1 100.6655806 MHz
NUC1 13C
P1 10.00 usec
PLW1 18.70700073 W
SFO2 400.3016012 MHz
NUC2 1H
CPDPRG[2 waltz16
PCPD2 80.00 usec
PLW2 4.64209986 W
PLW12 0.10445000 W
PLW13 0.05245300 W

F2 - Processing parameters
SI 131072
SF 100.6555028 MHz
WDW EM
SSB 0
LB 0 Hz
GB 0
PC 1.40

The ^{1}H NMR Spectrum of Compound **18a**

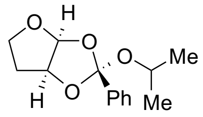

18a

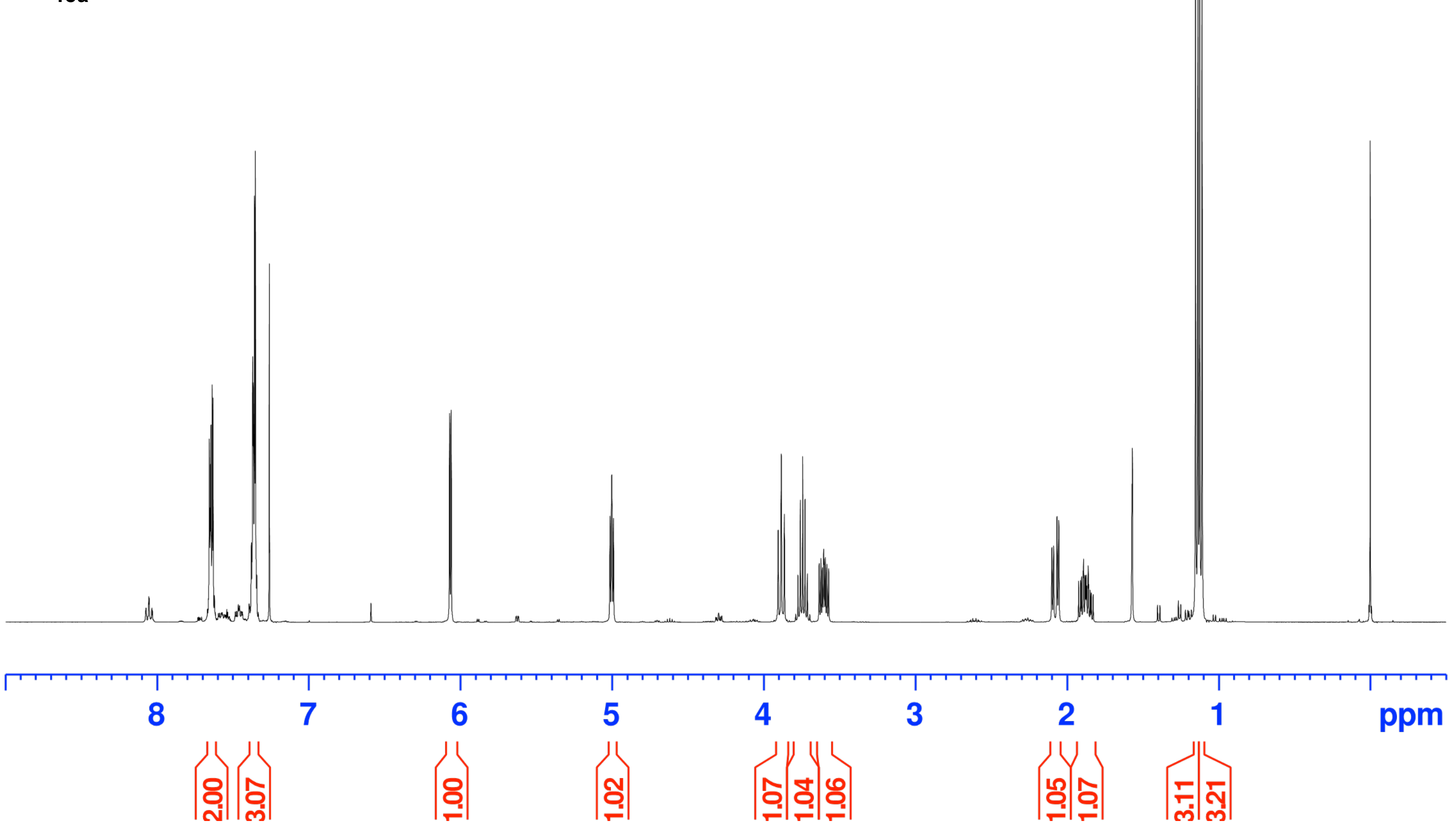


```
Current Data Parameters
NAME      YC-4-172-Fr104-121
EXPNO                      1
PROCNO                     1

F2 - Acquisition Parameters
Date_               20240502
Time                    5.05 h
INSTRUM                spect
PROBHD     Z150354_0001 (
PULPROG                 zg30
TD                     65536
SOLVENT                CDCl3
NS                        16
DS                         2
SWH                 8012.820 Hz
FIDRES              0.244532 Hz
AQ                 4.0894465 sec
RG                      92.4
DW                    62.400 usec
DE                     30.00 usec
TE                     298.0 K
D1                1.00000000 sec
TD0                        1
SFO1             400.3024719 MHz
NUC1                      1H
P1                     12.00 usec
PLW1              4.64209986 W

F2 - Processing parameters
SI                     65536
SF               400.3000093 MHz
WDW                       EM
SSB                        0
LB                      0.30 Hz
GB                         0
PC                      1.00
```

The $^{13}C\{^{1}H\}$ NMR Spectrum of Compound **18a**

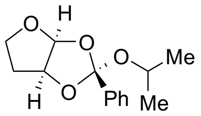

18a

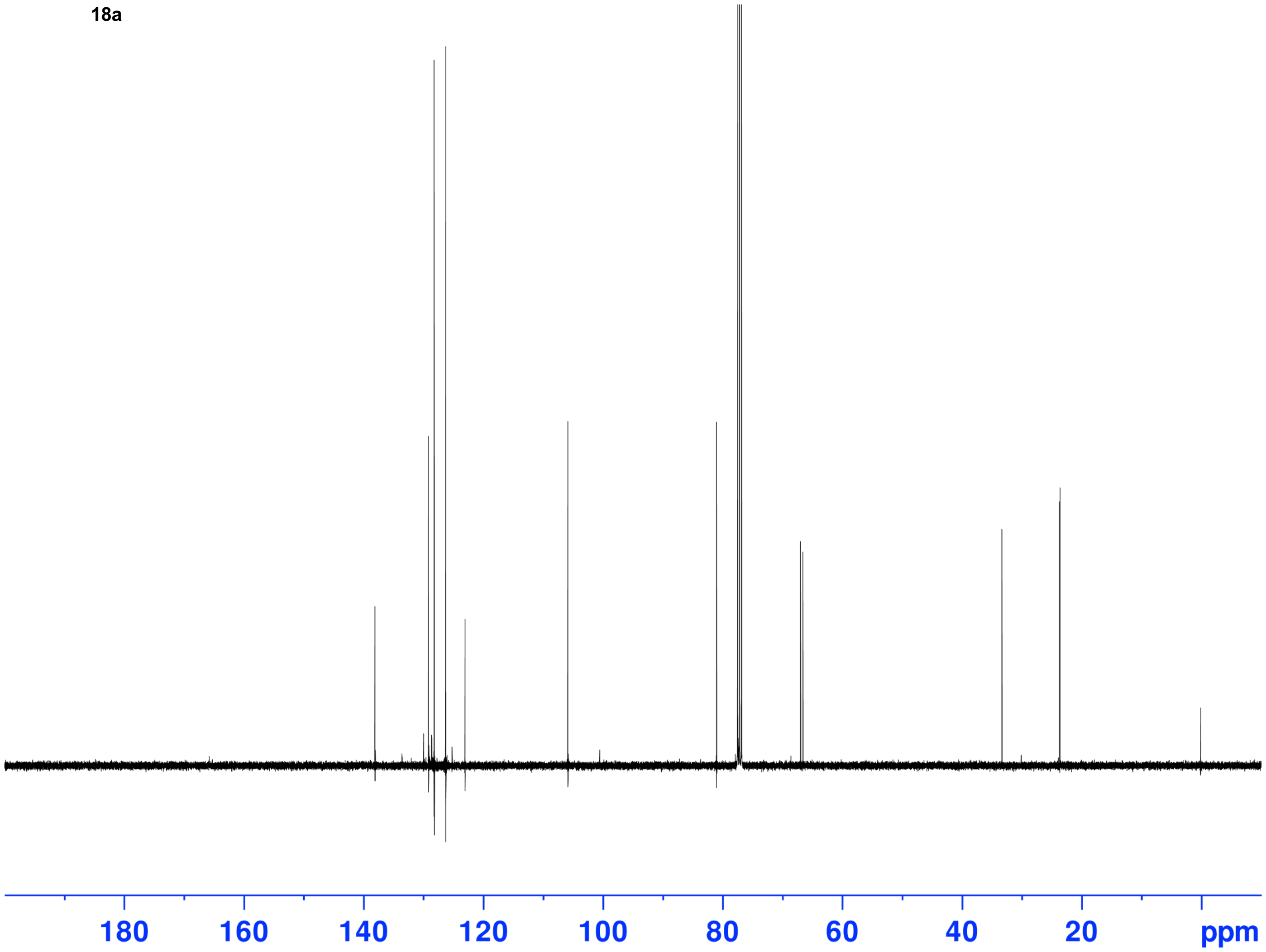


```
Current Data Parameters
NAME        YC-4-172-Fr104-121
EXPNO                        2
PROCNO                       1

F2 - Acquisition Parameters
Date_               20240502
Time                    7.48 h
INSTRUM                spect
PROBHD        Z150354_0001 (
PULPROG              zgpg30
TD                     65356
SOLVENT                CDCl3
NS                       128
DS                         4
SWH               24038.461 Hz
FIDRES             0.735616 Hz
AQ                1.3594048 sec
RG                     51.78
DW                    20.800 usec
DE                     25.00 usec
TE                     298.0 K
D1               2.00000000 sec
D11              0.03000000 sec
TD0                        1
SFO1            100.6655806 MHz
NUC1                     13C
P1                     10.00 usec
PLW1            18.70700073 W
SFO2            400.3016012 MHz
NUC2                      1H
CPDPRG[2             waltz16
PCPD2                  80.00 usec
PLW2             4.64209986 W
PLW12            0.10445000 W
PLW13            0.05245300 W

F2 - Processing parameters
SI                    131072
SF              100.6554995 MHz
WDW                       EM
SSB                        0
LB                         0 Hz
GB                         0
PC                      1.40
```

The ^{1}H NMR Spectrum of Compound *trans*-**9b**

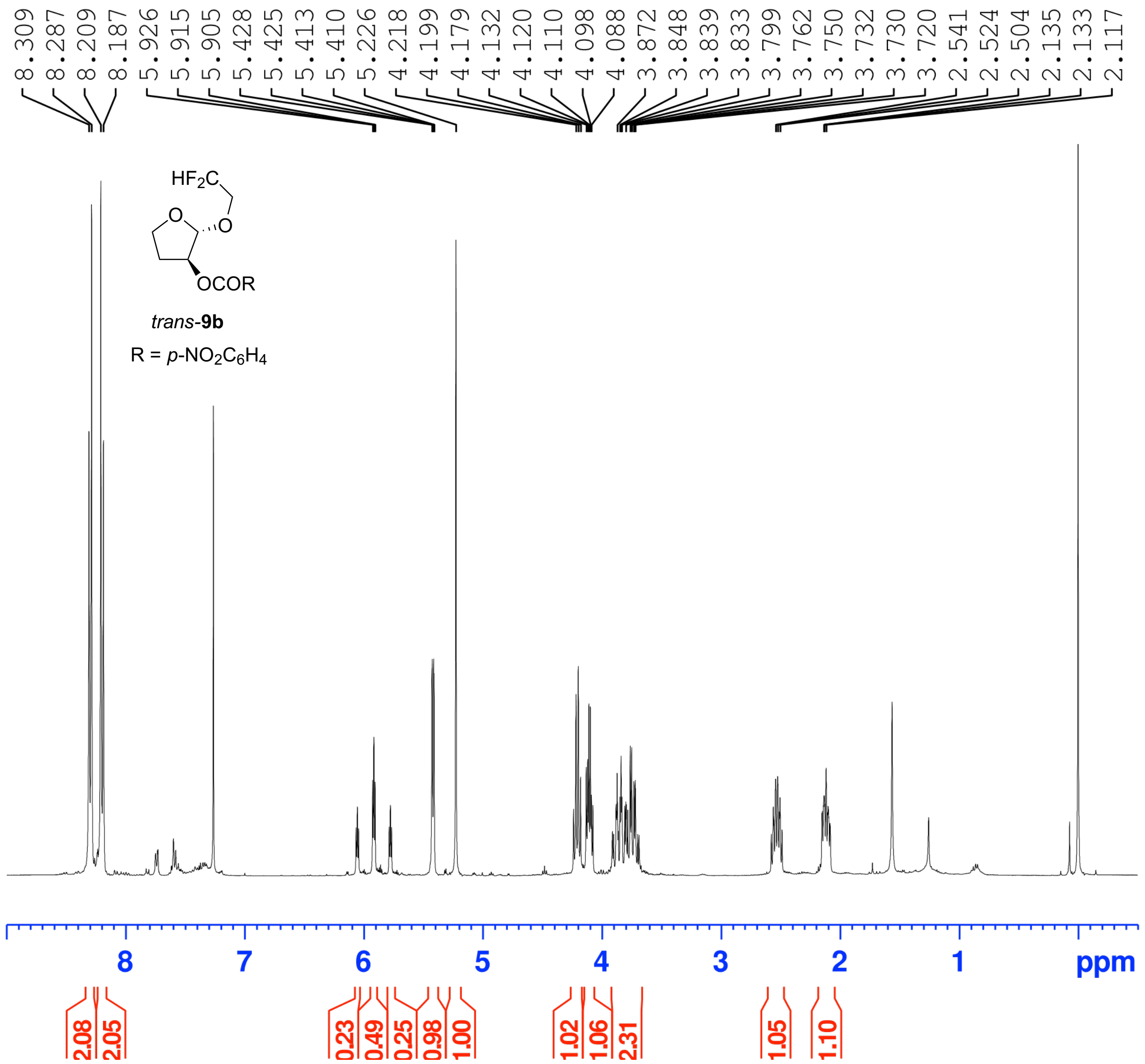


```
Current Data Parameters
NAME       YC-4-136-Fr31-34
EXPNO                     1
PROCNO                    1

F2 - Acquisition Parameters
Date_              20240305
Time                 0.30 h
INSTRUM               spect
PROBHD     Z150354_0001 (
PULPROG                zg30
TD                    65536
SOLVENT               CDCl3
NS                       16
DS                        2
SWH             8012.820 Hz
FIDRES          0.244532 Hz
AQ             4.0894465 sec
RG                   125.92
DW               62.400 usec
DE                30.00 usec
TE                298.0 K
D1            1.00000000 sec
TD0                       1
SFO1         400.3024719 MHz
NUC1                     1H
P1                12.00 usec
PLW1          4.64209986 W

F2 - Processing parameters
SI                    65536
SF           400.3000080 MHz
WDW                      EM
SSB                       0
LB                   0.30 Hz
GB                        0
PC                     1.00
```

The $^{13}C\{^1H\}$ NMR Spectrum of Compound *trans*-**9b**

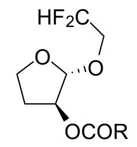

trans-**9b**

R = *p*-$NO_2C_6H_4$

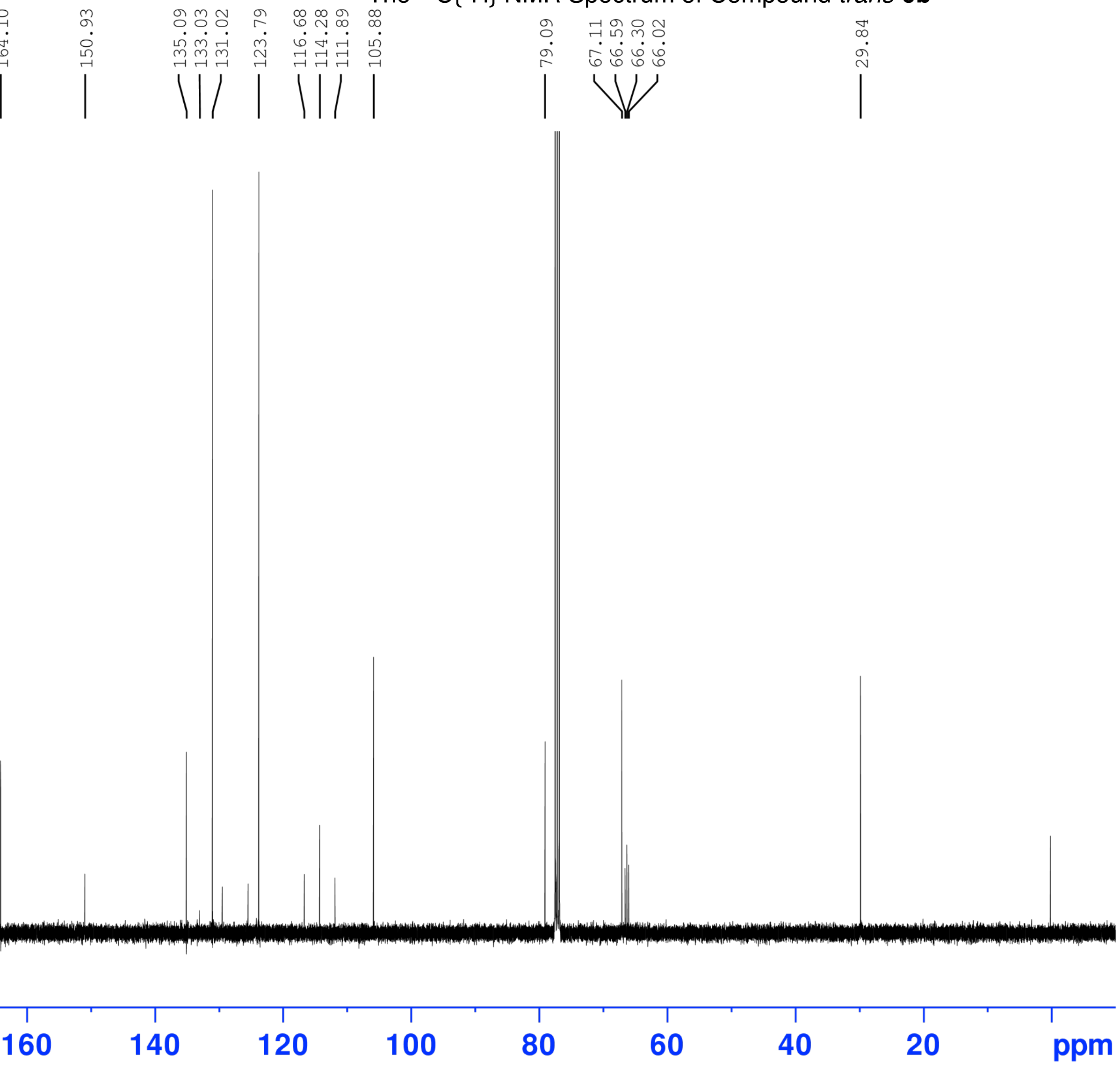

Current Data Parameters
NAME YC-4-136-Fr31-34
EXPNO 3
PROCNO 1

F2 - Acquisition Parameters
Date_ 20240305
Time 6.37 h
INSTRUM spect
PROBHD Z150354_0001 (
PULPROG zgpg30
TD 65356
SOLVENT CDCl3
NS 128
DS 4
SWH 24038.461 Hz
FIDRES 0.735616 Hz
AQ 1.3594048 sec
RG 29.21
DW 20.800 usec
DE 25.00 usec
TE 298.0 K
D1 2.00000000 sec
D11 0.03000000 sec
TD0 1
SFO1 100.6655806 MHz
NUC1 13C
P1 10.00 usec
PLW1 18.70700073 W
SFO2 400.3016012 MHz
NUC2 1H
CPDPRG[2 waltz16
PCPD2 80.00 usec
PLW2 4.64209986 W
PLW12 0.10445000 W
PLW13 0.05245300 W

F2 - Processing parameters
SI 131072
SF 100.6554989 MHz
WDW EM
SSB 0
LB 0 Hz
GB 0
PC 1.40

The ^{1}H NMR Spectrum of Compound *trans*-**10b**

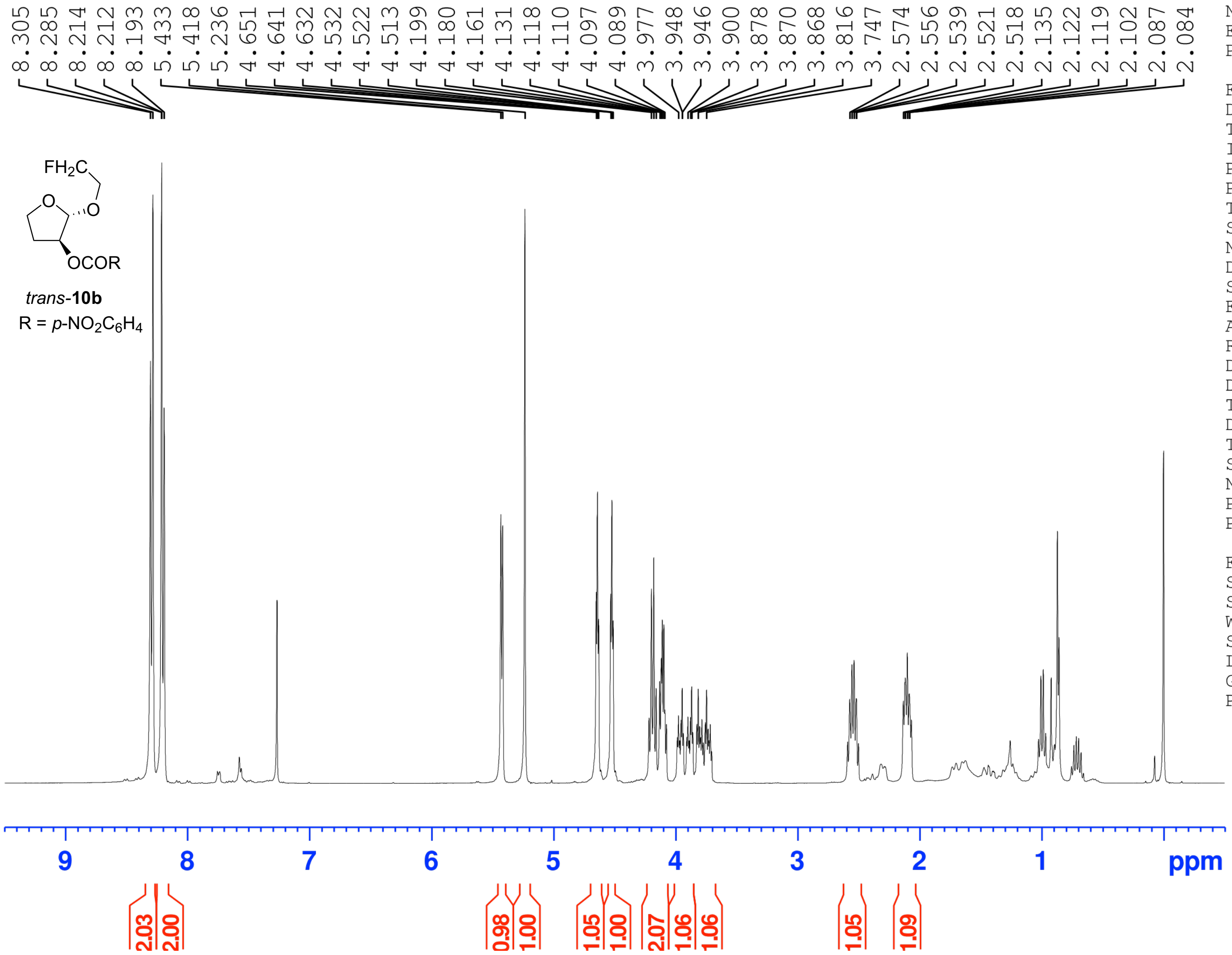

trans-**10b**

R = *p*-$NO_2C_6H_4$

Current Data Parameters
NAME YC-4-147-col1
EXPNO 1
PROCNO 1

F2 - Acquisition Parameters
Date_ 20240316
Time 1.49 h
INSTRUM spect
PROBHD Z150354_0001 (
PULPROG zg30
TD 65536
SOLVENT CDCl3
NS 16
DS 2
SWH 8012.820 Hz
FIDRES 0.244532 Hz
AQ 4.0894465 sec
RG 92.4
DW 62.400 usec
DE 30.00 usec
TE 298.0 K
D1 1.00000000 sec
TD0 1
SFO1 400.3024719 MHz
NUC1 1H
P1 12.00 usec
PLW1 4.64209986 W

F2 - Processing parameters
SI 65536
SF 400.3000058 MHz
WDW EM
SSB 0
LB 0.30 Hz
GB 0
PC 1.00

The $^{13}C\{^1H\}$ NMR Spectrum of Compound *trans*-**10b**

trans-**10b**

R = *p*-$NO_2C_6H_4$

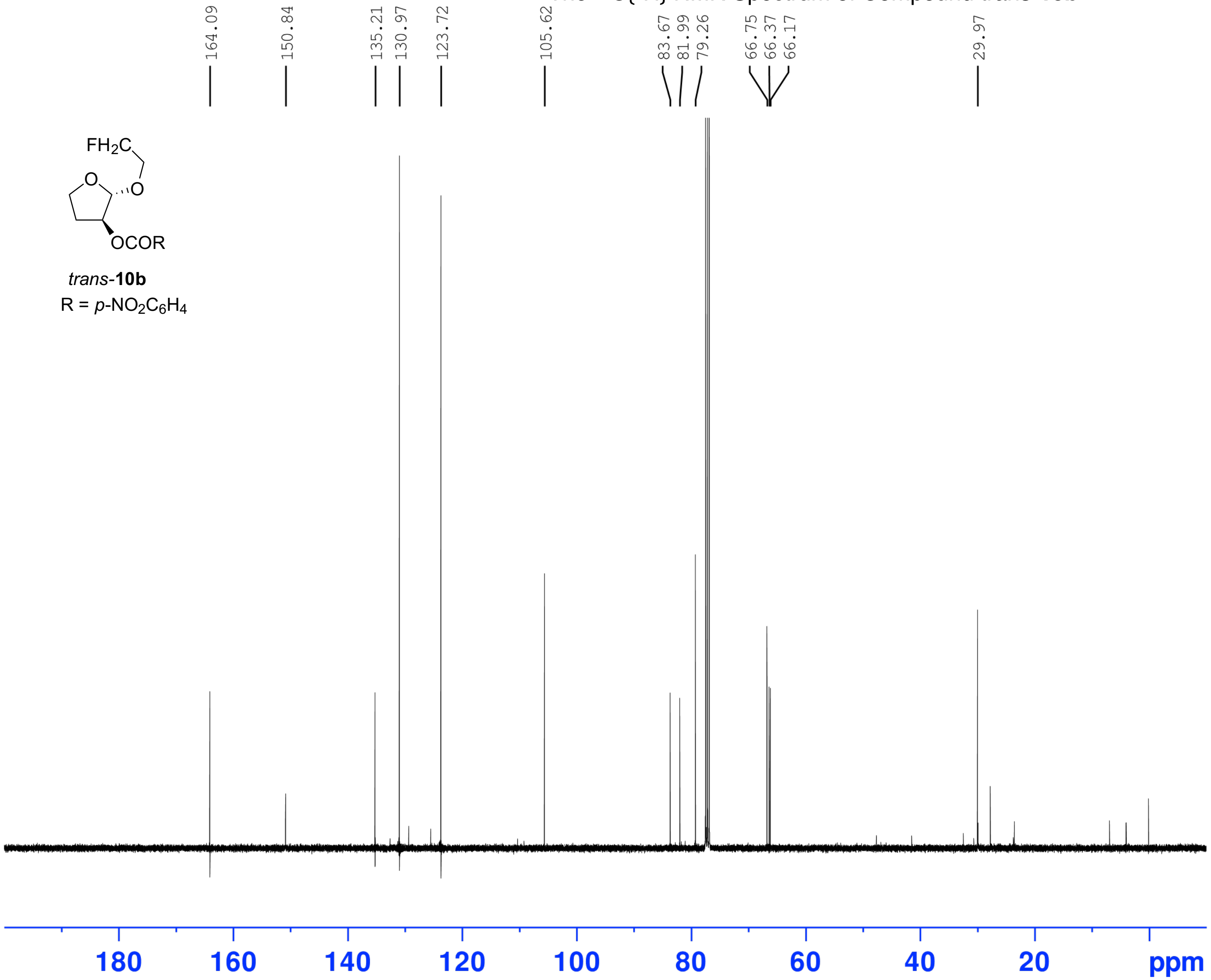


```
Current Data Parameters
NAME        YC-4-147-col1
EXPNO                   2
PROCNO                  1

F2 - Acquisition Parameters
Date_            20240316
Time                 7.35 h
INSTRUM             spect
PROBHD    Z150354_0001 (
PULPROG           zgpg30
TD                 65356
SOLVENT            CDCl3
NS                   128
DS                     4
SWH            24038.461 Hz
FIDRES          0.735616 Hz
AQ             1.3594048 sec
RG                 45.21
DW                20.800 usec
DE                 25.00 usec
TE                 298.0 K
D1            2.00000000 sec
D11           0.03000000 sec
TD0                    1
SFO1        100.6655806 MHz
NUC1                 13C
P1                 10.00 usec
PLW1         18.70700073 W
SFO2        400.3016012 MHz
NUC2                  1H
CPDPRG[2         waltz16
PCPD2              80.00 usec
PLW2          4.64209986 W
PLW12         0.10445000 W
PLW13         0.05245300 W

F2 - Processing parameters
SI                131072
SF           100.6555030 MHz
WDW                   EM
SSB                    0
LB                     0 Hz
GB                     0
PC                  1.40
```

The ¹H NMR Spectrum of Compound *cis*-**10b**

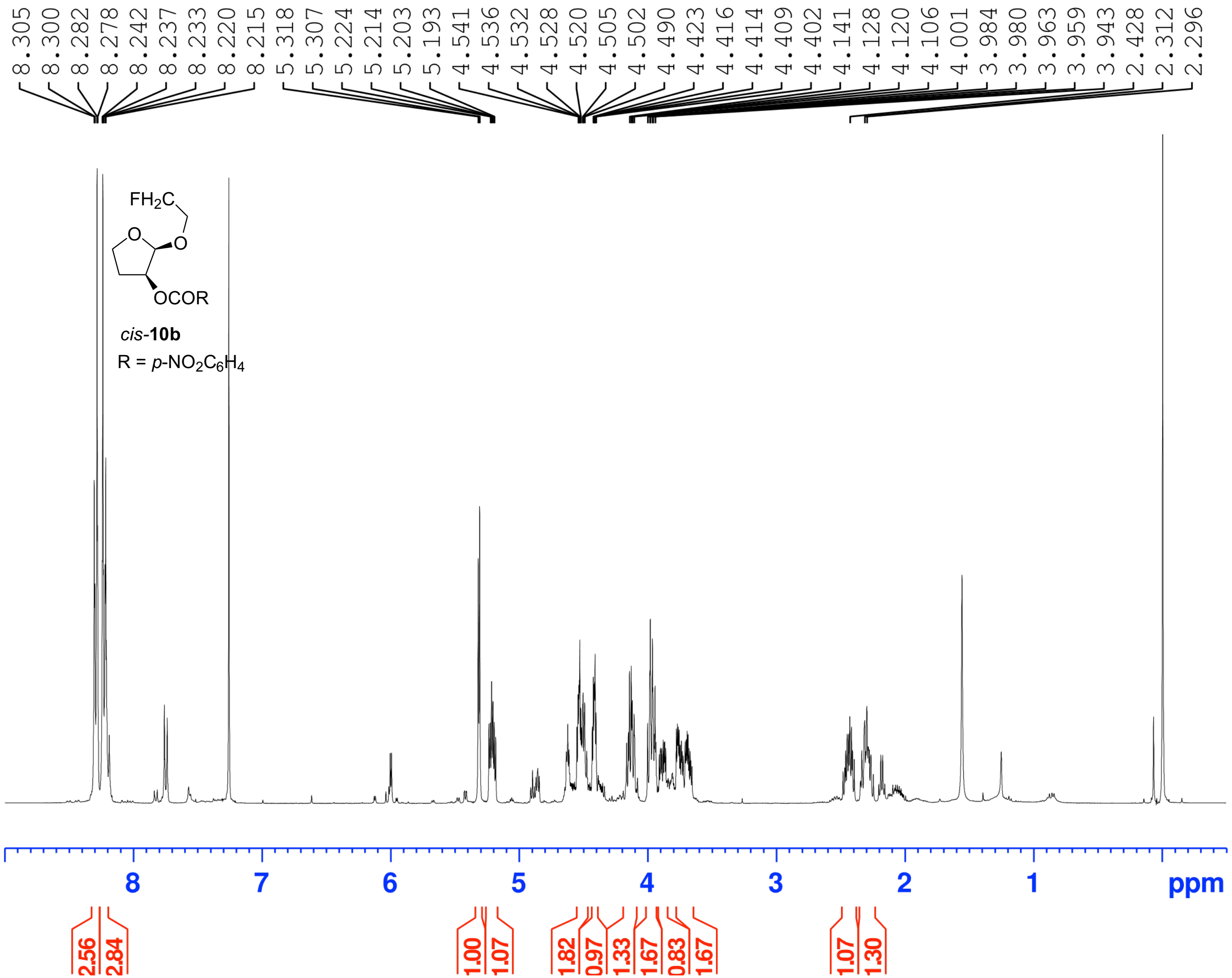

Current Data Parameters
NAME YC-4-147-col2
EXPNO 1
PROCNO 1

F2 - Acquisition Parameters
Date_ 20240321
Time 2.05 h
INSTRUM spect
PROBHD Z150354_0001 (
PULPROG zg30
TD 65536
SOLVENT CDCl3
NS 16
DS 2
SWH 8012.820 Hz
FIDRES 0.244532 Hz
AQ 4.0894465 sec
RG 125.92
DW 62.400 usec
DE 30.00 usec
TE 298.0 K
D1 1.00000000 sec
TD0 1
SFO1 400.3024719 MHz
NUC1 1H
P1 12.00 usec
PLW1 4.64209986 W

F2 - Processing parameters
SI 65536
SF 400.3000105 MHz
WDW EM
SSB 0
LB 0.30 Hz
GB 0
PC 1.00

The $^{13}C\{^{1}H\}$ NMR Spectrum of Compound *cis*-**10b**

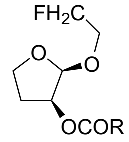

cis-**10b**
R = *p*-$NO_2C_6H_4$

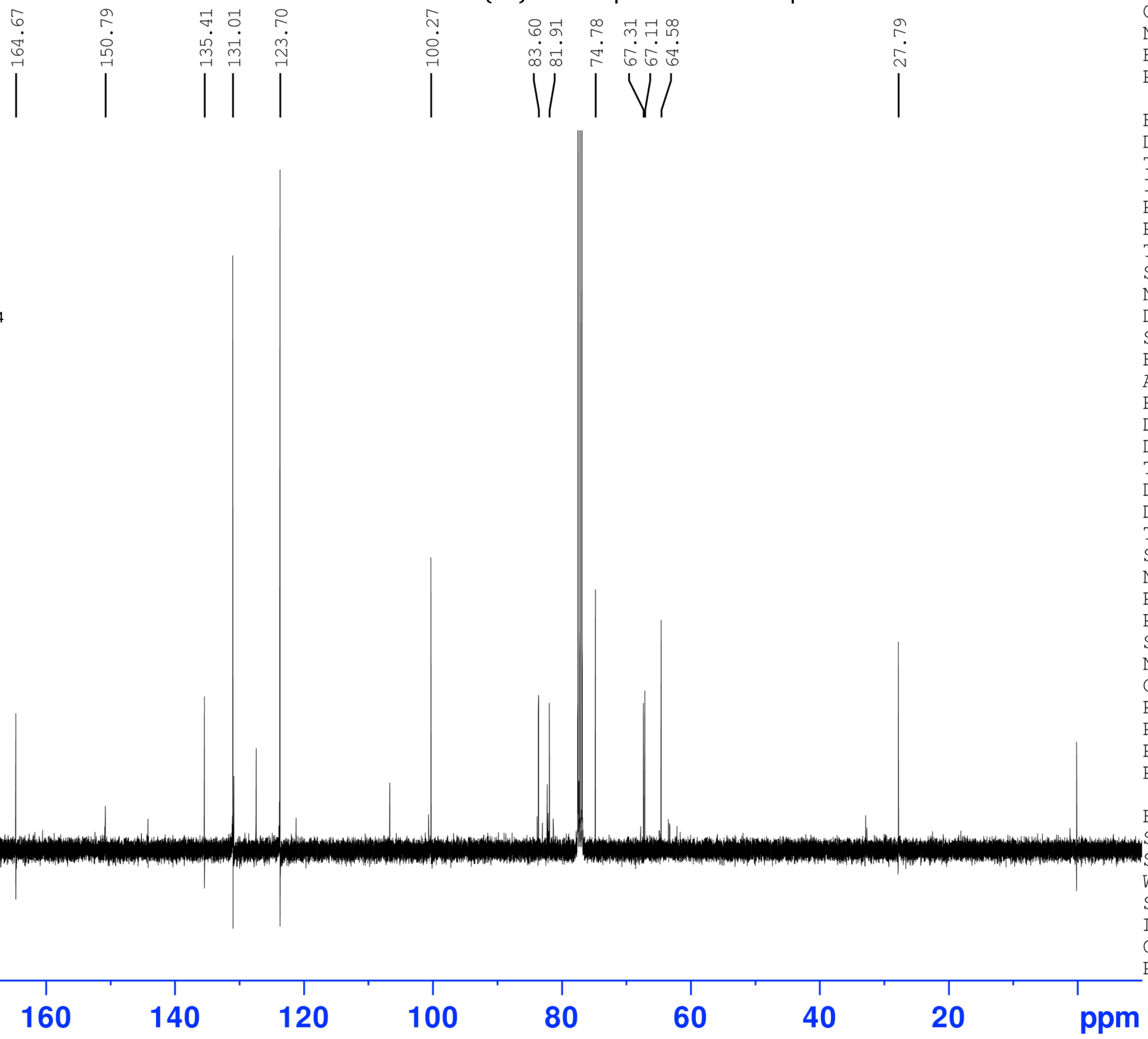

Current Data Parameters
NAME YC-4-147-col2
EXPNO 2
PROCNO 1

F2 - Acquisition Parameters
Date_ 20240321
Time 12.09 h
INSTRUM spect
PROBHD Z150354_0001 (
PULPROG zgpg30
TD 65356
SOLVENT CDCl3
NS 128
DS 4
SWH 24038.461 Hz
FIDRES 0.735616 Hz
AQ 1.3594048 sec
RG 45.21
DW 20.800 usec
DE 25.00 usec
TE 298.0 K
D1 2.00000000 sec
D11 0.03000000 sec
TD0 1
SFO1 100.6655806 MHz
NUC1 13C
P1 10.00 usec
PLW1 18.70700073 W
SFO2 400.3016012 MHz
NUC2 1H
CPDPRG[2 waltz16
PCPD2 80.00 usec
PLW2 4.64209986 W
PLW12 0.10445000 W
PLW13 0.05245300 W

F2 - Processing parameters
SI 131072
SF 100.6555015 MHz
WDW EM
SSB 0
LB 0 Hz
GB 0
PC 1.40

The ^{1}H NMR Spectrum of Compounds *trans*-**11b** and *cis*-**11b**

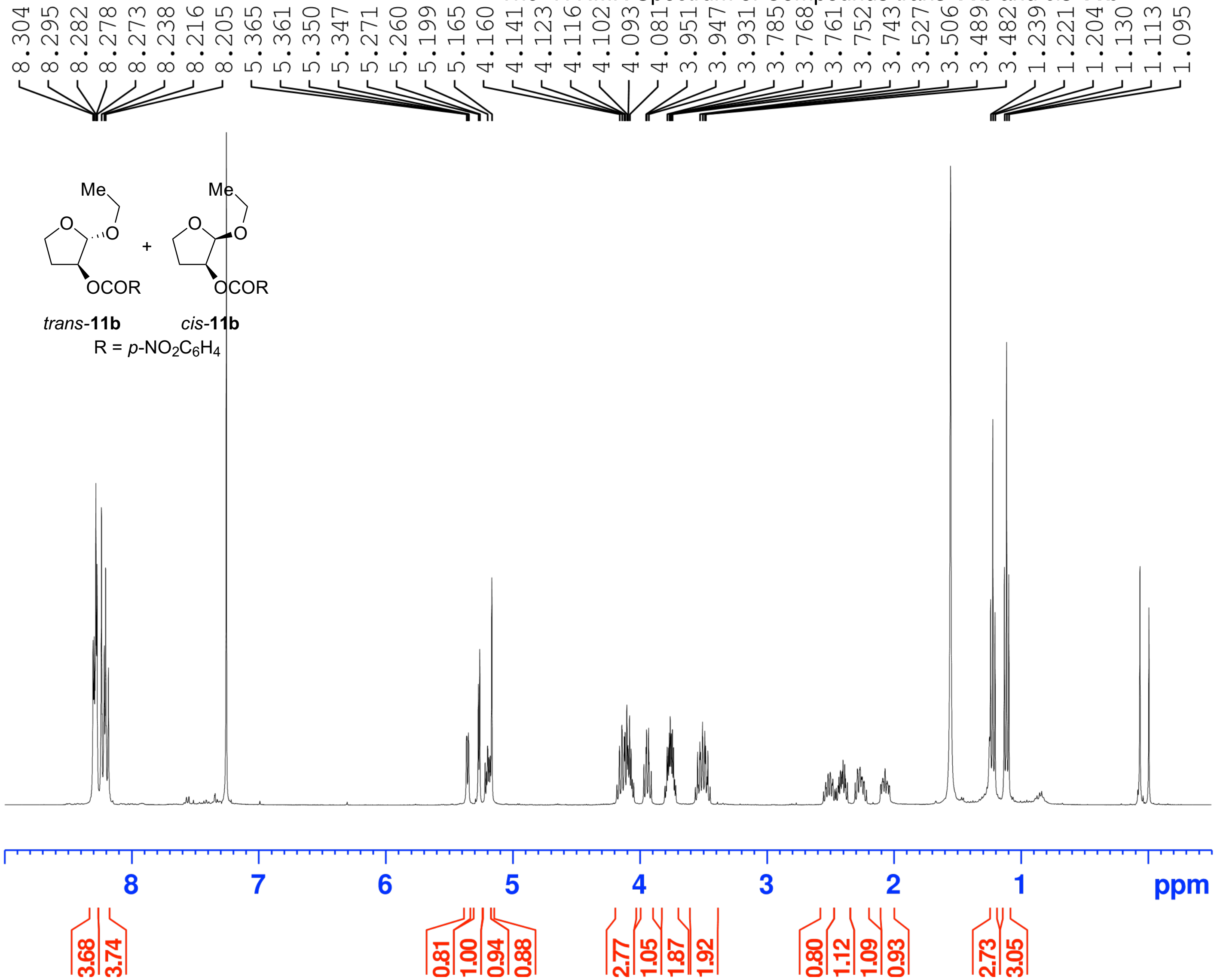


```
Current Data Parameters
NAME      YC-4-159-Fr44-56
EXPNO                    1
PROCNO                   1

F2 - Acquisition Parameters
Date_             20240411
Time                 23.47 h
INSTRUM              spect
PROBHD    Z150354_0001 (
PULPROG               zg30
TD                   65536
SOLVENT              CDCl3
NS                      16
DS                       2
SWH               8012.820 Hz
FIDRES            0.244532 Hz
AQ               4.0894465 sec
RG                   164.8
DW                  62.400 usec
DE                   30.00 usec
TE                   298.0 K
D1              1.00000000 sec
TD0                      1
SFO1           400.3024719 MHz
NUC1                    1H
P1                   12.00 usec
PLW1            4.64209986 W

F2 - Processing parameters
SI                   65536
SF             400.3000113 MHz
WDW                     EM
SSB                      0
LB                    0.30 Hz
GB                       0
PC                    1.00
```

The $^{13}C\{^{1}H\}$ NMR Spectrum of Compound *cis*-**12b**

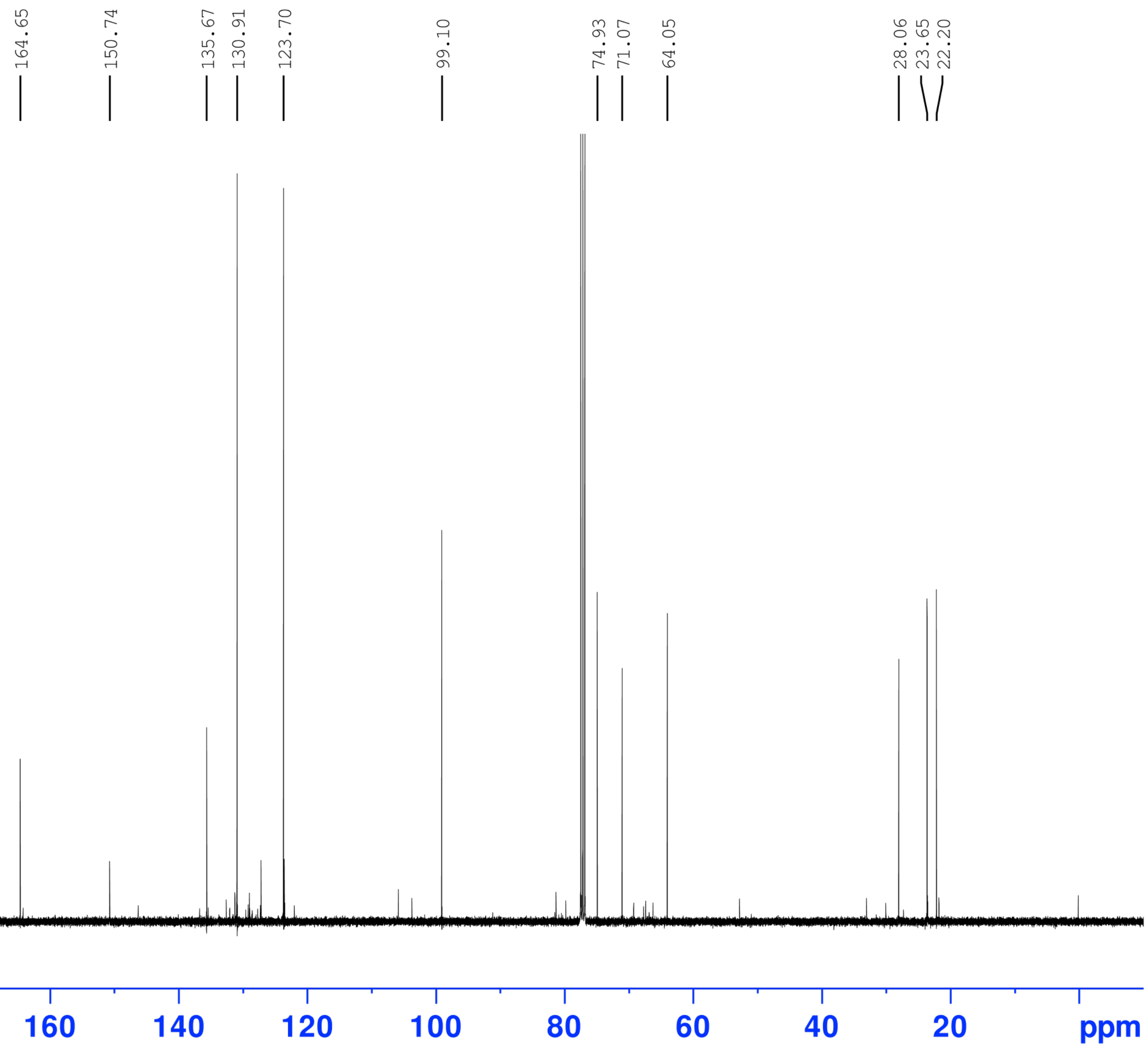

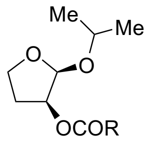

cis-**12b**

R = *p*-$NO_2C_6H_4$

Current Data Parameters
NAME YC-4-181-Fr46-53
EXPNO 2
PROCNO 1

F2 - Acquisition Parameters
Date_ 20240514
Time 1.30 h
INSTRUM spect
PROBHD Z150354_0001 (
PULPROG zgpg30
TD 65356
SOLVENT CDCl3
NS 128
DS 4
SWH 24038.461 Hz
FIDRES 0.735616 Hz
AQ 1.3594048 sec
RG 31.4
DW 20.800 usec
DE 25.00 usec
TE 298.0 K
D1 2.00000000 sec
D11 0.03000000 sec
TD0 1
SFO1 100.6655806 MHz
NUC1 13C
P1 10.00 usec
PLW1 18.70700073 W
SFO2 400.3016012 MHz
NUC2 1H
CPDPRG[2 waltz16
PCPD2 80.00 usec
PLW2 4.64209986 W
PLW12 0.10445000 W
PLW13 0.05245300 W

F2 - Processing parameters
SI 131072
SF 100.6555009 MHz
WDW EM
SSB 0
LB 0 Hz
GB 0
PC 1.40

The $^{13}C\{^{1}H\}$ NMR Spectrum of Compounds *trans*-**11b** and *cis*-**11b**

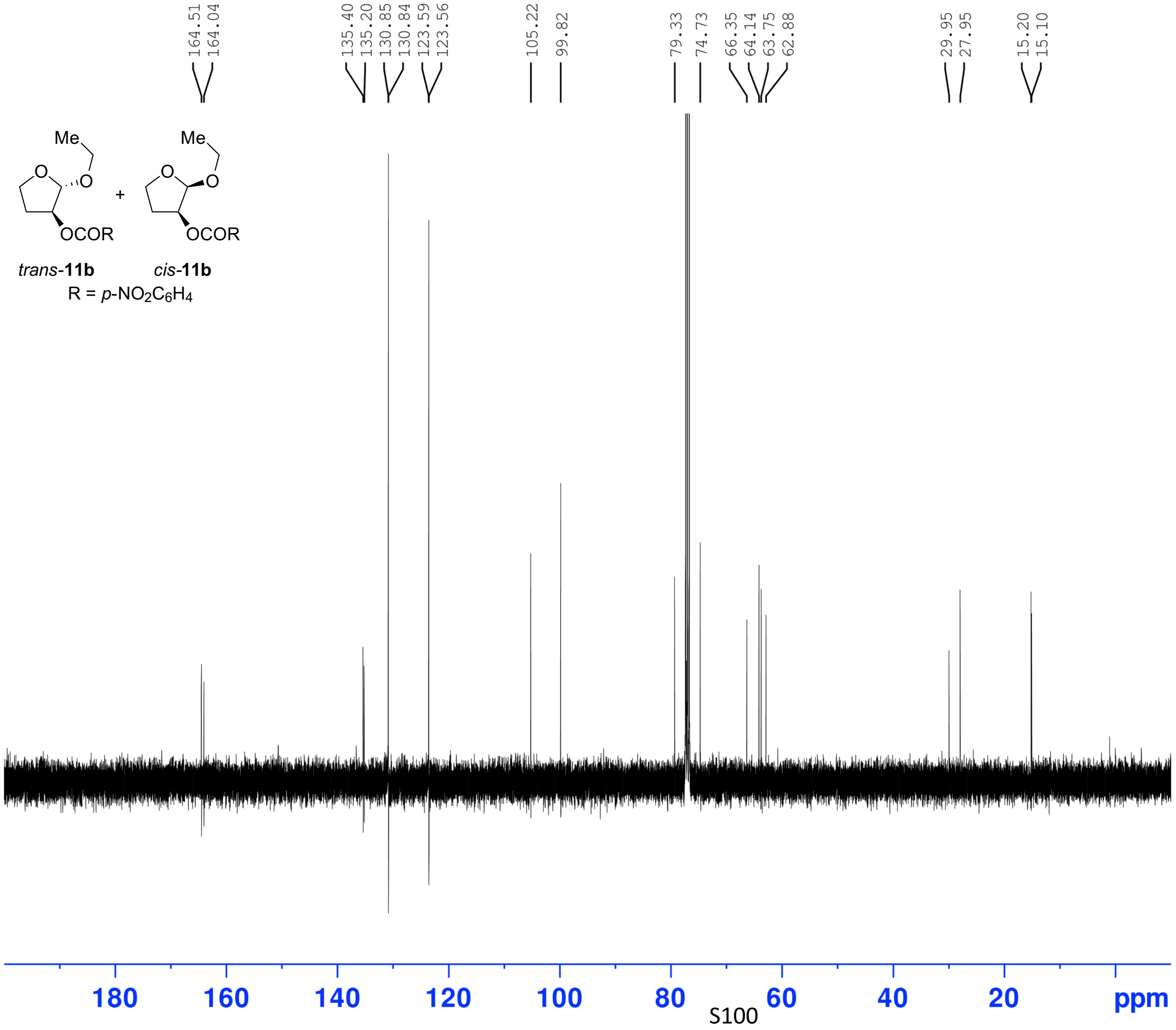

Current Data Parameters
NAME YC-4-159-Fr44-56
EXPNO 2
PROCNO 1

F2 - Acquisition Parameters
Date_ 20240412
Time 2.20 h
INSTRUM spect
PROBHD Z150354_0001 (
PULPROG zgpg30
TD 65356
SOLVENT CDCl3
NS 128
DS 4
SWH 24038.461 Hz
FIDRES 0.735616 Hz
AQ 1.3594048 sec
RG 51.78
DW 20.800 usec
DE 25.00 usec
TE 298.0 K
D1 2.00000000 sec
D11 0.03000000 sec
TD0 1
SFO1 100.6655806 MHz
NUC1 13C
P1 10.00 usec
PLW1 18.70700073 W
SFO2 400.3016012 MHz
NUC2 1H
CPDPRG[2 waltz16
PCPD2 80.00 usec
PLW2 4.64209986 W
PLW12 0.10445000 W
PLW13 0.05245300 W

F2 - Processing parameters
SI 131072
SF 100.6555151 MHz
WDW EM
SSB 0
LB 0 Hz
GB 0
PC 1.40

The ^{1}H NMR Spectrum of Compound *cis*-**12b**

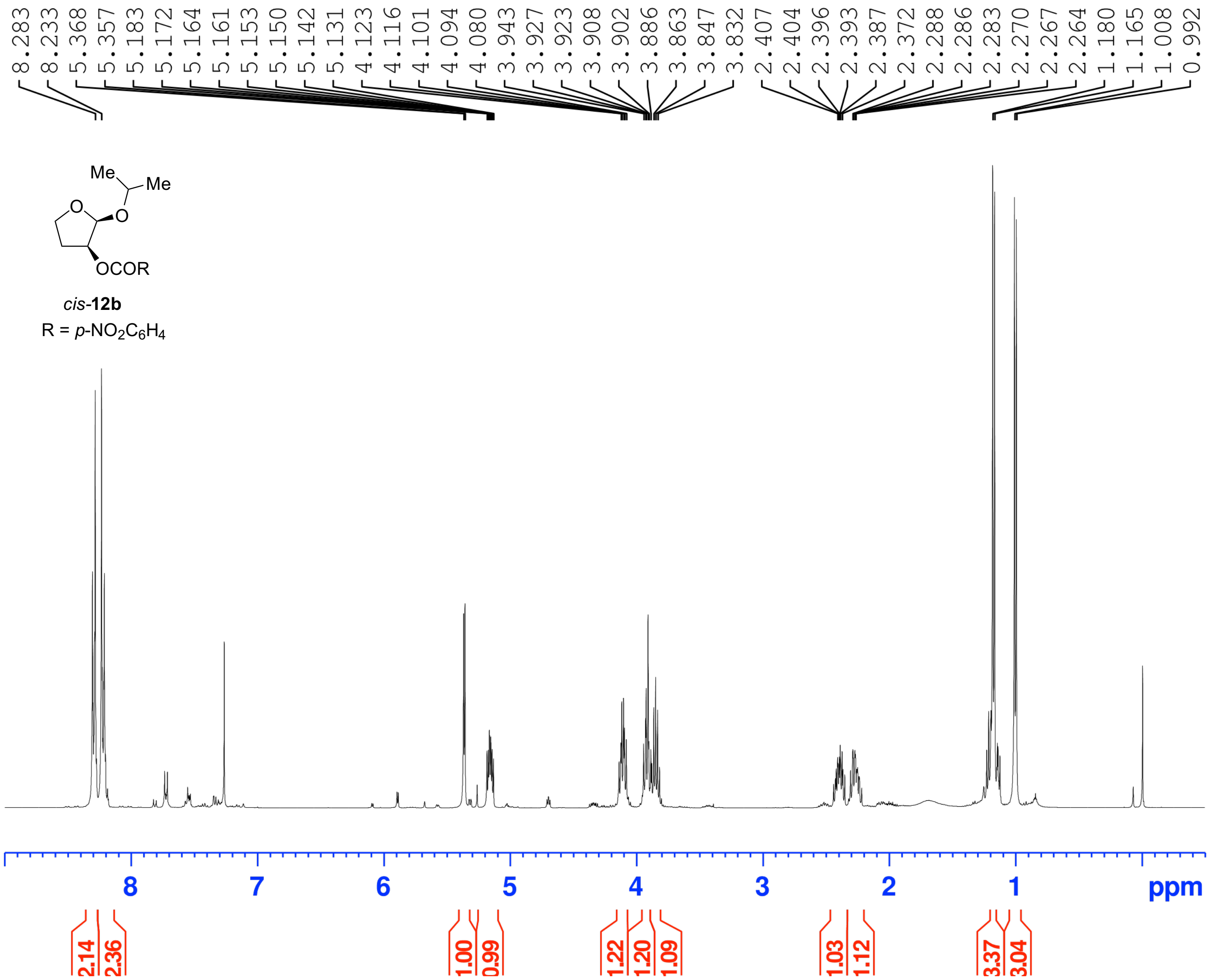

Current Data Parameters
NAME YC-4-181-Fr46-53
EXPNO 1
PROCNO 1

F2 - Acquisition Parameters
Date_ 20240514
Time 1.08 h
INSTRUM spect
PROBHD Z150354_0001 (
PULPROG zg30
TD 65536
SOLVENT CDCl3
NS 16
DS 2
SWH 8012.820 Hz
FIDRES 0.244532 Hz
AQ 4.0894465 sec
RG 70.76
DW 62.400 usec
DE 30.00 usec
TE 298.0 K
D1 1.00000000 sec
TD0 1
SFO1 400.3024719 MHz
NUC1 1H
P1 12.00 usec
PLW1 4.64209986 W

F2 - Processing parameters
SI 65536
SF 400.3000083 MHz
WDW EM
SSB 0
LB 0.30 Hz
GB 0
PC 1.00

The $^{13}C\{^{1}H\}$ NMR Spectrum of Compound *trans*-**12b**

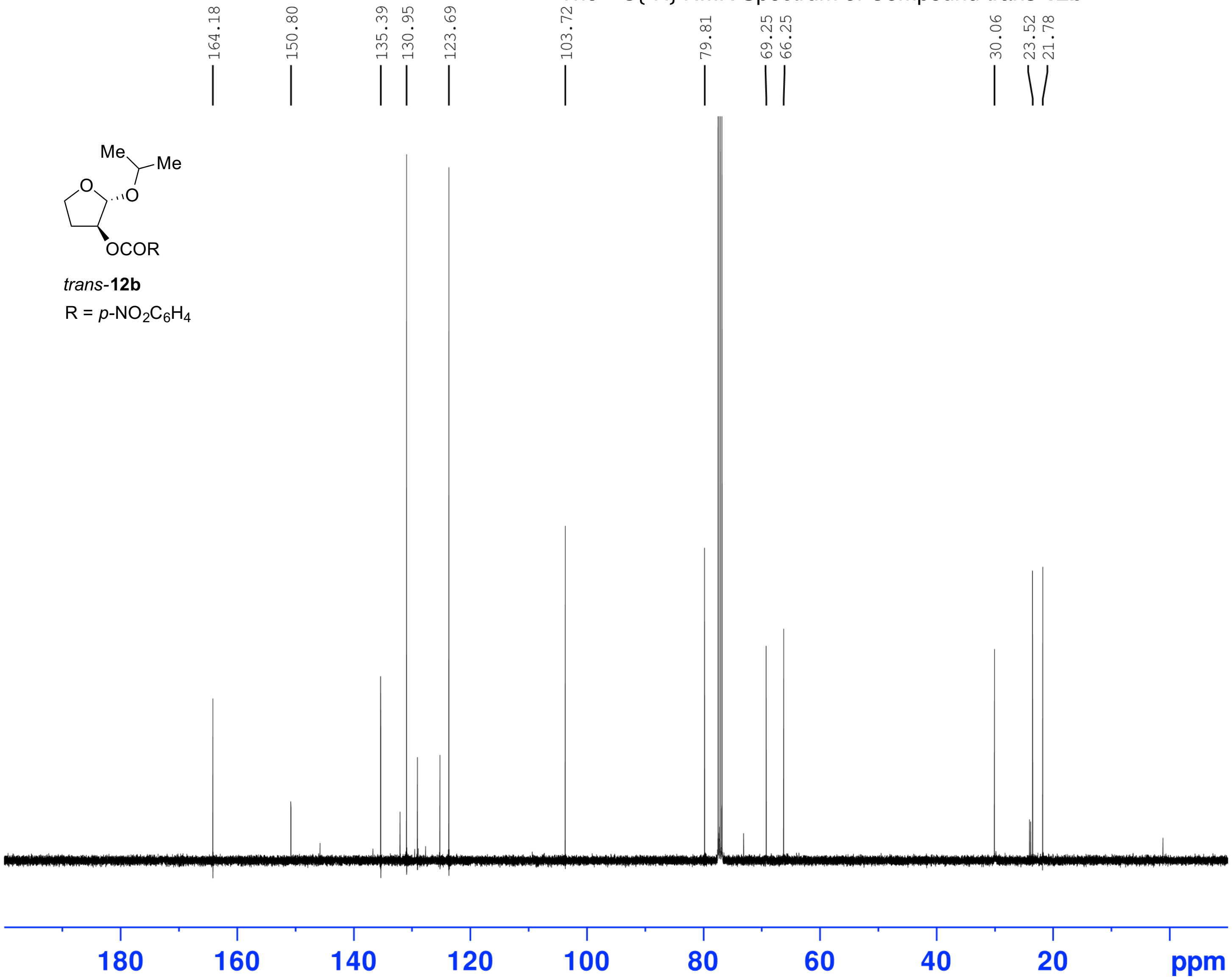

Current Data Parameters
NAME YC-4-163-COLUMN
EXPNO 2
PROCNO 1

F2 - Acquisition Parameters
Date_ 20240417
Time 5.29 h
INSTRUM spect
PROBHD Z150354_0001 (
PULPROG zgpg30
TD 65356
SOLVENT CDCl3
NS 128
DS 4
SWH 24038.461 Hz
FIDRES 0.735616 Hz
AQ 1.3594048 sec
RG 29.21
DW 20.800 usec
DE 25.00 usec
TE 298.0 K
D1 2.00000000 sec
D11 0.03000000 sec
TD0 1
SFO1 100.6655806 MHz
NUC1 13C
P1 10.00 usec
PLW1 18.70700073 W
SFO2 400.3016012 MHz
NUC2 1H
CPDPRG[2 waltz16
PCPD2 80.00 usec
PLW2 4.64209986 W
PLW12 0.10445000 W
PLW13 0.05245300 W

F2 - Processing parameters
SI 131072
SF 100.6555031 MHz
WDW EM
SSB 0
LB 0 Hz
GB 0
PC 1.40

The $^{13}C\{^1H\}$ NMR Spectrum of Compound *trans*-**13b**

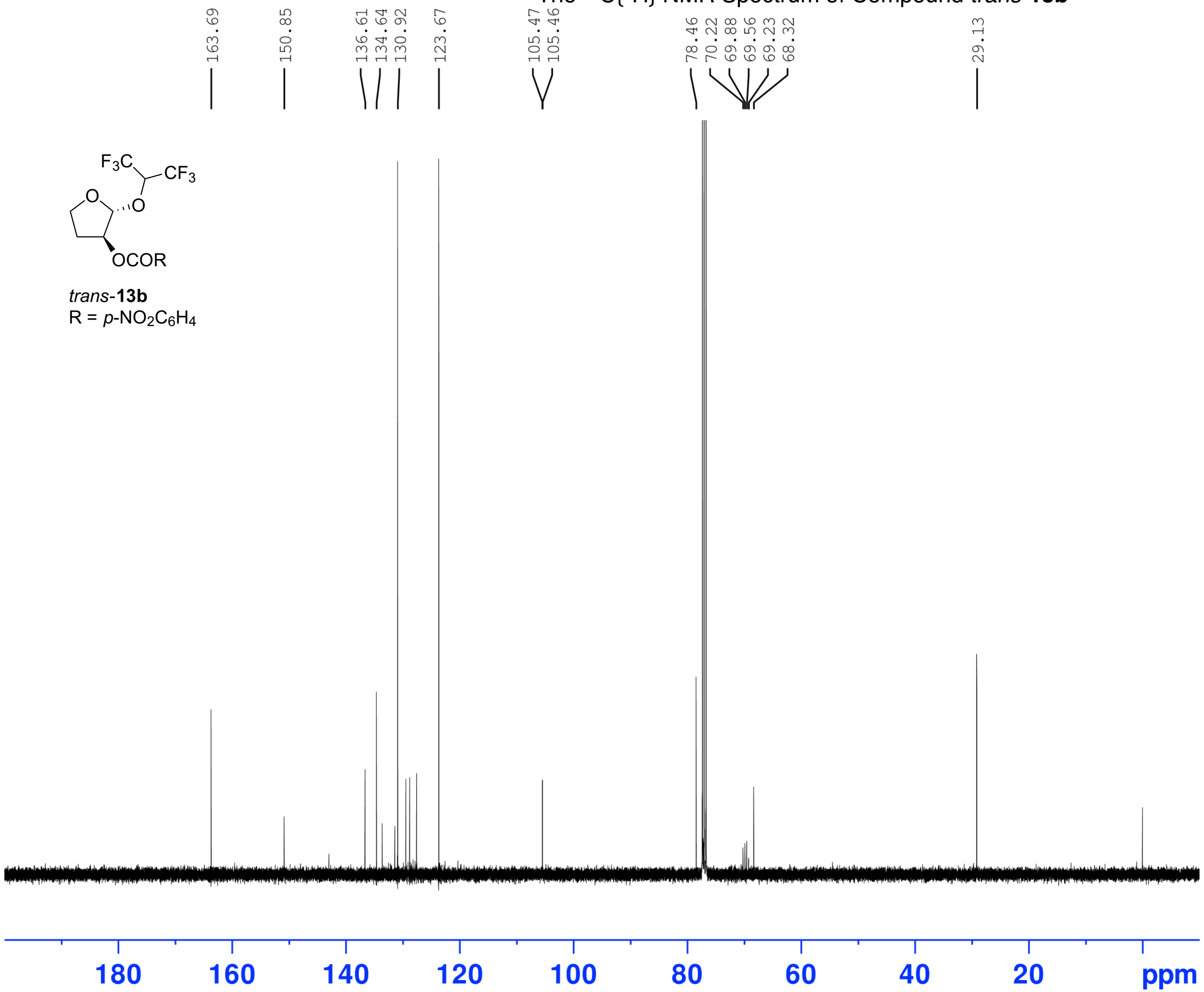

trans-**13b**
R = *p*-$NO_2C_6H_4$

Current Data Parameters
NAME YC-4-148-col2
EXPNO 2
PROCNO 1

F2 - Acquisition Parameters
Date_ 20240330
Time 1.19 h
INSTRUM spect
PROBHD Z150354_0001 (
PULPROG zgpg30
TD 65356
SOLVENT CDCl3
NS 128
DS 4
SWH 24038.461 Hz
FIDRES 0.735616 Hz
AQ 1.3594048 sec
RG 51.78
DW 20.800 usec
DE 25.00 usec
TE 298.0 K
D1 2.00000000 sec
D11 0.03000000 sec
TD0 1
SFO1 100.6655806 MHz
NUC1 13C
P1 10.00 usec
PLW1 18.70700073 W
SFO2 400.3016012 MHz
NUC2 1H
CPDPRG[2 waltz16
PCPD2 80.00 usec
PLW2 4.64209986 W
PLW12 0.10445000 W
PLW13 0.05245300 W

F2 - Processing parameters
SI 131072
SF 100.6555150 MHz
WDW EM
SSB 0
LB 0 Hz
GB 0
PC 1.40

The ¹H NMR Spectrum of Compound **15b**

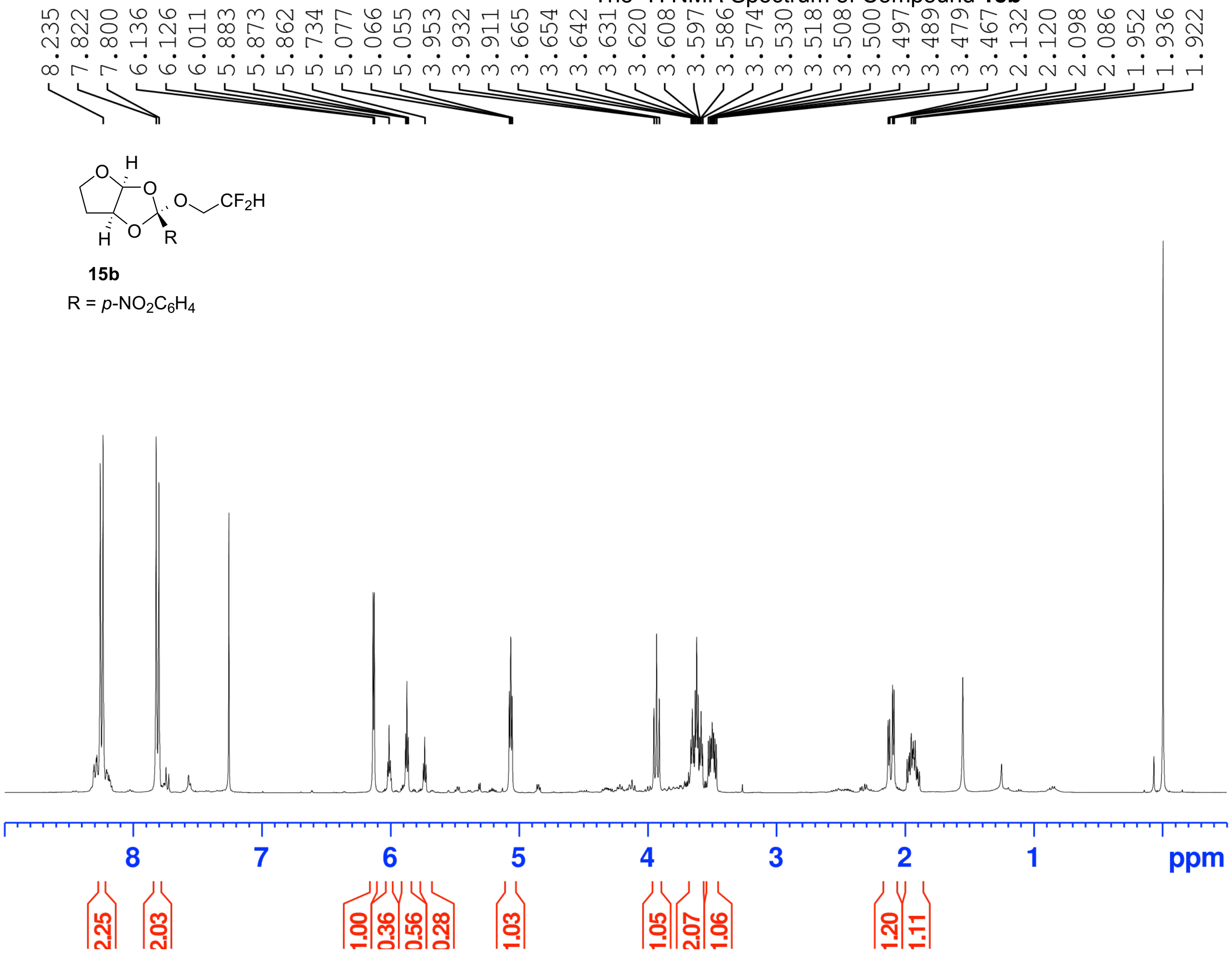

Current Data Parameters
NAME YC-4-136-Fr41-45
EXPNO 2
PROCNO 1

F2 - Acquisition Parameters
Date_ 20240305
Time 0.37 h
INSTRUM spect
PROBHD Z150354_0001 (
PULPROG zg30
TD 65536
SOLVENT CDCl3
NS 16
DS 2
SWH 8012.820 Hz
FIDRES 0.244532 Hz
AQ 4.0894465 sec
RG 125.92
DW 62.400 usec
DE 30.00 usec
TE 298.0 K
D1 1.00000000 sec
TD0 1
SFO1 400.3024719 MHz
NUC1 1H
P1 12.00 usec
PLW1 4.64209986 W

F2 - Processing parameters
SI 65536
SF 400.3000106 MHz
WDW EM
SSB 0
LB 0.30 Hz
GB 0
PC 1.00

The $^{13}C\{^{1}H\}$ NMR Spectrum of Compound **16b**

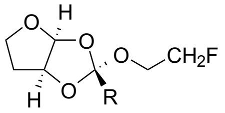

16b
R = *p*-$NO_2C_6H_4$

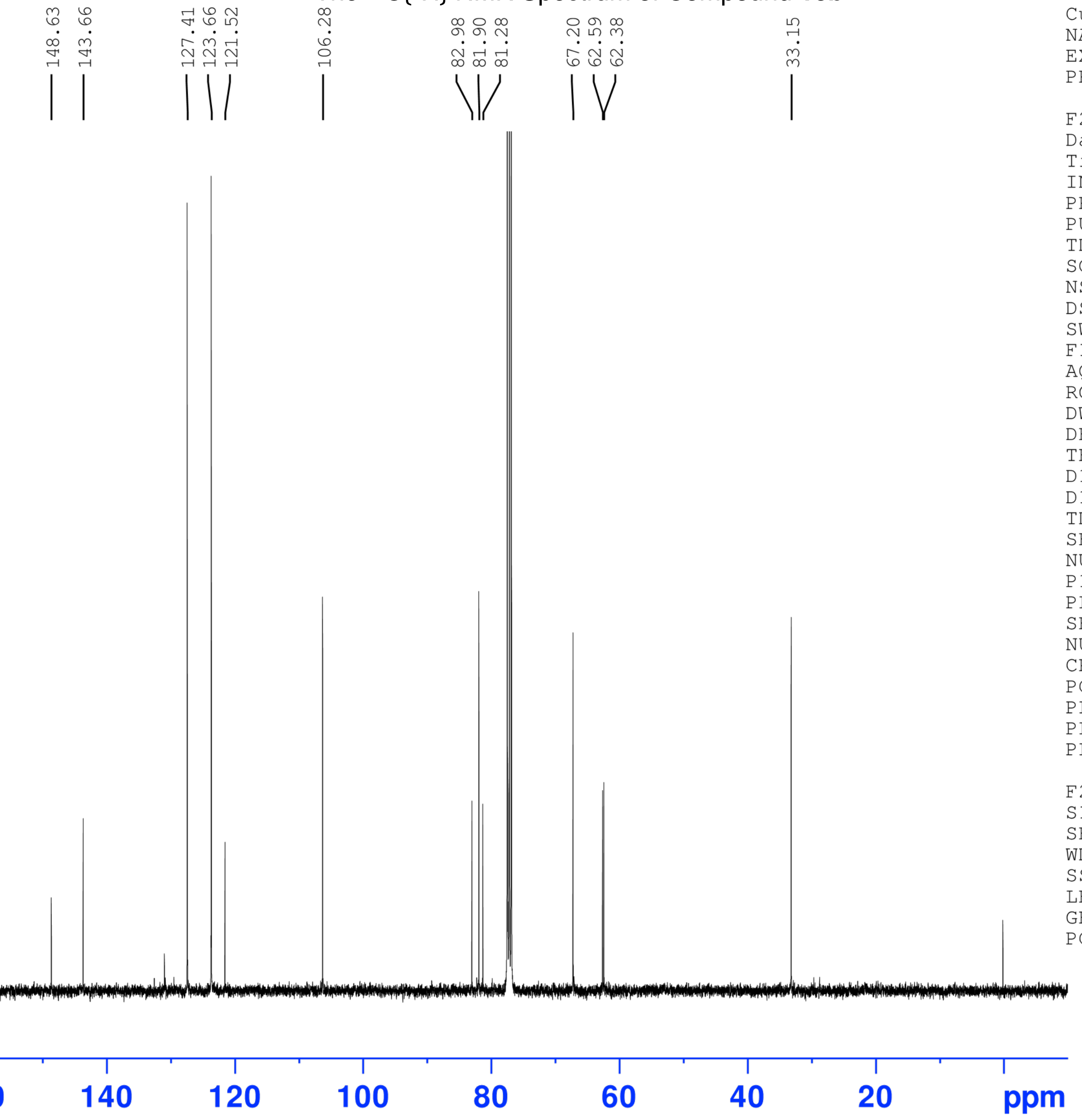


```
Current Data Parameters
NAME        YC-4-147-col3-Fr31-43
EXPNO                     3
PROCNO                    1

F2 - Acquisition Parameters
Date_              20240321
Time                   1.23 h
INSTRUM               spect
PROBHD      Z150354_0001 (
PULPROG             zgpg30
TD                   65356
SOLVENT              CDCl3
NS                     128
DS                       4
SWH              24038.461 Hz
FIDRES            0.735616 Hz
AQ               1.3594048 sec
RG                   25.77
DW                  20.800 usec
DE                   25.00 usec
TE                   298.0 K
D1              2.00000000 sec
D11             0.03000000 sec
TD0                      1
SFO1           100.6655806 MHz
NUC1                   13C
P1                   10.00 usec
PLW1           18.70700073 W
SFO2           400.3016012 MHz
NUC2                    1H
CPDPRG[2           waltz16
PCPD2                80.00 usec
PLW2            4.64209986 W
PLW12           0.10445000 W
PLW13           0.05245300 W

F2 - Processing parameters
SI                  131072
SF             100.6555045 MHz
WDW                     EM
SSB                      0
LB                    1.00 Hz
GB                       0
PC                    1.40
```

The $^{13}C\{^1H\}$ NMR Spectrum of Compound **16b**

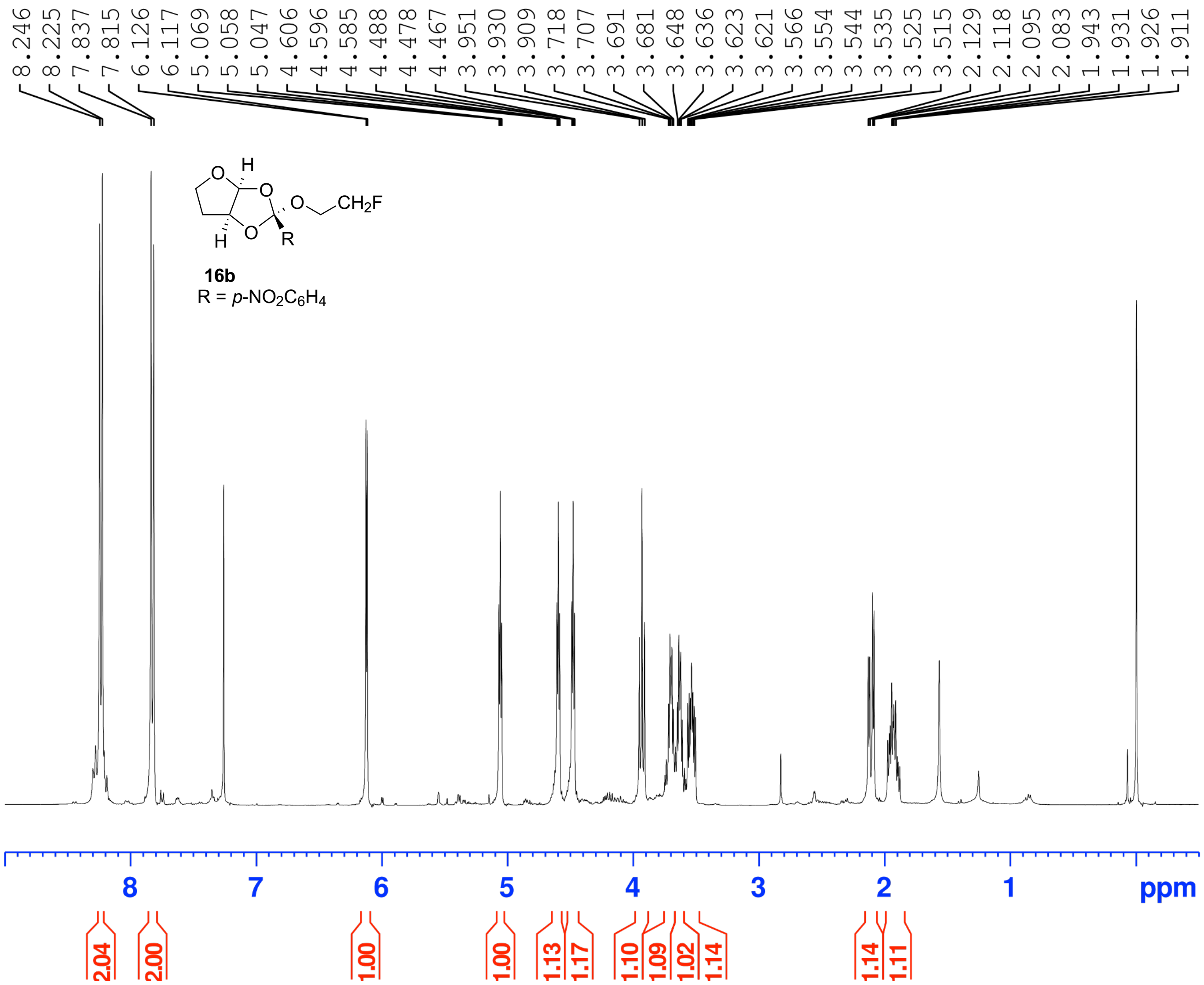


```
Current Data Parameters
NAME      YC-4-147-col3-Fr31-4[illegible]
EXPNO                    1
PROCNO                   1

F2 - Acquisition Parameters
Date_             20240321
Time                1.02 h
INSTRUM              spect
PROBHD    Z150354_0001 (
PULPROG               zg30
TD                   65536
SOLVENT              CDCl3
NS                      16
DS                       2
SWH            8012.820 Hz
FIDRES         0.244532 Hz
AQ            4.0894465 sec
RG                  117.47
DW              62.400 usec
DE               30.00 usec
TE                298.0 K
D1           1.00000000 sec
TD0                      1
SFO1        400.3024719 MHz
NUC1                    1H
P1               12.00 usec
PLW1          4.64209986 W

F2 - Processing parameters
SI                   65536
SF          400.3000101 MHz
WDW                     EM
SSB                      0
LB                 0.30 Hz
GB                       0
PC                    1.00
```

The ^{1}H NMR Spectrum of Compound **17b**

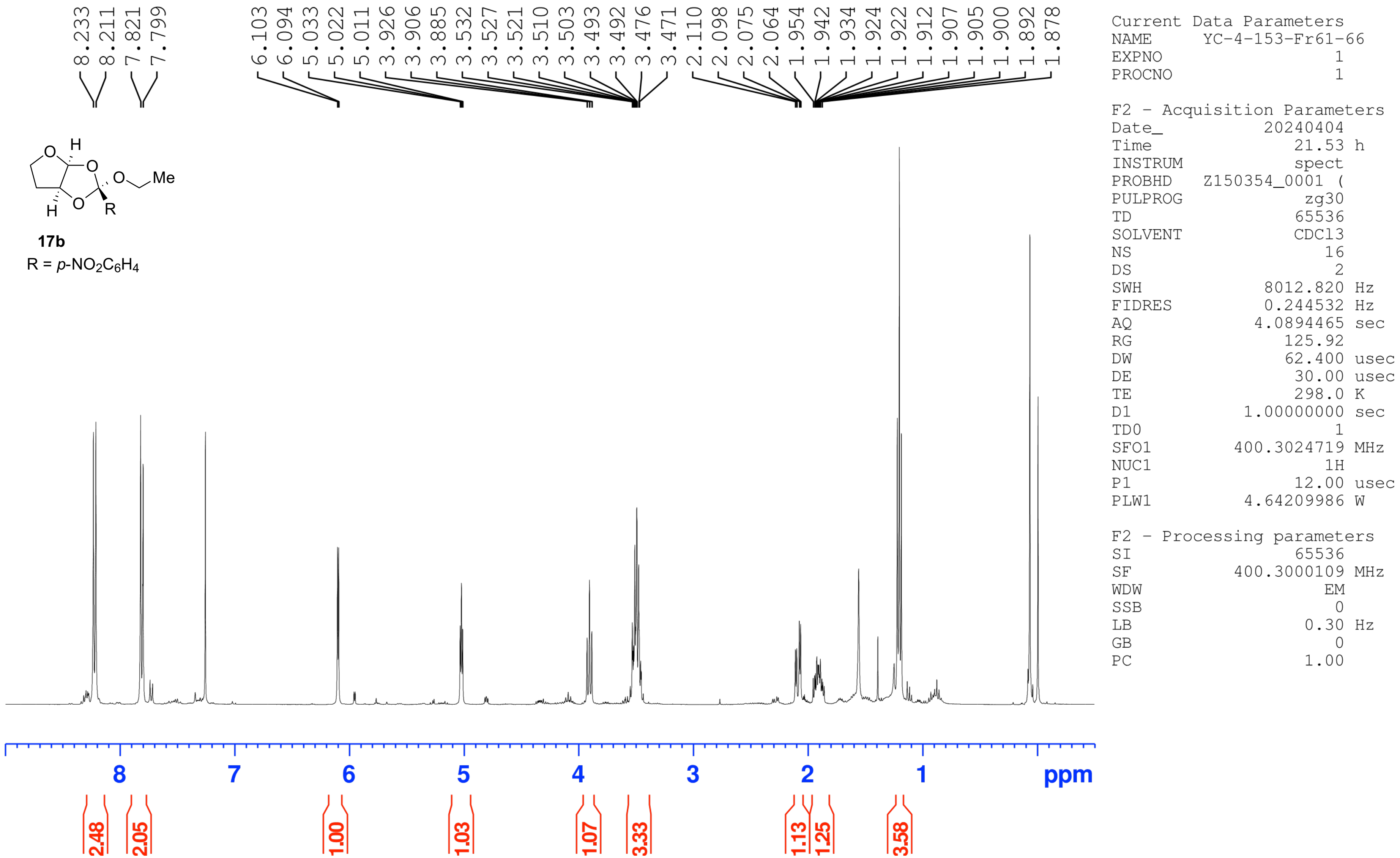

17b

R = p-$NO_2C_6H_4$

The $^{13}C\{^{1}H\}$ NMR Spectrum of Compound **18b**

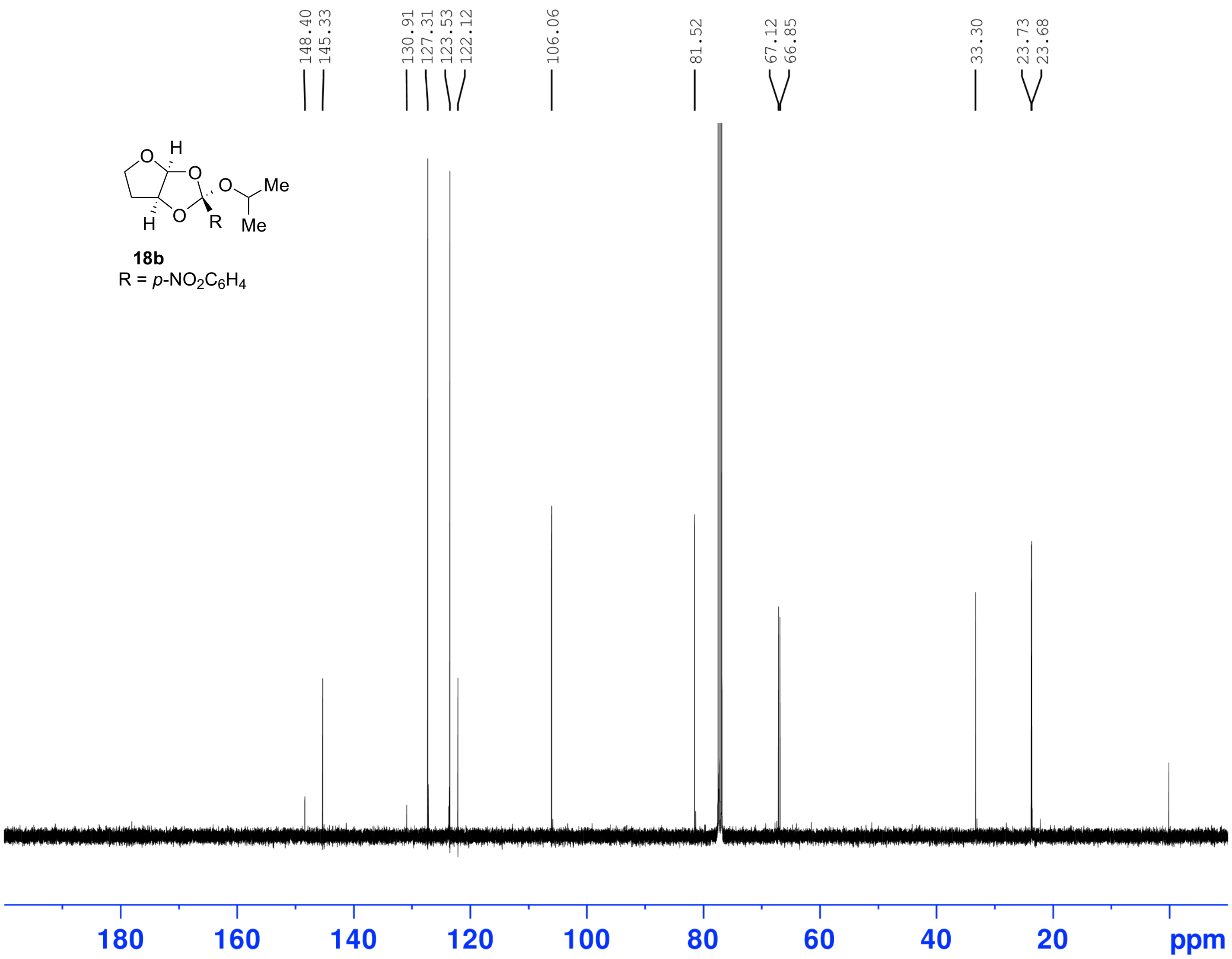


```
Current Data Parameters
NAME      YC-4-154-Fr43-62
EXPNO                    2
PROCNO                   1

F2 - Acquisition Parameters
Date_             20240405
Time                1.09 h
INSTRUM              spect
PROBHD    Z150354_0001 (
PULPROG            zgpg30
TD                  65356
SOLVENT             CDCl3
NS                    128
DS                      4
SWH             24038.461 Hz
FIDRES           0.735616 Hz
AQ              1.3594048 sec
RG                  51.78
DW                 20.800 usec
DE                  25.00 usec
TE                  298.0 K
D1             2.00000000 sec
D11            0.03000000 sec
TD0                     1
SFO1          100.6655806 MHz
NUC1                  13C
P1                  10.00 usec
PLW1          18.70700073 W
SFO2          400.3016012 MHz
NUC2                   1H
CPDPRG[2          waltz16
PCPD2               80.00 usec
PLW2           4.64209986 W
PLW12          0.10445000 W
PLW13          0.05245300 W

F2 - Processing parameters
SI                 131072
SF            100.6555013 MHz
WDW                    EM
SSB                     0
LB                      0 Hz
GB                      0
PC                   1.40
```

The ^{1}H NMR Spectrum of Compound *trans*-**18b**

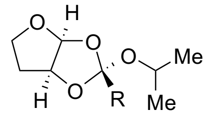

18b
R = p-$NO_2C_6H_4$

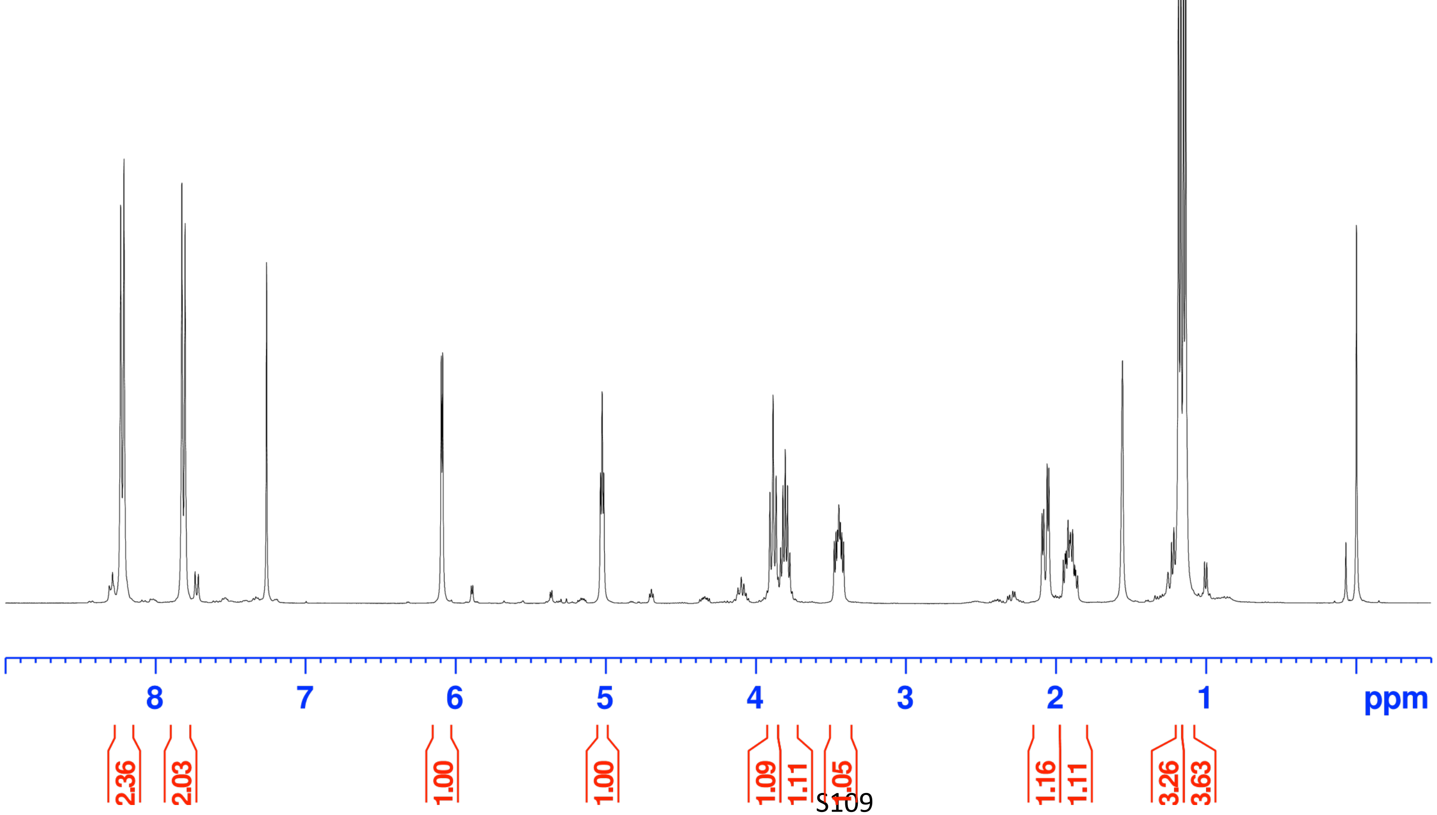

Current Data Parameters
NAME YC-4-154-Fr43-62
EXPNO 1
PROCNO 1

F2 - Acquisition Parameters
Date_ 20240404
Time 22.12 h
INSTRUM spect
PROBHD Z150354_0001 (
PULPROG zg30
TD 65536
SOLVENT CDCl3
NS 16
DS 2
SWH 8012.820 Hz
FIDRES 0.244532 Hz
AQ 4.0894465 sec
RG 125.92
DW 62.400 usec
DE 30.00 usec
TE 298.0 K
D1 1.00000000 sec
TD0 1
SFO1 400.3024719 MHz
NUC1 1H
P1 12.00 usec
PLW1 4.64209986 W

F2 - Processing parameters
SI 65536
SF 400.3000094 MHz
WDW EM
SSB 0
LB 0.30 Hz
GB 0
PC 1.00

The ^{1}H NMR Spectrum of Compound *cis*-**22a**

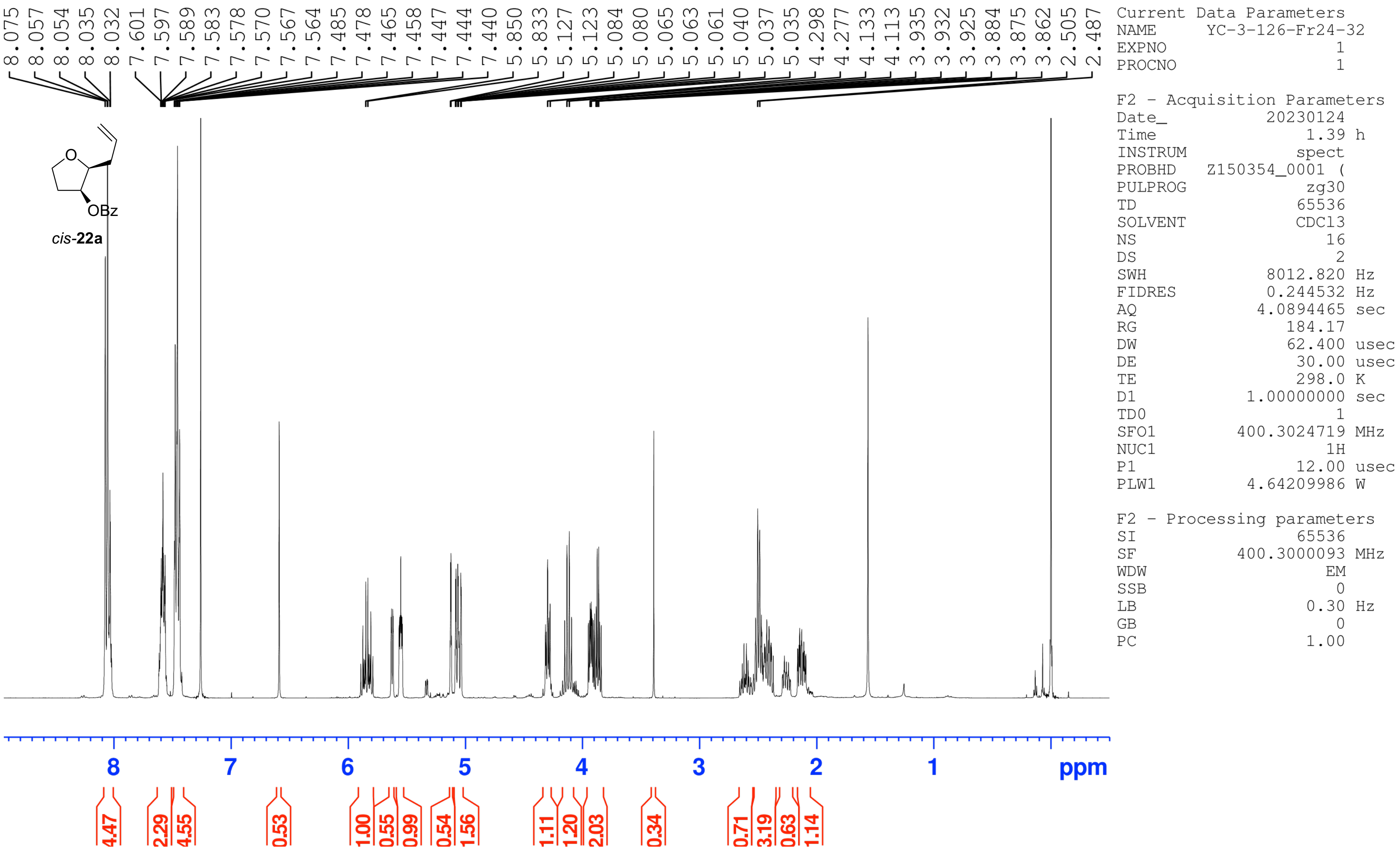

The $^{13}C\{^{1}H\}$ NMR Spectrum of Compound *cis*-**22a**

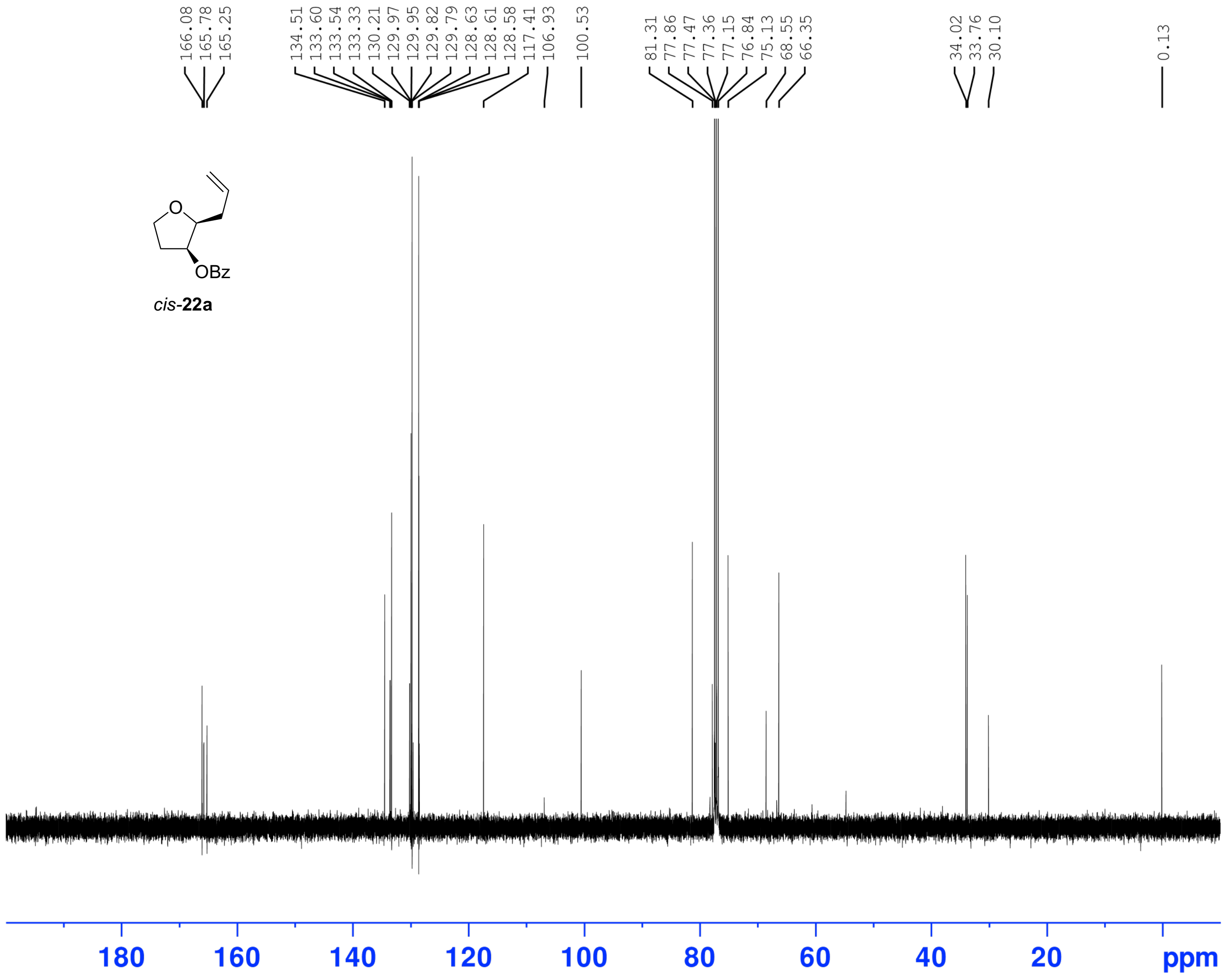

Current Data Parameters
NAME YC-3-126-Fr24-32
EXPNO 2
PROCNO 1

F2 - Acquisition Parameters
Date_ 20230124
Time 2.24 h
INSTRUM spect
PROBHD Z150354_0001 (
PULPROG zgpg30
TD 65356
SOLVENT CDCl3
NS 128
DS 4
SWH 24038.461 Hz
FIDRES 0.735616 Hz
AQ 1.3594048 sec
RG 45.21
DW 20.800 usec
DE 25.00 usec
TE 298.0 K
D1 2.00000000 sec
D11 0.03000000 sec
TD0 1
SFO1 100.6655806 MHz
NUC1 13C
P1 10.00 usec
PLW1 17.99699974 W
SFO2 400.3016012 MHz
NUC2 1H
CPDPRG[2 waltz16
PCPD2 80.00 usec
PLW2 4.64209986 W
PLW12 0.10445000 W
PLW13 0.05245300 W

F2 - Processing parameters
SI 131072
SF 100.6555018 MHz
WDW EM
SSB 0
LB 0 Hz
GB 0
PC 1.40

The ^{1}H NMR Spectrum of Compound *trans*-**22a**

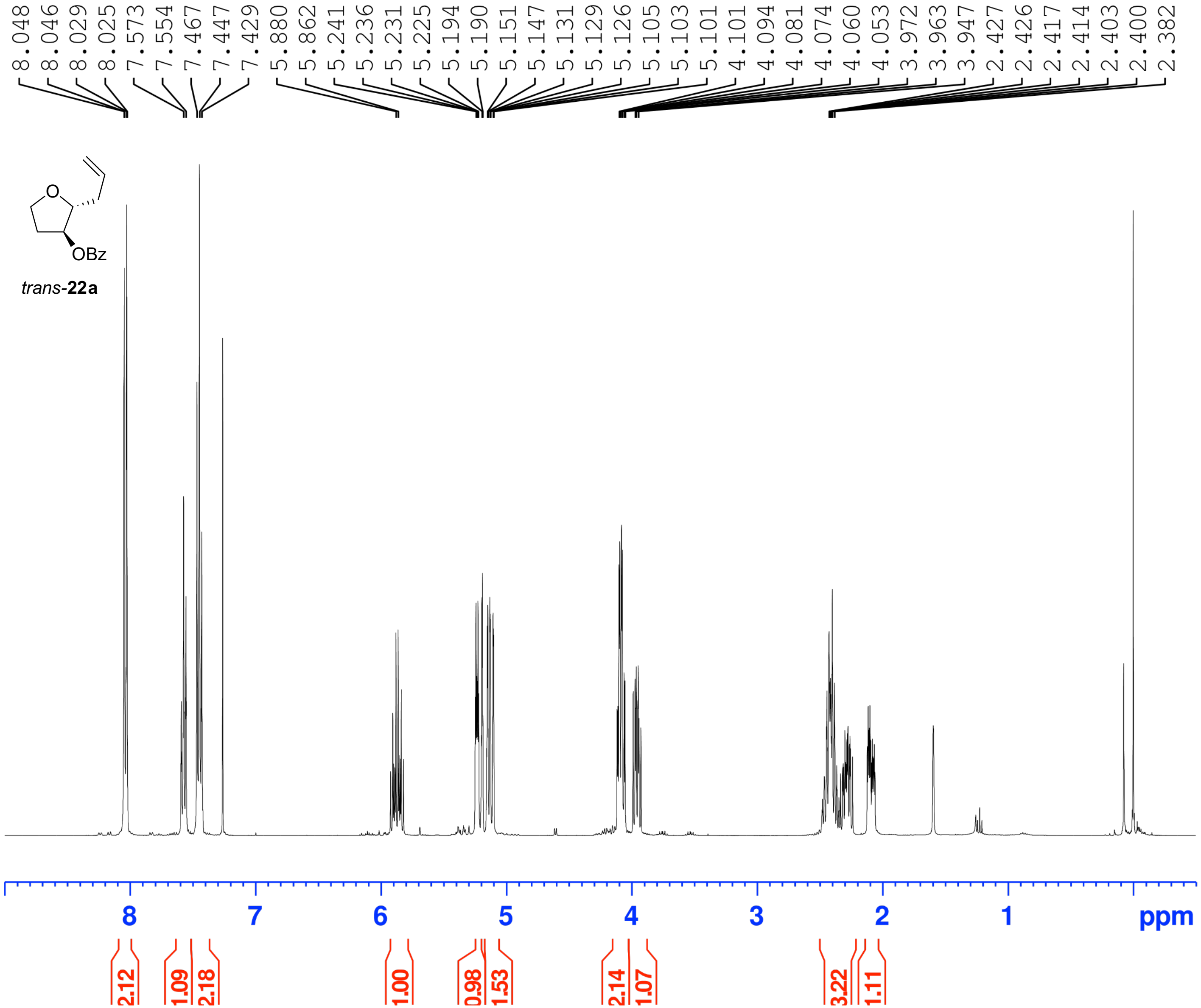


```
Current Data Parameters
NAME        YC-3-140-Fr15.16
EXPNO                      1
PROCNO                     1

F2 - Acquisition Parameters
Date_               20230201
Time                  0.55 h
INSTRUM                spect
PROBHD      Z150354_0001 (
PULPROG                 zg30
TD                     65536
SOLVENT                CDCl3
NS                        16
DS                         2
SWH                 8012.820 Hz
FIDRES              0.244532 Hz
AQ                 4.0894465 sec
RG                      92.4
DW                    62.400 usec
DE                     30.00 usec
TE                     298.0 K
D1                1.00000000 sec
TD0                        1
SFO1             400.3024719 MHz
NUC1                      1H
P1                     12.00 usec
PLW1              4.64209986 W

F2 - Processing parameters
SI                     65536
SF               400.3000090 MHz
WDW                       EM
SSB                        0
LB                      0.30 Hz
GB                         0
PC                      1.00
```

The $^{13}C\{^1H\}$ NMR Spectrum of Compound *trans*-**22a**

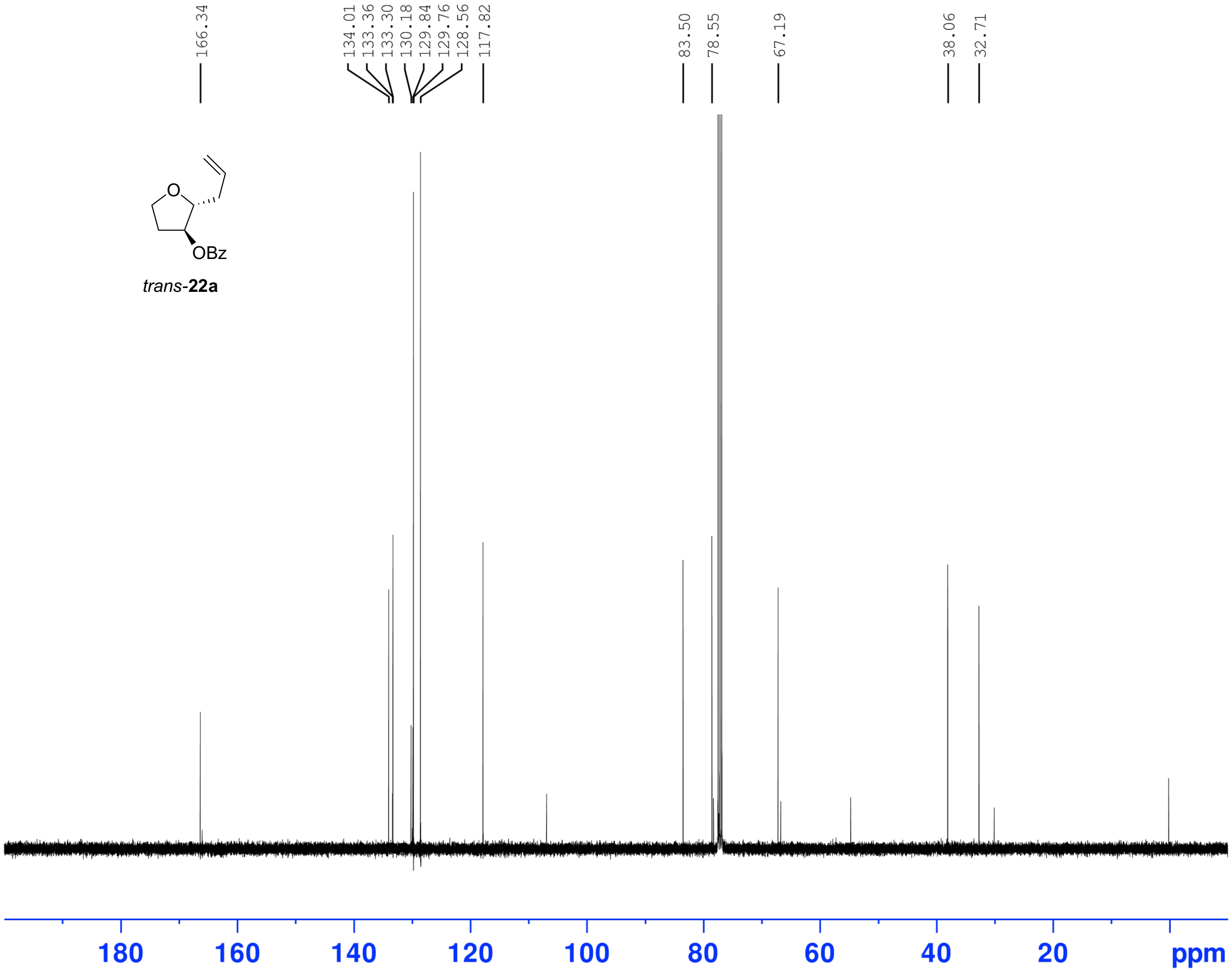

Current Data Parameters
NAME YC-3-126-Fr16-23
EXPNO 2
PROCNO 1

F2 - Acquisition Parameters
Date_ 20230124
Time 1.34 h
INSTRUM spect
PROBHD Z150354_0001 (
PULPROG zgpg30
TD 65356
SOLVENT CDCl3
NS 128
DS 4
SWH 24038.461 Hz
FIDRES 0.735616 Hz
AQ 1.3594048 sec
RG 29.21
DW 20.800 usec
DE 25.00 usec
TE 298.0 K
D1 2.00000000 sec
D11 0.03000000 sec
TD0 1
SFO1 100.6655806 MHz
NUC1 13C
P1 10.00 usec
PLW1 17.99699974 W
SFO2 400.3016012 MHz
NUC2 1H
CPDPRG[2 waltz16
PCPD2 80.00 usec
PLW2 4.64209986 W
PLW12 0.10445000 W
PLW13 0.05245300 W

F2 - Processing parameters
SI 131072
SF 100.6555013 MHz
WDW EM
SSB 0
LB 0 Hz
GB 0
PC 1.40

The $^{13}C\{^{1}H\}$ NMR Spectrum of Compound *trans*-**S3**

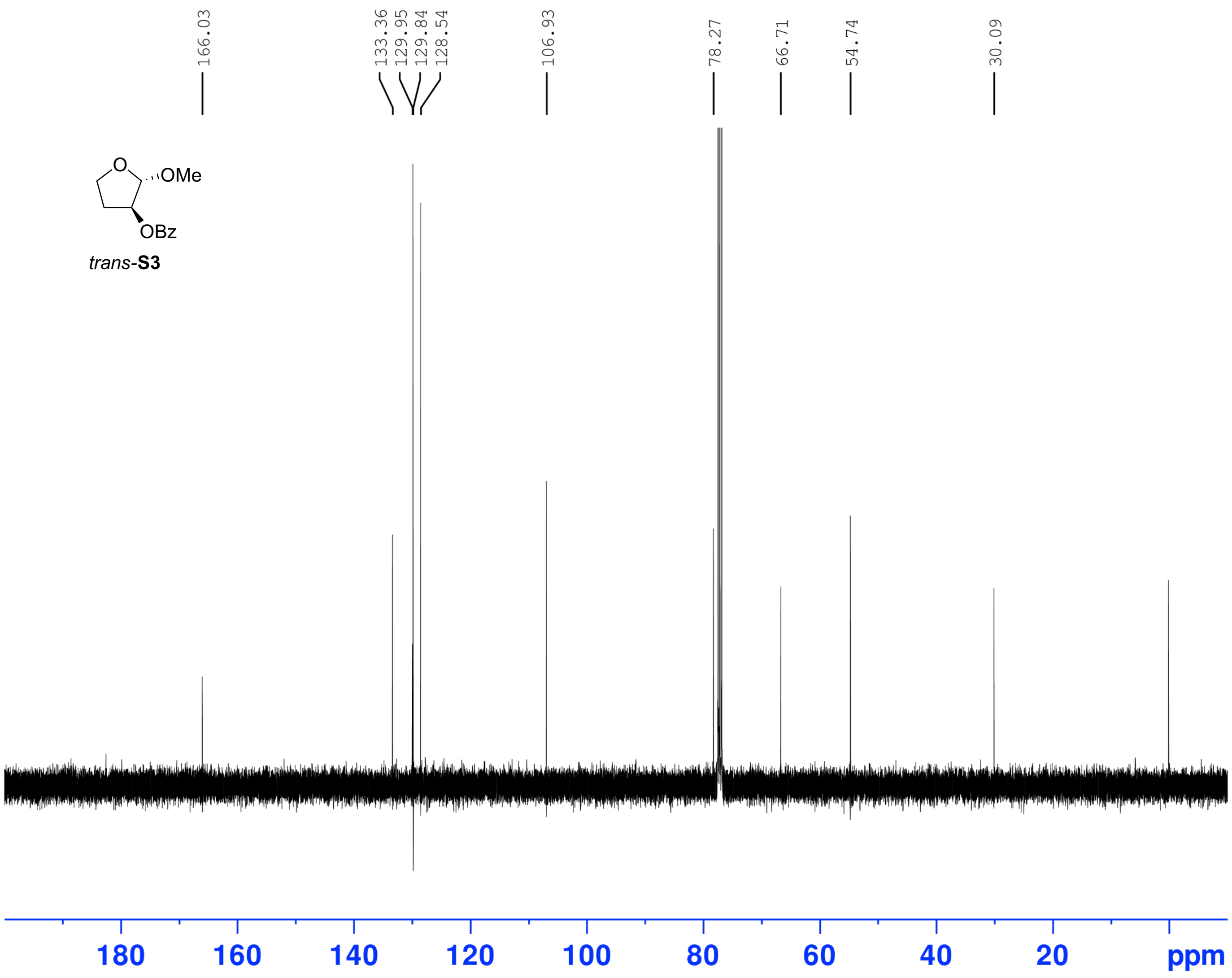

Current Data Parameters
NAME YC-4-132-COLUMN
EXPNO 2
PROCNO 1

F2 - Acquisition Parameters
Date_ 20240228
Time 3.17 h
INSTRUM spect
PROBHD Z150354_0001 (
PULPROG zgpg30
TD 65356
SOLVENT CDCl3
NS 128
DS 4
SWH 24038.461 Hz
FIDRES 0.735616 Hz
AQ 1.3594048 sec
RG 25.77
DW 20.800 usec
DE 25.00 usec
TE 298.0 K
D1 2.00000000 sec
D11 0.03000000 sec
TD0 1
SFO1 100.6655806 MHz
NUC1 13C
P1 10.00 usec
PLW1 18.70700073 W
SFO2 400.3016012 MHz
NUC2 1H
CPDPRG[2 waltz16
PCPD2 80.00 usec
PLW2 4.64209986 W
PLW12 0.10445000 W
PLW13 0.05245300 W

F2 - Processing parameters
SI 131072
SF 100.6555018 MHz
WDW EM
SSB 0
LB 0 Hz
GB 0
PC 1.40

The ^{1}H NMR Spectrum of Compound *trans*-**S3**

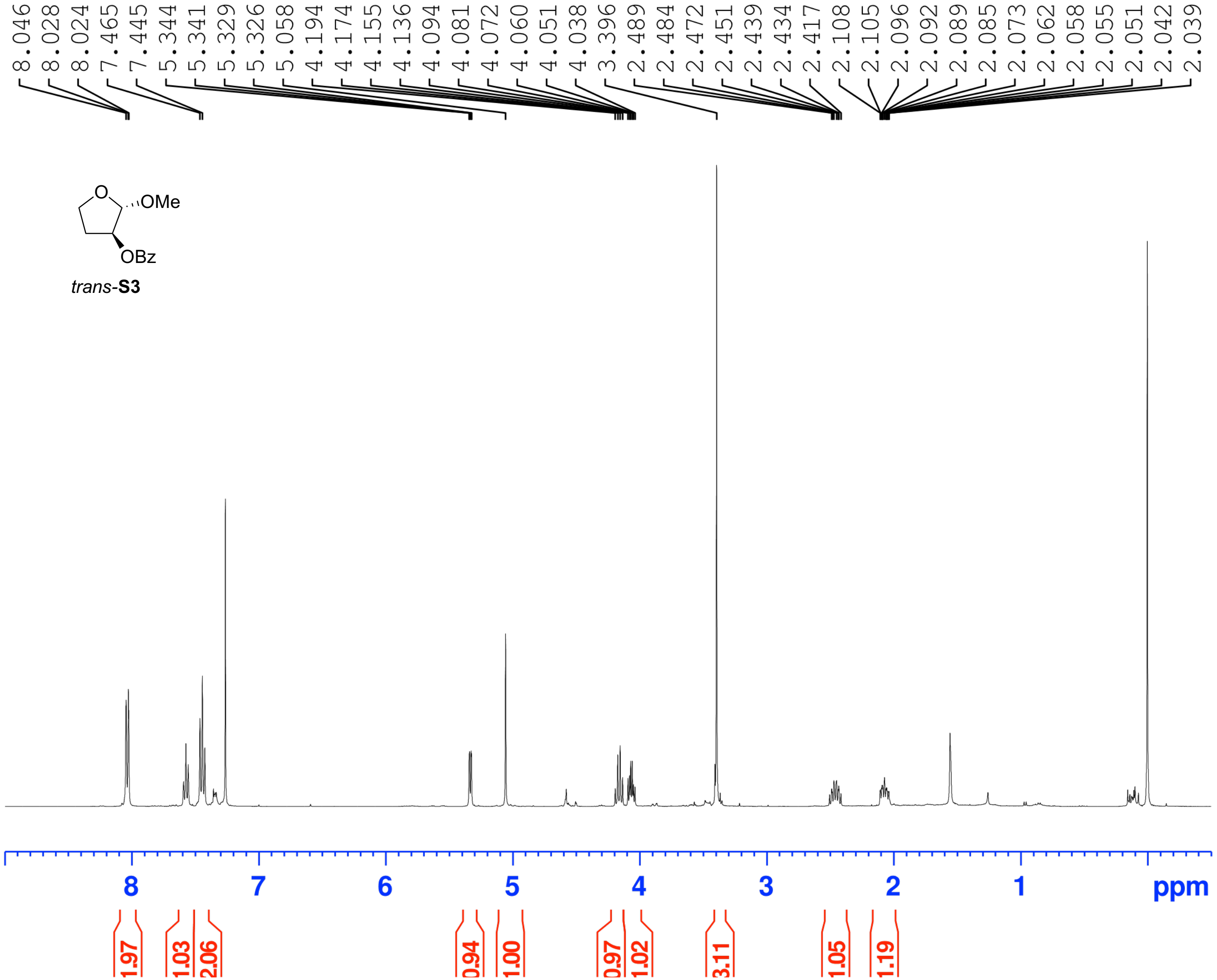


```
Current Data Parameters
NAME      YC-4-132-COLUMN
EXPNO                   1
PROCNO                  1

F2 - Acquisition Parameters
Date_            20240228
Time                 1.57 h
INSTRUM             spect
PROBHD     Z150354_0001 (
PULPROG              zg30
TD                  65536
SOLVENT             CDCl3
NS                     16
DS                      2
SWH              8012.820 Hz
FIDRES           0.244532 Hz
AQ              4.0894465 sec
RG                 141.61
DW                 62.400 usec
DE                  30.00 usec
TE                  298.0 K
D1             1.00000000 sec
TD0                     1
SFO1          400.3024719 MHz
NUC1                   1H
P1                  12.00 usec
PLW1           4.64209986 W

F2 - Processing parameters
SI                  65536
SF            400.3000077 MHz
WDW                    EM
SSB                     0
LB                   0.30 Hz
GB                      0
PC                   1.00
```

The ^{1}H NMR Spectrum of Compounds *trans*-**S4** and *cis*-**S4**

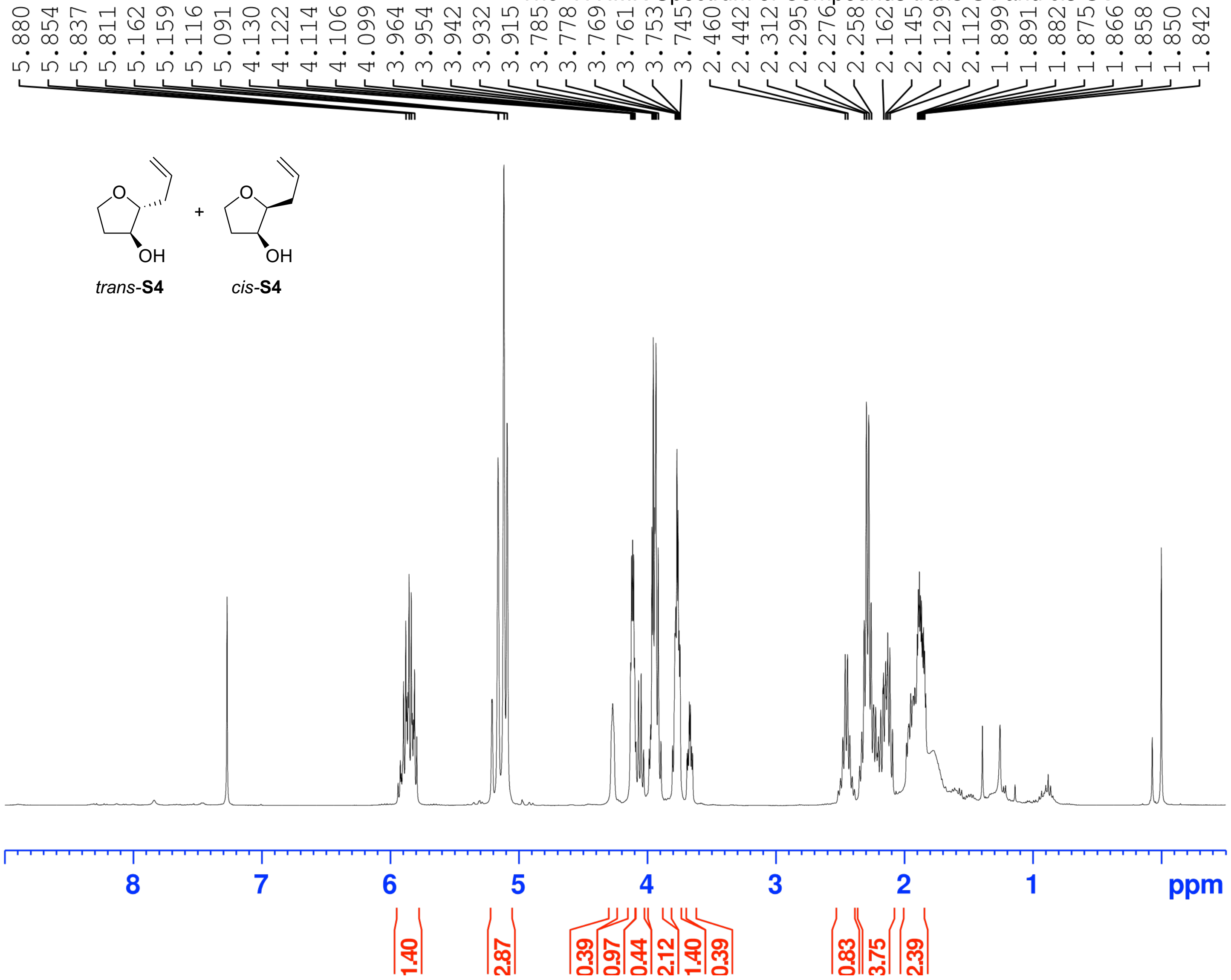

Current Data Parameters
NAME YC-3-139-COLUMN
EXPNO 1
PROCNO 1

F2 - Acquisition Parameters
Date_ 20230201
Time 0.46 h
INSTRUM spect
PROBHD Z150354_0001 (
PULPROG zg30
TD 65536
SOLVENT CDCl3
NS 16
DS 2
SWH 8012.820 Hz
FIDRES 0.244532 Hz
AQ 4.0894465 sec
RG 63.04
DW 62.400 usec
DE 30.00 usec
TE 298.0 K
D1 1.00000000 sec
TD0 1
SFO1 400.3024719 MHz
NUC1 1H
P1 12.00 usec
PLW1 4.64209986 W

F2 - Processing parameters
SI 65536
SF 400.3000050 MHz
WDW EM
SSB 0
LB 0.30 Hz
GB 0
PC 1.00

The $^{13}C\{^{1}H\}$ NMR Spectrum of Compounds *trans*-**S4** and *cis*-**S4**

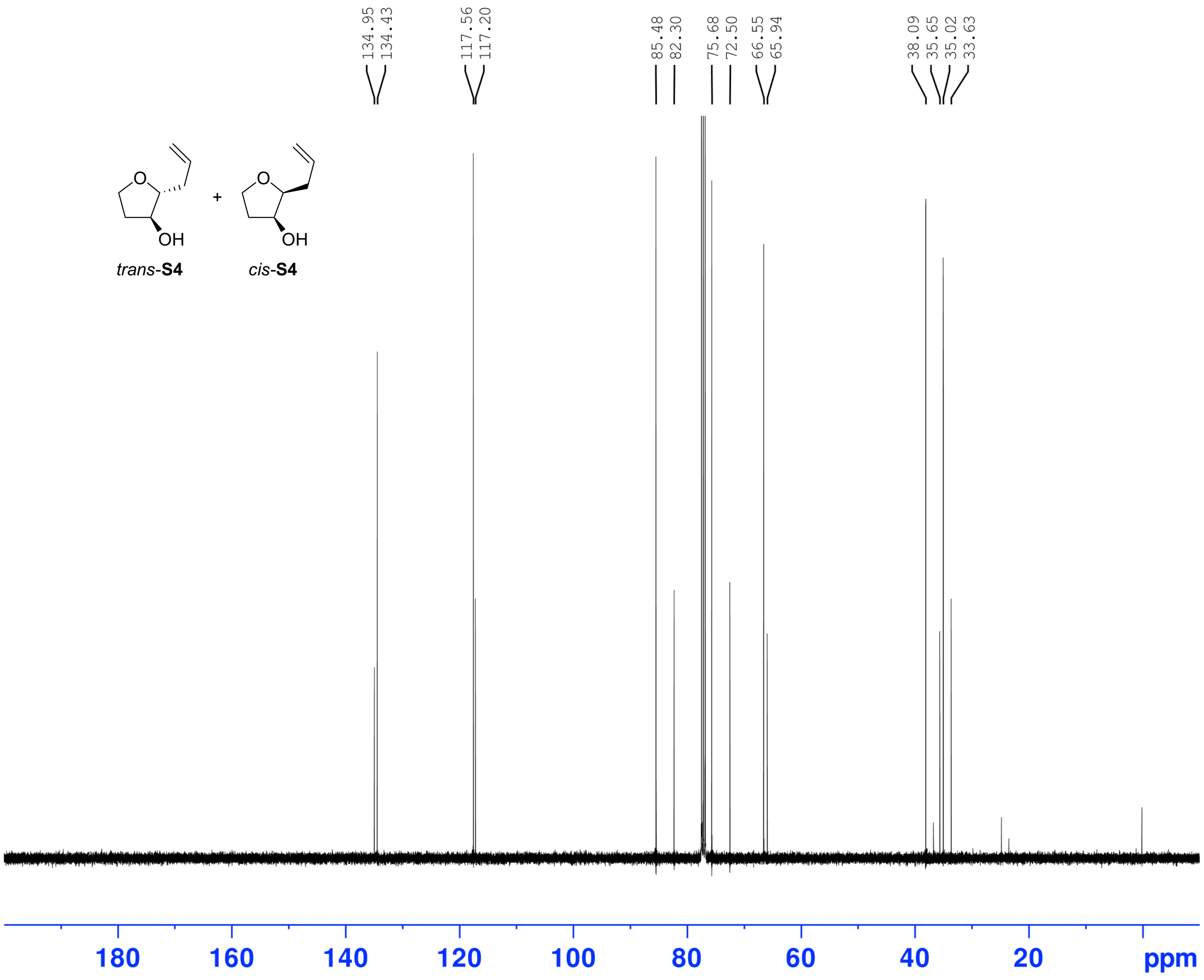


```
Current Data Parameters
NAME       YC-3-139-COLUMN
EXPNO                    2
PROCNO                   1

F2 - Acquisition Parameters
Date_             20230201
Time                1.25 h
INSTRUM              spect
PROBHD     Z150354_0001 (
PULPROG            zgpg30
TD                  65356
SOLVENT             CDCl3
NS                    128
DS                      4
SWH             24038.461 Hz
FIDRES           0.735616 Hz
AQ              1.3594048 sec
RG                  25.77
DW                 20.800 usec
DE                  25.00 usec
TE                  298.0 K
D1             2.00000000 sec
D11            0.03000000 sec
TD0                     1
SFO1          100.6655806 MHz
NUC1                  13C
P1                  10.00 usec
PLW1          17.99699974 W
SFO2          400.3016012 MHz
NUC2                   1H
CPDPRG[2          waltz16
PCPD2               80.00 usec
PLW2           4.64209986 W
PLW12          0.10445000 W
PLW13          0.05245300 W

F2 - Processing parameters
SI                 131072
SF            100.6555030 MHz
WDW                    EM
SSB                     0
LB                      0 Hz
GB                      0
PC                   1.40
```

The $^{13}C\{^1H\}$ NMR Spectrum of the Substitution Reaction of Acetal **6a** with 2,2-Difluoroethanol

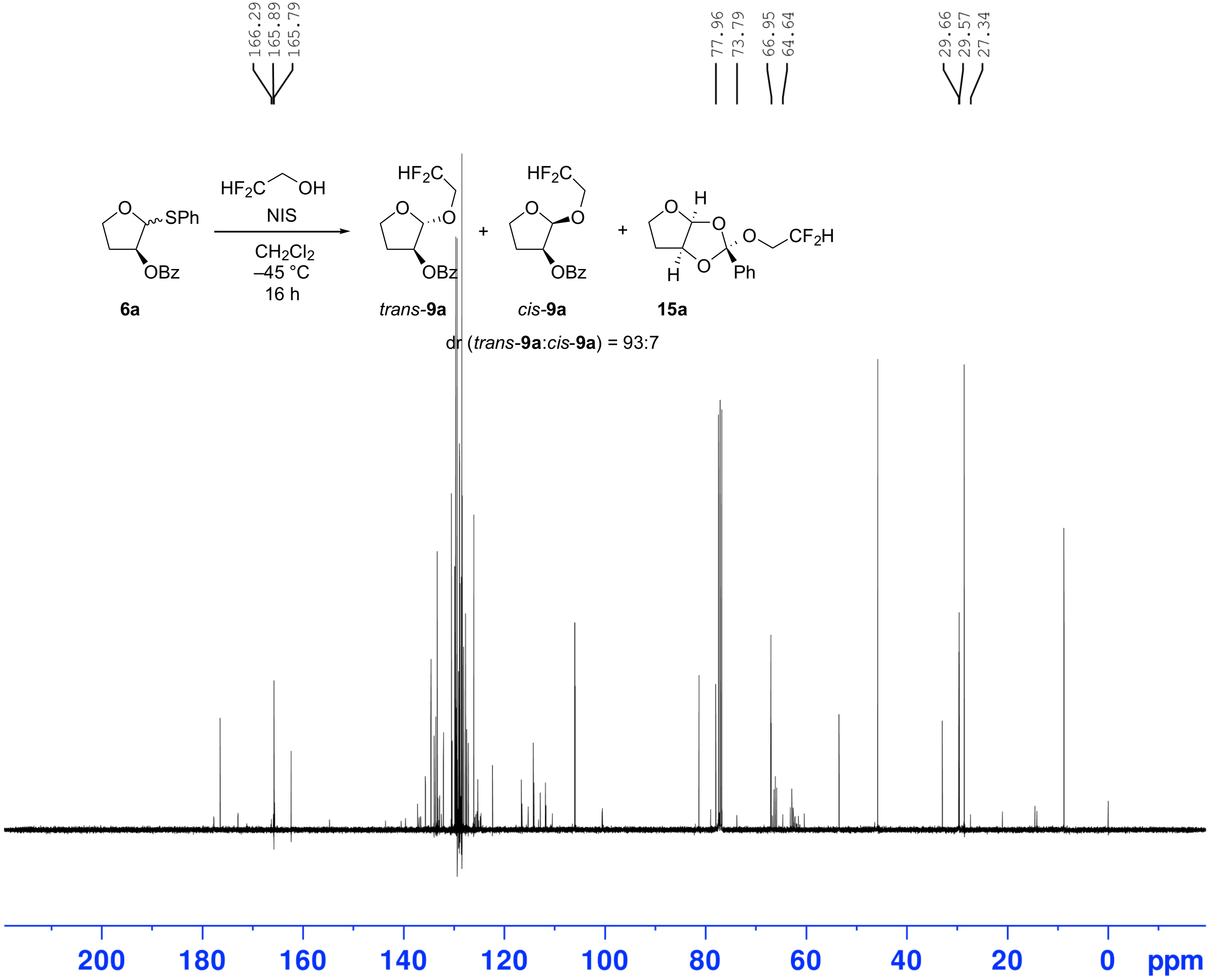


```
Current Data Parameters
NAME        YC-4-173-CRUDE
EXPNO                    2
PROCNO                   1

F2 - Acquisition Parameters
Date_             20240503
Time                  2.13 h
INSTRUM              spect
PROBHD    Z150354_0001 (
PULPROG            zgpg30
TD                  65356
SOLVENT             CDCl3
NS                    128
DS                      4
SWH             24038.461 Hz
FIDRES           0.735616 Hz
AQ              1.3594048 sec
RG                   31.4
DW                 20.800 usec
DE                  25.00 usec
TE                  298.0 K
D1             2.00000000 sec
D11            0.03000000 sec
TD0                     1
SFO1         100.6655806 MHz
NUC1                  13C
P1                  10.00 usec
PLW1          18.70700073 W
SFO2         400.3016012 MHz
NUC2                   1H
CPDPRG[2          waltz16
PCPD2               80.00 usec
PLW2           4.64209986 W
PLW12          0.10445000 W
PLW13          0.05245300 W

F2 - Processing parameters
SI                 131072
SF           100.6555182 MHz
WDW                    EM
SSB                     0
LB                      0 Hz
GB                      0
PC                   1.40
```

The $^{13}C\{^1H\}$ NMR Spectrum of the Substitution Reaction of Acetal **6b** with 2,2-Difluoroethanol

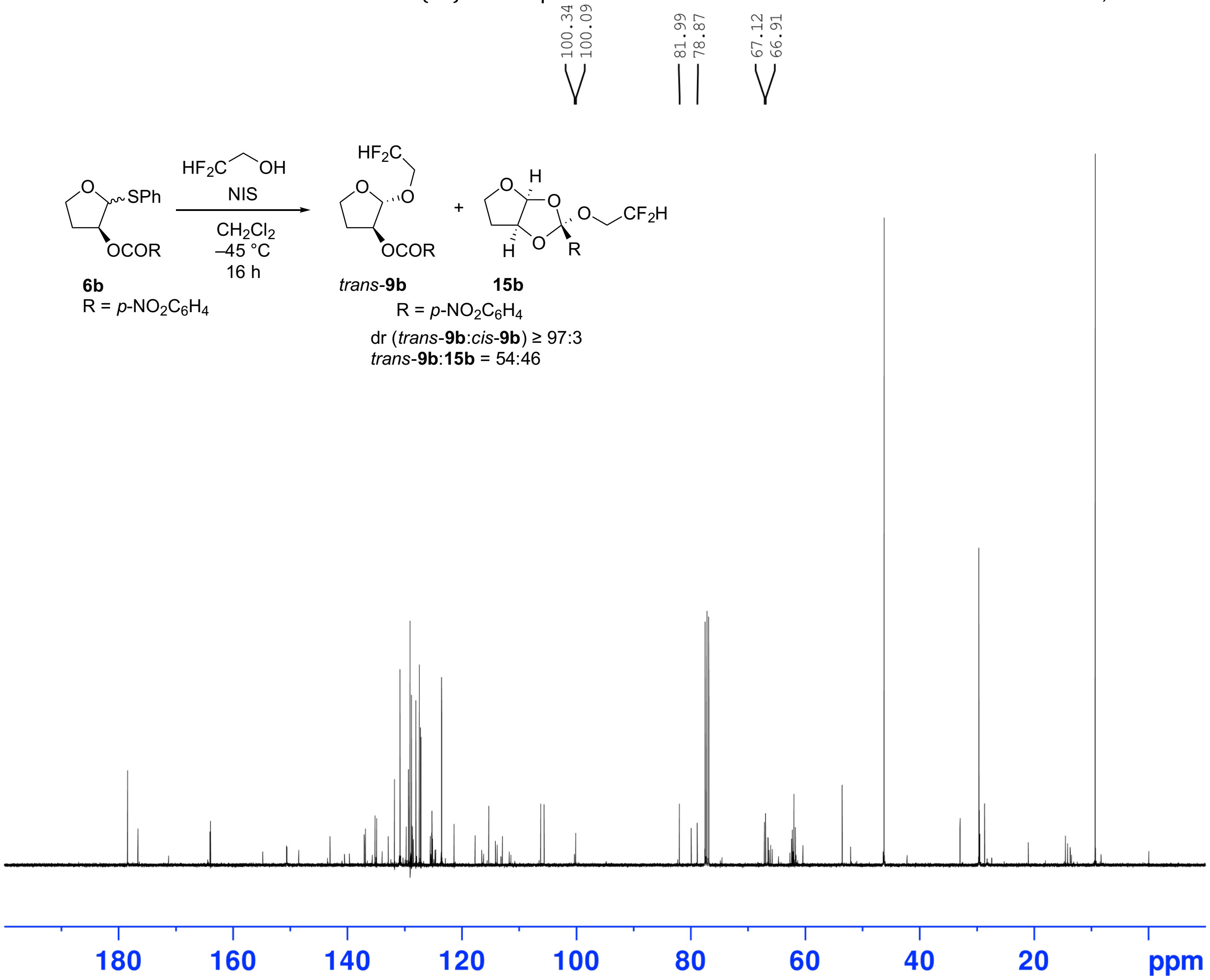


```
Current Data Parameters
NAME        YC-4-136-CRUDE
EXPNO                    2
PROCNO                   1

F2 - Acquisition Parameters
Date_             20240301
Time                  7.00 h
INSTRUM              spect
PROBHD     Z150354_0001 (
PULPROG            zgpg30
TD                   65356
SOLVENT              CDCl3
NS                     128
DS                       4
SWH              24038.461 Hz
FIDRES            0.735616 Hz
AQ               1.3594048 sec
RG                   45.21
DW                  20.800 usec
DE                   25.00 usec
TE                   298.0 K
D1              2.00000000 sec
D11             0.03000000 sec
TD0                      1
SFO1           100.6655806 MHz
NUC1                   13C
P1                   10.00 usec
PLW1           18.70700073 W
SFO2           400.3016012 MHz
NUC2                    1H
CPDPRG[2           waltz16
PCPD2                80.00 usec
PLW2            4.64209986 W
PLW12           0.10445000 W
PLW13           0.05245300 W

F2 - Processing parameters
SI                  131072
SF             100.6555175 MHz
WDW                     EM
SSB                      0
LB                       0 Hz
GB                       0
PC                    1.40
```

The $^{13}C\{^1H\}$ NMR Spectrum of the Substitution Reaction of Acetal **6a** with 2-Fluoroethanol

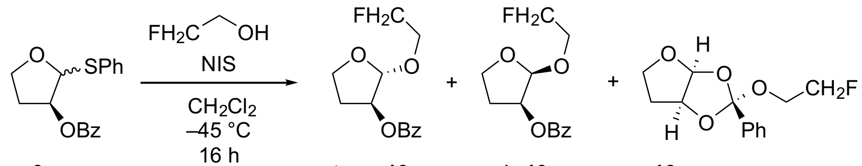

6a *trans*-**10a** *cis*-**10a** **16a**

dr (*trans*-**10a**:*cis*-**10a**) = 65:35

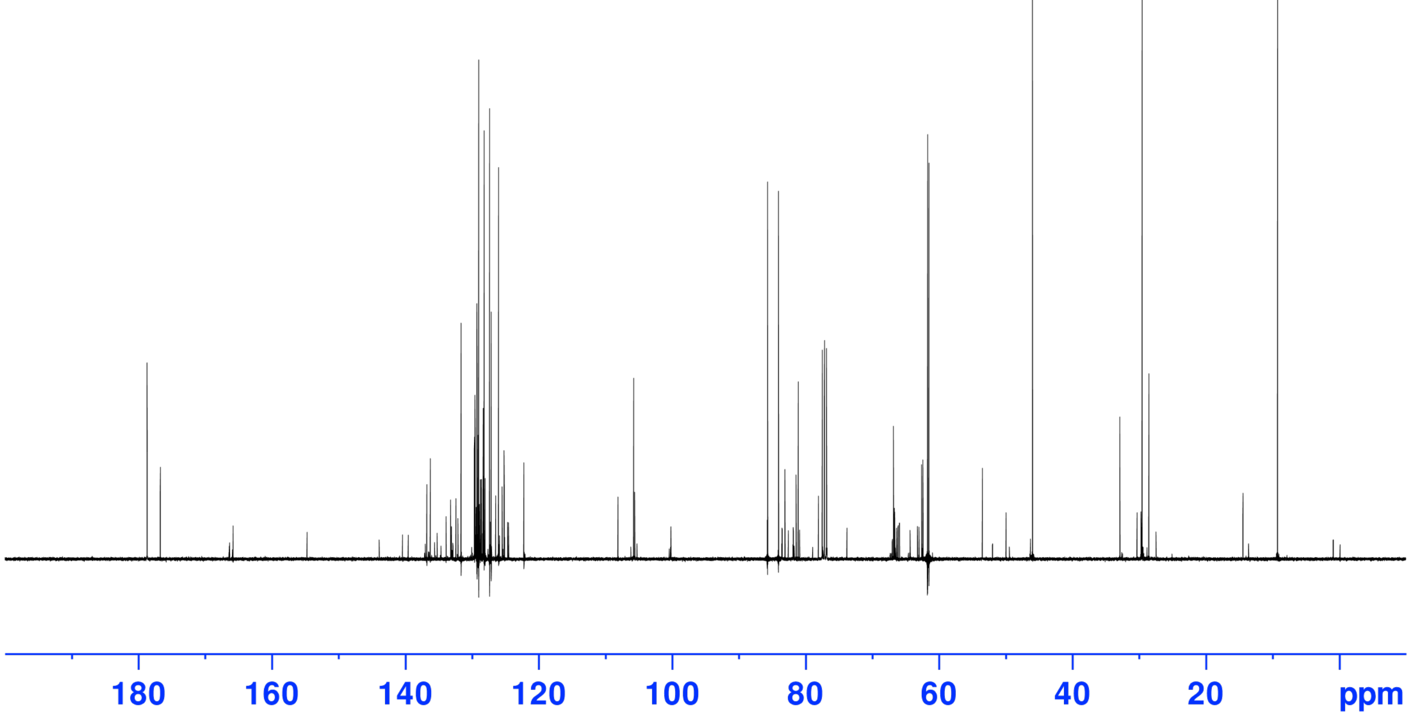

Current Data Parameters
NAME YC-4-138-CRUDE
EXPNO 2
PROCNO 1

F2 - Acquisition Parameters
Date_ 20240316
Time 6.21 h
INSTRUM spect
PROBHD Z150354_0001 (
PULPROG zgpg30
TD 65356
SOLVENT CDCl3
NS 128
DS 4
SWH 24038.461 Hz
FIDRES 0.735616 Hz
AQ 1.3594048 sec
RG 29.21
DW 20.800 usec
DE 25.00 usec
TE 298.0 K
D1 2.00000000 sec
D11 0.03000000 sec
TD0 1
SFO1 100.6655806 MHz
NUC1 13C
P1 10.00 usec
PLW1 18.70700073 W
SFO2 400.3016012 MHz
NUC2 1H
CPDPRG[2 waltz16
PCPD2 80.00 usec
PLW2 4.64209986 W
PLW12 0.10445000 W
PLW13 0.05245300 W

F2 - Processing parameters
SI 131072
SF 100.6555239 MHz
WDW EM
SSB 0
LB 0 Hz
GB 0
PC 1.40

The $^{13}C\{^{1}H\}$ NMR Spectrum of the Substitution Reaction of Acetal **6b** with 2-Fluoroethanol

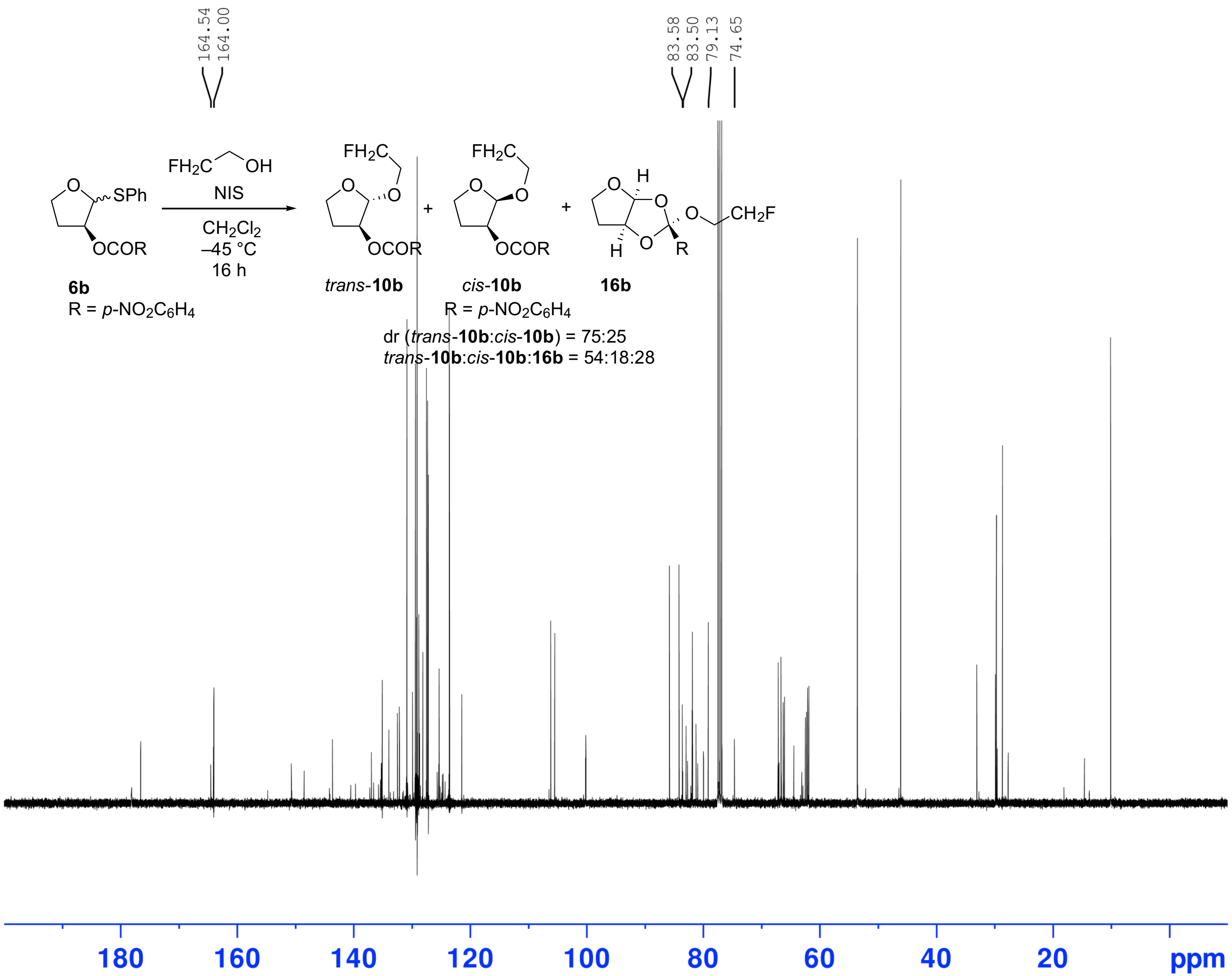

Current Data Parameters
NAME YC-4-137-CRUDE
EXPNO 2
PROCNO 1

F2 - Acquisition Parameters
Date_ 20240309
Time 3.35 h
INSTRUM spect
PROBHD Z150354_0001 (
PULPROG zgpg30
TD 65356
SOLVENT CDCl3
NS 128
DS 4
SWH 24038.461 Hz
FIDRES 0.735616 Hz
AQ 1.3594048 sec
RG 31.4
DW 20.800 usec
DE 25.00 usec
TE 298.0 K
D1 2.00000000 sec
D11 0.03000000 sec
TD0 1
SFO1 100.6655806 MHz
NUC1 13C
P1 10.00 usec
PLW1 18.70700073 W
SFO2 400.3016012 MHz
NUC2 1H
CPDPRG[2 waltz16
PCPD2 80.00 usec
PLW2 4.64209986 W
PLW12 0.10445000 W
PLW13 0.05245300 W

F2 - Processing parameters
SI 131072
SF 100.6555135 MHz
WDW EM
SSB 0
LB 0 Hz
GB 0
PC 1.40

The $^{13}C\{^1H\}$ NMR Spectrum of the Substitution Reaction of Acetal **6a** with Ethanol

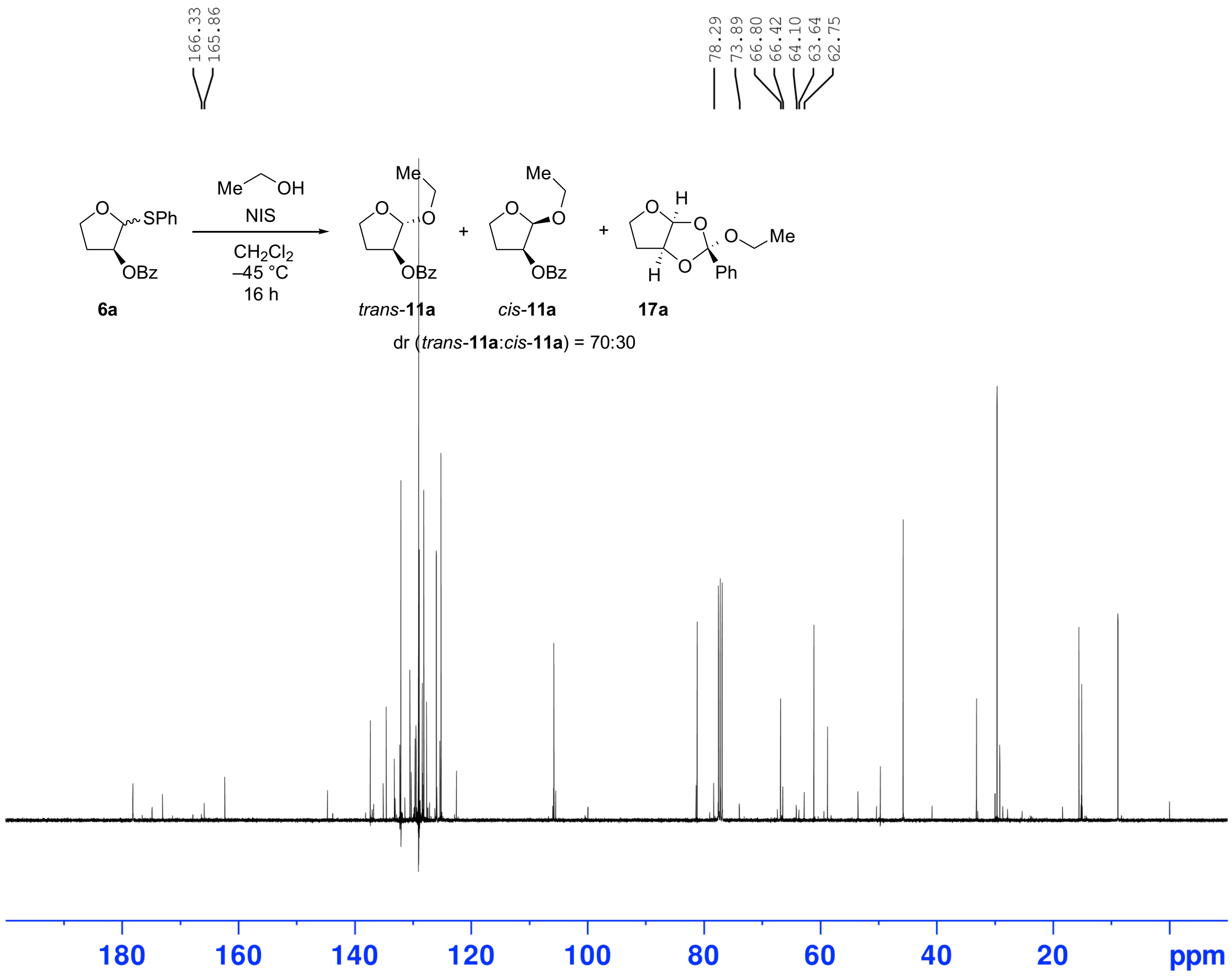


```
Current Data Parameters
NAME      YC-4-171-CRUDE
EXPNO                  2
PROCNO                 1

F2 - Acquisition Parameters
Date_           20240501
Time              1.38 h
INSTRUM            spect
PROBHD    Z150354_0001 (
PULPROG          zgpg30
TD                65356
SOLVENT           CDCl3
NS                  128
DS                    4
SWH           24038.461 Hz
FIDRES         0.735616 Hz
AQ            1.3594048 sec
RG                39.47
DW               20.800 usec
DE                25.00 usec
TE                298.0 K
D1           2.00000000 sec
D11          0.03000000 sec
TD0                   1
SFO1        100.6655806 MHz
NUC1                13C
P1                10.00 usec
PLW1        18.70700073 W
SFO2        400.3016012 MHz
NUC2                 1H
CPDPRG[2        waltz16
PCPD2             80.00 usec
PLW2         4.64209986 W
PLW12        0.10445000 W
PLW13        0.05245300 W

F2 - Processing parameters
SI               131072
SF          100.6555182 MHz
WDW                  EM
SSB                   0
LB                    0 Hz
GB                    0
PC                 1.40
```

The $^{13}C\{^1H\}$ NMR Spectrum of the Substitution Reaction of Acetal **6b** with 2-Fluoroethanol

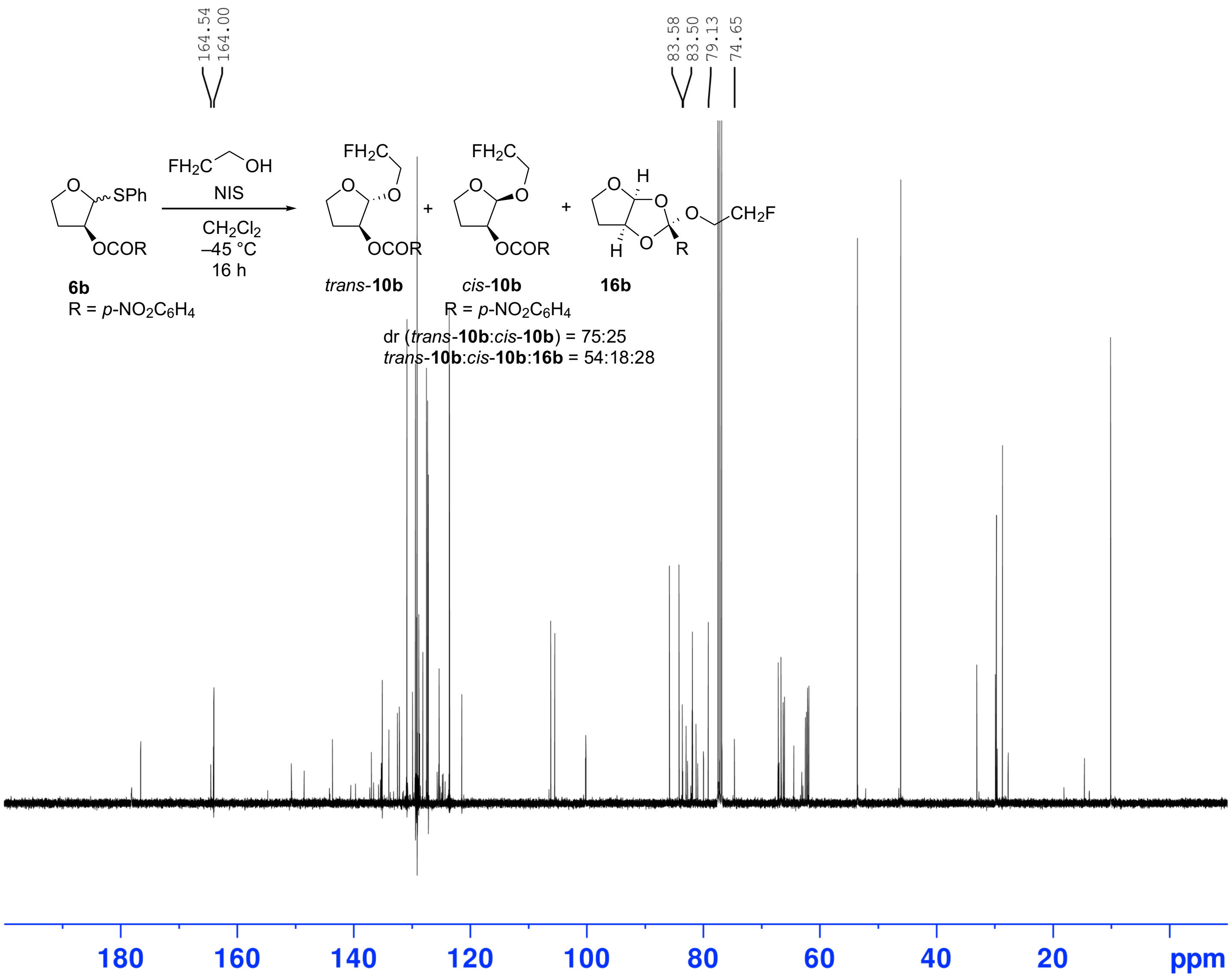


```
Current Data Parameters
NAME        YC-4-137-CRUDE
EXPNO                    2
PROCNO                   1

F2 - Acquisition Parameters
Date_             20240309
Time                  3.35 h
INSTRUM              spect
PROBHD     Z150354_0001 (
PULPROG            zgpg30
TD                   65356
SOLVENT              CDCl3
NS                     128
DS                       4
SWH              24038.461 Hz
FIDRES            0.735616 Hz
AQ               1.3594048 sec
RG                    31.4
DW                  20.800 usec
DE                   25.00 usec
TE                   298.0 K
D1              2.00000000 sec
D11             0.03000000 sec
TD0                      1
SFO1           100.6655806 MHz
NUC1                   13C
P1                   10.00 usec
PLW1           18.70700073 W
SFO2           400.3016012 MHz
NUC2                    1H
CPDPRG[2           waltz16
PCPD2                80.00 usec
PLW2            4.64209986 W
PLW12           0.10445000 W
PLW13           0.05245300 W

F2 - Processing parameters
SI                  131072
SF             100.6555135 MHz
WDW                     EM
SSB                      0
LB                       0 Hz
GB                       0
PC                    1.40
```

The $^{13}C\{^1H\}$ NMR Spectrum of the Substitution Reaction of Acetal **6a** with Ethanol

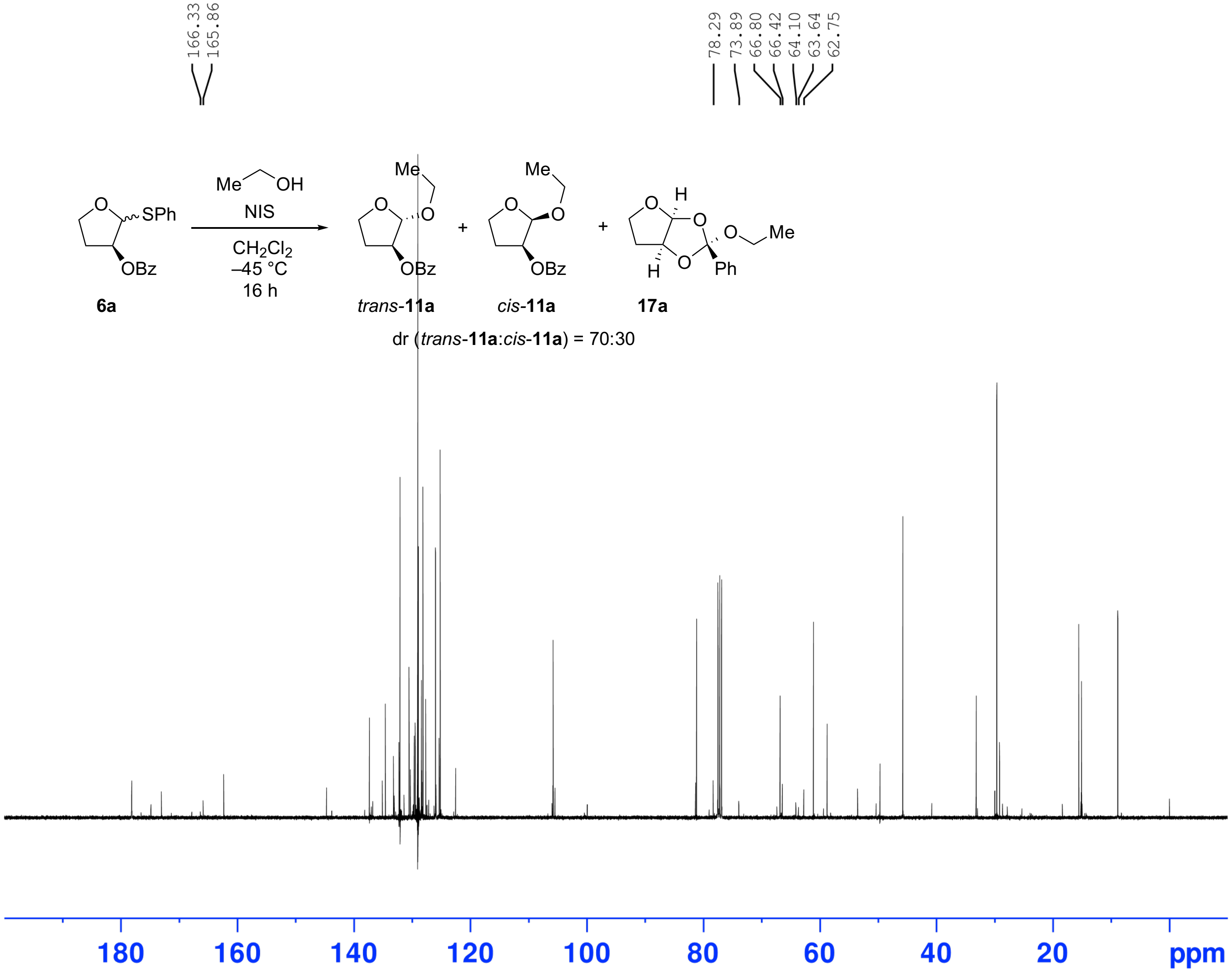

Current Data Parameters
NAME YC-4-171-CRUDE
EXPNO 2
PROCNO 1

F2 - Acquisition Parameters
Date_ 20240501
Time 1.38 h
INSTRUM spect
PROBHD Z150354_0001 (
PULPROG zgpg30
TD 65356
SOLVENT CDCl3
NS 128
DS 4
SWH 24038.461 Hz
FIDRES 0.735616 Hz
AQ 1.3594048 sec
RG 39.47
DW 20.800 usec
DE 25.00 usec
TE 298.0 K
D1 2.00000000 sec
D11 0.03000000 sec
TD0 1
SFO1 100.6655806 MHz
NUC1 13C
P1 10.00 usec
PLW1 18.70700073 W
SFO2 400.3016012 MHz
NUC2 1H
CPDPRG[2 waltz16
PCPD2 80.00 usec
PLW2 4.64209986 W
PLW12 0.10445000 W
PLW13 0.05245300 W

F2 - Processing parameters
SI 131072
SF 100.6555182 MHz
WDW EM
SSB 0
LB 0 Hz
GB 0
PC 1.40

The $^{13}C\{^1H\}$ NMR Spectrum of the Substitution Reaction of Acetal **6b** with Ethanol

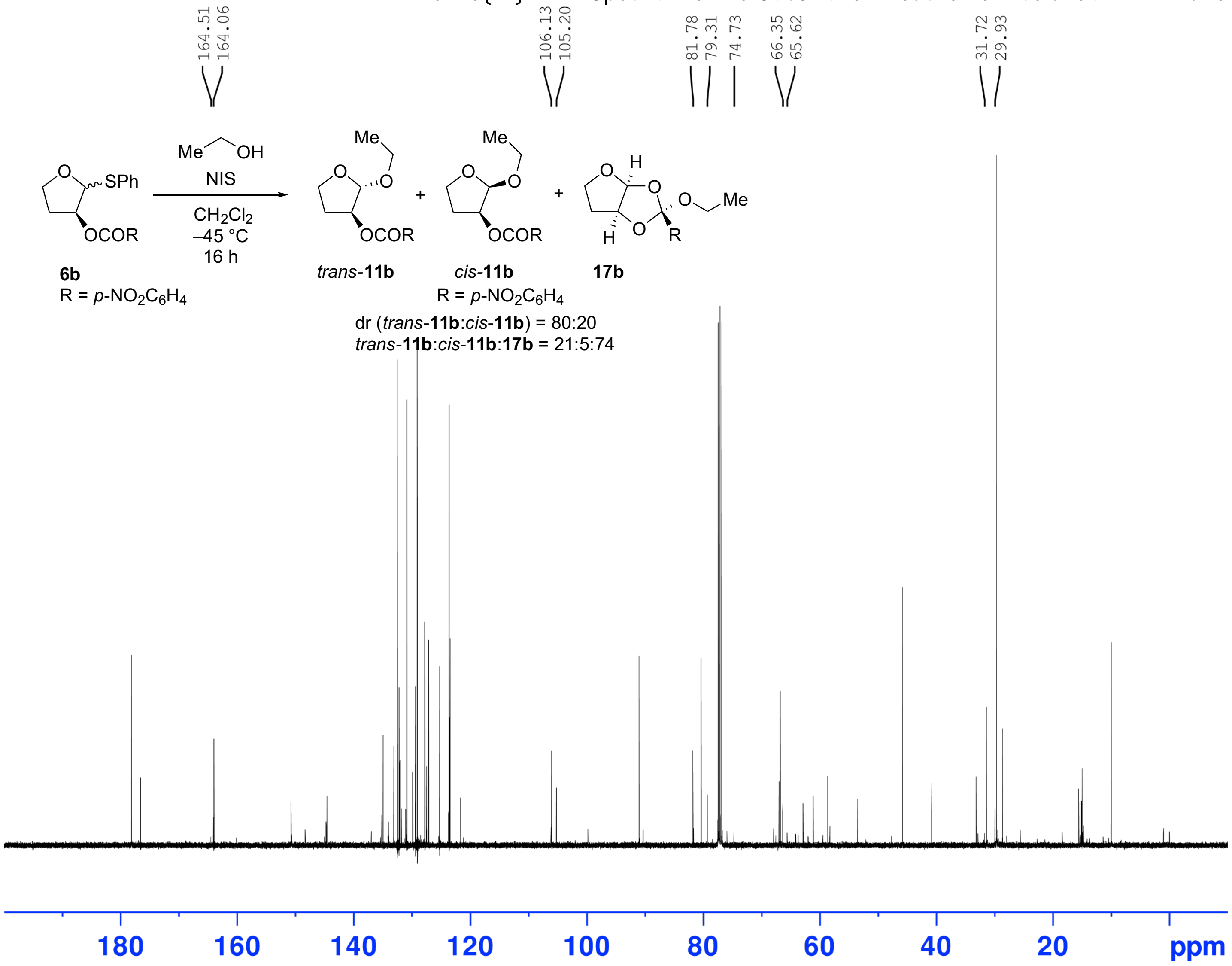


```
Current Data Parameters
NAME      YC-4-153-CRUDE
EXPNO                  2
PROCNO                 1

F2 - Acquisition Parameters
Date_           20240403
Time                3.44 h
INSTRUM            spect
PROBHD    Z150354_0001 (
PULPROG          zgpg30
TD                 65356
SOLVENT            CDCl3
NS                   128
DS                     4
SWH            24038.461 Hz
FIDRES          0.735616 Hz
AQ             1.3594048 sec
RG                 29.21
DW                20.800 usec
DE                 25.00 usec
TE                 298.0 K
D1            2.00000000 sec
D11           0.03000000 sec
TD0                    1
SFO1         100.6655806 MHz
NUC1                 13C
P1                 10.00 usec
PLW1         18.70700073 W
SFO2         400.3016012 MHz
NUC2                  1H
CPDPRG[2         waltz16
PCPD2              80.00 usec
PLW2          4.64209986 W
PLW12         0.10445000 W
PLW13         0.05245300 W

F2 - Processing parameters
SI                131072
SF           100.6555135 MHz
WDW                   EM
SSB                    0
LB                     0 Hz
GB                     0
PC                  1.40
```

The $^{13}C\{^1H\}$ NMR Spectrum of the Substitution Reaction of Acetal **6a** with Isopropanol

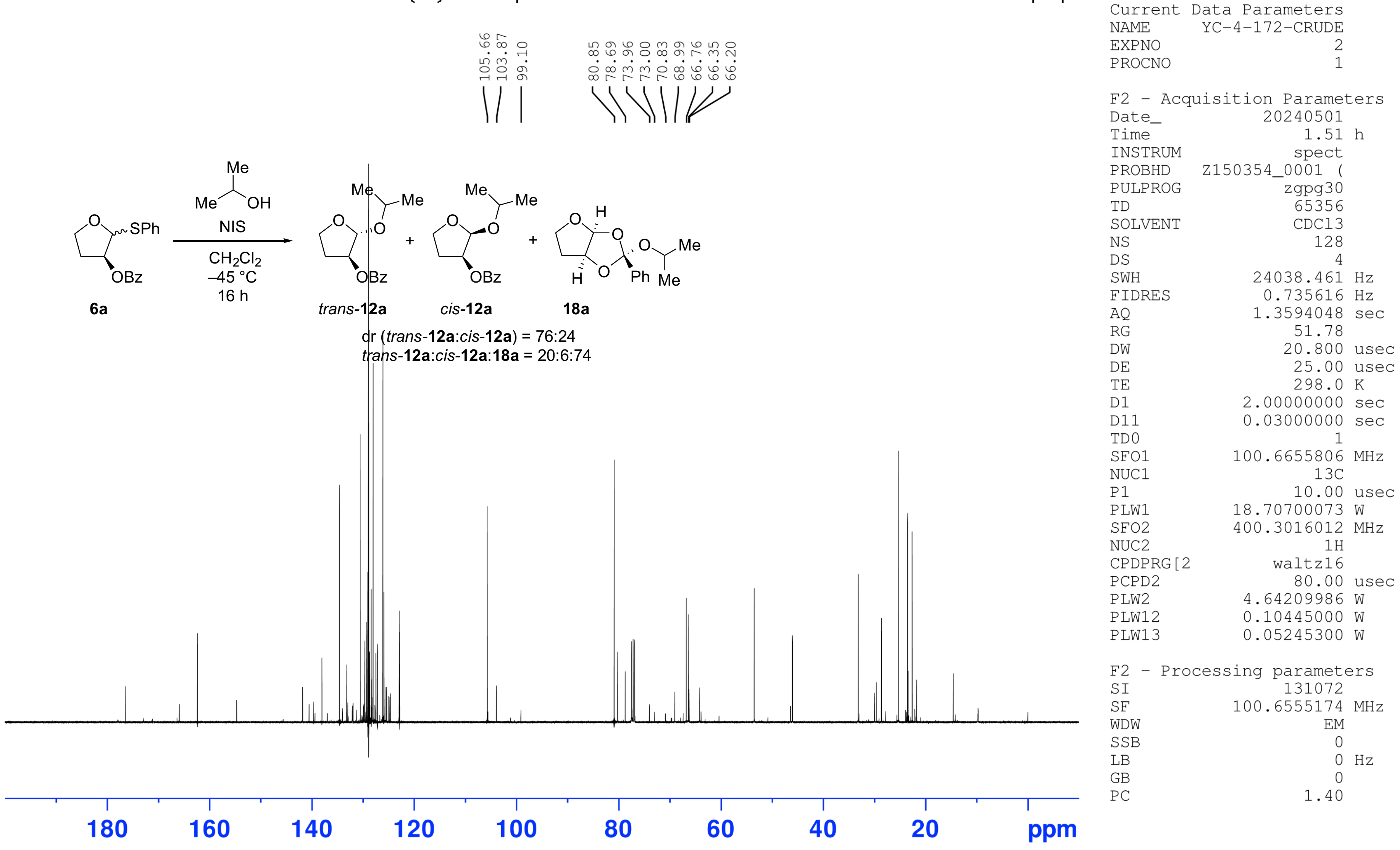

The $^{13}C\{^1H\}$ NMR Spectrum of the Substitution Reaction of Acetal **6b** with Isopropanol

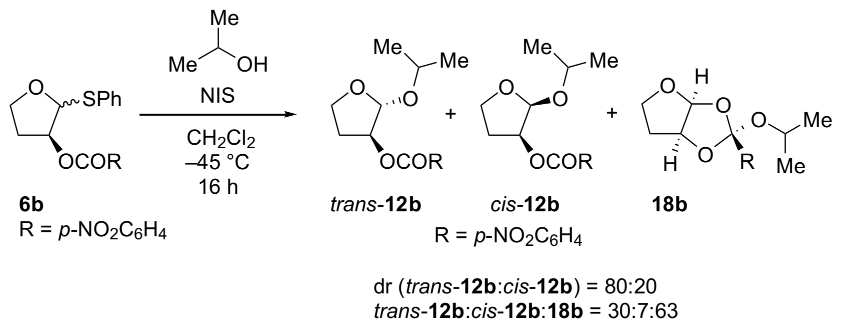

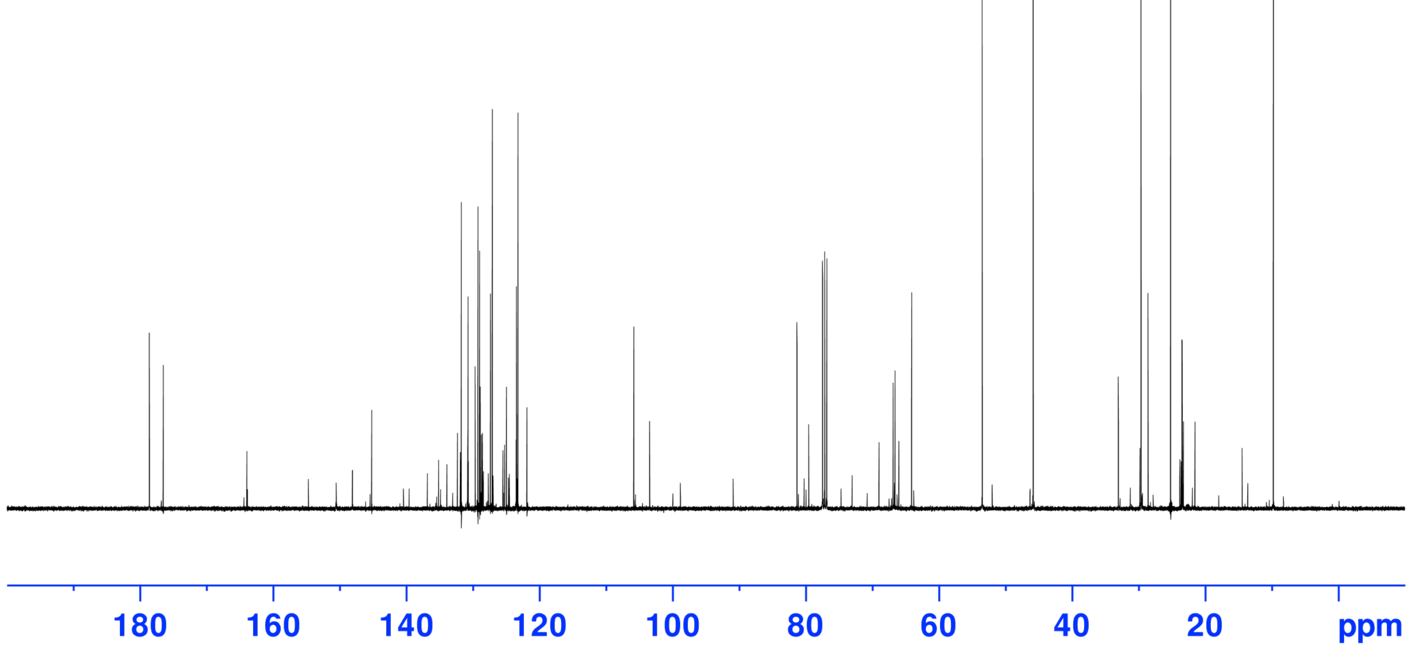

Current Data Parameters
NAME YC-4-154-CRUDE
EXPNO 2
PROCNO 1

F2 - Acquisition Parameters
Date_ 20240403
Time 4.03 h
INSTRUM spect
PROBHD Z150354_0001 (
PULPROG zgpg30
TD 65356
SOLVENT CDCl3
NS 128
DS 4
SWH 24038.461 Hz
FIDRES 0.735616 Hz
AQ 1.3594048 sec
RG 39.47
DW 20.800 usec
DE 25.00 usec
TE 298.0 K
D1 2.00000000 sec
D11 0.03000000 sec
TD0 1
SFO1 100.6655806 MHz
NUC1 13C
P1 10.00 usec
PLW1 18.70700073 W
SFO2 400.3016012 MHz
NUC2 1H
CPDPRG[2 waltz16
PCPD2 80.00 usec
PLW2 4.64209986 W
PLW12 0.10445000 W
PLW13 0.05245300 W

F2 - Processing parameters
SI 131072
SF 100.6555202 MHz
WDW EM
SSB 0
LB 0 Hz
GB 0
PC 1.40

The $^{13}C\{^{1}H\}$ NMR Spectrum of the Substitution Reaction of Acetal **6a** with 1,1,3,3-Hexafluoroisopropanol

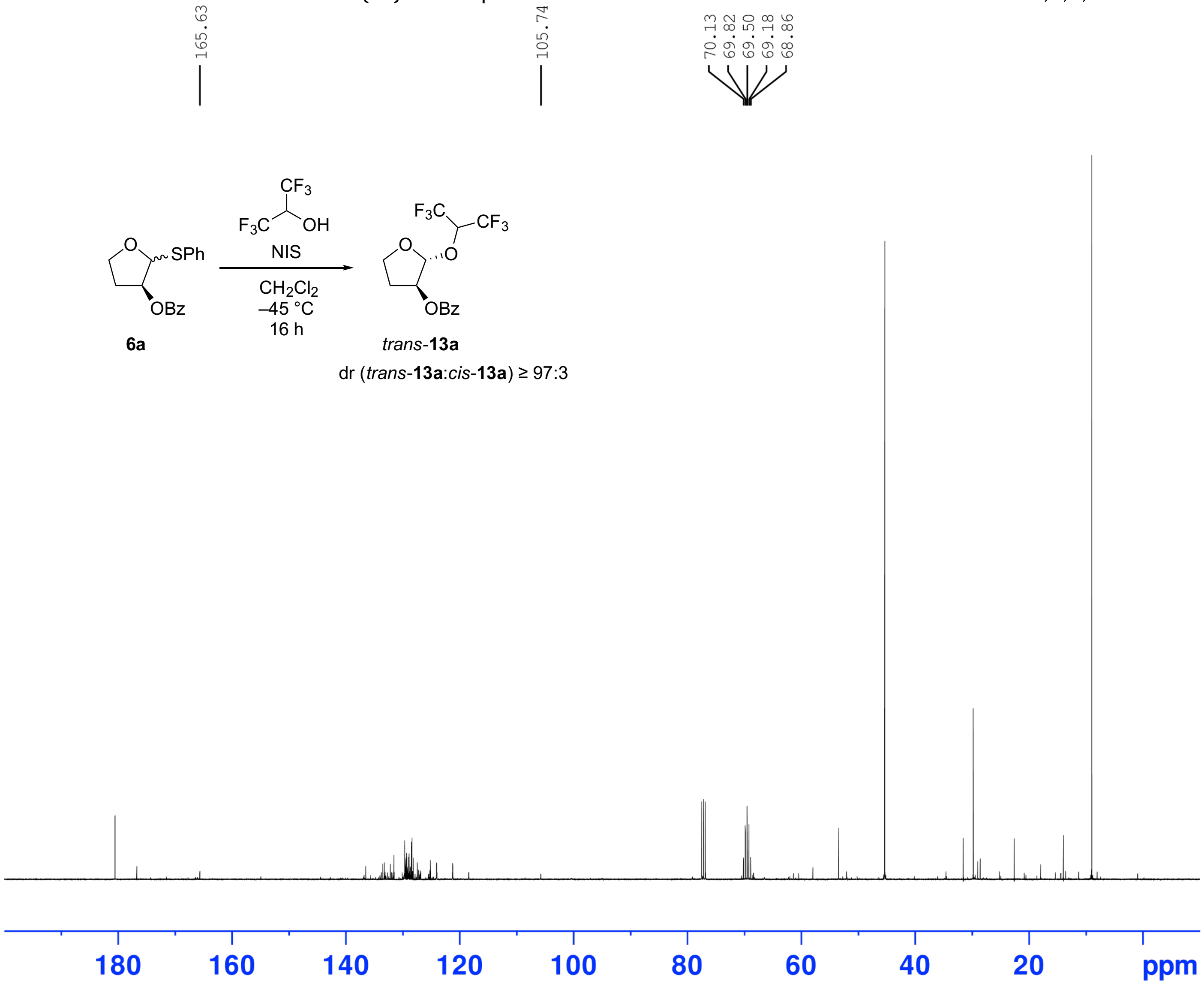

6a *trans*-**13a**

dr (*trans*-**13a**:*cis*-**13a**) ≥ 97:3

Current Data Parameters
NAME YC-4-149-CRUDE
EXPNO 3
PROCNO 1

F2 - Acquisition Parameters
Date_ 20240322
Time 6.52 h
INSTRUM spect
PROBHD Z150354_0001 (
PULPROG zgpg30
TD 65356
SOLVENT CDCl3
NS 128
DS 4
SWH 24038.461 Hz
FIDRES 0.735616 Hz
AQ 1.3594048 sec
RG 45.21
DW 20.800 usec
DE 25.00 usec
TE 298.0 K
D1 2.00000000 sec
D11 0.03000000 sec
TD0 1
SFO1 100.6655806 MHz
NUC1 13C
P1 10.00 usec
PLW1 18.70700073 W
SFO2 400.3016012 MHz
NUC2 1H
CPDPRG[2 waltz16
PCPD2 80.00 usec
PLW2 4.64209986 W
PLW12 0.10445000 W
PLW13 0.05245300 W

F2 - Processing parameters
SI 131072
SF 100.6555131 MHz
WDW EM
SSB 0
LB 0 Hz
GB 0
PC 1.40

The $^{13}C\{^1H\}$ NMR Spectrum of the Substitution Reaction of Acetal **6b** with 1,1,3,3-Hexafluoroisopropanol

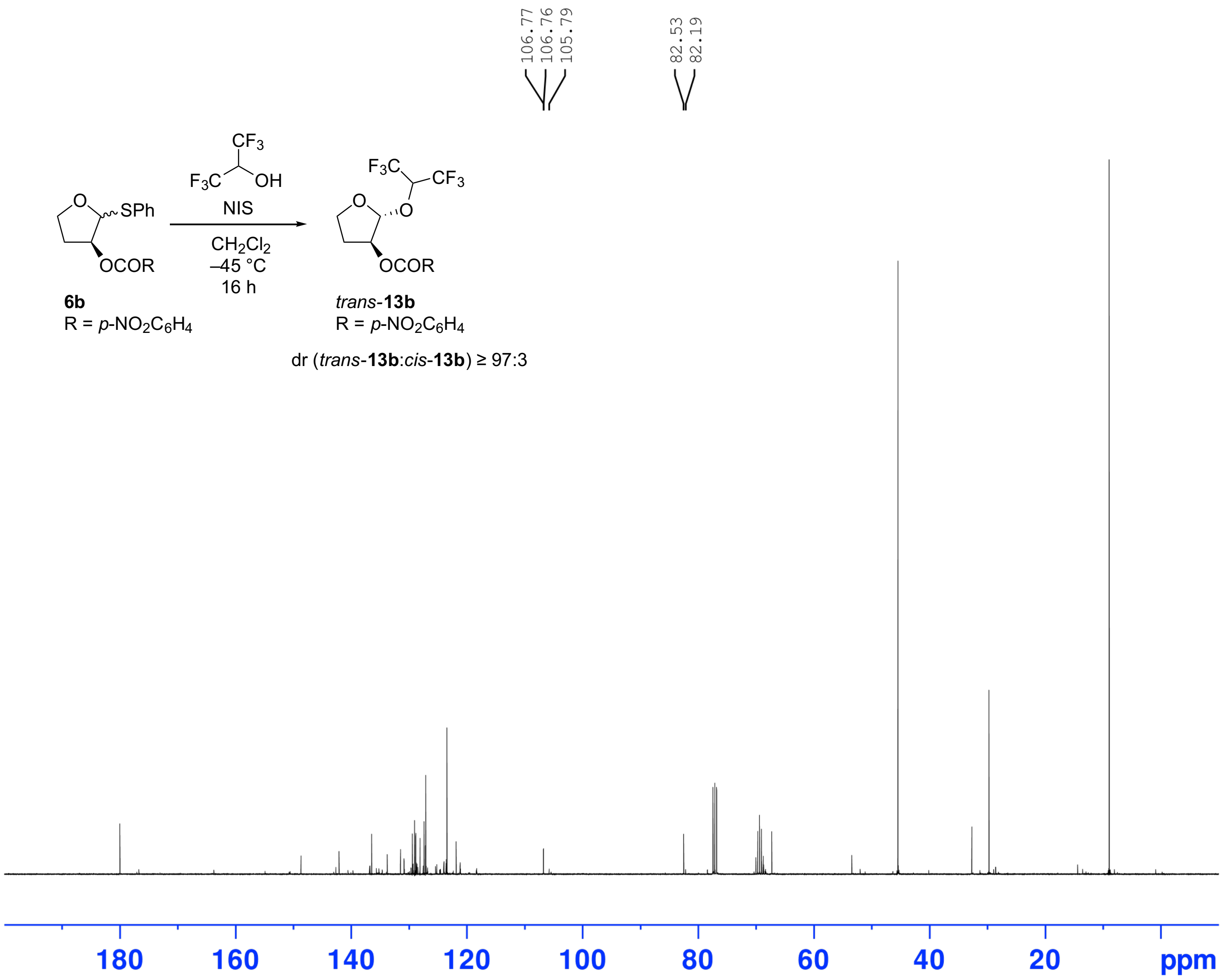

6b
R = *p*-$NO_2C_6H_4$

trans-**13b**
R = *p*-$NO_2C_6H_4$

dr (*trans*-**13b**:*cis*-**13b**) ≥ 97:3


```
Current Data Parameters
NAME       YC-4-148-CRUDE
EXPNO                   3
PROCNO                  1

F2 - Acquisition Parameters
Date_            20240322
Time                 6.40 h
INSTRUM             spect
PROBHD     Z150354_0001 (
PULPROG           zgpg30
TD                  65356
SOLVENT             CDCl3
NS                    128
DS                      4
SWH             24038.461 Hz
FIDRES           0.735616 Hz
AQ              1.3594048 sec
RG                   31.4
DW                 20.800 usec
DE                  25.00 usec
TE                  298.0 K
D1             2.00000000 sec
D11            0.03000000 sec
TD0                     1
SFO1          100.6655806 MHz
NUC1                  13C
P1                  10.00 usec
PLW1          18.70700073 W
SFO2          400.3016012 MHz
NUC2                   1H
CPDPRG[2          waltz16
PCPD2               80.00 usec
PLW2           4.64209986 W
PLW12          0.10445000 W
PLW13          0.05245300 W

F2 - Processing parameters
SI                 131072
SF            100.6555150 MHz
WDW                    EM
SSB                     0
LB                      0 Hz
GB                      0
PC                   1.40
```

The $^{13}C\{^1H\}$ NMR Spectrum of the Substitution Reaction of Acetal **6b** with 2,2,2-Trifluoroethanol

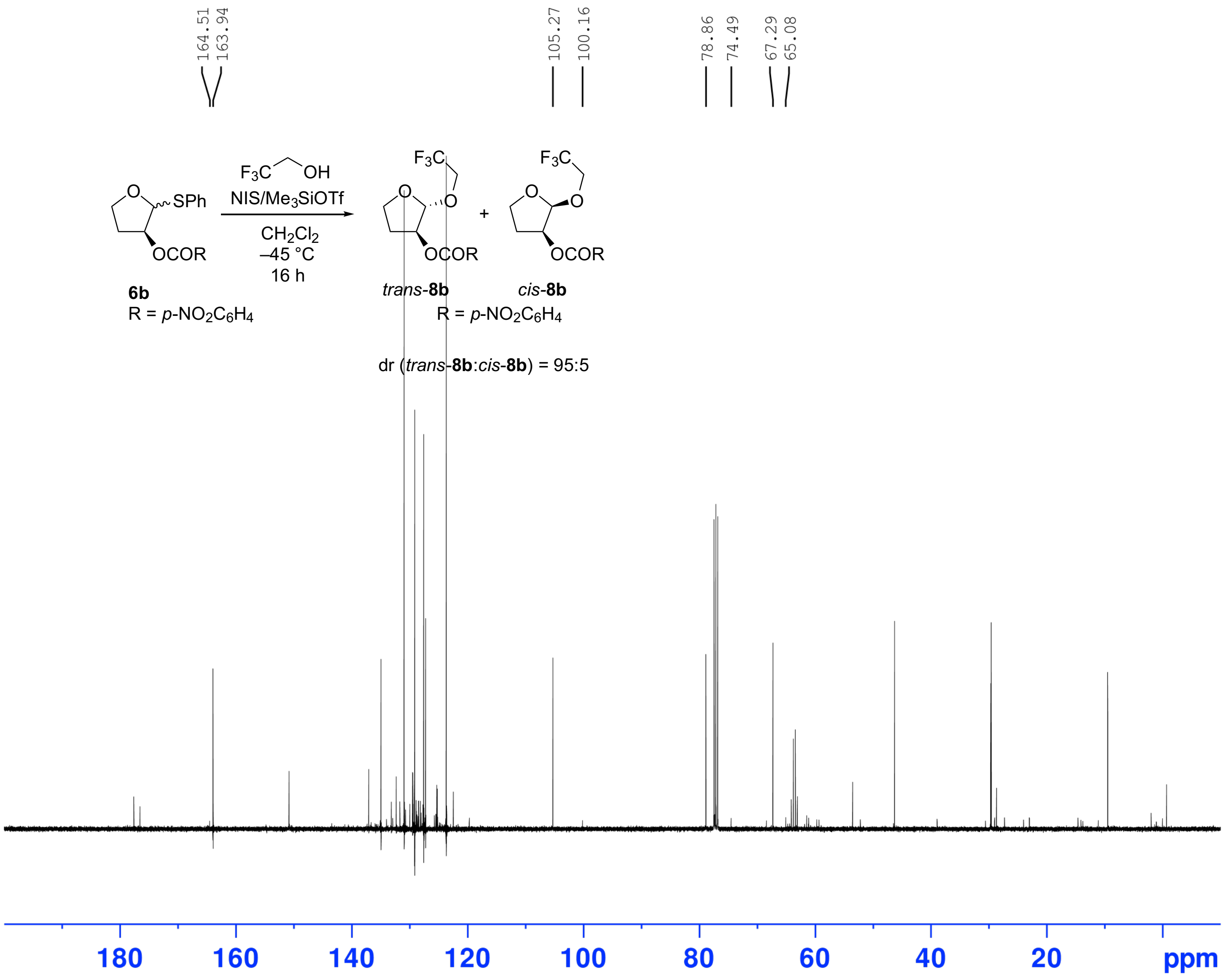


```
Current Data Parameters
NAME       YC-4-183-CRUDE
EXPNO                   2
PROCNO                  1

F2 - Acquisition Parameters
Date_            20240515
Time                 5.18 h
INSTRUM             spect
PROBHD     Z150354_0001 (
PULPROG            zgpg30
TD                  65356
SOLVENT             CDCl3
NS                    128
DS                      4
SWH             24038.461 Hz
FIDRES           0.735616 Hz
AQ              1.3594048 sec
RG                  25.77
DW                 20.800 usec
DE                  25.00 usec
TE                  298.0 K
D1             2.00000000 sec
D11            0.03000000 sec
TD0                     1
SFO1         100.6655806 MHz
NUC1                  13C
P1                  10.00 usec
PLW1          18.70700073 W
SFO2         400.3016012 MHz
NUC2                   1H
CPDPRG[2          waltz16
PCPD2               80.00 usec
PLW2           4.64209986 W
PLW12          0.10445000 W
PLW13          0.05245300 W

F2 - Processing parameters
SI                 131072
SF           100.6555083 MHz
WDW                    EM
SSB                     0
LB                      0 Hz
GB                      0
PC                   1.40
```

The $^{13}C\{^{1}H\}$ NMR Spectrum of the Substitution Reaction of Acetal **6b** with 2,2,2-Trifluoroethanol

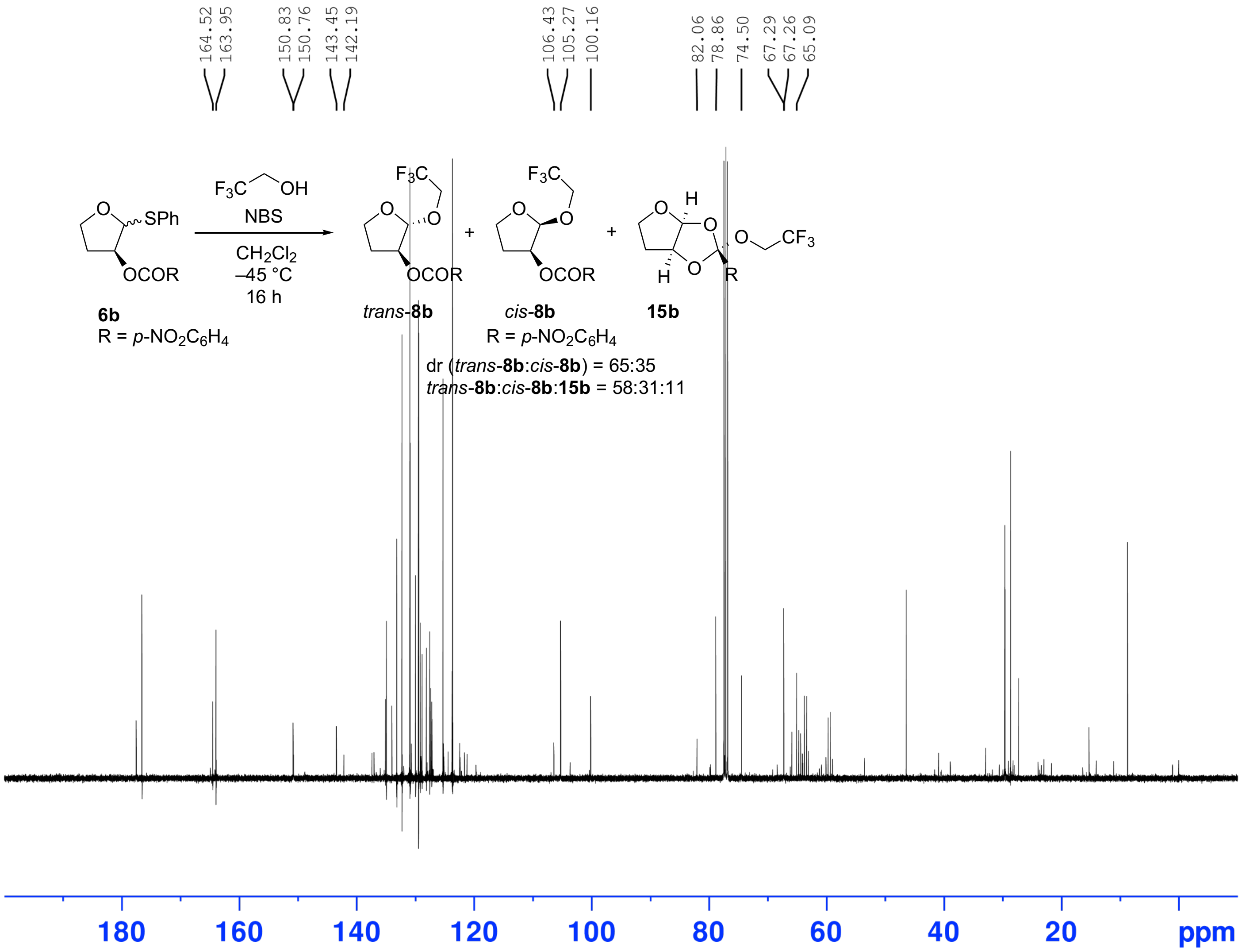


```
Current Data Parameters
NAME      YC-4-184-CRUDE
EXPNO                  2
PROCNO                 1

F2 - Acquisition Parameters
Date_           20240515
Time                5.38 h
INSTRUM            spect
PROBHD    Z150354_0001 (
PULPROG          zgpg30
TD                 65356
SOLVENT            CDCl3
NS                   128
DS                     4
SWH            24038.461 Hz
FIDRES          0.735616 Hz
AQ             1.3594048 sec
RG                 36.68
DW                20.800 usec
DE                 25.00 usec
TE                 298.0 K
D1            2.00000000 sec
D11           0.03000000 sec
TD0                    1
SFO1         100.6655806 MHz
NUC1                 13C
P1                 10.00 usec
PLW1         18.70700073 W
SFO2         400.3016012 MHz
NUC2                  1H
CPDPRG[2         waltz16
PCPD2              80.00 usec
PLW2          4.64209986 W
PLW12         0.10445000 W
PLW13         0.05245300 W

F2 - Processing parameters
SI                131072
SF           100.6555075 MHz
WDW                   EM
SSB                    0
LB                     0 Hz
GB                     0
PC                  1.40
```

The $^{13}C\{^1H\}$ NMR Spectrum of the Substitution Reaction of Acetal **6b** with 2,2,2-Trifluoroethanol

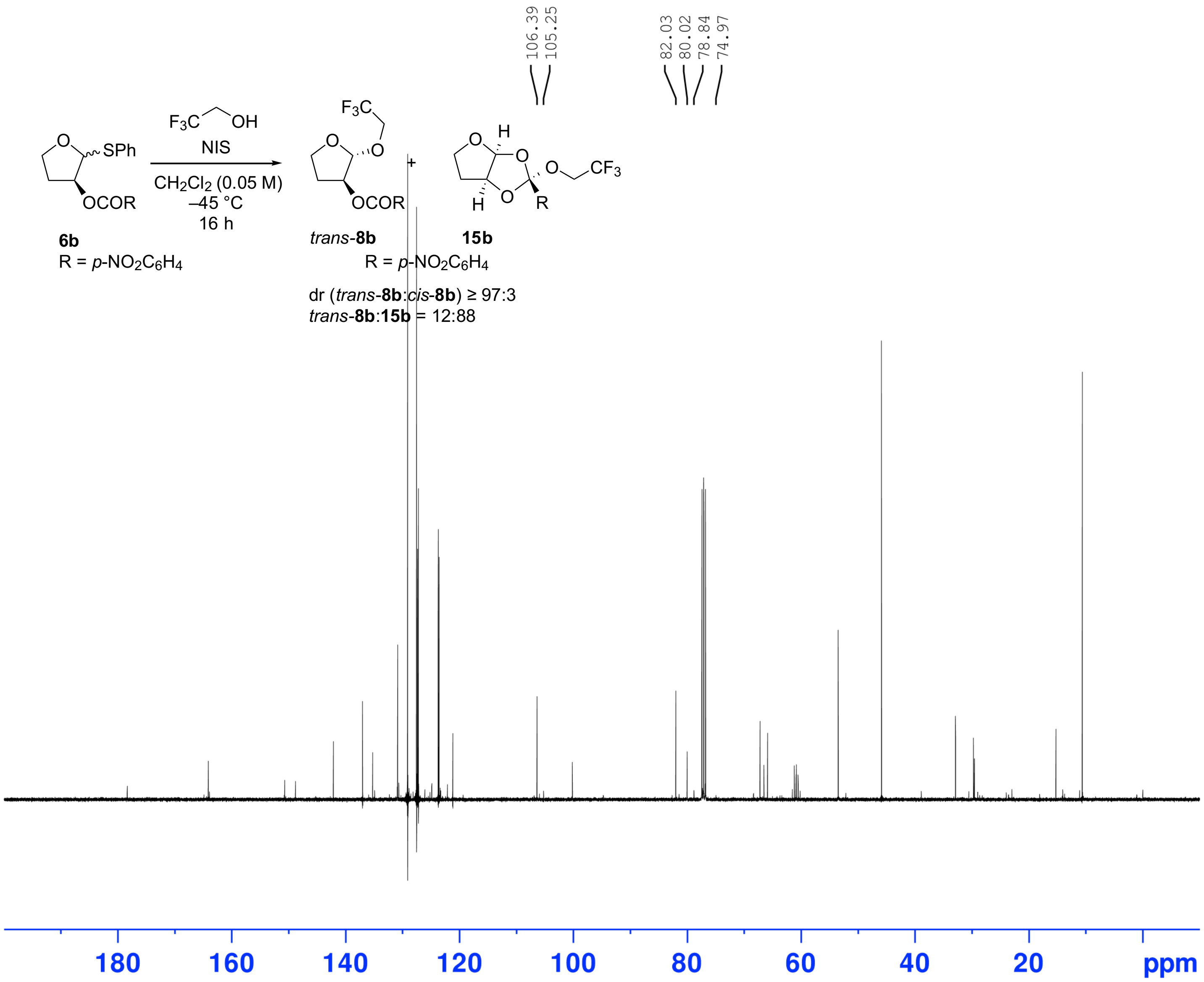

Current Data Parameters
NAME YC-4-185-CRUDE
EXPNO 2
PROCNO 1

F2 - Acquisition Parameters
Date_ 20240515
Time 5.50 h
INSTRUM spect
PROBHD Z150354_0001 (
PULPROG zgpg30
TD 65356
SOLVENT CDCl3
NS 128
DS 4
SWH 24038.461 Hz
FIDRES 0.735616 Hz
AQ 1.3594048 sec
RG 36.68
DW 20.800 usec
DE 25.00 usec
TE 298.0 K
D1 2.00000000 sec
D11 0.03000000 sec
TD0 1
SFO1 100.6655806 MHz
NUC1 13C
P1 10.00 usec
PLW1 18.70700073 W
SFO2 400.3016012 MHz
NUC2 1H
CPDPRG[2 waltz16
PCPD2 80.00 usec
PLW2 4.64209986 W
PLW12 0.10445000 W
PLW13 0.05245300 W

F2 - Processing parameters
SI 131072
SF 100.6555108 MHz
WDW EM
SSB 0
LB 0 Hz
GB 0
PC 1.40

The $^{13}C\{^{1}H\}$ NMR Spectrum of the Substitution Reaction of Acetal **6b** with 2,2,2-Trifluoroethanol

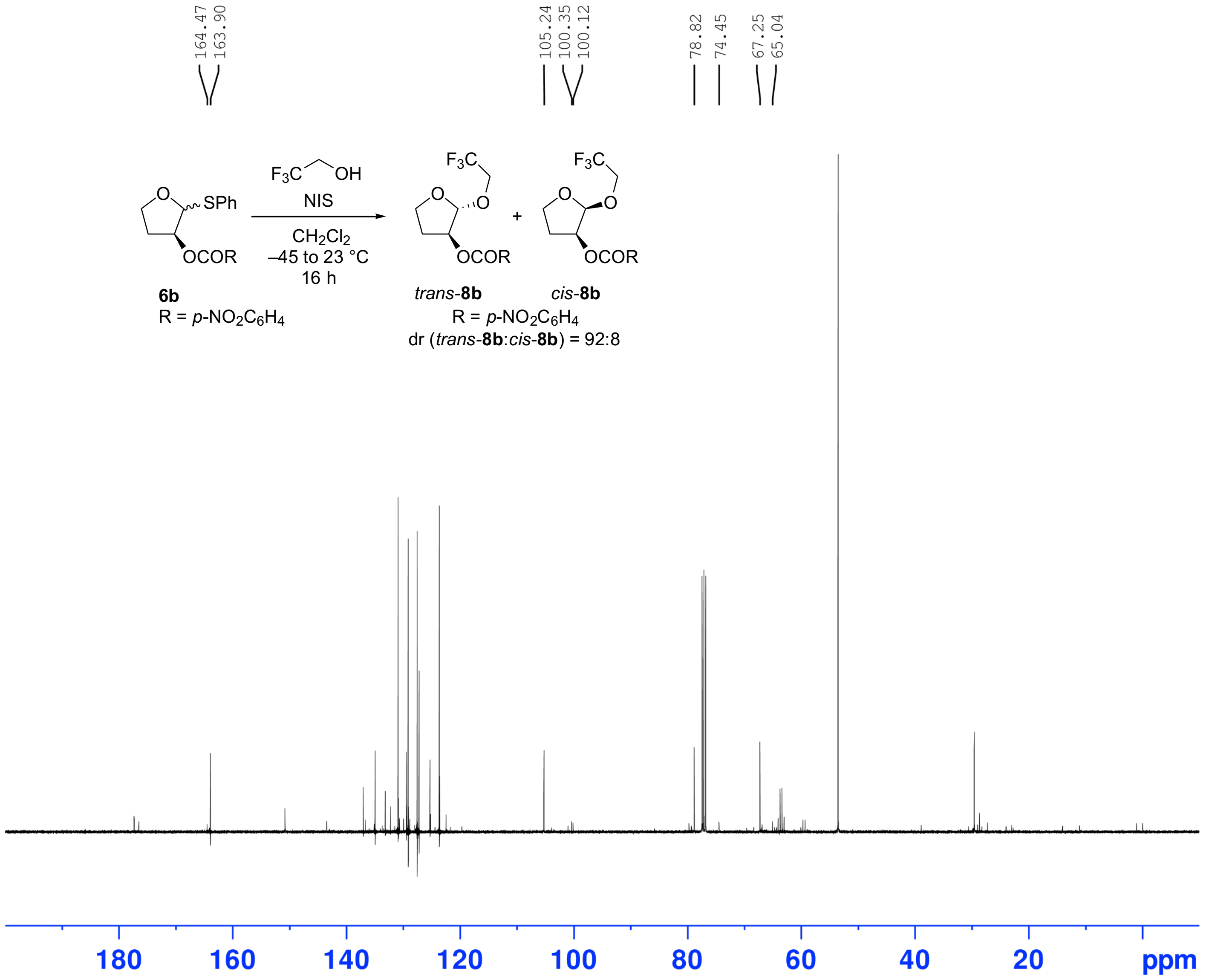

Current Data Parameters
NAME YC-4-182-CRUDE
EXPNO 2
PROCNO 1

F2 - Acquisition Parameters
Date_ 20240515
Time 3.31 h
INSTRUM spect
PROBHD Z150354_0001 (
PULPROG zgpg30
TD 65356
SOLVENT CDCl3
NS 128
DS 4
SWH 24038.461 Hz
FIDRES 0.735616 Hz
AQ 1.3594048 sec
RG 51.78
DW 20.800 usec
DE 25.00 usec
TE 298.0 K
D1 2.00000000 sec
D11 0.03000000 sec
TD0 1
SFO1 100.6655806 MHz
NUC1 13C
P1 10.00 usec
PLW1 18.70700073 W
SFO2 400.3016012 MHz
NUC2 1H
CPDPRG[2 waltz16
PCPD2 80.00 usec
PLW2 4.64209986 W
PLW12 0.10445000 W
PLW13 0.05245300 W

F2 - Processing parameters
SI 131072
SF 100.6555126 MHz
WDW EM
SSB 0
LB 0 Hz
GB 0
PC 1.40

The $^{13}C\{^{1}H\}$ NMR Spectrum of the Substitution Reaction of Acetal **6b** with 2,2,2-Trifluoroethanol

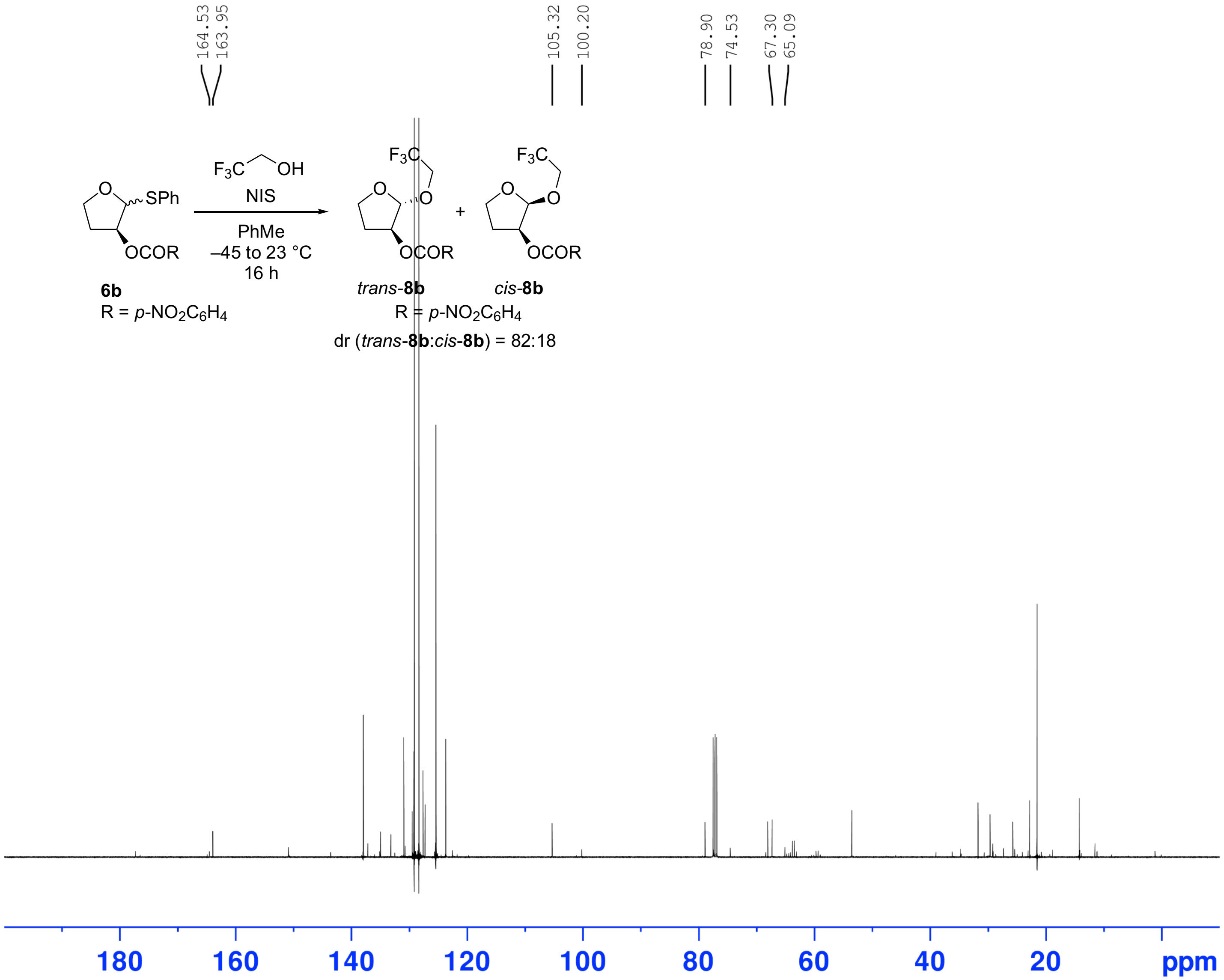


```
Current Data Parameters
NAME      YC-4-186-CRUDE
EXPNO                  2
PROCNO                 1

F2 - Acquisition Parameters
Date_           20240516
Time                0.17 h
INSTRUM            spect
PROBHD    Z150354_0001 (
PULPROG          zgpg30
TD                 65356
SOLVENT            CDCl3
NS                   128
DS                     4
SWH            24038.461 Hz
FIDRES          0.735616 Hz
AQ             1.3594048 sec
RG                 51.78
DW                20.800 usec
DE                 25.00 usec
TE                 298.0 K
D1            2.00000000 sec
D11           0.03000000 sec
TD0                    1
SFO1         100.6655806 MHz
NUC1                 13C
P1                 10.00 usec
PLW1         18.70700073 W
SFO2         400.3016012 MHz
NUC2                  1H
CPDPRG[2         waltz16
PCPD2              80.00 usec
PLW2          4.64209986 W
PLW12         0.10445000 W
PLW13         0.05245300 W

F2 - Processing parameters
SI                131072
SF           100.6555105 MHz
WDW                   EM
SSB                    0
LB                     0 Hz
GB                     0
PC                  1.40
```

The $^{13}C\{^{1}H\}$ NMR Spectrum of the Substitution Reaction of Acetal **6b** with 2,2,2-Trifluoroethanol

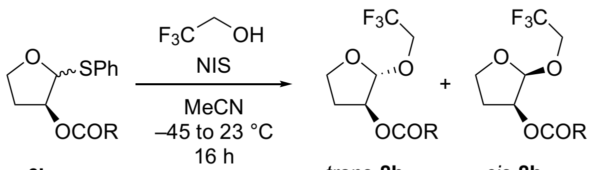

6b
R = *p*-$NO_2C_6H_4$

trans-**8b** *cis*-**8b**
R = *p*-$NO_2C_6H_4$

dr (*trans*-**8b**:*cis*-**8b**) = 57:43

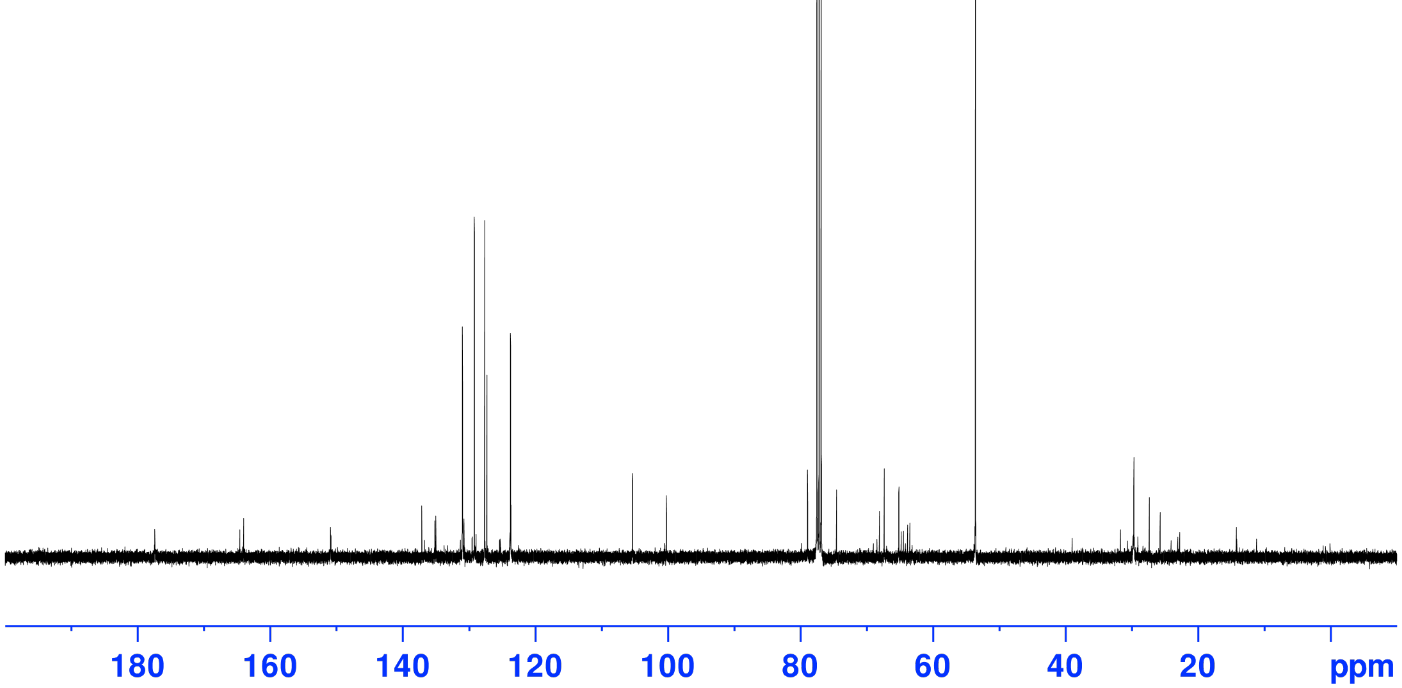

Current Data Parameters	
NAME	YC-4-187-CRUDE
EXPNO	2
PROCNO	1
F2 - Acquisition Parameters	
Date_	20240516
Time	2.55 h
INSTRUM	spect
PROBHD	Z150354_0001 (
PULPROG	zgpg30
TD	65356
SOLVENT	CDCl3
NS	128
DS	4
SWH	24038.461 Hz
FIDRES	0.735616 Hz
AQ	1.3594048 sec
RG	36.68
DW	20.800 usec
DE	25.00 usec
TE	298.0 K
D1	2.00000000 sec
D11	0.03000000 sec
TD0	1
SFO1	100.6655806 MHz
NUC1	13C
P1	10.00 usec
PLW1	18.70700073 W
SFO2	400.3016012 MHz
NUC2	1H
CPDPRG[2	waltz16
PCPD2	80.00 usec
PLW2	4.64209986 W
PLW12	0.10445000 W
PLW13	0.05245300 W
F2 - Processing parameters	
SI	131072
SF	100.6555023 MHz
WDW	EM
SSB	0
LB	0 Hz
GB	0
PC	1.40

The $^{13}C\{^{1}H\}$ NMR Spectrum of the Substitution Reaction of Acetal **6b** with 2,2,2-Trifluoroethanol

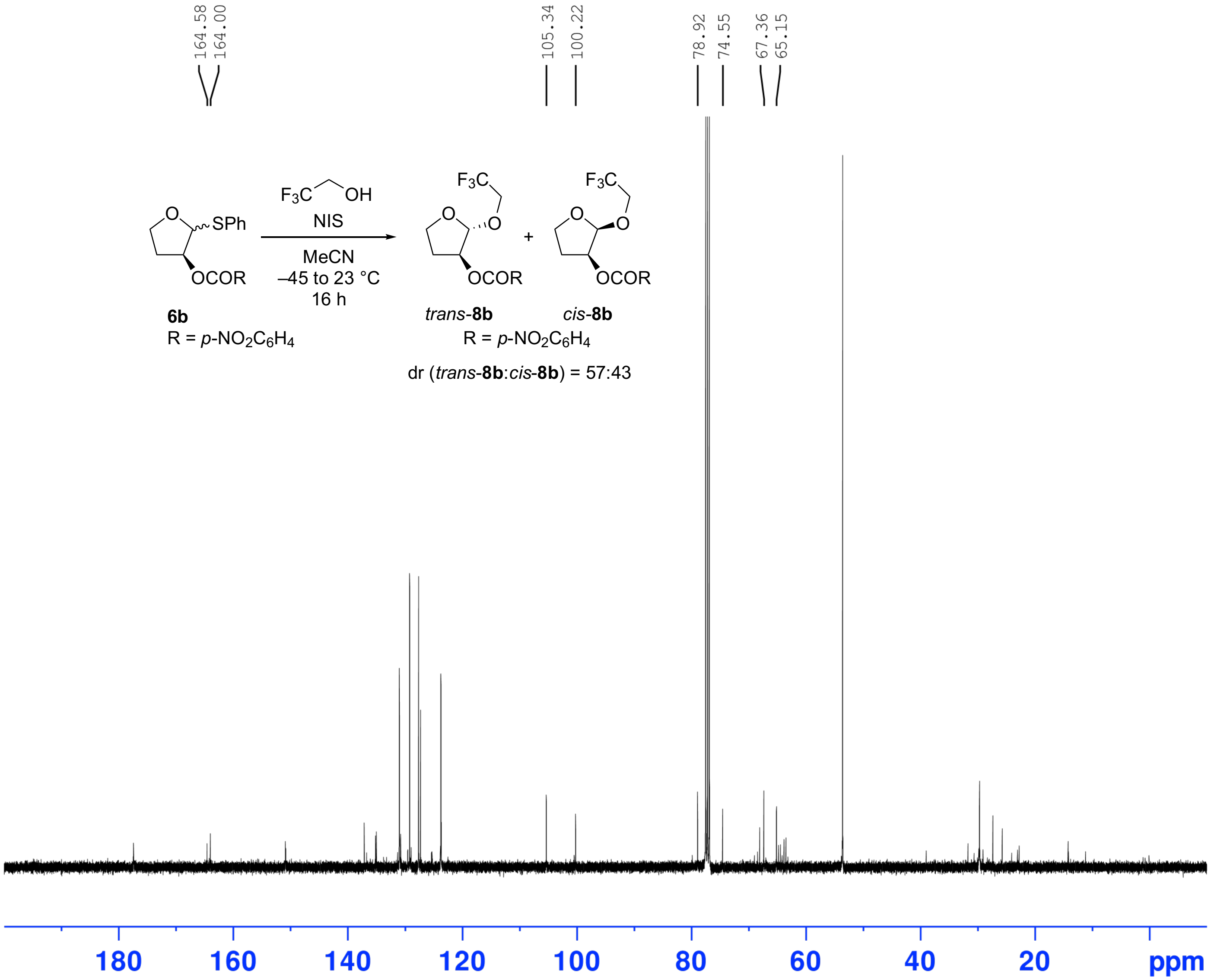

6b
R = *p*-$NO_2C_6H_4$

trans-**8b** *cis*-**8b**
R = *p*-$NO_2C_6H_4$

dr (*trans*-**8b**:*cis*-**8b**) = 57:43

Current Data Parameters

NAME	YC-4-187-CRUDE
EXPNO	2
PROCNO	1

F2 - Acquisition Parameters

Date_	20240516
Time	2.55 h
INSTRUM	spect
PROBHD	Z150354_0001 (
PULPROG	zgpg30
TD	65356
SOLVENT	CDCl3
NS	128
DS	4
SWH	24038.461 Hz
FIDRES	0.735616 Hz
AQ	1.3594048 sec
RG	36.68
DW	20.800 usec
DE	25.00 usec
TE	298.0 K
D1	2.00000000 sec
D11	0.03000000 sec
TD0	1
SFO1	100.6655806 MHz
NUC1	13C
P1	10.00 usec
PLW1	18.70700073 W
SFO2	400.3016012 MHz
NUC2	1H
CPDPRG[2	waltz16
PCPD2	80.00 usec
PLW2	4.64209986 W
PLW12	0.10445000 W
PLW13	0.05245300 W

F2 - Processing parameters

SI	131072
SF	100.6555023 MHz
WDW	EM
SSB	0
LB	0 Hz
GB	0
PC	1.40

The $^{13}C\{^1H\}$ NMR Spectrum of the Substitution Reaction of Acetal **21b** with Allylchlorodimethylsilane

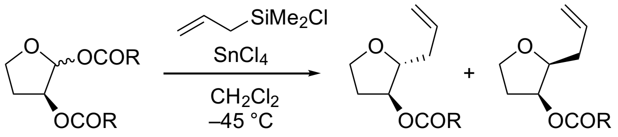

21b
R = *p*-$NO_2C_6H_4$

trans-**22b** *cis*-**22b**
R = *p*-$NO_2C_6H_4$
dr (*trans*-**22b**:*cis*-**22b**) = 61:39

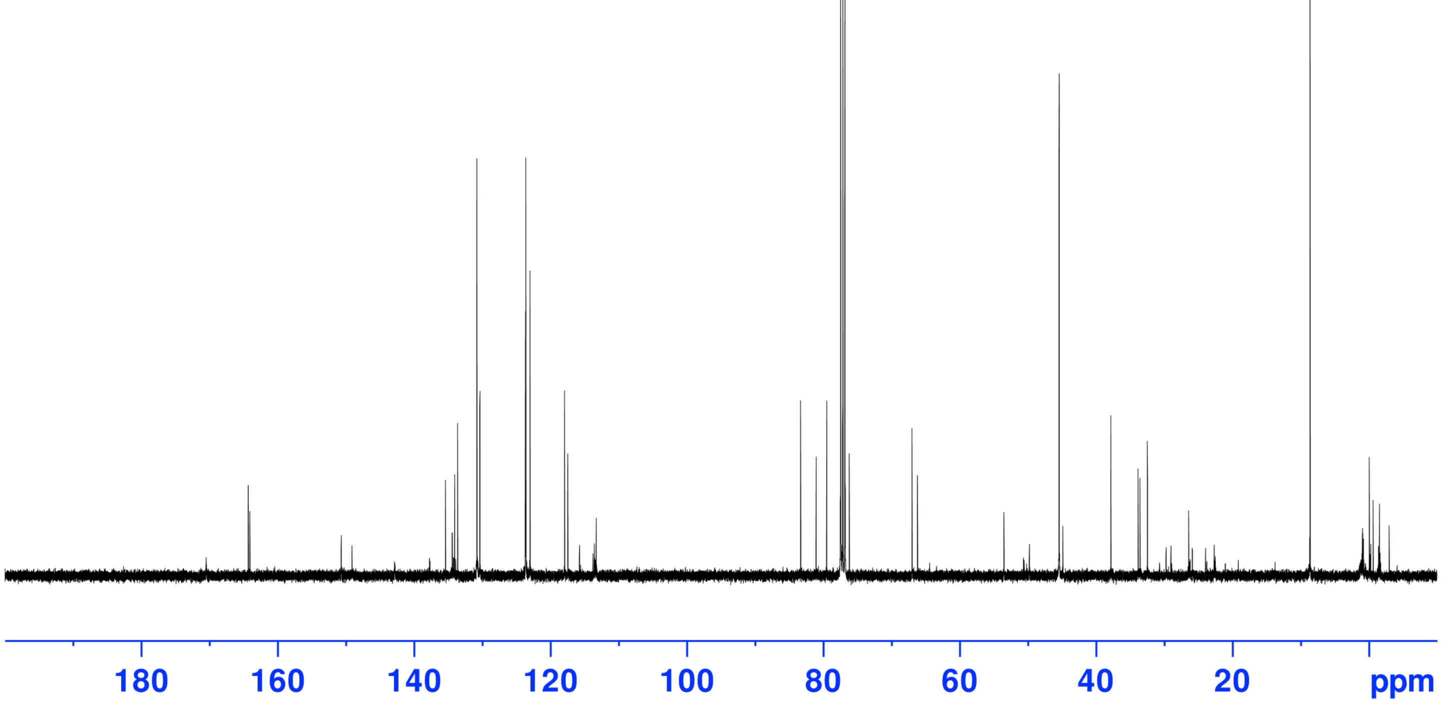

Current Data Parameters
NAME YC-4-127-CRUDE
EXPNO 2
PROCNO 1

F2 - Acquisition Parameters
Date_ 20240223
Time 2.10 h
INSTRUM spect
PROBHD Z150354_0001 (
PULPROG zgpg30
TD 65356
SOLVENT CDCl3
NS 128
DS 4
SWH 24038.461 Hz
FIDRES 0.735616 Hz
AQ 1.3594048 sec
RG 31.4
DW 20.800 usec
DE 25.00 usec
TE 298.0 K
D1 2.00000000 sec
D11 0.03000000 sec
TD0 1
SFO1 100.6655806 MHz
NUC1 13C
P1 10.00 usec
PLW1 18.70700073 W
SFO2 400.3016012 MHz
NUC2 1H
CPDPRG[2 waltz16
PCPD2 80.00 usec
PLW2 4.64209986 W
PLW12 0.10445000 W
PLW13 0.05245300 W

F2 - Processing parameters
SI 131072
SF 100.6555098 MHz
WDW EM
SSB 0
LB 0 Hz
GB 0
PC 1.40

The $^{13}C\{^{1}H\}$ NMR Spectrum of the Substitution Reaction of Acetal **21a** with Allylchlorodimethylsilane

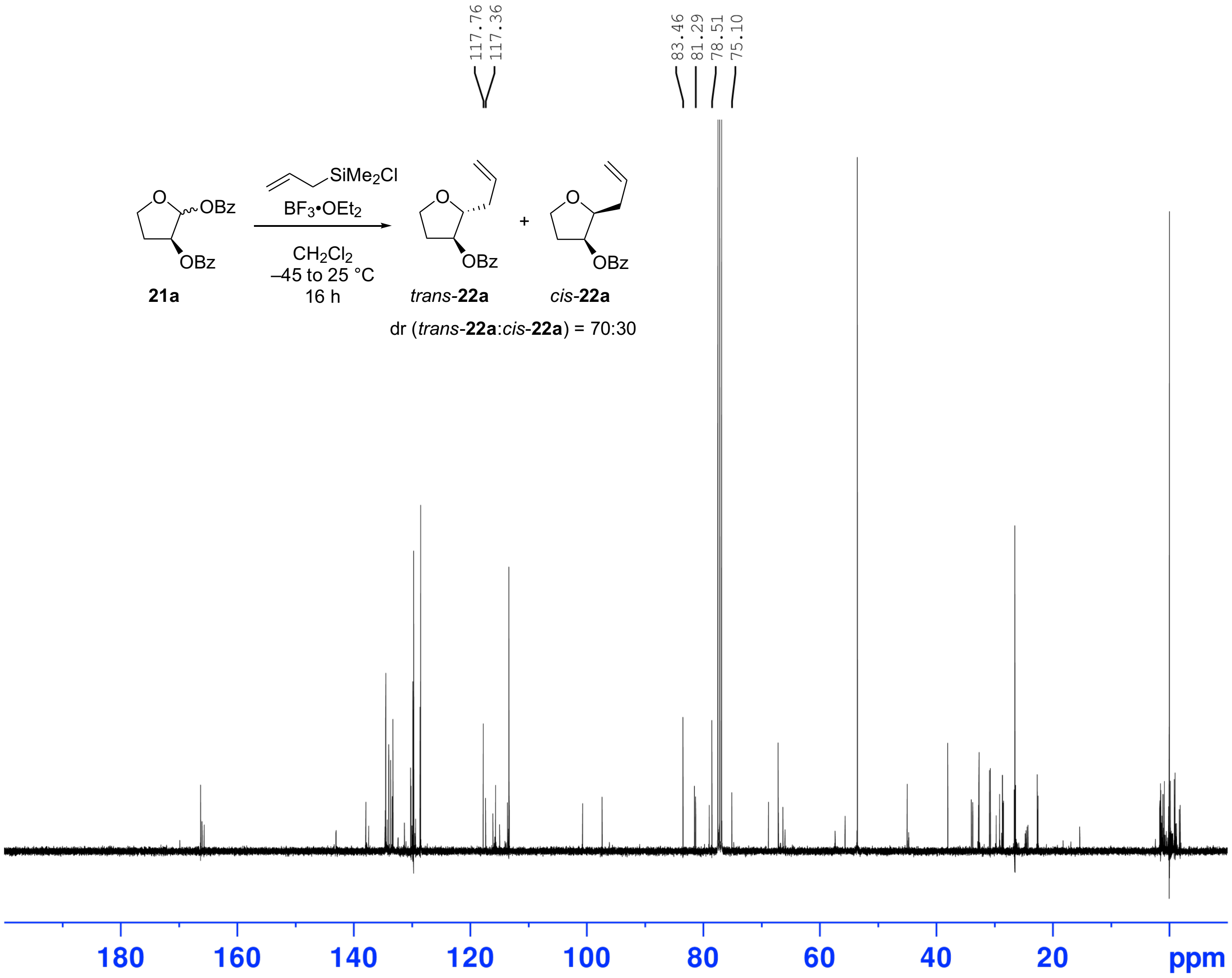

Current Data Parameters	
NAME	YC-4-140-CRUDE
EXPNO	2
PROCNO	1
F2 - Acquisition Parameters	
Date_	20240306
Time	3.06 h
INSTRUM	spect
PROBHD	Z150354_0001 (
PULPROG	zgpg30
TD	65356
SOLVENT	CDCl3
NS	128
DS	4
SWH	24038.461 Hz
FIDRES	0.735616 Hz
AQ	1.3594048 sec
RG	25.77
DW	20.800 usec
DE	25.00 usec
TE	298.0 K
D1	2.00000000 sec
D11	0.03000000 sec
TD0	1
SFO1	100.6655806 MHz
NUC1	13C
P1	10.00 usec
PLW1	18.70700073 W
SFO2	400.3016012 MHz
NUC2	1H
CPDPRG[2	waltz16
PCPD2	80.00 usec
PLW2	4.64209986 W
PLW12	0.10445000 W
PLW13	0.05245300 W
F2 - Processing parameters	
SI	131072
SF	100.6555044 MHz
WDW	EM
SSB	0
LB	0 Hz
GB	0
PC	1.40

The $^{13}C\{^{1}H\}$ NMR Spectrum of the Substitution Reaction of Acetal **21b** with Allylchlorodimethylsilane

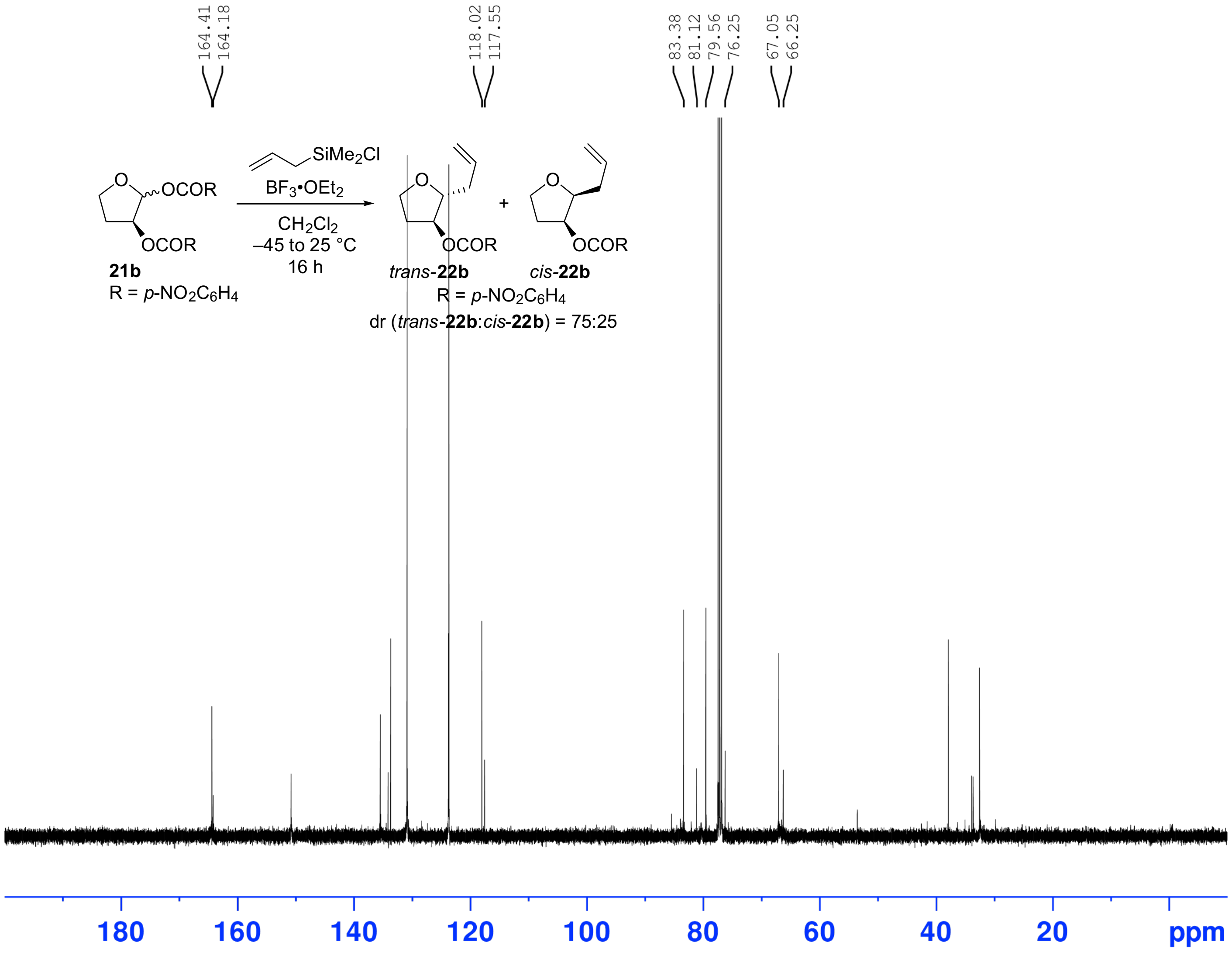

Current Data Parameters

NAME	YC-4-141-CRUDE
EXPNO	2
PROCNO	1

F2 - Acquisition Parameters

Date_	20240309
Time	3.47 h
INSTRUM	spect
PROBHD	Z150354_0001 (
PULPROG	zgpg30
TD	65356
SOLVENT	CDCl3
NS	128
DS	4
SWH	24038.461 Hz
FIDRES	0.735616 Hz
AQ	1.3594048 sec
RG	45.21
DW	20.800 usec
DE	25.00 usec
TE	298.0 K
D1	2.00000000 sec
D11	0.03000000 sec
TD0	1
SFO1	100.6655806 MHz
NUC1	13C
P1	10.00 usec
PLW1	18.70700073 W
SFO2	400.3016012 MHz
NUC2	1H
CPDPRG[2	waltz16
PCPD2	80.00 usec
PLW2	4.64209986 W
PLW12	0.10445000 W
PLW13	0.05245300 W

F2 - Processing parameters

SI	131072
SF	100.6555049 MHz
WDW	EM
SSB	0
LB	0 Hz
GB	0
PC	1.40

The $^{13}C\{^{1}H\}$ NMR Spectrum of the Substitution Reaction of Acetal **21a** with Allyltrimethylsilane

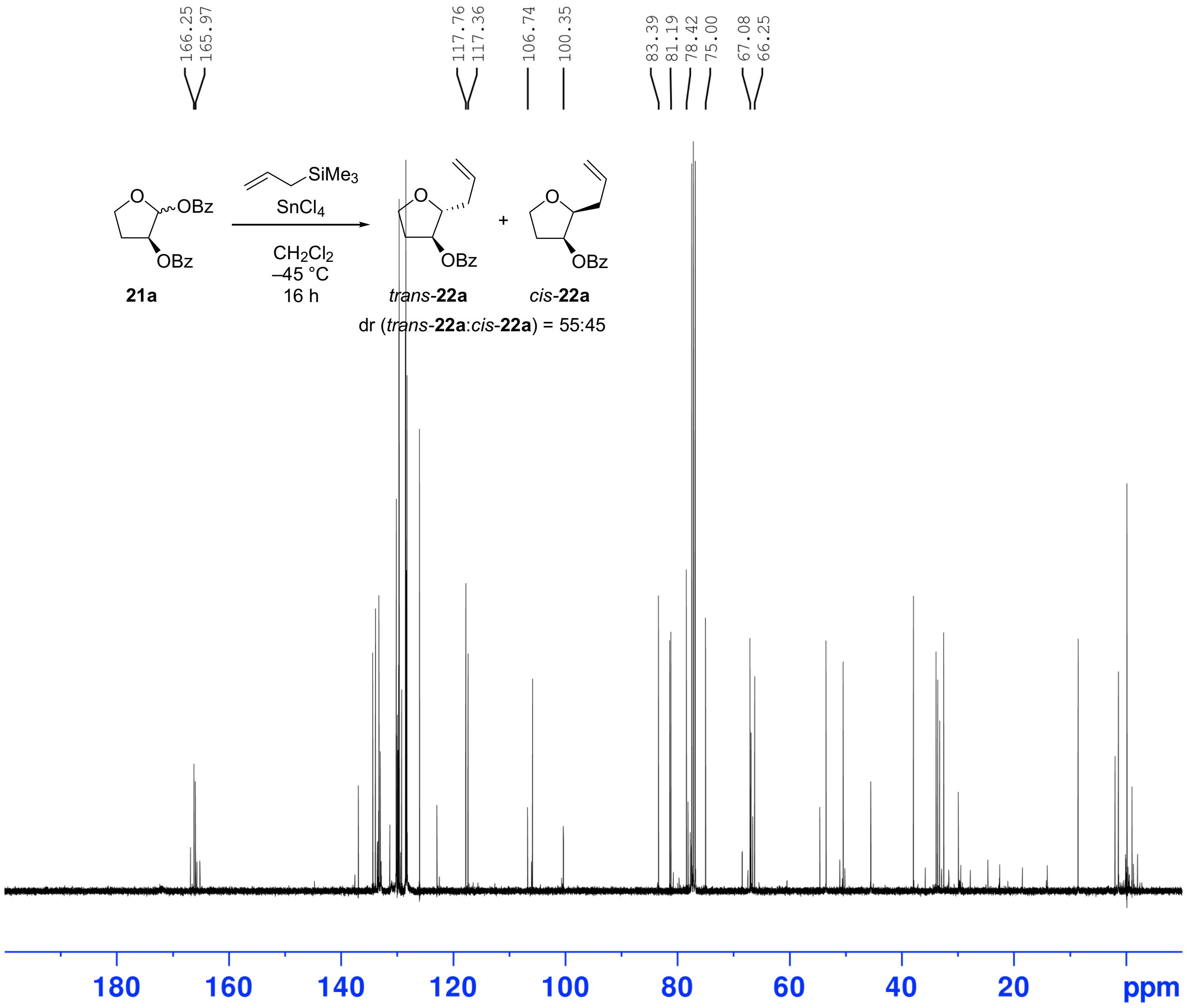

Current Data Parameters
NAME YC-3-126-CRUDE
EXPNO 2
PROCNO 1

F2 - Acquisition Parameters
Date_ 20230121
Time 2.14 h
INSTRUM spect
PROBHD Z150354_0001 (
PULPROG zgpg30
TD 65356
SOLVENT CDCl3
NS 128
DS 4
SWH 24038.461 Hz
FIDRES 0.735616 Hz
AQ 1.3594048 sec
RG 25.77
DW 20.800 usec
DE 25.00 usec
TE 289.9 K
D1 2.00000000 sec
D11 0.03000000 sec
TD0 1
SFO1 100.6655806 MHz
NUC1 13C
P1 10.00 usec
PLW1 17.99699974 W
SFO2 400.3016012 MHz
NUC2 1H
CPDPRG[2 waltz16
PCPD2 80.00 usec
PLW2 4.64209986 W
PLW12 0.10445000 W
PLW13 0.05245300 W

F2 - Processing parameters
SI 131072
SF 100.6555140 MHz
WDW EM
SSB 0
LB 0 Hz
GB 0
PC 1.40

The $^{13}C\{^{1}H\}$ NMR Spectrum of the Substitution Reaction of Acetal **21b** with Allyltrimethylsilane

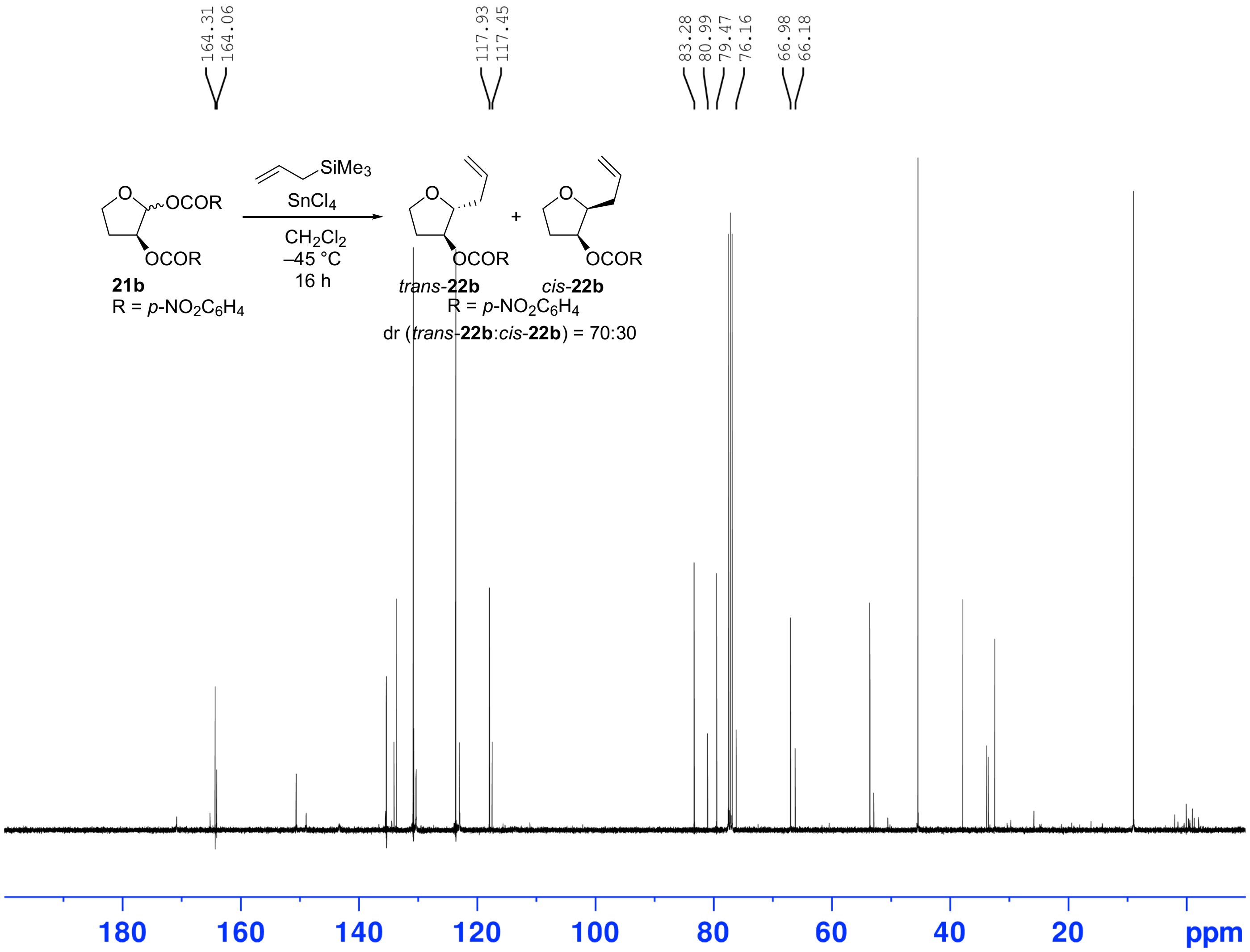


```
Current Data Parameters
NAME      YC-3-127-CRUDE
EXPNO                  2
PROCNO                 1

F2 - Acquisition Parameters
Date_           20230121
Time                2.30 h
INSTRUM            spect
PROBHD    Z150354_0001 (
PULPROG          zgpg30
TD                 65356
SOLVENT            CDCl3
NS                   128
DS                     4
SWH            24038.461 Hz
FIDRES          0.735616 Hz
AQ             1.3594048 sec
RG                 36.68
DW                20.800 usec
DE                 25.00 usec
TE                 290.0 K
D1            2.00000000 sec
D11           0.03000000 sec
TD0                    1
SFO1         100.6655806 MHz
NUC1                 13C
P1                 10.00 usec
PLW1         17.99699974 W
SFO2         400.3016012 MHz
NUC2                  1H
CPDPRG[2         waltz16
PCPD2              80.00 usec
PLW2          4.64209986 W
PLW12         0.10445000 W
PLW13         0.05245300 W

F2 - Processing parameters
SI                131072
SF           100.6555128 MHz
WDW                   EM
SSB                    0
LB                     0 Hz
GB                     0
PC                  1.40
```

The $^{13}C\{^{1}H\}$ NMR Spectrum of the Substitution Reaction of Acetal **21b** with Allyltributylstannane

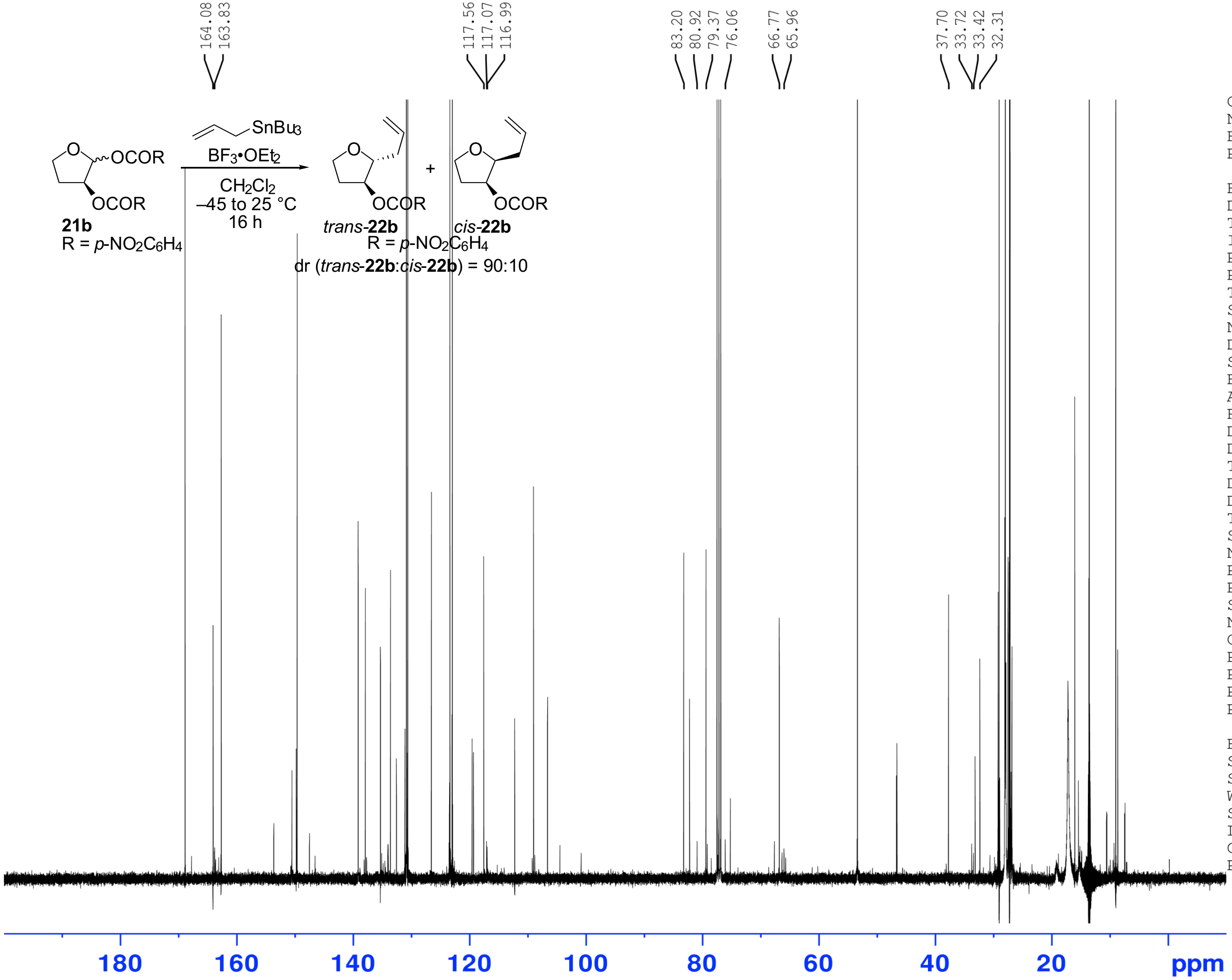

Current Data Parameters
NAME YC-3-136-CRUDE
EXPNO 2
PROCNO 1

F2 - Acquisition Parameters
Date_ 20230126
Time 6.51 h
INSTRUM spect
PROBHD Z150354_0001 (
PULPROG zgpg30
TD 65356
SOLVENT CDCl3
NS 128
DS 4
SWH 24038.461 Hz
FIDRES 0.735616 Hz
AQ 1.3594048 sec
RG 51.78
DW 20.800 usec
DE 25.00 usec
TE 298.0 K
D1 2.00000000 sec
D11 0.03000000 sec
TD0 1
SFO1 100.6655806 MHz
NUC1 13C
P1 10.00 usec
PLW1 17.99699974 W
SFO2 400.3016012 MHz
NUC2 1H
CPDPRG[2 waltz16
PCPD2 80.00 usec
PLW2 4.64209986 W
PLW12 0.10445000 W
PLW13 0.05245300 W

F2 - Processing parameters
SI 131072
SF 100.6555163 MHz
WDW EM
SSB 0
LB 0 Hz
GB 0
PC 1.40

The $^{13}C\{^1H\}$ NMR Spectrum of the Substitution Reaction of Acetal **21a** with Allyltributylstannane

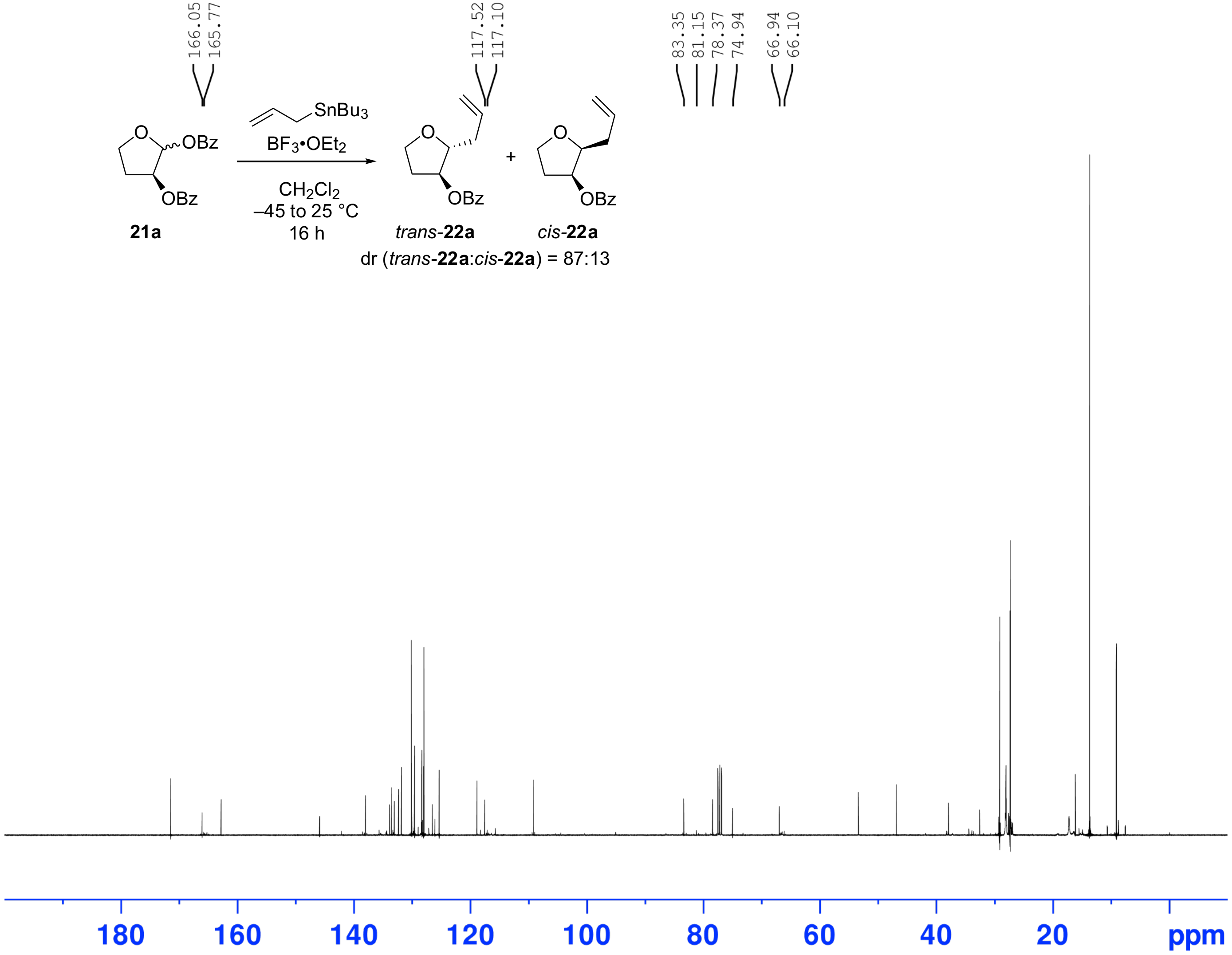


```
Current Data Parameters
NAME       YC-3-135-CRUDE
EXPNO                   2
PROCNO                  1

F2 - Acquisition Parameters
Date_            20230126
Time                 5.57 h
INSTRUM             spect
PROBHD      Z150354_0001 (
PULPROG           zgpg30
TD                 65356
SOLVENT            CDCl3
NS                   128
DS                     4
SWH            24038.461 Hz
FIDRES          0.735616 Hz
AQ             1.3594048 sec
RG                 25.77
DW                20.800 usec
DE                 25.00 usec
TE                 298.0 K
D1            2.00000000 sec
D11           0.03000000 sec
TD0                    1
SFO1         100.6655806 MHz
NUC1                 13C
P1                 10.00 usec
PLW1         17.99699974 W
SFO2         400.3016012 MHz
NUC2                  1H
CPDPRG[2         waltz16
PCPD2              80.00 usec
PLW2          4.64209986 W
PLW12         0.10445000 W
PLW13         0.05245300 W

F2 - Processing parameters
SI                131072
SF           100.6555140 MHz
WDW                   EM
SSB                    0
LB                     0 Hz
GB                     0
PC                  1.40
```

The $^{13}C\{^{1}H\}$ NMR Spectrum of the Substitution Reaction of Acetal **21b** with Allyltrimethylsilane

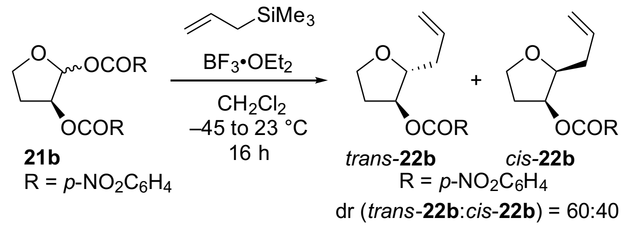

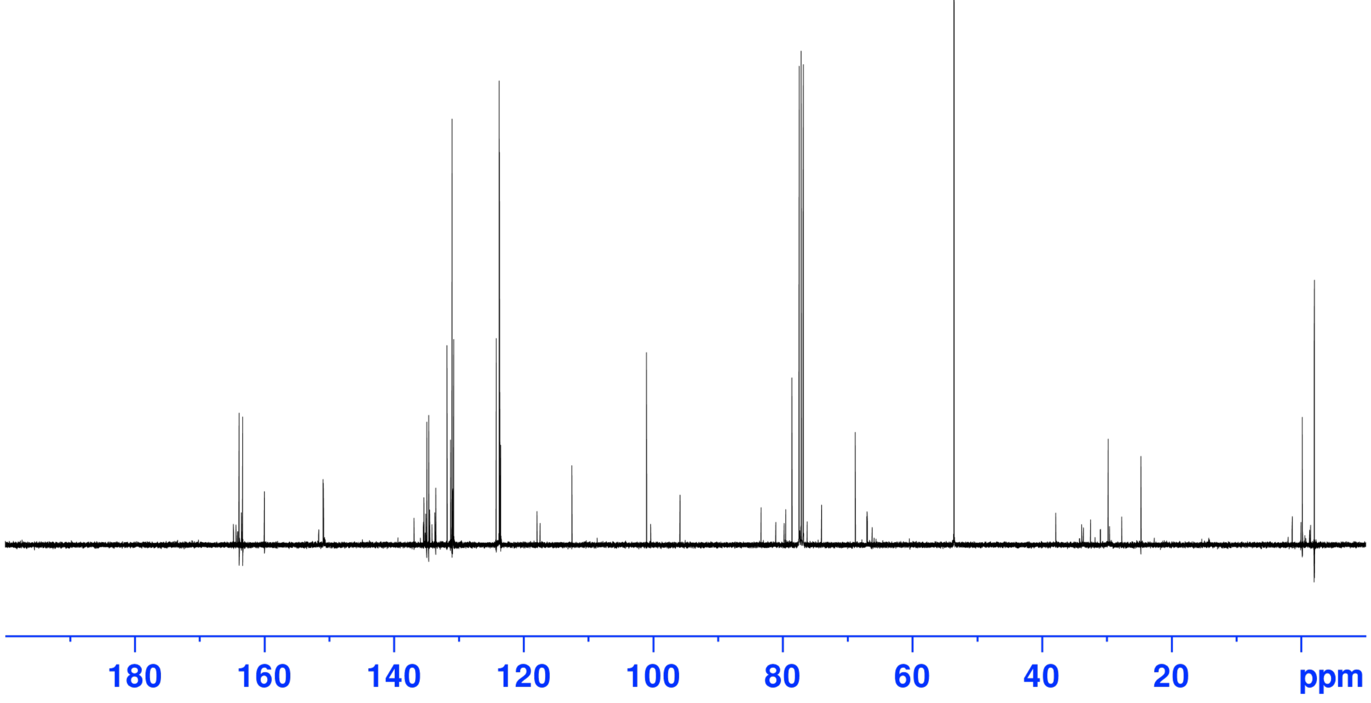


```
Current Data Parameters
NAME       YC-3-141-CRUDE
EXPNO                   2
PROCNO                  1

F2 - Acquisition Parameters
Date_            20230129
Time                 7.20 h
INSTRUM             spect
PROBHD    Z150354_0001 (
PULPROG            zgpg30
TD                  65356
SOLVENT             CDCl3
NS                    128
DS                      4
SWH             24038.461 Hz
FIDRES           0.735616 Hz
AQ              1.3594048 sec
RG                  25.77
DW                 20.800 usec
DE                  25.00 usec
TE                  298.0 K
D1             2.00000000 sec
D11            0.03000000 sec
TD0                     1
SFO1          100.6655806 MHz
NUC1                  13C
P1                  10.00 usec
PLW1          17.99699974 W
SFO2          400.3016012 MHz
NUC2                   1H
CPDPRG[2          waltz16
PCPD2               80.00 usec
PLW2           4.64209986 W
PLW12          0.10445000 W
PLW13          0.05245300 W

F2 - Processing parameters
SI                 131072
SF            100.6555080 MHz
WDW                    EM
SSB                     0
LB                      0 Hz
GB                      0
PC                   1.40
```

The $^{13}C\{^1H\}$ NMR Spectrum of the Substitution Reaction of Acetal **21b** with Allyltrimethylsilane

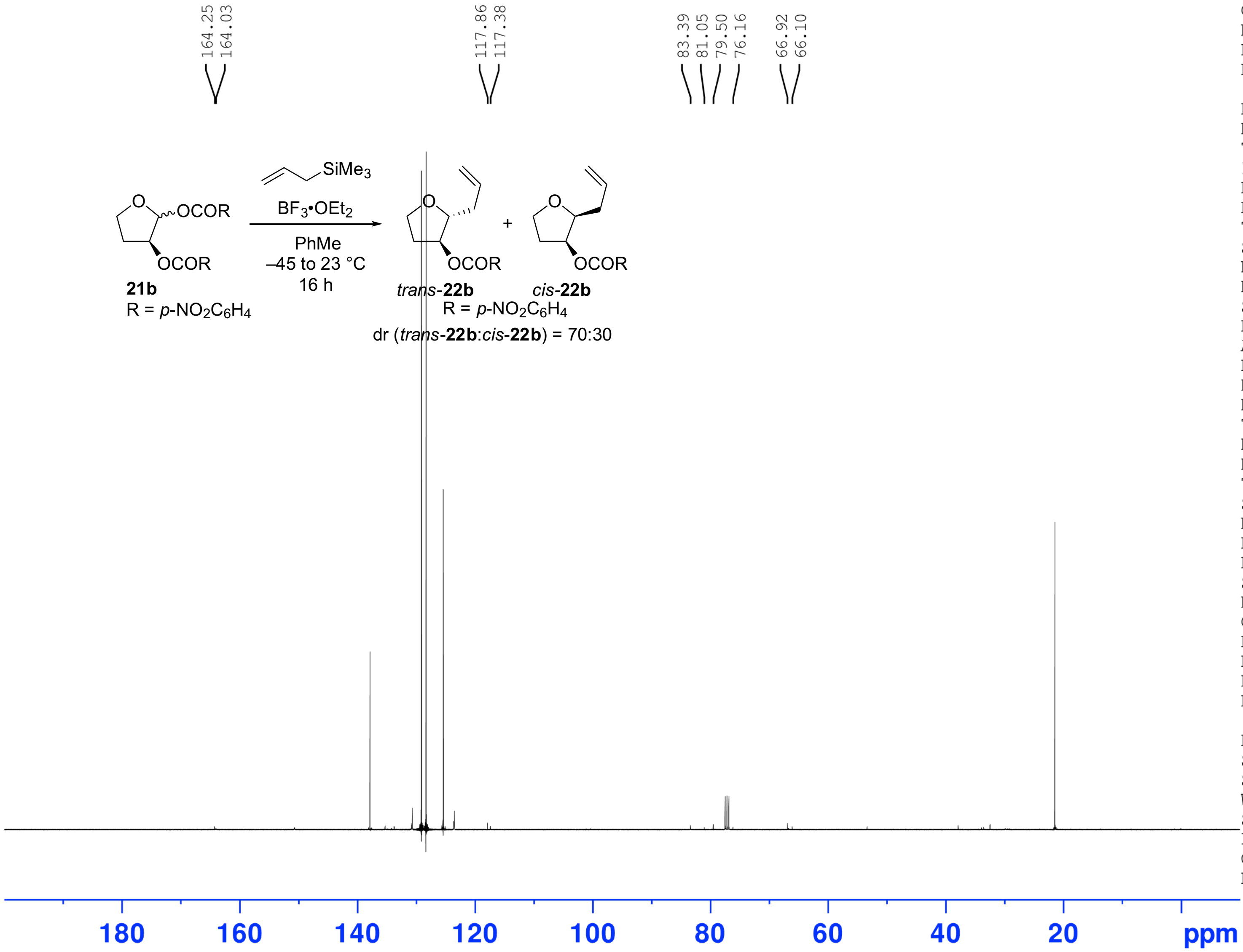


```
Current Data Parameters
NAME       YC-4-130-CRUDE
EXPNO                   2
PROCNO                  1

F2 - Acquisition Parameters
Date_            20240224
Time                7.15 h
INSTRUM             spect
PROBHD    Z150354_0001 (
PULPROG           zgpg30
TD                 65356
SOLVENT            CDCl3
NS                   128
DS                     4
SWH            24038.461 Hz
FIDRES          0.735616 Hz
AQ             1.3594048 sec
RG                 45.21
DW                20.800 usec
DE                 25.00 usec
TE                 298.0 K
D1            2.00000000 sec
D11           0.03000000 sec
TD0                    1
SFO1         100.6655806 MHz
NUC1                 13C
P1                 10.00 usec
PLW1         18.70700073 W
SFO2         400.3016012 MHz
NUC2                  1H
CPDPRG[2         waltz16
PCPD2              80.00 usec
PLW2          4.64209986 W
PLW12         0.10445000 W
PLW13         0.05245300 W

F2 - Processing parameters
SI                131072
SF           100.6555405 MHz
WDW                   EM
SSB                    0
LB                     0 Hz
GB                     0
PC                  1.40
```

The $^{13}C\{^{1}H\}$ NMR Spectrum of the Substitution Reaction of Acetal **21b** with Allyltrimethylsilane

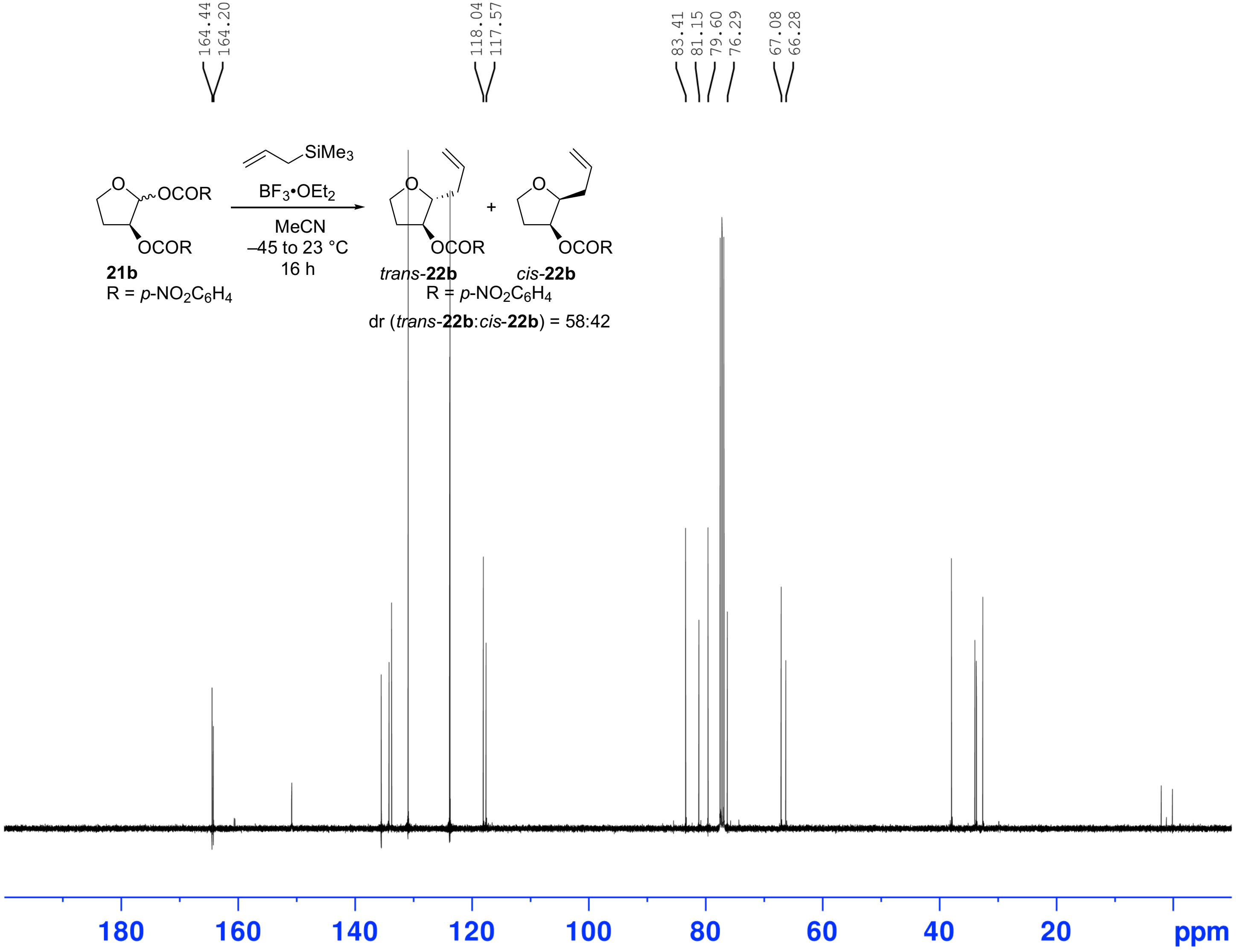


```
Current Data Parameters
NAME      YC-4-131-CRUDE
EXPNO                  2
PROCNO                 1

F2 - Acquisition Parameters
Date_           20240224
Time                7.40 h
INSTRUM            spect
PROBHD     Z150354_0001 (
PULPROG          zgpg30
TD                65356
SOLVENT           CDCl3
NS                  128
DS                    4
SWH           24038.461 Hz
FIDRES         0.735616 Hz
AQ            1.3594048 sec
RG                29.21
DW               20.800 usec
DE                25.00 usec
TE                298.0 K
D1           2.00000000 sec
D11          0.03000000 sec
TD0                   1
SFO1        100.6655806 MHz
NUC1                13C
P1                10.00 usec
PLW1        18.70700073 W
SFO2        400.3016012 MHz
NUC2                 1H
CPDPRG[2         waltz16
PCPD2             80.00 usec
PLW2         4.64209986 W
PLW12        0.10445000 W
PLW13        0.05245300 W

F2 - Processing parameters
SI               131072
SF          100.6555015 MHz
WDW                  EM
SSB                   0
LB                    0 Hz
GB                    0
PC                 1.40
```

The $^{13}C\{^{1}H\}$ NMR Spectrum of the Epimerization Nucleophilic Substitution Reaction of Acetals **10a** and Orthoester **16a**

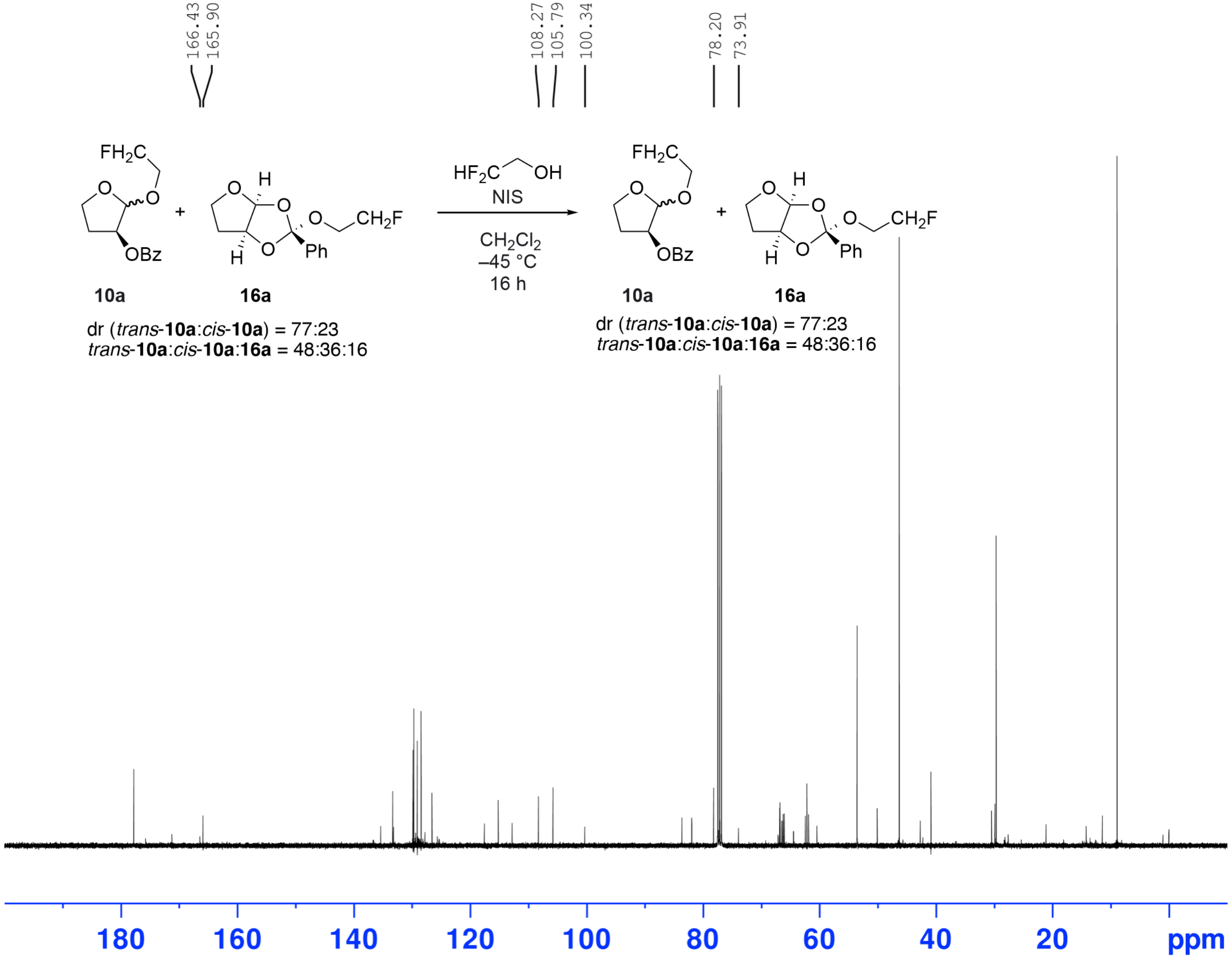


```
Current Data Parameters
NAME      YC-4-156-CRUDE
EXPNO                  2
PROCNO                 1

F2 - Acquisition Parameters
Date_           20240406
Time                3.57 h
INSTRUM            spect
PROBHD     Z150354_0001 (
PULPROG           zgpg30
TD                 65356
SOLVENT            CDCl3
NS                   128
DS                     4
SWH            24038.461 Hz
FIDRES          0.735616 Hz
AQ             1.3594048 sec
RG                 51.78
DW                20.800 usec
DE                 25.00 usec
TE                 298.0 K
D1            2.00000000 sec
D11           0.03000000 sec
TD0                    1
SFO1         100.6655806 MHz
NUC1                 13C
P1                 10.00 usec
PLW1         18.70700073 W
SFO2         400.3016012 MHz
NUC2                  1H
CPDPRG[2         waltz16
PCPD2              80.00 usec
PLW2          4.64209986 W
PLW12         0.10445000 W
PLW13         0.05245300 W

F2 - Processing parameters
SI                131072
SF           100.6555111 MHz
WDW                   EM
SSB                    0
LB                     0 Hz
GB                     0
PC                  1.40
```

The $^{13}C\{^{1}H\}$ NMR Spectrum of the Epimerization Nucleophilic Substitution Reaction of Acetal *trans*-**11a** and Acetal *cis*-**11a**

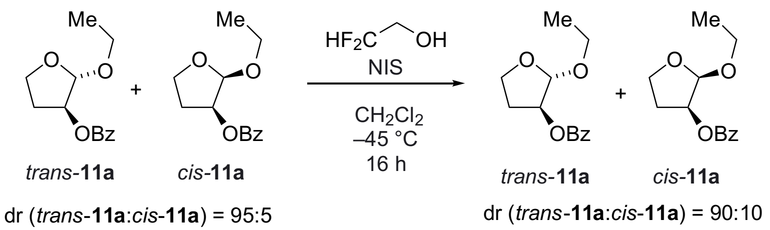

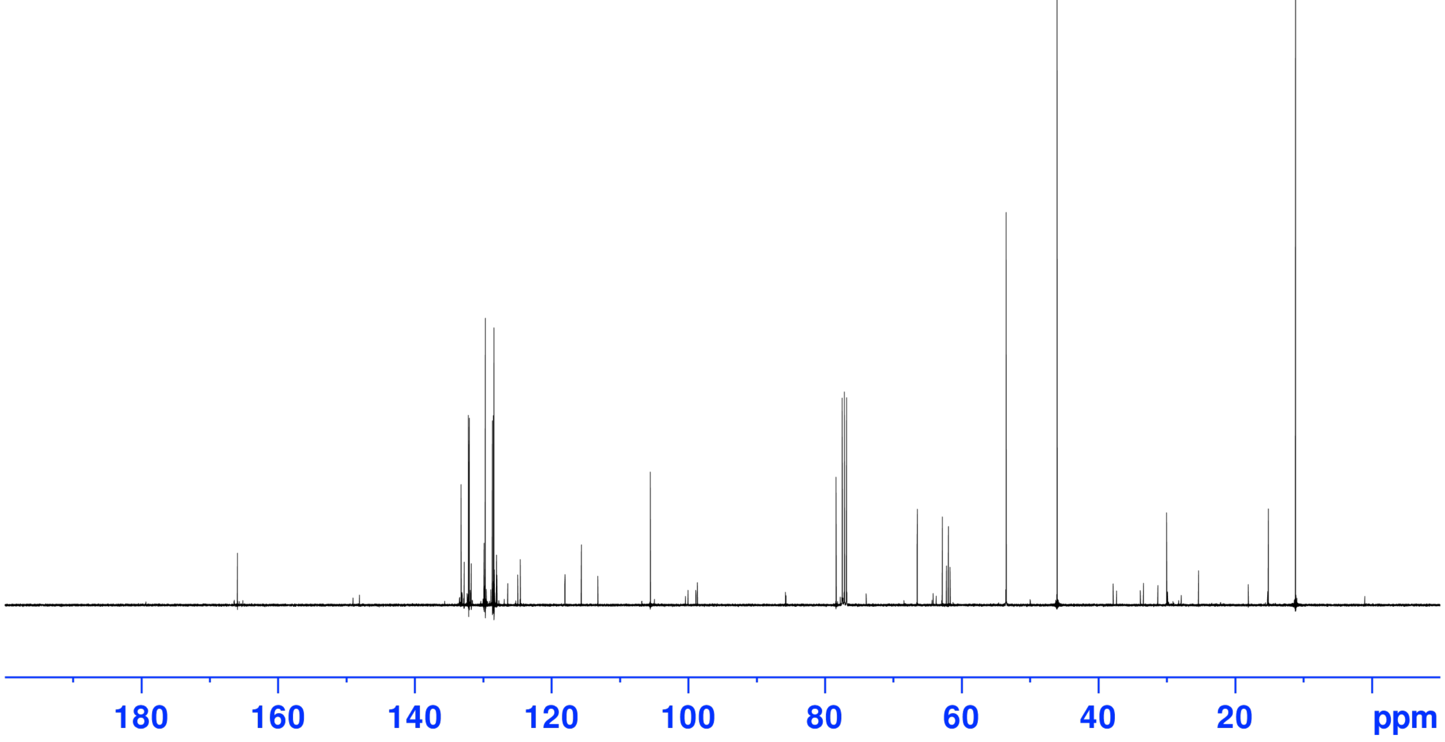

Current Data Parameters
NAME YC-4-178-CRUDE
EXPNO 3
PROCNO 1

F2 - Acquisition Parameters
Date_ 20240523
Time 2.30 h
INSTRUM spect
PROBHD Z150354_0001 (
PULPROG zgpg30
TD 65356
SOLVENT CDCl3
NS 128
DS 4
SWH 24038.461 Hz
FIDRES 0.735616 Hz
AQ 1.3594048 sec
RG 36.68
DW 20.800 usec
DE 25.00 usec
TE 298.0 K
D1 2.00000000 sec
D11 0.03000000 sec
TD0 1
SFO1 100.6655806 MHz
NUC1 13C
P1 10.00 usec
PLW1 18.70700073 W
SFO2 400.3016012 MHz
NUC2 1H
CPDPRG[2 waltz16
PCPD2 80.00 usec
PLW2 4.64209986 W
PLW12 0.10445000 W
PLW13 0.05245300 W

F2 - Processing parameters
SI 131072
SF 100.6555084 MHz
WDW EM
SSB 0
LB 0 Hz
GB 0
PC 1.40

The $^{13}C\{^{1}H\}$ NMR Spectrum of the Epimerization Nucleophilic Substitution Reaction of Acetal *trans*-**12a** and Acetal *cis*-**12a**

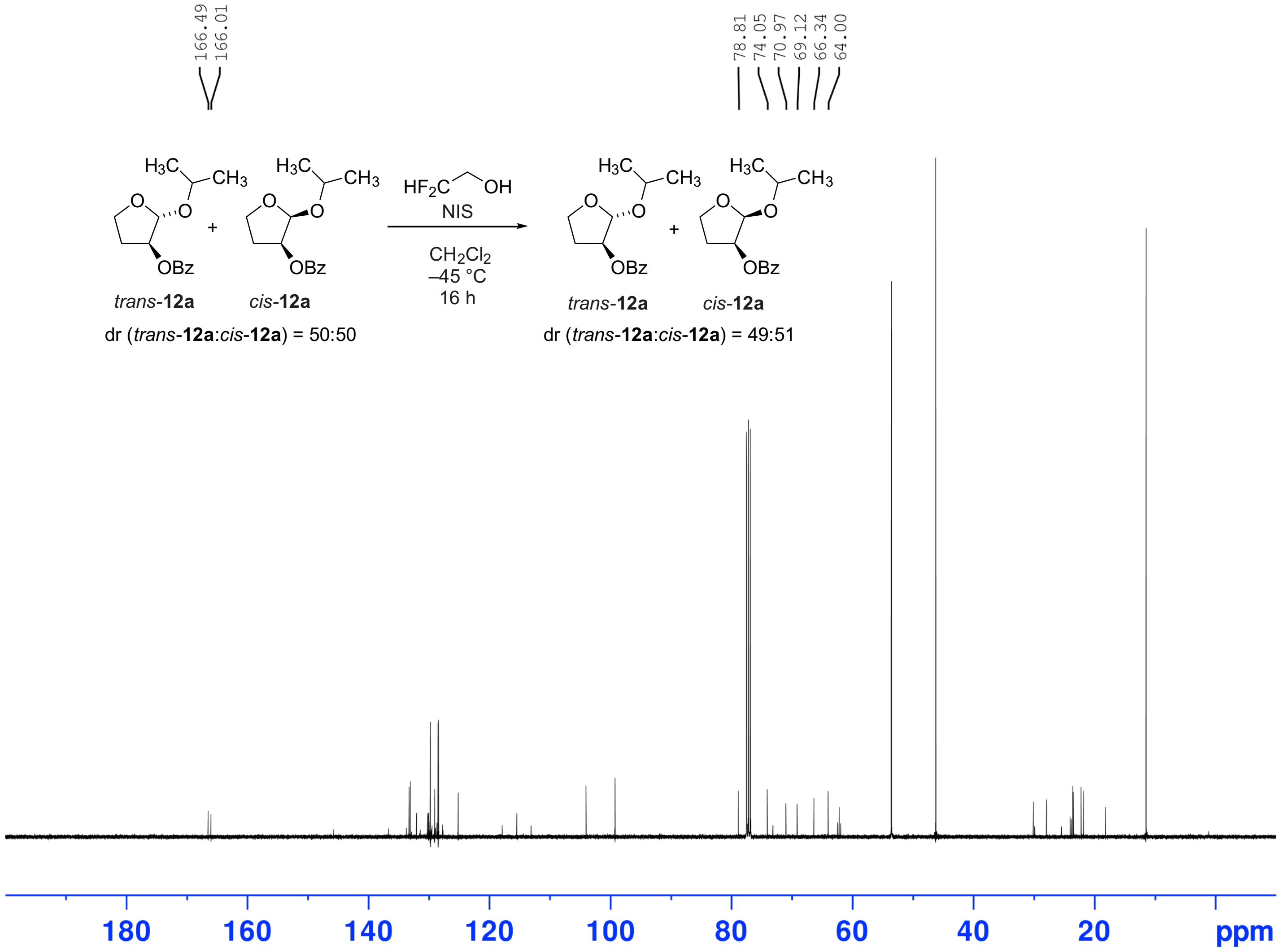


```
Current Data Parameters
NAME       YC-4-179-CRUDE
EXPNO                   2
PROCNO                  1

F2 - Acquisition Parameters
Date_            20240523
Time                 2.42 h
INSTRUM             spect
PROBHD     Z150354_0001 (
PULPROG           zgpg30
TD                 65356
SOLVENT            CDCl3
NS                   128
DS                     4
SWH            24038.461 Hz
FIDRES          0.735616 Hz
AQ             1.3594048 sec
RG                 45.21
DW                20.800 usec
DE                 25.00 usec
TE                 298.0 K
D1            2.00000000 sec
D11           0.03000000 sec
TD0                    1
SFO1         100.6655806 MHz
NUC1                 13C
P1                 10.00 usec
PLW1         18.70700073 W
SFO2         400.3016012 MHz
NUC2                  1H
CPDPRG[2         waltz16
PCPD2              80.00 usec
PLW2          4.64209986 W
PLW12         0.10445000 W
PLW13         0.05245300 W

F2 - Processing parameters
SI                131072
SF           100.6555055 MHz
WDW                   EM
SSB                    0
LB                     0 Hz
GB                     0
PC                  1.40
```

The $^{13}C\{^1H\}$ NMR Spectrum of the Epimerization Nucleophilic Substitution Reaction of Acetal *trans*-**13a**

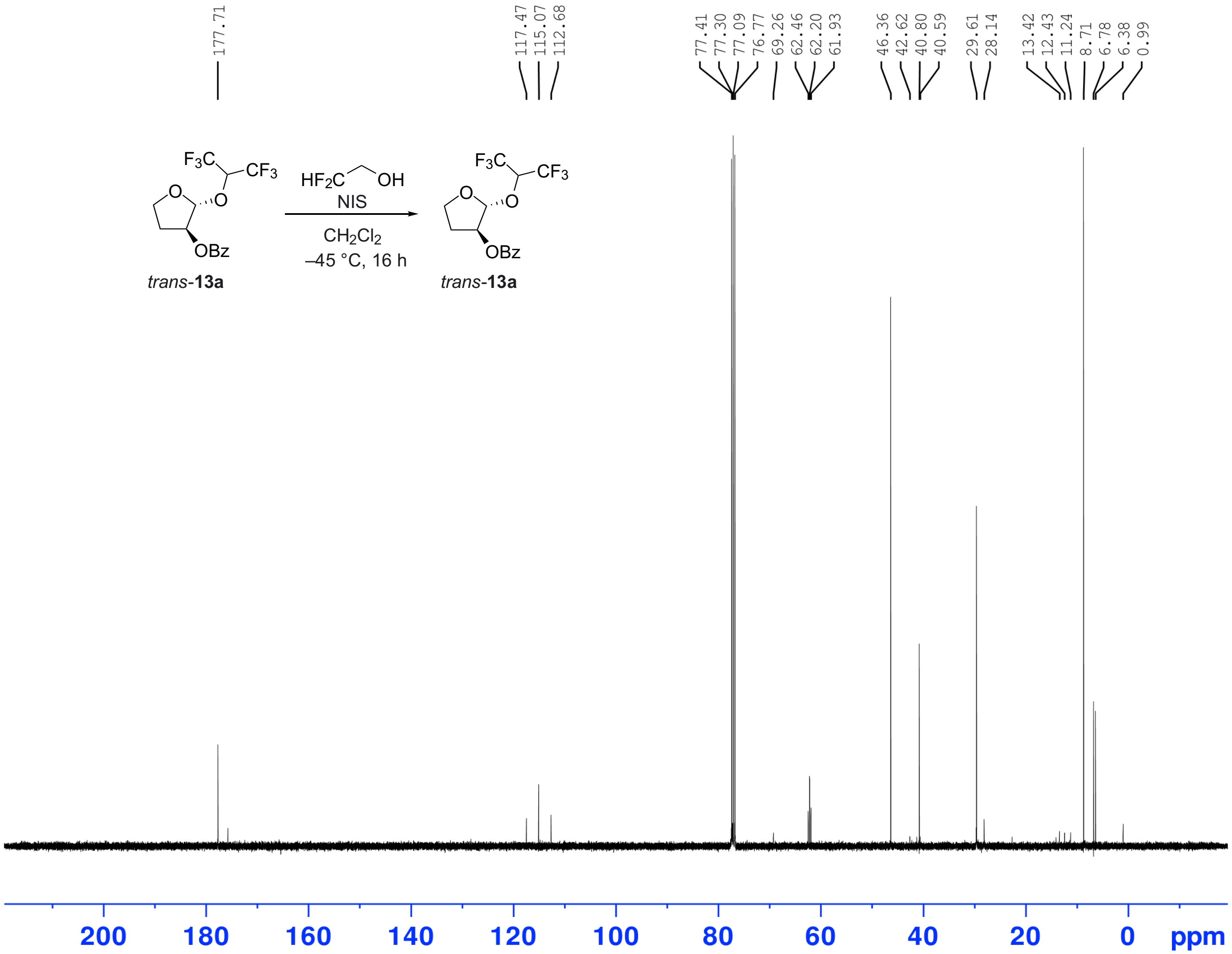

Current Data Parameters
NAME YC-4-158-CRUDE
EXPNO 2
PROCNO 1

F2 - Acquisition Parameters
Date_ 20240417
Time 4.56 h
INSTRUM spect
PROBHD Z150354_0001 (
PULPROG zgpg30
TD 65356
SOLVENT CDCl3
NS 128
DS 4
SWH 24038.461 Hz
FIDRES 0.735616 Hz
AQ 1.3594048 sec
RG 45.21
DW 20.800 usec
DE 25.00 usec
TE 298.0 K
D1 2.00000000 sec
D11 0.03000000 sec
TD0 1
SFO1 100.6655806 MHz
NUC1 13C
P1 10.00 usec
PLW1 18.70700073 W
SFO2 400.3016012 MHz
NUC2 1H
CPDPRG[2 waltz16
PCPD2 80.00 usec
PLW2 4.64209986 W
PLW12 0.10445000 W
PLW13 0.05245300 W

F2 - Processing parameters
SI 131072
SF 100.6555151 MHz
WDW EM
SSB 0
LB 0 Hz
GB 0
PC 1.40

The $^{13}C\{^1H\}$ NMR Spectrum of the Epimerization Nucleophilic Substitution Reaction of Acetal *trans*-**9b** and Orthoester **15b**

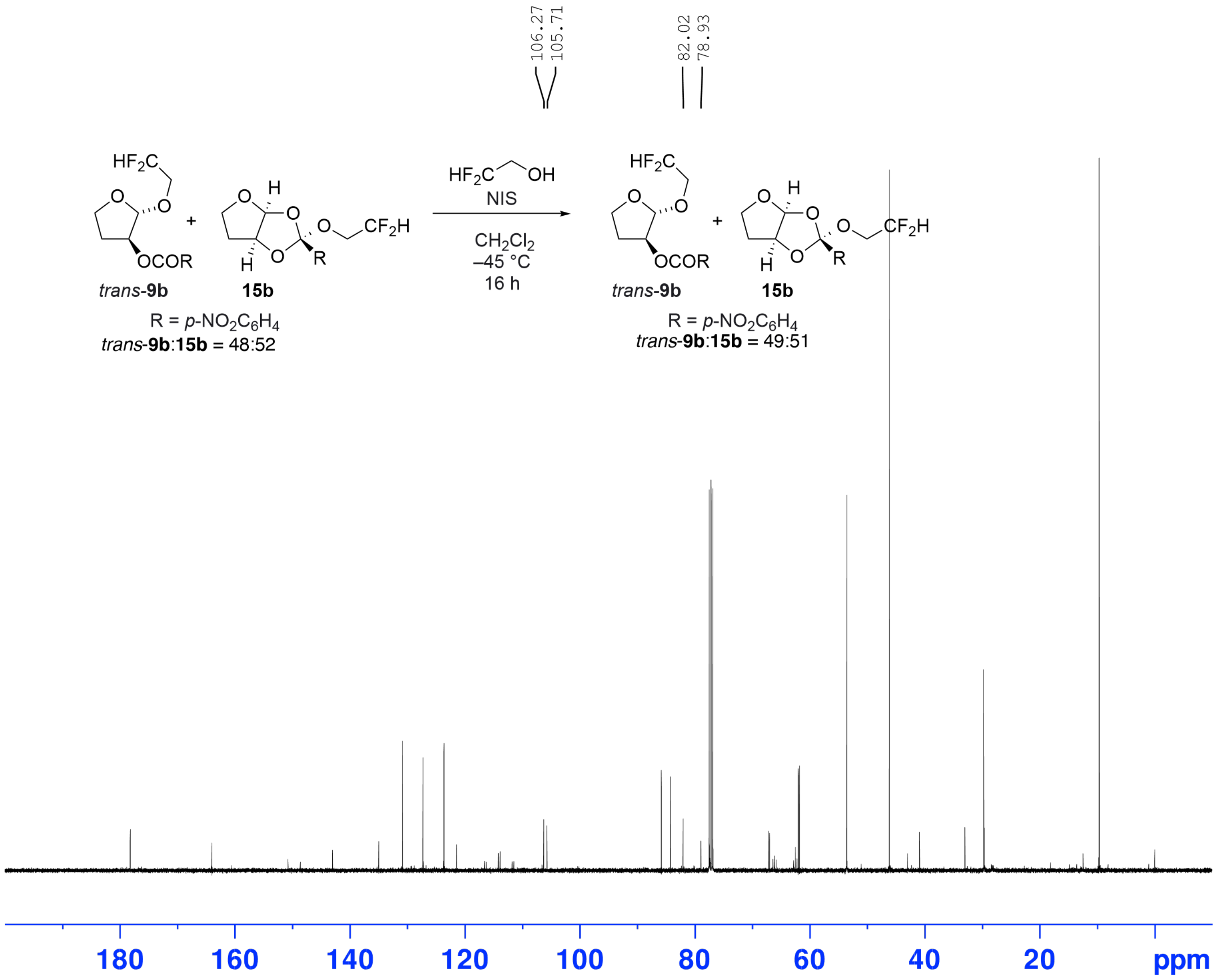


```
Current Data Parameters
NAME       YC-4-144-CRUDE
EXPNO                   2
PROCNO                  1

F2 - Acquisition Parameters
Date_            20240316
Time               7.07 h
INSTRUM             spect
PROBHD     Z150354_0001 (
PULPROG           zgpg30
TD                  65356
SOLVENT             CDCl3
NS                    128
DS                      4
SWH             24038.461 Hz
FIDRES           0.735616 Hz
AQ              1.3594048 sec
RG                  36.68
DW                 20.800 usec
DE                  25.00 usec
TE                  298.0 K
D1             2.00000000 sec
D11            0.03000000 sec
TD0                     1
SFO1          100.6655806 MHz
NUC1                  13C
P1                  10.00 usec
PLW1          18.70700073 W
SFO2          400.3016012 MHz
NUC2                   1H
CPDPRG[2          waltz16
PCPD2               80.00 usec
PLW2           4.64209986 W
PLW12          0.10445000 W
PLW13          0.05245300 W

F2 - Processing parameters
SI                 131072
SF            100.6555116 MHz
WDW                    EM
SSB                     0
LB                      0 Hz
GB                      0
PC                   1.40
```

The $^{13}C\{^{1}H\}$ NMR Spectrum of the Epimerization Nucleophilic Substitution Reaction of Acetal **10b** and Orthoester **16b**

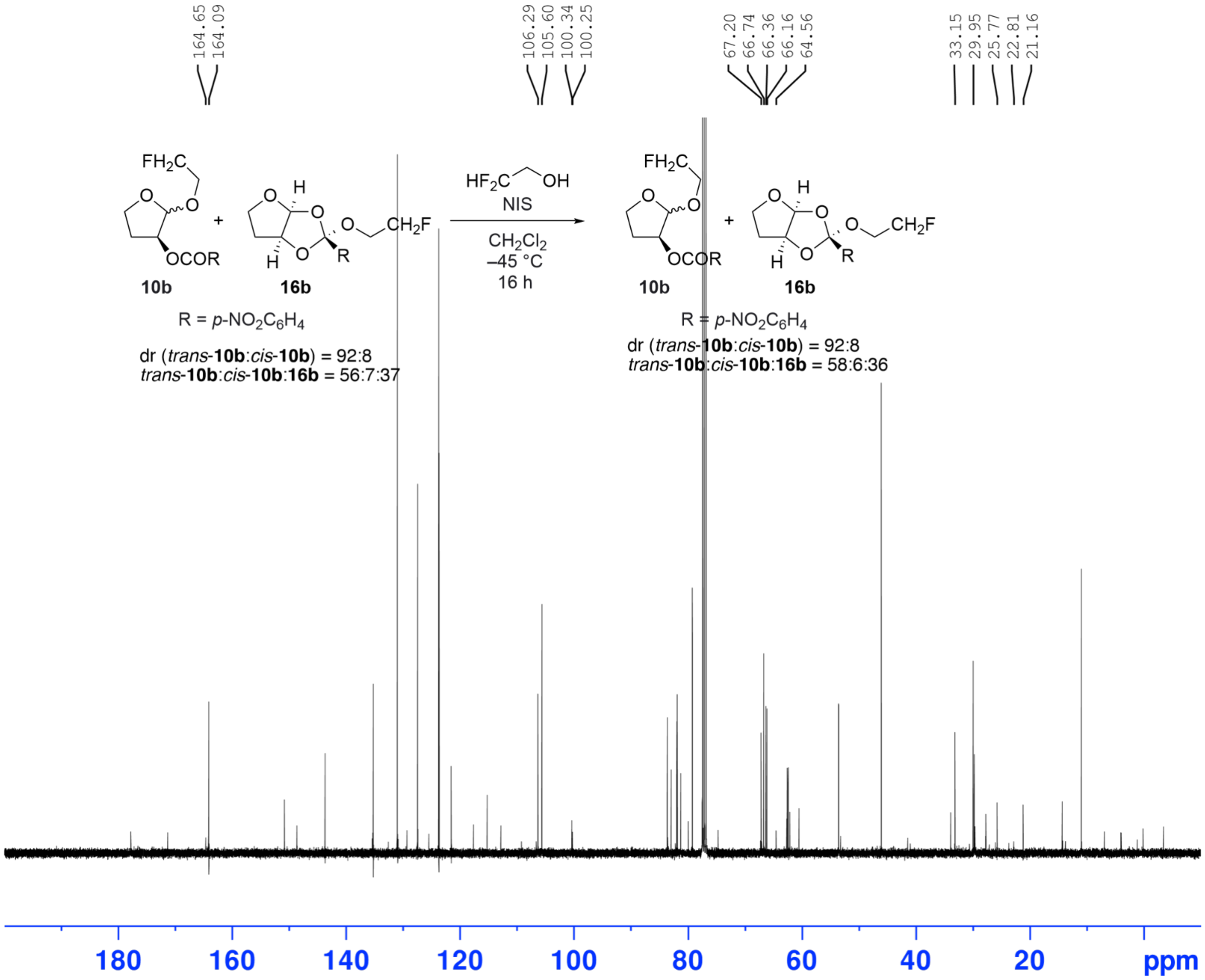


```
Current Data Parameters
NAME      YC-4-155-CRUDE
EXPNO                  2
PROCNO                 1

F2 - Acquisition Parameters
Date_           20240406
Time                3.44 h
INSTRUM            spect
PROBHD     Z150354_0001 (
PULPROG           zgpg30
TD                 65356
SOLVENT            CDCl3
NS                   128
DS                     4
SWH            24038.461 Hz
FIDRES          0.735616 Hz
AQ             1.3594048 sec
RG                 29.21
DW                20.800 usec
DE                 25.00 usec
TE                 298.0 K
D1            2.00000000 sec
D11           0.03000000 sec
TD0                    1
SFO1         100.6655806 MHz
NUC1                 13C
P1                 10.00 usec
PLW1         18.70700073 W
SFO2         400.3016012 MHz
NUC2                  1H
CPDPRG[2         waltz16
PCPD2              80.00 usec
PLW2          4.64209986 W
PLW12         0.10445000 W
PLW13         0.05245300 W

F2 - Processing parameters
SI                131072
SF           100.6555030 MHz
WDW                   EM
SSB                    0
LB                     0 Hz
GB                     0
PC                  1.40
```

The $^{13}C\{^{1}H\}$ NMR Spectrum of the Epimerization Nucleophilic Substitution Reaction of Acetal **11b** and Orthoester **17b**

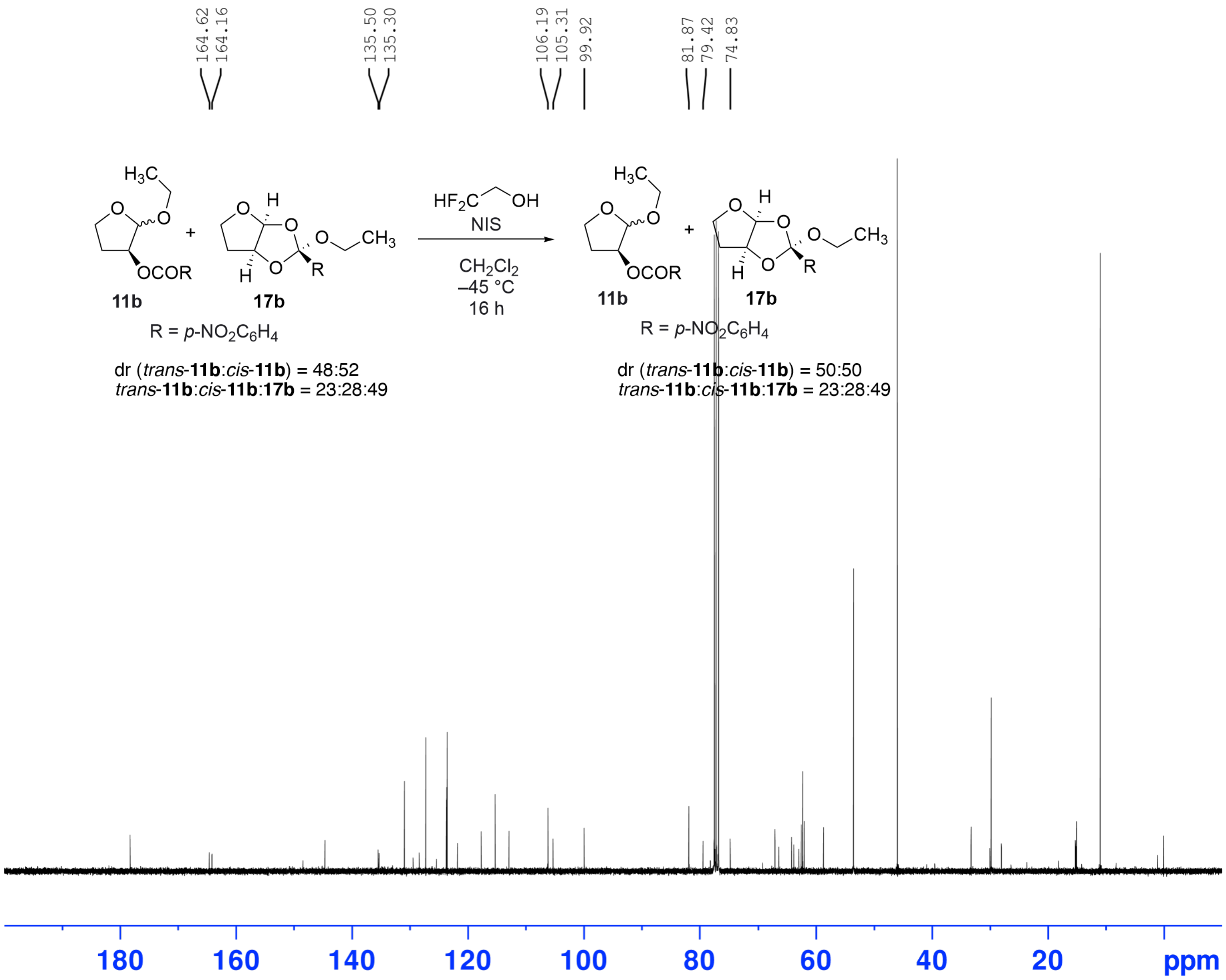


```
Current Data Parameters
NAME      YC-4-165-CRUDE
EXPNO                  2
PROCNO                 1

F2 - Acquisition Parameters
Date_           20240418
Time               23.31 h
INSTRUM            spect
PROBHD    Z150354_0001 (
PULPROG          zgpg30
TD                 65356
SOLVENT            CDCl3
NS                   128
DS                     4
SWH            24038.461 Hz
FIDRES          0.735616 Hz
AQ             1.3594048 sec
RG                 36.68
DW                20.800 usec
DE                 25.00 usec
TE                 298.0 K
D1            2.00000000 sec
D11           0.03000000 sec
TD0                    1
SFO1         100.6655806 MHz
NUC1                 13C
P1                 10.00 usec
PLW1         18.70700073 W
SFO2         400.3016012 MHz
NUC2                  1H
CPDPRG[2         waltz16
PCPD2              80.00 usec
PLW2          4.64209986 W
PLW12         0.10445000 W
PLW13         0.05245300 W

F2 - Processing parameters
SI                131072
SF           100.6555037 MHz
WDW                   EM
SSB                    0
LB                     0 Hz
GB                     0
PC                  1.40
```

The $^{13}C\{^{1}H\}$ NMR Spectrum of the Epimerization Nucleophilic Substitution Reaction of Acetal **12b** and Orthoester **18b**

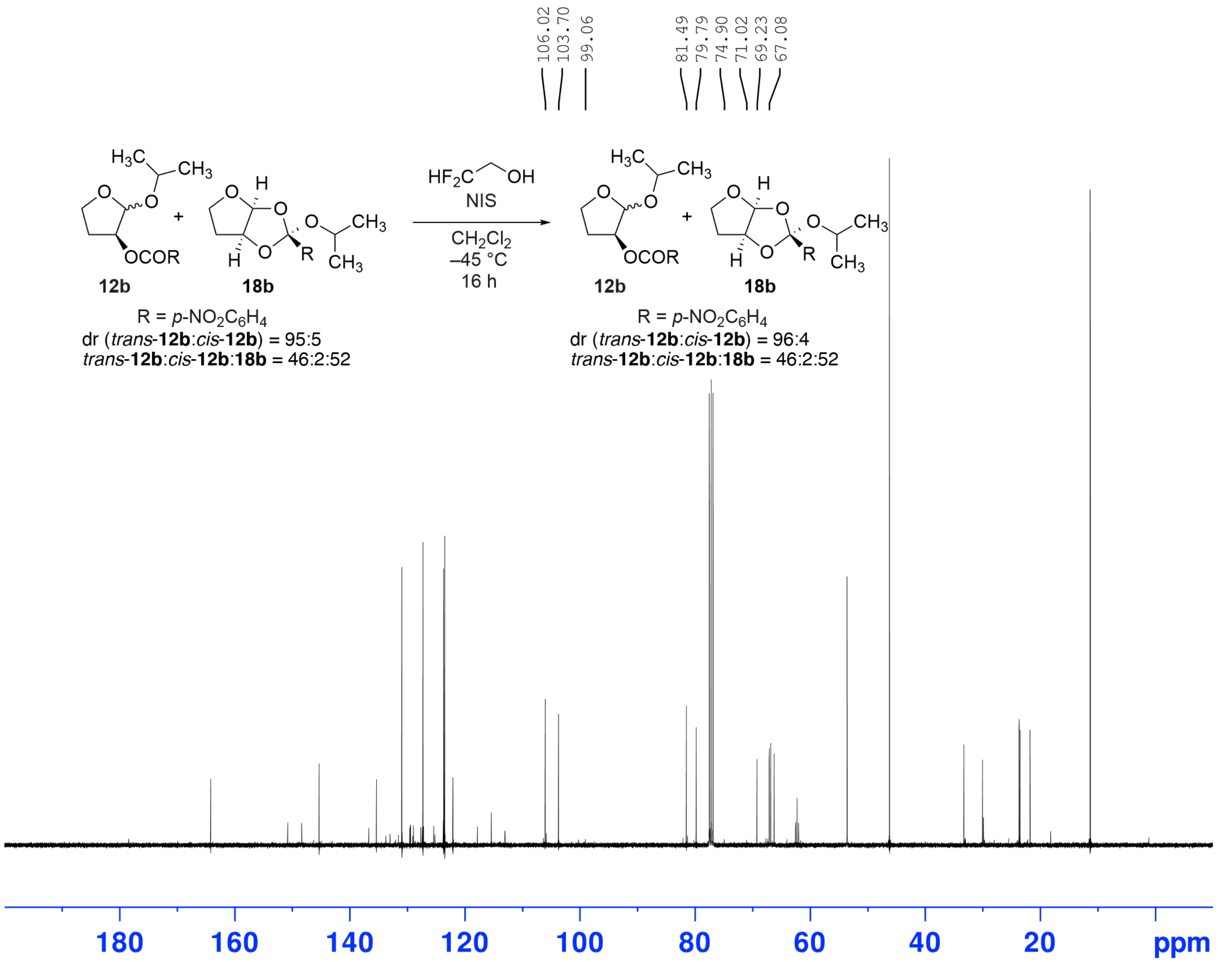


```
Current Data Parameters
NAME      YC-4-166-CRUDE
EXPNO                  2
PROCNO                 1

F2 - Acquisition Parameters
Date_           20240523
Time                2.09 h
INSTRUM            spect
PROBHD    Z150354_0001 (
PULPROG           zgpg30
TD                 65356
SOLVENT            CDCl3
NS                   128
DS                     4
SWH           24038.461 Hz
FIDRES         0.735616 Hz
AQ            1.3594048 sec
RG                 36.68
DW                20.800 usec
DE                 25.00 usec
TE                 298.0 K
D1            2.00000000 sec
D11           0.03000000 sec
TD0                    1
SFO1         100.6655806 MHz
NUC1                 13C
P1                 10.00 usec
PLW1         18.70700073 W
SFO2         400.3016012 MHz
NUC2                  1H
CPDPRG[2         waltz16
PCPD2              80.00 usec
PLW2          4.64209986 W
PLW12         0.10445000 W
PLW13         0.05245300 W

F2 - Processing parameters
SI                131072
SF           100.6555036 MHz
WDW                   EM
SSB                    0
LB                     0 Hz
GB                     0
PC                  1.40
```

The $^{13}C\{^1H\}$ NMR Spectrum of the Epimerization Nucleophilic Substitution Reaction of Acetal *trans*-**13b**

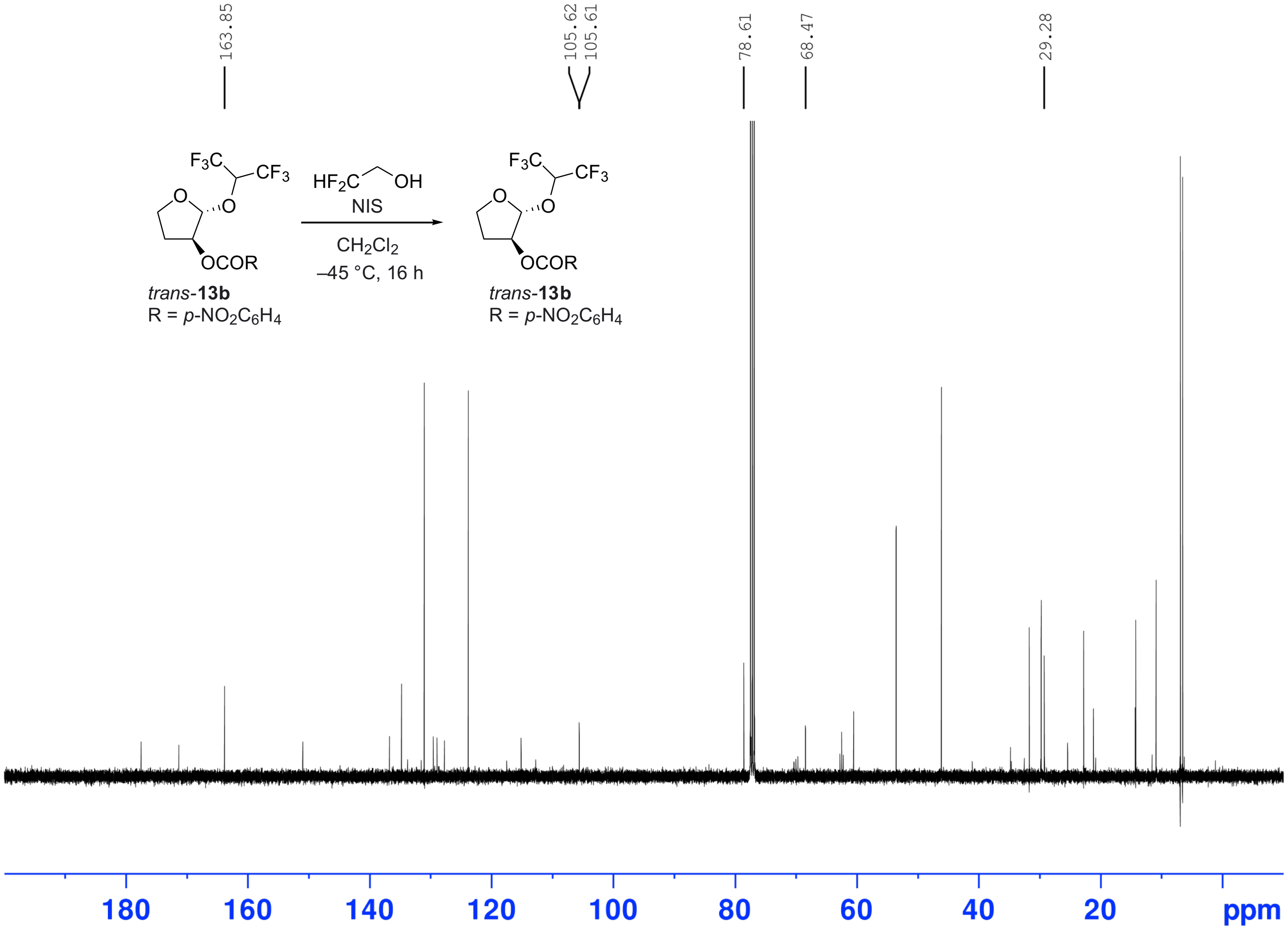

Current Data Parameters
NAME YC-4-157-CRUDE
EXPNO 3
PROCNO 1

F2 - Acquisition Parameters
Date_ 20240417
Time 4.43 h
INSTRUM spect
PROBHD Z150354_0001 (
PULPROG zgpg30
TD 65356
SOLVENT CDCl3
NS 128
DS 4
SWH 24038.461 Hz
FIDRES 0.735616 Hz
AQ 1.3594048 sec
RG 51.78
DW 20.800 usec
DE 25.00 usec
TE 298.0 K
D1 2.00000000 sec
D11 0.03000000 sec
TD0 1
SFO1 100.6655806 MHz
NUC1 13C
P1 10.00 usec
PLW1 18.70700073 W
SFO2 400.3016012 MHz
NUC2 1H
CPDPRG[2 waltz16
PCPD2 80.00 usec
PLW2 4.64209986 W
PLW12 0.10445000 W
PLW13 0.05245300 W

F2 - Processing parameters
SI 131072
SF 100.6554996 MHz
WDW EM
SSB 0
LB 0 Hz
GB 0
PC 1.40
